# Global, regional, and national burden of chronic obstructive pulmonary disease from 1990 to 2019

**DOI:** 10.3389/fphys.2022.925132

**Published:** 2022-08-09

**Authors:** Haifeng Wang, Xiaojuan Ye, Yafeng Zhang, Shiliang Ling

**Affiliations:** ^1^ Department of Hematology and Oncology, The People’s Hospital of Beilun District, Beilun Branch of the First Affiliated Hospital of Medical College of Zhejiang University, Ningbo, China; ^2^ Department of Oncology, Ningbo Hospital of Traditional Chinese Medicine, Ningbo, China

**Keywords:** COPD, incidence, mortality, disability-adjusted life years, global burden of disease

## Abstract

**Background:** We aimed to estimate the incidence, mortality, disability-adjusted life years (DALYs) for chronic obstructive pulmonary disease (COPD) in 204 countries and territories. We examined the variations in these trends by country, gender, age group, and sociodemographic index (SDI).

**Methods:** We calculated the estimated annual percentage changes (EAPCs) to assess temporal trends in the age-standardized incidence rate, age-standardized mortality rate, and age-standardized DALYs of COPD from 1990 to 2019.

**Results:** From 1990 to 2019, the COPD incidence and COPD-associated deaths and DALYs increased worldwide by 86%, 30%, and 26%, respectively. From 1990 to 2019, the global age-standardized incidence rate (EAPC, −0.11; 95% confidence interval (CI), −0.25 to 0.04), age-standardized mortality rate (EAPC, −2.10; 95% CI, −2.19 to −2.00), and age-standardized DALYs (EAPC, −1.87; 95% CI, −1.94 to −1.81) of COPD decreased. The age-standardized incidence of COPD increased most in areas with high SDI (EAPC 0.56). The largest increases in the age-standardized incidence rate of COPD were recorded in High-income North America (EAPC, 1.41), Southern Latin America (EAPC, 0.29), and North Africa and the Middle East (EAPC, 0.09). The three countries that recorded the largest increases in COPD incidence from 1990 to 2019 were the United States of America (EAPC, 1.51), Saudi Arabia (EAPC, 1.17), and Oman (EAPC, 1.10).

**Conclusion:** Despite the decreased burden of COPD globally from 1990 to 2019, the age-standardized incidence rate of COPD increased in areas with high SDI, High-income North America, Southern Latin America, North Africa, and the Middle East.

## Introduction

Chronic obstructive pulmonary disease (COPD) is a common and preventable chronic respiratory disease. COPD occurs when airflow in the airway is persistently obstructed due to abnormalities in the lungs that are caused by harmful gases or particles (Soriano et al., 2018). In 2012, the World Health Assembly proposed a new health target (the “25 by 25 goal”) that aimed to reduce the numbers of premature deaths caused by COPD and other noncommunicable diseases by 25% by 2025. Nonetheless, the morbidity, mortality, and disease burden of COPD has continued to increase globally. The global prevalence of COPD increased by 5.9% between 1990 and 2017 (GBD Chronic Respiratory Disease Collaborators, 2020), during which the disease was responsible for at least 2.9 million deaths each year. According to forecasts by the World Health Organization (WHO), COPD will become one of the three leading causes of death worldwide by 2030.

The incidence, death, and disease burden of COPD vary by gender, region, and age group. The prevalence of COPD increases with age in most regions, but in some regions such as Uganda the disease is more common among children and young adults (van Gemert et al., 2015). Although the prevalence of COPD is low in some Asian countries such as India, its associated mortality remains high (GBD 2013 Mortality and Causes of Death Collaborators, 2015). COPD imposes a greater burden of disease in low-income countries, where resources for healthcare are devoted to the management of acute diseases (such as infectious diseases) rather than chronic diseases. Assessing the burden of COPD and identifying high-risk groups at the regional and country levels can allow the common characteristics of high-risk areas and populations to be identified. Such knowledge can guide the rational allocation of health resources and the development of effective strategies for prevention and treatment.

To the best of our knowledge, there has been no systematic analysis of the morbidity, mortality, and disease burden of COPD using the latest data from the Global Burden of Disease (GBD) study. Here, we use data from GBD 2019 to estimate the rates of incidence, death, and disability-adjusted life years (DALYs) of COPD for 204 countries and territories, and examine the variations in these trends based on country, gender, age group, and sociodemographic index (SDI).

## Materials and methods

### Data source

Data on COPD from GBD 2019 were obtained using the Global Health Data Exchange (GHDx) query tool (http://ghdx.healthdata.org/GBD-results-tool). Following the instructions of the GBD 2019 online tool guide, we extracted the absolute sums and rates of morbidity, mortality, and DALYs of COPD between 1990 and 2019 by age, gender, SDI, region, and country. Details of the methods used to estimate the incidences of diseases and injuries, identify risk factors, and calculate disease burdens in the GBD 2019 have been presented previously (GBD 2019 Diseases, and Injuries Collaborators, 2020).

COPD is defined in the Global Initiative for Chronic Obstructive Pulmonary Disease (GOLD) classification as an FEV1/FVC (forced exhalation/one second of total forced exhalation) value of less than 0.7 for post-bronchiectasis spirometry. The codes for COPD in the International Classification of Diseases Code Version 10 (ICD-10) are D86–D86.2, D86.9, G47.3–G47.39, J30–J35.9, J37–J39.9, J41–J42.4, J43–J46.0, J47–J47.9, J60–J68.9, J70.8–J70.9, J80–J80.9, J82, J84–J84.9, J90–J90.0, J91, J91.8–J93.12, J93.8–J94.9, J96–J96.92, J98–J99.8, R05.0–R06.9, R09–R09.89, R84–R84.9, R91–R91.8, and Z82. The codes for COPD in the ICD-9 include: 135–135.9, 278.03, 327.2–327.29, 470, 470.9–474.9, 476–479, 491–508.9, 512–513, 514–518.53, 518.8–519, 519.11–519.9, 786–786.9, 793.1–793.2, 799.0–799.1, V07.1, V12.6–V12.60, V12.69, V13.81, V14–V15.09, V15.84, V17.5–V17.6, V19.6, V42.6, V43.81, V45.76, V58.74, and V81.3–V81.4.

The SDI is a composite index that reflects the level of social development in a country. It is calculated based on a country’s GDP per capita, fertility rate, and average years of schooling in the population. The SDI is an important variable for assessing the disease burden and level of health development in a region. The GBD classifies 204 countries and territories into five SDI quintiles (i.e., “low,” “low-middle,” “middle,” “high-middle,” and “high”) (GBD 2019 Demographics Collaborators, 2020).

GBD 2019 uses the world standard population to calculate age-standardized incidence, mortality, and DALYs rates. The calculation formula of the standardized rate is: age-standardized rate= (∑A i = 1ai wi)/(∑A i = 1wi) × 100,000, where ai represents the age-standardized rate of the *i*th age group, and w represents the number (or weight) of the same *i*th age group in the reference standard population, A represents the number of age groups. Number of cases refers to the quantity.

### Statistical analysis

We calculated age-standardized rates (ASRs) according to global standards (Ahmad et al., 2001). Specifically, we calculated each ASR (per 100,000 population) as follows:

$\mathrm{ASR}=\frac{\sum_{i=1}^{A}a_{1w_{i}}}{\sum_{i=1}^{A}w_{i}}\times 100,000$ where α_i_ is the age-specific rate in the *i*th age group, *w*_*i*_ is the population number (or weight) of the corresponding *i*th age subgroup in the population selected as the reference standard, and A is the number of age groups.

The EAPC is an established method for quantifying changes and describing trends in ASRs using a regression model (Hankey et al., 2000; Liu et al., 2019). The EAPC calculates the average annual rates of change in ASRs in all specified intervals. To estimate the EAPC, we used a linear regression: Y=α+βX+ε , where y = ln (age-standardized rate), and x = Gregorian calendar year. The EAPC was calculated by 100×[exp(β)−1] _,_ and the 95% confidence interval (CI) was calculated based on the linear regression. Positive values (i.e., >0) for EAPC and its 95% CI indicate that an ASR increased over time, while negative values (i.e., <0) indicate that an ASR decreased over time; all other values indicate that an ASR remained relatively stable over time.

## Results

### Chronic obstructive pulmonary disease incidence

**FIGURE 1:**
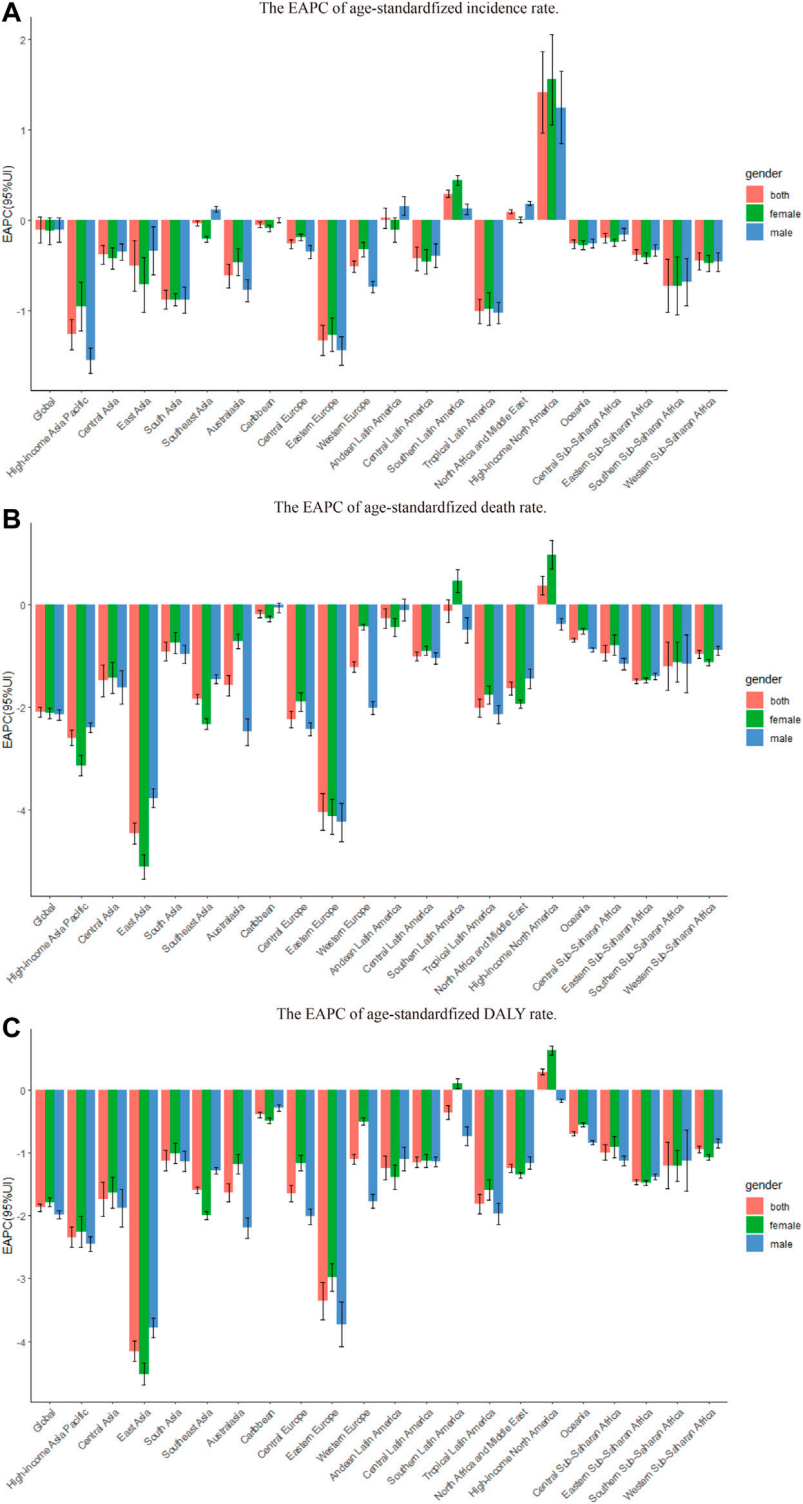
The EAPC of chronic obstructive pulmonary disease age-standardized rates from 1990 to 2019, by gender and region. **(A)** The EAPC of age-standardized incidence rate. **(B)** The EAPC of age standardized death rate. **(C)** The EAPC of age-standardized DALY rate. EAPC = estimated annual percentage change. DALY = disability adjusted life-year.

From 1990 to 2019, the global incidence rate of COPD increased by 86% from 8,722,965.84 to 16,214,828.28 (Table 1). The global age-standardized incidence rate of COPD displayed a consistent decreasing trend with an EAPC of -0.11 (95% CI, -0.25 to 0.04) (Table 1; Figure 1A; Supplementary Figure S1A). The age-standardized incidence rate of COPD in both gender decreased from 1990 to 2019 (male EAPC, −0.11; female EAPC, −0.12) (Table 1; Figure 1A). The age-standardized incidence rate was higher in males than in females (Table 1).

**TABLE 1:** The age-standardized incidence rate (ASIR) of chronic obstructive pulmonary disease in 1990–2019 and its temporal trends.

Characteristics	1990		2019		1990–2019	
	ASIR (per 100,000)		ASIR (per 100,000)		Percent change	EAPC
	No. (95% UI)	Male/female ratio	No. (95% UI)	Male/female ratio		No. (95% CI)
Global	1057.45 (1202.87, 942.35)	1.067590945	1001.57 (1144.44, 882.99)	1.062339899	0.49 (0.56, 0.42)	−0.11 (−0.25, 0.04)
gender	—	—	—	—	—	—
Male	1096.81 (1246.84, 974.43)	—	1034.12 (1187.41, 910.04)	—	0.48 (0.55, 0.41)	−0.11 (−0.24, 0.02)
Female	1027.37 (1166.39, 915.07)	—	973.44 (1108.64, 860.64)	—	0.50 (0.57, 0.44)	−0.12 (−0.27, 0.03)
Sociodemographic index	—	—	—	—	—	—
Low SDI	1047.71 (1184.30, 929.44)	0.985176926	961.70 (1094.95, 852.91)	0.884049947	0.85 (0.91, 0.79)	−0.12 (−0.24, −0.01)
Low-middle SDI	1096.34 (1220.24, 977.34)	1.025843737	1022.38 (1141.98, 907.93)	1.010102937	0.60 (0.68, 0.52)	−0.21 (−0.34, −0.08)
Middle SDI	968.05 (1111.93, 851.44)	1.079775235	908.19 (1050.90, 789.51)	1.037672657	0.44 (0.54, 0.35)	−0.09 (−0.22, 0.04)
High-middle SDI	998.71 (1151.72, 881.44)	1.099574828	880.44 (1029.04,759.84)	1.083055847	0.21 (0.27, 0.15)	−0.58 (−0.73, −0.42)
High SDI	1412.44 (1656.49, 1231.53)	1.042871843	1460.92 (1686.87, 1263.85)	0.988146748	0.42 (0.50, 0.35)	0.56 (0.31, 0.82)
Region	—	—	—	—	—	—
Andean latin america	1478.26 (1746.09, 1225.36)	0.928893732	1547.13 (1850.67, 1297.26)	0.974652673	0.56 (0.76, 0.37)	0.02 (−0.09, 0.14)
Australasia	1311.62 (1492.04, 1142.05)	1.274405278	1178.62 (1379.69, 1010.97)	1.180066819	0.54 (0.67, 0.42)	−0.62 (−0.74, −0.49)
Caribbean	1247.17 (1494.39, 1041.26)	0.913087654	1237.37 (1487.33, 1030.00)	0.938556072	0.20 (0.26, 0.15)	−0.05 (−0.08, −0.02)
Central asia	854.56 (965.53, 762.57)	1.100377309	794.68 (912.68, 696.91)	1.127933391	0.31 (0.35, 0.26)	−0.38 (−0.48, −0.28)
Central europe	1235.55 (1407.60, 1098.45)	1.116778138	1173.11 (1357.57, 1024.63)	1.073997711	−0.02 (0.03, −0.06)	−0.26 (−0.31, −0.21)
Central latin America	1184.97 (1387.02, 1012.10)	0.968743304	1096.95 (1301.24, 928.55)	0.98518579	0.38 (0.49, 0.29)	−0.42 (−0.56, −0.29)
Central sub-saharan africa	899.02 (1033.32, 779.32)	0.892135691	866.59 (991.77, 761.14)	0.916979832	1.10 (1.23, 0.96)	−0.20 (−0.25, −0.14)
East asia	864.92 (998.62, 757.65)	1.153086979	771.01 (910.73,661.24)	1.226626829	0.39 (0.51, 0.27)	−0.50 (−0.78, −0.22)
Eastern europe	1165.87 (1345.56, 1020.24)	1.120290655	866.65 (1035.50,731.93)	1.094255131	−0.31 (−0.28, −0.34)	−1.33 (−1.49, −1.16)
Eastern sub-saharan africa	1007.22 (1187.71, 865.61)	1.055315681	921.06 (1090.70, 780.42)	1.076450722	0.85 (0.93, 0.75)	−0.38 (−0.44, −0.32)
High-income asia pacific	1275.03 (1478.87, 1112.78)	1.185318148	940.10 (1123.60, 800.25)	1.146675472	0.07 (0.17, −0.01)	−1.26 (−1.43, −1.10)
High-income north america	2140.10 (2567.49, 1827.88)	0.982985632	2445.90 (2810.37, 2134.79)	0.955074094	0.66 (0.78, 0.53)	1.41 (0.97, 1.87)
North africa and middle east	954.24 (1095.51, 835.77)	0.921135185	1052.81 (1209.38, 924.34)	0.948629747	0.84 (0.97, 0.72)	0.09 (0.07, 0.12)
Oceania	1357.59 (1499.25, 1229.27)	1.043796579	1291.17 (1420.65, 1172.30)	1.057660191	0.95 (1.04, 0.86)	−0.26 (−0.31, −0.22)
South asia	1163.23 (1298.50, 1031.05)	0.986288053	1090.30 (1219.11, 966.85)	0.997707238	0.89 (0.98, 0.80)	−0.88 (−0.98, −0.77)
Southeast asia	851.55 (986.03, 749.18)	1.203180104	853.54 (996.49, 745.86)	1.29022744	0.45 (0.53,0.37)	−0.03 (−0.06,−0.01)
Southern latin america	1075.86 (1243.05, 949.42)	1.094037172	1169.03 (1388.03, 1005.45)	1.035832844	0.51 (0.60, 0.42)	0.29 (0.25, 0.33)
Southern sub-saharan africa	1138.88 (1307.32, 978.59)	1.102208251	1027.37 (1185.62, 884.74)	1.11138584	0.41 (0.48,0.35)	−0.72 (−1.02,−0.43)
Tropical latin america	1488.31 (1829.11, 1209.48)	0.984948226	1251.15 (1559.09, 980.72)	0.950505943	0.09 (0.16, 0.02)	−1.01 (−1.14, −0.87)
Western europe	878.30 (1004.69, 774.95)	1.051006899	768.49 (897.95, 652.01)	0.947420451	0.08 (0.13, 0.05)	−0.51(−0.58, −0.45)
Western sub-saharan africa	855.18 (997.78, 745.68)	0.95637404	778.52 (917.80, 673.19)	0.967062096	1.13 (1.18, 1.07)	−0.45 (−0.55, −0.36)
Abbreviations: ASIR, age-standardized incidence rate; EAPC, estimated annual percentage change; NA, not available; UI, uncertainty interval.						

The male-to-female ratio of COPD incidence peaked in the 75–79 (years) age group globally; it also peaked in this age group in high, high-middle, and low SDI regions. The ratio peaked in the 70–74 age group in middle and low-middle SDI regions (Supplementary Figure S2). The incidence of COPD increased with age globally, as well as in all SDI regions (Supplementary Figure S3).

**FIGURE 2:**
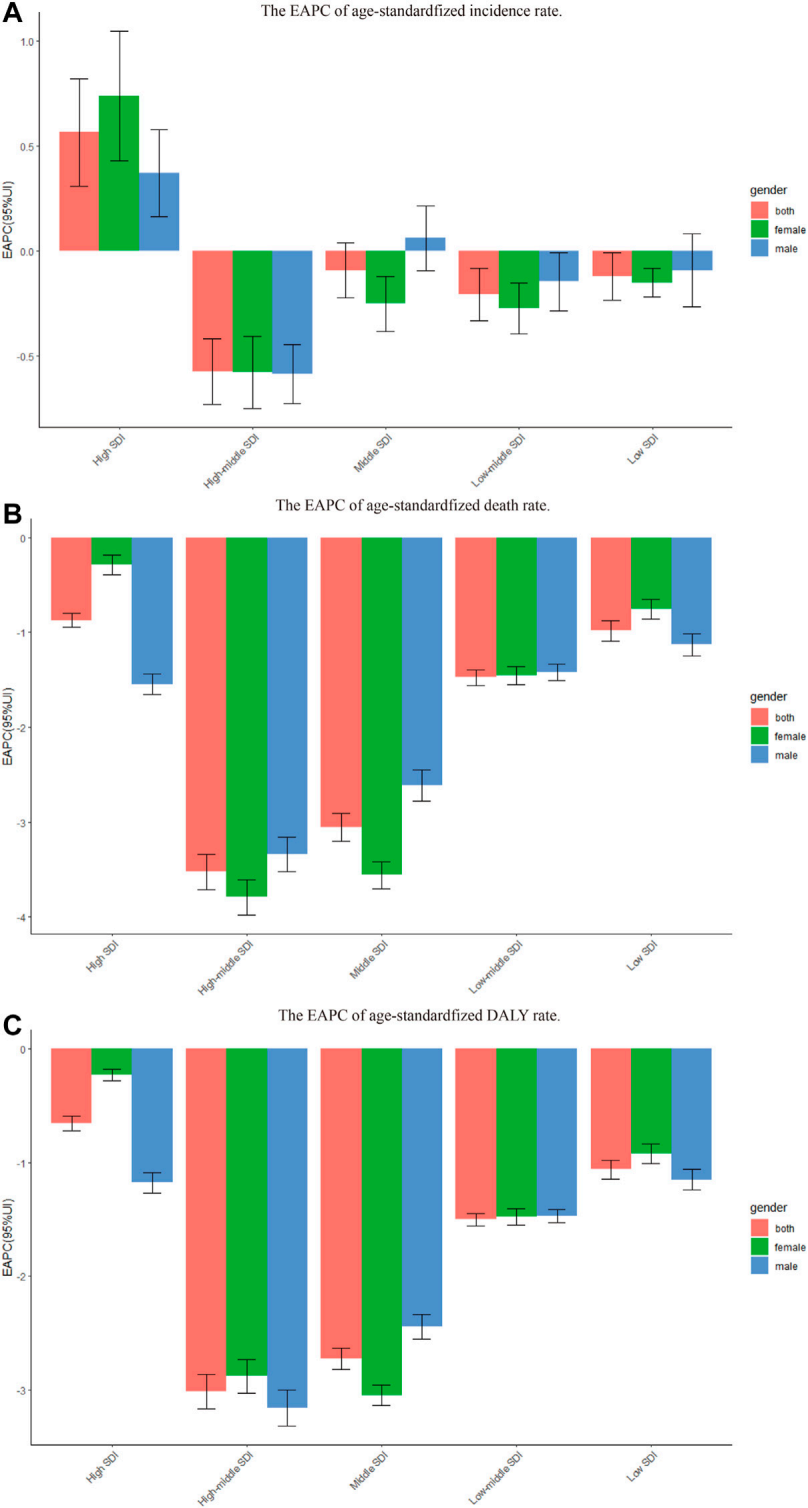
The EAPC of chronic obstructive pulmonary disease age-standardized rates from 1990 to 2019, by gender and SDI. **(A)** The EAPC of age-standardized incidence rate. **(B)** The EAPC of age standardized death rate. **(C)** The EAPC of age-standardized DALY rate. EAPC = estimated annual percentage change. DALY = disability adjusted life-year.

As shown in Table 1 and Supplementary Figure S2A, the age-standardized incidence of COPD was substantially higher in high SDI regions than in other regions. In 2019, high SDI regions (1460.92 per 100,000 population; 95% UI, 1263.85–1686.87) recorded the highest age-standardized incidence rates of COPD, followed by the low-middle SDI regions (1022.38 per 100,000 population; 95% UI, 907.93–1141.98). The age-standardized incidence rate increased most in the high SDI regions, where EAPC peaked at 0.56 (Table 1; Figure 2A).

The EAPC of the age-standardized incidence rate of COPD was negatively associated with both the age-standardized incidence rate (*ρ* = −0.111, *p* = 0.115) (Supplementary Figure S4A) and the SDI (*ρ* = −20.084, *p* = 0.230) (Supplementary Figure S4A). In 2019, middle SDI regions had the highest incidences of COPD among older adults (i.e., 70 + years), as did regions in which SDI increased between 1990 and 2019 (Supplementary Figures S5A,B). The annual incidence of COPD decreased among young people but increased among older adults (Supplementary Figure S6A).

In 2019, the highest regional age-standardized incidence rate of COPD was observed in High-income North America (2445.90 per 100,000 population; 95% UI, 2134.79–2810.37), followed by Andean Latin America (1547.13 per 100,000 population; 95% UI, 1297.26–1850.67) and Oceania (1291.17 per 100,000 population; 95% UI, 1172.30–1420.65), while the lowest was observed in Western Europe (768.49 per 100,000 population; 95% UI, 652.01–897.95), followed by East Asia (771.01 per 100,000 population; 95% UI, 661.24–910.73) and Western sub-Saharan Africa (778.52 per 100,000 population; 95% UI, 673.19–917.80) (Table 1; Supplementary Table S2). From 1990 to 2019, the age-standardized incidence rate of COPD increased most in High-income North America (EAPC, 1.41; 95% CI, 0.97–1.87), Southern Latin America (EAPC, 0.29; 95% CI, 0.25–0.33), and North Africa and the Middle East (EAPC, 0.09; 95% CI, 0.07–0.12), and decreased most in Eastern Europe (EAPC −1.33; 95% CI, −1.49 to −1.16), High-income Asia Pacific (EAPC, −1.26; 95% CI, −1.43 to −1.10), and Tropical Latin America (EAPC, −1.01; 95% CI, −1.14 to −0.87) (Table 1; Figure 1A; Supplementary Table S2).

**FIGURE 3:**
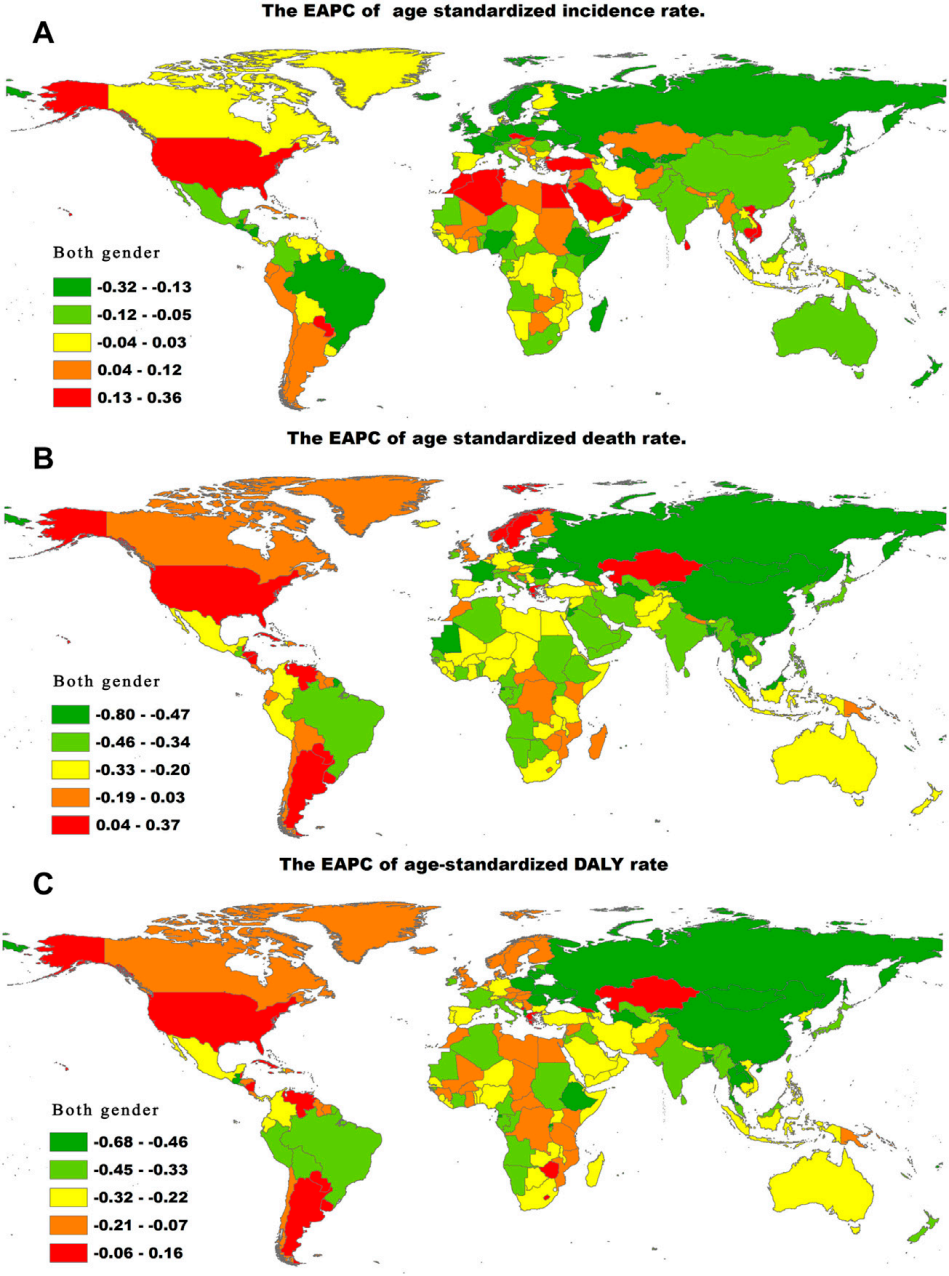
The EAPC of chronic obstructive pulmonary disease age-standardized rates from 1990 to 2019, by countries. **(A)** The EAPC of age-standardized incidence rate. **(B)** The EAPC of age standardized death rate. **(C)** The EAPC of age-standardized DALY rate. EAPC = estimated annual percentage change. DALY = disability adjusted life-year.

At the country level, the highest age-standardized incidence rate of COPD in 2019 was observed in the United States of America (2550.01 per 100,000 population; 95% UI, 2228.81–2931.83), followed by Greenland (1785.53 per 100,000 population; 95% UI, 1552.64–2075.43) and Puerto Rico (1638.51 per 100,000 population; 95% UI, 1361.88–1989.08). The lowest age-standardized incidence rate of COPD was observed in Israel (619.51 per 100,000 population; 95% UI, 518.53–730.60), followed by Turkmenistan (635.93 per 100,000 population; 95% UI, 534.97–753.96) and Finland (639.08 per 100,000 population; 95% UI, 543.10–750.98) (Supplementary Tables S1, S3). From 1990 to 2019, the age-standardized incidence rate of COPD decreased most in Ukraine (total EAPC, −1.71; male EAPC, −1.73; female EAPC, −1.70) and increased most in the United States of America (total EAPC, 1.51; male EAPC, 1.35; female EAPC, 1.64) (Figure 3A, Supplementary Tables S1, S3).

### Chronic obstructive pulmonary disease-associated deaths

From 1990 to 2019, the number of deaths due to COPD globally increased by 30%, from 2,520,219.25 in 1990 to 3,280,636.19 in 2019 (Table 2). However, the age-standardized death rate of COPD decreased globally, as indicated by an EAPC of −2.10 (95% CI, −2.19 to −2.00) (Table 2; Figure 1B; Supplementary Figure S1B). The age-standardized death rate decreased in both gender from 1990 to 2019 (male EAPC, −2.14; female EAPC, −2.11) (Table 2; Figure 1B).

**TABLE 2:** The age-standardized death rate (ASDR) of chronic obstructive pulmonary disease in 1990–2019 and its temporal trends.

Characteristics	1990		2019		1990–2019	
	ASDR (per 100,000)		ASDR (per 100,000)		Change in number No. (%)	EAPC
	No. (95% UI)	Male/female ratio	No. (95% UI)	Male/female ratio		No. (95% CI)
Global	87.89 (95.10, 73.87)	1.721028262	51.28 (55.51, 45.90)	1.679242846	0.28 (0.50, 0.15)	−2.10 (-2.19, −2.00)
Gender	—	—	—	—	—	—
Male	116.75 (126.76, 102.61)	—	66.72 (73.06, 60.55)	—	0.29 (0.50, 0.13)	−2.14 (−2.24, −2.04)
Female	67.84 (75.41, 51.59)	—	39.73 (44.75, 33.24)	—	0.28 (0.62, 0.09)	−2.11 (−2.21, −2.01)
Sociodemographic index	—	—	—	—	—	—
Low SDI	114.69 (133.08,96.34)	1.43198661	87.82 (97.67, 74.17)	1.272951786	0.63 (0.82, 0.44)	−0.98 −1.09, −0.88)
Low-middle SDI	160.43 (179.49, 135.86)	1.414921529	107.29 (120.82, 90.10)	1.388745303	0.61 (0.83, 0.36)	−1.47 (−1.56, −1.39)
Middle SDI	134.26 (147.00, 106.75)	1.313550334	59.82 (66.61, 52.30)	1.680553185	0.16 (0.51, 0.00)	−3.06 (−3.20, −2.91)
High-middle SDI	80.81 (87.92, 64.52)	1.931444022	33.22 (39.31, 29.45)	2.14453388	−0.12 (0.24, −0.24)	−3.53 (−3.71, −3.34)
High SDI	30.30 (33.21, 28.30)	2.392380044	24.64 (26.07, 21.49)	1.675408763	0.63 (0.71, 0.44)	−0.87 (−0.95, −0.80)
Region	—	—	—	—	—	—
Andean latin america	33.72 (40.56, 30.10)	1.288394758	26.84 (32.10, 20.79)	1.321598859	1.06 (1.50, 0.61)	−0.27 (−0.46, −0.08)
Australasia	36.52 (38.72, 33.93)	2.592392512	24.82 (27.50, 20.92)	1.560130411	0.60 (0.77, 0.37)	−1.57 (−1.77, −1.37)
Caribbean	28.30 (31.68, 24.92)	1.474622583	26.87 (31.67, 22.23)	1.566406547	0.88 (1.16, 0.61)	−0.18 (−0.25, −0.11)
Central asia	51.12 (54.07, 43.53)	2.051566314	39.41 (46.52, 35.47)	1.966014313	0.02 (0.30, −0.09)	−1.48 (−1.79, −1.17)
Central europe	36.96 (38.21, 33.94)	2.897494434	19.12 (22.05, 16.63)	2.492140287	−0.15(−0.02, −0.26)	−2.23 (−2.39, −2.07)
Central latin America	42.34 (44.43, 37.85)	1.378195789	33.69 (38.66, 28.27)	1.368875775	1.46 (1.78, 1.18)	−1.00 (−1.09, −0.92)
Central sub-saharan africa	86.50 (123.44,60.43)	1.103333074	65.80 (104.69, 44.51)	1.022401761	0.67(1.13,0.30)	−0.94 (−1.10,-0.79)
East asia	221.48 (246.45, 166.31)	1.369501637	67.48 (82.27, 57.80)	1.90612686	−0.15 (0.32, −0.31)	−4.45 (−4.66, −4.25)
Eastern europe	39.44 (42.36, 30.85)	3.339697612	16.23 (20.72, 14.22)	3.424205094	−0.45 (−0.20, −0.52)	−4.03 (−4.39, −3.68)
Eastern sub-saharan africa	63.20 (75.14, 53.59)	1.681368428	42.40 (48.27, 36.86)	1.722186593	0.34 (0.57,0.17)	−1.49 (−1.54, −1.44)
High-income asia pacific	24.00 (25.25, 20.85)	2.364091884	12.31 (13.74, 10.40)	2.850097152	0.70 (1.00, 0.48)	−2.59 (−2.74, −2.44)
High-income north america	31.29( 36.15, 29.23)	1.977827493	36.24 (38.58, 29.59)	1.35260301	1.11 (1.25, 0.64)	0.38 (0.20, 0.55)
North africa and middle east	53.80 (61.58, 47.11)	1.440704008	36.10 (40.31, 30.90)	1.47898833	0.64 (0.88, 0.43)	−1.63 (−1.76, −1.50)
Oceania	201.85 (235.52, 164.72)	1.279788751	166.28 (202.63, 133.34)	1.165949847	0.92 (1.38, 0.52)	−0.69 (−0.73, −0.64)
South asia	179.62 (204.39, 154.45)	1.363431814	118.75 (135.84, 97.56)	1.2520666	0.82 (1.14, 0.51)	−0.91 (−1.09, −0.73)
Southeast asia	90.37 (102.45, 70.75)	1.793211022	53.72 (59.45, 46.49)	2.220909162	0.40 (0.66, 0.20)	−1.84 (−1.92, −1.75)
Southern latin america	32.72 (37.11, 30.22)	2.291569684	32.56 (35.95, 28.24)	1.797407492	1.00 (1.22, 0.71)	−0.13 (−0.35, 0.10)
Southern sub-saharan africa	65.65 (77.28, 55.94)	1.821498954	49.21 (54.24 ,44.37)	1.966963493	0.44 (0.68, 0.22)	−1.20 (−1.67, −0.73)
Tropical latin america	54.94 (58.05, 49.56)	1.765480975	34.10 (38.25, 30.50)	1.582497701	0.91 (1.09, 0.77)	−2.01 (−2.19, −1.84)
Western europe	31.00 (33.56, 28.87)	2.92828913	22.45 (24.03, 19.62)	1.912011336	0.32 (0.41, 0.18)	−1.21 (−1.32, −1.11)
Western sub-saharan africa	54.64 (63.75, 45.14)	1.521501628	39.14 (44.62 ,33.48)	1.590955366	0.48 (0.74, 0.24)	−0.96 (−1.04, −0.88)
Abbreviations: ASDR, age-standardized death rate; EAPC, estimated annual percentage change; NA, not available; UI, uncertainty interval						

Between 1990 and 2019, the age-standardized death rate of COPD was higher in males than in females, as reflected in the male-to-female ratio of 1.72 in 1990 and 1.68 in 2019 (Table 2). The male-to-female ratio peaked in the 85–89 age group globally, as well as in high and high-middle SDI regions. The ratio peaked in the 55–59 age group in middle and low-middle SDI regions, and in the 90–94 age group in low SDI regions (Supplementary Figure S7). The rate of COPD-related deaths increased with age globally and across all SDI regions (Supplementary Figure S8).

As shown in Table 2 and Supplementary Figure S2B, the age-standardized death rates of COPD were substantially higher than those in low-middle SDI regions. In 2019, the highest age-standardized death rate for COPD was observed in the low-middle SDI regions (107.29 per 100,000 population; 95% UI, 90.10–120.82), followed by the low SDI regions (87.82 per 100,000 population; 95% UI, 74.17–97.67). The age-standardized death rate of COPD decreased most in the high-middle SDI regions where the EAPC was lowest (−3.53; 95% CI −3.71 to −3.34) (Table 2; Figure 2B).

The EAPC of the age-standardized death rate of COPD was negatively associated with the age-standardized death rate (*ρ* = −0.204, *p* = 0.003) (Supplementary Figure S4C) and the SDI (*ρ* = −0.132, *p* = 0.060) (Supplementary Figure S4D). Across regions, the proportion of COPD-related deaths among young people decreased with increasing SDI. The regions in which the SDI increased from 1990 to 2019 had a higher proportion of COPD-related deaths among older adults (Supplementary Figures S5C,D). From year to year, the annual proportion of COPD-related deaths decreased among young people but increased among older adults (Supplementary Figure S6B).

In 2019, the region with the highest age-standardized death rate of COPD was Oceania (166.28 per 100,000 population; 95% UI, 133.34–202.63), followed by South Asia (118.75 per 100,000 population; 95% UI, 97.56–135.84) and East Asia (67.48 per 100,000 population; 95% UI, 57.80–82.27). In contrast, the lowest age-standardized death rate of COPD was observed in High-income Asia Pacific (12.31 per 100,000 population; 95% UI, 10.40–13.74), followed by Eastern Europe (16.23 per 100,000 population; 95% UI, 14.22–20.72) and Central Europe (19.12 per 100,000 population; 95% UI, 16.63–22.05) (Table 2, Supplementary Table S2). From 1990 to 2019, the age-standardized death rate of COPD increased most in High-income North America (EAPC, 0.38; 95% CI, 0.20–0.55), and decreased most in East Asia (EAPC, −4.45; 95% CI -4.66 to −4.25), followed by Eastern Europe (EAPC, −4.03; 95% CI −4.39 to −3.68) and High-income Asia Pacific (EAPC −2.59; 95% CI, −2.74 to −2.44) (Table 2; Figure 1B; Supplementary Table S2).

In 2019, the three countries with the highest age-standardized death rates of COPD were Nepal (231.20 per 100,000 population; 95% UI, 175.79–270.35), Papua New Guinea (209.49 per 100,000 population; 95% UI, 162.01–259.45), and the Solomon Islands (145.87 per 100,000 population; 95% UI, 118.53–169.97). The countries with the lowest age-standardized death rates of COPD were Montenegro (9.32 per 100,000 population; 95% UI, 7.48–10.91), Latvia (9.92 per 100,000 population; 95% UI, 7.94–13.53), and Estonia (10.27 per 100,000 population; 95% UI, 8.01–13.09) (Supplementary Tables S1, S4). The age-standardized death rate of COPD decreased most in Singapore (total EAPC, −5.99; male EAPC, −6.64; female EAPC, −4.93) and increased most in Nicaragua (total EAPC 1.26; male EAPC 0.59; female EAPC 1.90) (Figure 3B; Supplementary Tables S1, S4).

### Chronic obstructive pulmonary disease-associated DALYs worldwide

From 1990 to 2019, the number of COPD-related DALYs globally increased by 26% from 59,241,939.23 to 74,432,366.82 (Table 3). In contrast, the age-standardized DALYs showed a decreasing trend, with an EAPC of −1.87 (95% CI, −1.94 to −1.81) (Table 3; Figure 1C; Supplementary Figure S1C). The age-standardized DALYs decreased in both gender from 1990 to 2019 (male EAPC, −1.99; female EAPC, −1.78; Table 3).

**TABLE 3:** The age-standardized DALY rate of chronic obstructive pulmonary disease in 1990–2019 and its temporal trends.

Characteristics	1990		2019		1990–2019	
	Age-standardized DALY rate (per 100,000)		Age-standardized DALY rate (per 100,000)		Change in number No. (%)	EAPC
	No. (95% UI)	Male/female ratio	No. (95% UI)	Male/female ratio		No. (95% CI)
Global	2107.59 (2266.19, 1836.22)	1.50707409	1293.74 (1403.57, 1182.99)	1.407727897	0.21 (0.36, 0.12)	−1.87 (−1.94, −1.81)
Gender	—	—	—	—	—	—
Male	2601.44 (2817.61, 2288.63)	—	1538.69 (1690.30, 1399.47)	—	0.21 (0.36, 0.12)	−1.99 (−2.05, −1.92)
Female	1726.15 (1896.69, 1395.38)	—	1093.03 (1208.96, 965.72)	—	0.20 (0.35, 0.08)	−1.78 (−1.85, −1.71)
Sociodemographic index	—	—	—	—	—	—
Low SDI	2749.84 (3068.74, 2370.45)	1.265562689	2048.37 (2247.14, 1802.01)	1.175737033	0.55 (0.72, 0.41)	−1.06 (−1.14, −0.98)
Low-middle SDI	3504.76 (3836.69, 2997.35)	1.371108933	2314.24 (2562.08, 2029.67)	1.332012113	0.43 (0.59, 0.27)	−1.50 (−1.56, −1.45)
Middle SDI	2712.23 (2951.54, 2230.86)	1.293526541	1316.93 (1449.40, 1199.96)	1.497322867	0.11 (0.35, −0.01)	−2.73 (−2.82, −2.63)
High-middle SDI	1767.21 (1926.22, 1507.45)	1.729409501	837.00 (948.78, 750.92)	1.590300714	−0.14 (0.10, −0.22)	−3.01 (−3.17, −2.86)
High SDI	1109.21 (1283.53,961.09)	1.518449598	924.07 (1067.03, 797.20)	1.196220418	0.36 (0.41, 0.28)	−0.66 (−0.73, −0.60)
Region	—	—	—	—	—	—
Andean latin america	1035.12 (1176.30, 903.56)	1.139196949	680.12 (811.82, 559.51)	1.161461396	0.13 (0.31, −0.02)	−1.24 (−1.43, −1.05)
Australasia	1376.88 (1606.72, 1179.57)	1.449318312	946.08 (1128.11, 793.37)	1.069345037	0.28 (0.37, 0.19)	−1.63 (−1.78, −1.49)
Caribbean	1082.25 (1261.62, 917.66)	1.159544094	960.20 (1118.46, 797.86)	1.215213762	0.34 (0.52, 0.19)	−0.39 (−0.44, −0.34)
Central asia	1290.81 (1380.62, 1147.50)	1.807079373	937.22 (1056.72, 847.05)	1.724021074	0.06 (0.27, −0.04)	−1.74 (−2.01, −1.46)
Central europe	1072.36 (1197.13, 963.33)	2.096441934	678.01 (787.39, 583.53)	1.63068517	−0.18 (−0.11, −0.24)	−1.64 (−1.78, −1.51)
Central latin America	1030.45 (1132.59, 936.74)	1.218180072	784.43 (890.61, 686.77)	1.239158911	0.62 (0.81, 0.46)	−1.15 (−1.23, −1.06)
Central sub-saharan africa	2152.87 (2792.55, 1630.09)	1.02899157	1625.00 (2246.80, 1229.32)	0.988275754	0.61 (0.96, 0.30)	−0.99 (−1.12, −0.87)
East asia	3845.41 (4279.44, 2938.76)	1.304870342	1270.89 (1470.61, 1120.48)	1.537069094	−0.21 (0.14, −0.33)	−4.16(-4.32, -4.00)
Eastern europe	1111.67 (1234.04, 943.64)	2.429138227	537.15 (633.55, 467.11)	2.202838767	−0.46 (−0.32, −0.51)	−3.36 (−3.66, −3.06)
Eastern sub-saharan africa	1822.22 (2047.46, 1588.45)	1.322199991	1231.52 (1392.41, 1082.84)	1.362047031	0.37 (0.63, 0.22)	−1.46 (−1.50, −1.42)
High-income asia pacific	837.64 (984.42, 713.06)	1.722457204	468.37 (561.90, 392.53)	1.695201421	0.12 (0.23, 0.03)	−2.34 (−2.50, −2.18)
High-income north america	1309.32 (1514.62, 1138.88)	1.29660907	1374.00 (1570.24, 1180.95)	1.072346616	0.73 (0.81, 0.55)	0.29 (0.24, 0.34)
North africa and middle east	1403.74 (1560.45, 1252.64)	1.272865941	1033.42 (1149.27, 906.68)	1.295093124	0.61 (0.76, 0.47)	−1.24 (−1.31, −1.17)
Oceania	4495.51 (5182.71, 3837.12)	1.153149037	3677.62 (4477.19, 3020.87)	1.07170978	0.84 (1.24, 0.51)	−0.69 (−0.72, −0.66)
South asia	3830.22 (4261.91, 3350.24)	1.327130642	2559.27 (2879.03, 2206.93)	1.239458147	0.64 (0.86, 0.43)	−1.12 (−1.28, −0.96)
Southeast asia	2163.45 (2373.96, 1822.55)	1.617545423	1383.12 (1511.65, 1235.32)	1.925190833	0.31 (0.46, 0.18)	−1.59 (−1.64, −1.54)
Southern latin america	1006.41 (1149.56, 889.66)	1.646307733	942.10 (1086.82, 817.88)	1.342995234	0.57 (0.68, 0.46)	−0.36 (−0.47, −0.25)
Southern sub-saharan africa	1861.61 (2094.22, 1647.57)	1.456911392	1387.64 (1520.81, 1263.76)	1.584044386	0.34 (0.48, 0.22)	−1.20 (−1.57, −0.83)
Tropical latin america	1366.72 (1516.07, 1238.72)	1.456546424	909.91 (1037.21, 808.60)	1.295126164	0.43 (0.53, 0.33)	−1.81 (−1.97, −1.65)
Western europe	1034.21 (1205.75, 899.30)	1.751412642	769.63 (900.99, 660.74)	1.260445148	0.09 (0.14, 0.03)	−1.10 (−1.18, −1.02)
Western sub-saharan africa	1524.38 (1739.37, 1310.15)	1.243206475	1128.76 (1272.71, 986.27)	1.285658232	0.68 (0.89, 0.47)	−0.95 (−1.00, −0.90)
Abbreviations: DALY, disability adjusted life-years; NA, not available; UI, uncertainty interval						

From 1990 to 2019, the age-standardized DALYs from COPD was higher in males than in females, as reflected by the male-to-female ratios of 1.51 in 1990 and 1.41 in 2019 (Table 3). The male-to-female DALYs ratio peaked in the 85–89 age group globally; it also peaked in this age group in the high and high-middle SDI regions. The ratio peaked in the 65–69 age group in the middle and the low-middle SDI regions and in the 90–94 age group in the low SDI regions (Supplementary Figure S9). The number of DALYs increased with age globally and across all SDI regions except the low-middle SDI regions (Supplementary Figure S10).

As shown in Table 3 and Supplementary Figure S2C, the age-standardized DALYs of COPD were substantially higher in low-middle SDI regions than in other regions. In 2019, the highest regional age-standardized DALYs were observed in the low-middle SDI regions (2314.24 per 100,000 population; 95% UI, 2029.67–2562.08), followed by the low SDI regions (2048.37 per 100,000 population; 95% UI, 1802.01–2247.14). The age-standardized DALYs decreased most in the high-middle SDI regions, where the values of EAPC peaked (−3.01; 95% CI, −3.17 to −2.86) (Table 3; Figure 2C).

The EAPC of the age-standardized DALYs of COPD was negatively associated with the age-standardized DALYs (*ρ* = −0.252, *p* = 0.000) (Supplementary Figure S4E) and the SDI (*ρ* = −0.059, *p* = 0.405) (Supplementary Figure S4F). In 2019, the highest rates of DALYs in high SDI regions were observed among young people (i.e., individuals 15–49 years), while regions in which the SDI had increased from 1990 to 2019 had higher rates of DALYs among older adults (Supplementary Figures S5E,F). Overall, the annual rates of DALYs decreased among young people but increased among older adults (Supplementary Figure S6C).

Across regions, the highest age-standardized DALYs of COPD in 2019 were observed in Oceania (3677.62 per 100,000 population; 95% UI, 3020.87–4477.19), followed by South Asia (2559.27 per 100,000 population; 95% UI, 2206.93–2879.03) and Central sub-Saharan Africa (1625.00 per 100,000 population; 95% UI, 1229.32–2246.80), while the lowest rates were observed in High-income Asia Pacific (468.37 per 100,000 population; 95% UI, 392.53–561.90), followed by Eastern Europe (537.15 per 100,000 population; 95% UI, 467.11–633.55) and Central Europe (678.01 per 100,000 population; 95% UI, 583.53–787.39) (Table 3; Supplementary Table S2). From 1990 to 2019, the age-standardized rates of DALYs increased most in High-income North America (EAPC, 0.29; 95% CI, 0.24–0.34) and decreased most in East Asia (EAPC, −4.16; 95% CI, −4.32 to −4.00), Eastern Europe (EAPC, −3.36; 95% CI, −3.66 to −3.06), and High-income Asia Pacific (EAPC, −2.34; 95% CI, −2.50 to −2.18) (Table 3; Figure 1C; Supplementary Table S2).

Across countries globally, the highest age-standardized DALYs of COPD in 2019 were observed in Papua New Guinea (4452.56 per 100,000 population; 95% UI, 3566.00–5534.37), followed by Nepal (4339.27 per 100,000 population; 95% UI, 3410.62–5078.79) and the Solomon Islands (3335.25 per 100,000 population; 95% UI, 2738.79–3951.13), while the lowest age-standardized DALYs were observed in Estonia (354.15 per 100,000 population; 95% UI, 293.97–425.71), followed by Montenegro (374.22 per 100,000 population; 95% UI, 302.81–459.92) and Latvia (390.72 per 100,000 population; 95% UI, 320.13–483.54) (Supplementary Tables S1, S5). Between 1990 and 2019, the age-standardized DALYs of COPD decreased most in Turkmenistan (total EAPC, −4.50; male EAPC, −4.77; female EAPC, −4.26) and increased most in Georgia (total EAPC, 0.81; male EAPC, 1.05; female EAPC, 0.31). (Figure 3C; Supplementary Tables S1, S5).

## Discussion

In this study, we systematically analyzed trends in the incidences, morbidity, mortality, and DALYs of COPD by gender, age, and SDI, using the latest GBD data from the period between 1990 and 2019. We found that the numbers of COPD-related cases, deaths, and DALYs globally increased globally from 1990 to 2019 by 86%, 30%, and 26%, respectively. In comparison, the global age-standardized COPD incidence rate (EAPC, −0.11), mortality rate (EAPC, −2.10), and DALYs decreased over the same period. The global increases in the morbidity, mortality, DALYs, and absolute numbers of COPD-related cases may have been due to the aging and increased life expectancy of the global population, as well as improvements in the diagnosis of COPD with advances in medical technology (which reduced the numbers of missed diagnoses).

We found that the age-standardized incidence and mortality rates and DALYs of COPD were higher in males than in females. Previous studies have likewise documented higher rates of COPD prevalence and mortality in men than in women (Brusselle and Lahousse, 2017). The literature suggests that there may be several reasons for this difference. First, men are more likely than women to be exposed to environmental and occupational ozone, and thus are at greater risk of developing COPD. Second, compared with women, men have lower levels of estrogen, which can stimulate the formation and release of surfactant phospholipids and reduce the incidence of respiratory distress syndrome (Seaborn et al., 2010). Although its physiological function is not fully understood, estrogen is known to boost the immune function of lung tissue. Third, the gender differences in COPD prevalence and mortality may also relate to gender differences in smoking, which is a major behavioral risk for COPD. It is estimated that men are five times more likely to smoke than women (Boukhenouna et al., 2018; Office of the Surgeon General (US) and Office on Smoking and Health (US), 2004); and studies have shown that cigarette smoke (an exogenous source of reactive oxygen species) generates an imbalance of antioxidants in the body and an increase in the restriction of airflow, which together can result in a higher risk of COPD (GBD 2015 Tobacco Collaborators, 2017). Therefore, to reduce the burden of disease caused by tobacco on men in the coming decades, governments will need to take rigorous actions to control the sales and consumption of tobacco, and actively educate the public about the harmful effects of smoking.

Pulmonary function test, as a relatively complicated inspection method, has high equipment cost and far low penetration rate (Waxman, 2001). However, developed countries have a high equipment penetration rate, and attach great importance to the screening and intervention of chronic respiratory disease, pulmonary function testing has been incorporated into routine physical examinations (Shah et al., 2016). Therefore, COPD has a high detection rate in developed countries.

We found that despite having lower age-standardized rates of COPD morbidity between 1990 and 2019, low SDI and low-middle SDI regions had higher age-standardized COPD mortality rates and DALYs, while high SDI regions showed the opposite trends. This may have been due to the fact that the actual incidence of COPD in low and middle-income countries was underestimated by local doctors who failed to diagnose and report cases of COPD due to their insufficient knowledge of the disease (Ho et al., 2019). Higher mortality rates among patients with COPD in low and middle-income countries are due in part to their limited access to healthcare and the shortage of resources in public healthcare systems. Observational studies have shown that the 1-year mortality rate from COPD after hospitalization is eight times higher in low-income areas than in high-income areas (Dagenais et al., 2020). In low and medium SDI regions, measures should be taken to improve understanding of COPD and its risk factors, enhance self-management, improve the diagnostic accuracy of medical and health systems, and develop and implement guidelines for COPD prevention and treatment (Tabyshova et al., 2021; Ghimire et al., 2022).

We found that the incidence and mortality rates and DALYs of COPD varied among regions and countries globally. Specifically, following an increase from 1990 to 2019, the age-standardized incidence rate was highest in High-income North America in 2019. This was mainly due to contributions from Puerto Rico, Greenland, and the United States. Differences between countries in the diagnostic criteria and data sources used for reporting the incidence of COPD may have driven these trends. For example, in the United States, the diagnostic criteria for COPD are continuously updated to make diagnoses of COPD more comprehensive so that the disease can be treated sooner and patients can receive better care. Nonetheless, the increasing incidence of COPD is a matter of concern. Despite decreasing from 1990 to 2019, the age-specific mortality rate and DALYs from COPD were highest in Oceania and South Asia, mainly due to contributions from Papua New Guinea, Nepal, and the Solomon Islands. Previously, a systematic analysis of data from the GBD 2015 ranked Nepal, Papua New Guinea, India, and Lesotho as the four countries with the highest age-standardized DALYs from COPD (Adhikari et al., 2018). In these low-income countries, access to primary care for COPD has typically been hindered by the limited facilities and resources available. Nonetheless, in recent years, improvements in treatments for COPD in these countries have reduced mortality rates. It is important to note that the burden of COPD is likely to increase substantially as populations age (Adhikari et al., 2018). Therefore, a more comprehensive model of care needs to be developed for low-income countries (Yadav et al., 2020).

To the best of our knowledge, this is the first study to describe trends in the incidence of COPD by gender, age, and SDI at the global, regional, and country levels using data from the GBD 2019. A key advantage of this study is the systematic use of the latest GBD data to assess the incidence and mortality rates and DALYs of COPD in various regions of the world between 1990 and 2019.

Although the study draws from the data and methods of the GBD 2019, several limitations should be noted when interpreting the findings. First, the raw data from the GBD 2019 do not include all countries and regions globally. Second, as the GBD compiles information from a variety of data sources and estimation methods, our estimates may be higher than those reported in other studies. Third, as GBD data are collected across multiple healthcare systems, the rates of underreporting and the diagnostic criteria used may vary. Fourth, as the GBD study mainly collects data at the scale of countries and regions, the data provide limited insights into potential racial and ethnic differences in the prevalence of COPD. Thus, we recommend that future studies be carried out to allow for a more comprehensive assessment of the global COPD prevalence globally. Despite the limitations of this study, the results are important for informing clinical guidelines and developing public health policies to address COPD.

In summary, despite worldwide declines in the age-standardized incidence and mortality rates and DALYs of COPD from 1990 to 2019, their absolute numbers remained high during the study period in high SDI regions. In particular, High-income North America, Southern Latin America, North Africa, and the Middle East were the regions with the greatest burdens of COPD.

## Data Availability

The original contributions presented in the study are included in the article/Supplementary Material, further inquiries can be directed to the corresponding authors.
