# Supplementary material for: Global, regional, and national burden of chronic obstructive pulmonary disease from 1990 to 2019
Source: Front Physiol. 2022 Aug 9;13:925132. doi: 10.3389/fphys.2022.925132 (PMC9396373; doi:10.3389/fphys.2022.925132)
Supplement: Supplementary file 1 [file Table1.DOC]

**
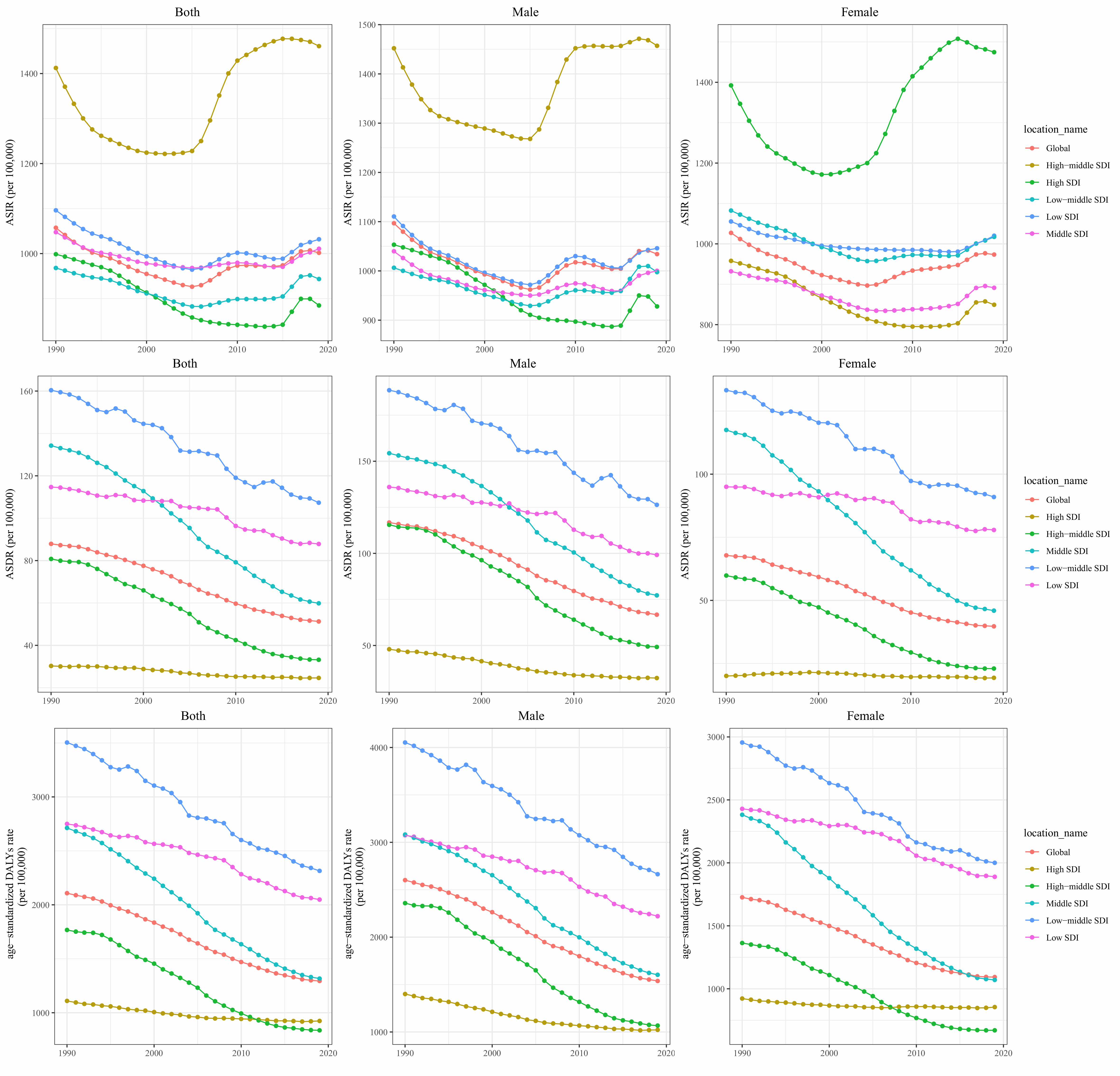
**

**Supplementary Figure 1. The change trends of age-standardized chronic obstructive pulmonary disease incidence, death, and DALY rates among different SDI quintiles and gender from 1990 to 2019.** (A) Age-standardized incidence rate. (B) Age-standardized death rate. (C) Age-standardized DALY rate. Abbreviations: DALY = disability adjusted life-year.

**
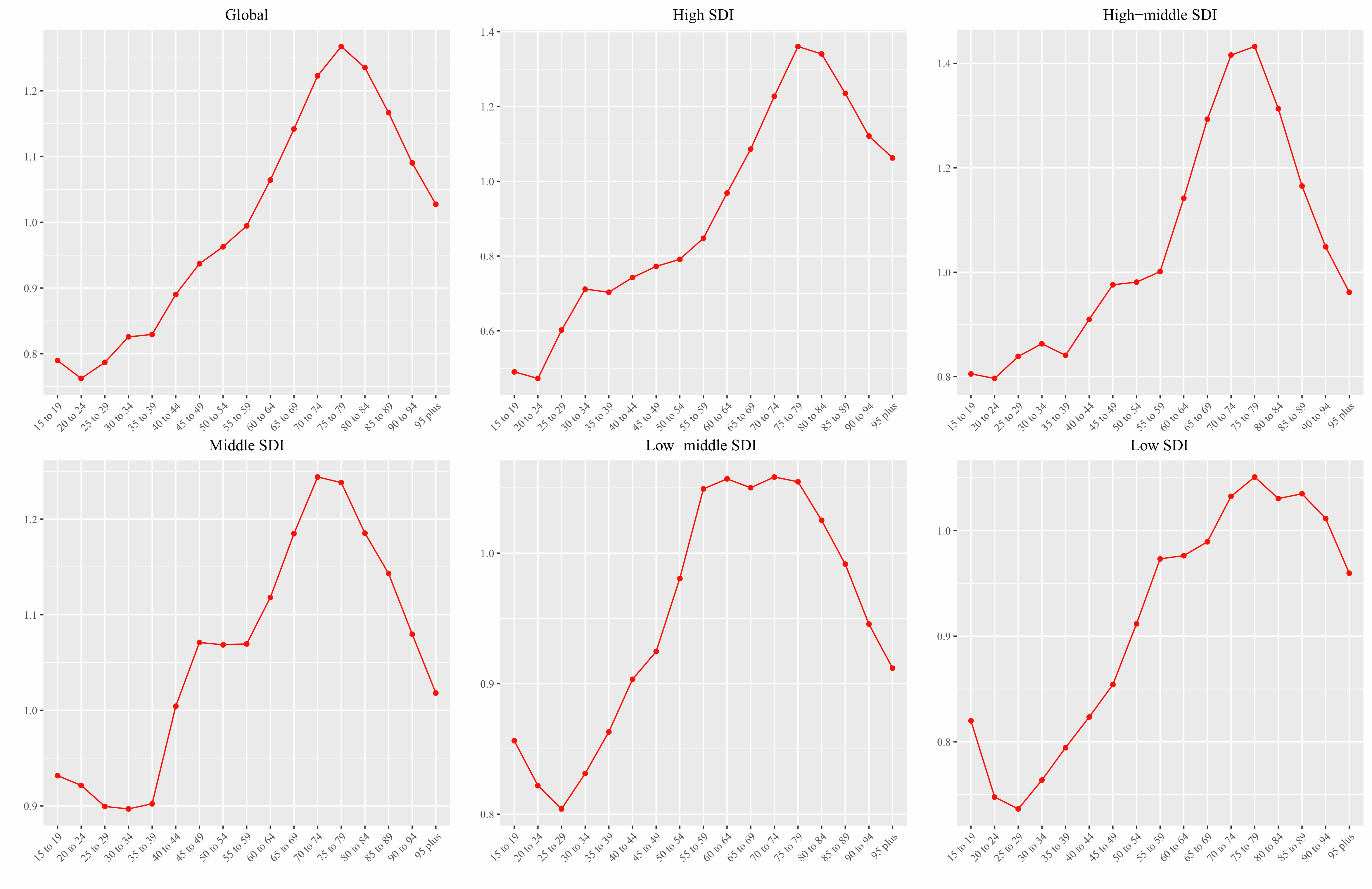
**

**Supplementary Figure 2. The ratio of male to female incidence among different age groups in 2019.** (A) Global. (B) High SDI. (C) High-middle SDI. (D) Middle SDI. (E) Middle-low SDI. (F) Low SDI. Abbreviations: SDI = socio-demographic index.

**
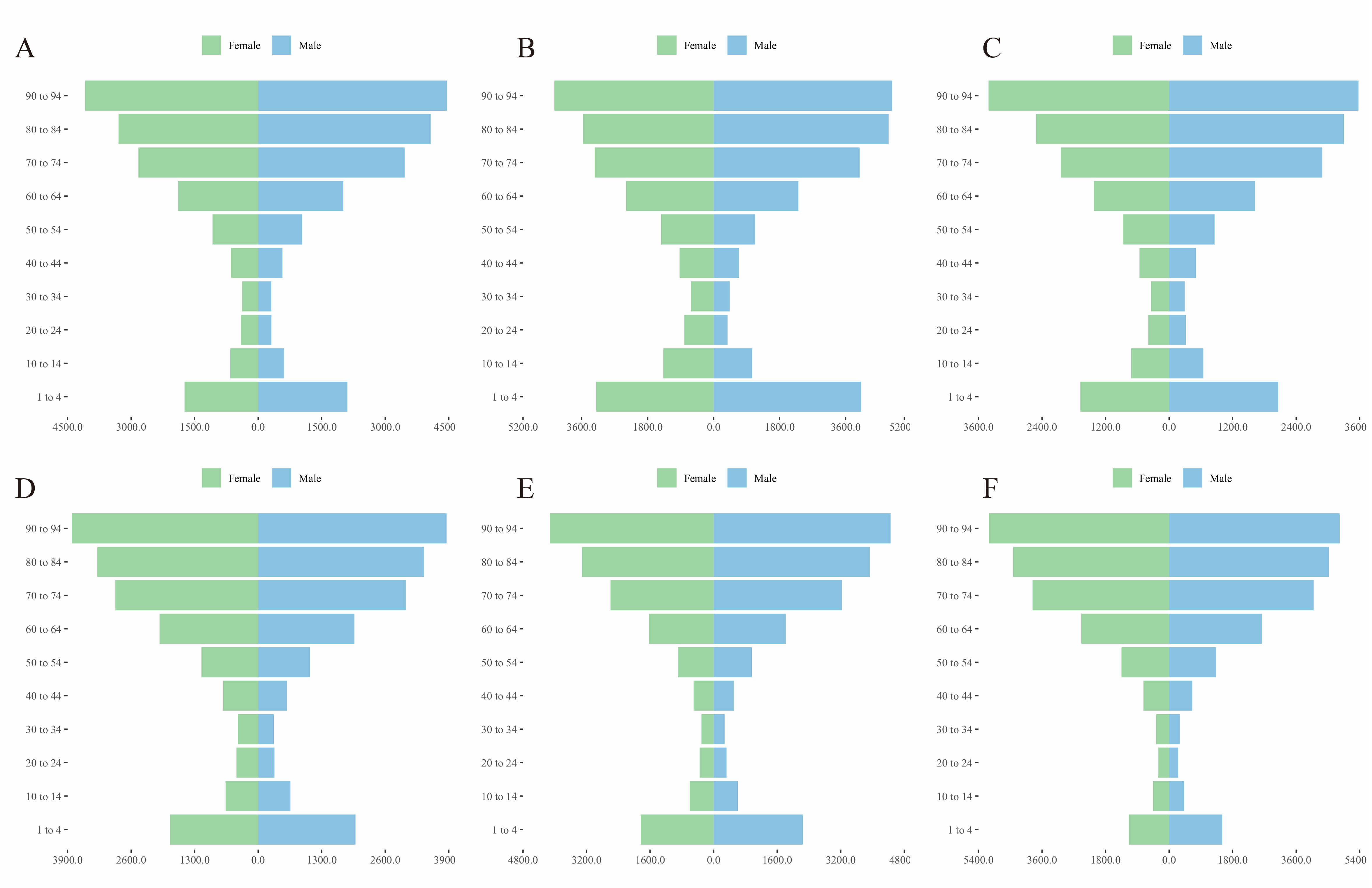
**

**Supplementary Figure 3.** Distribution of different ages in chronic obstructive pulmonary disease incidence in global (A), high SDI (B), high-middle SDI (C), middle SDI (D), middle-low SDI (E), low SDI (F). Abbreviations: SDI, socio-demographic index.

**
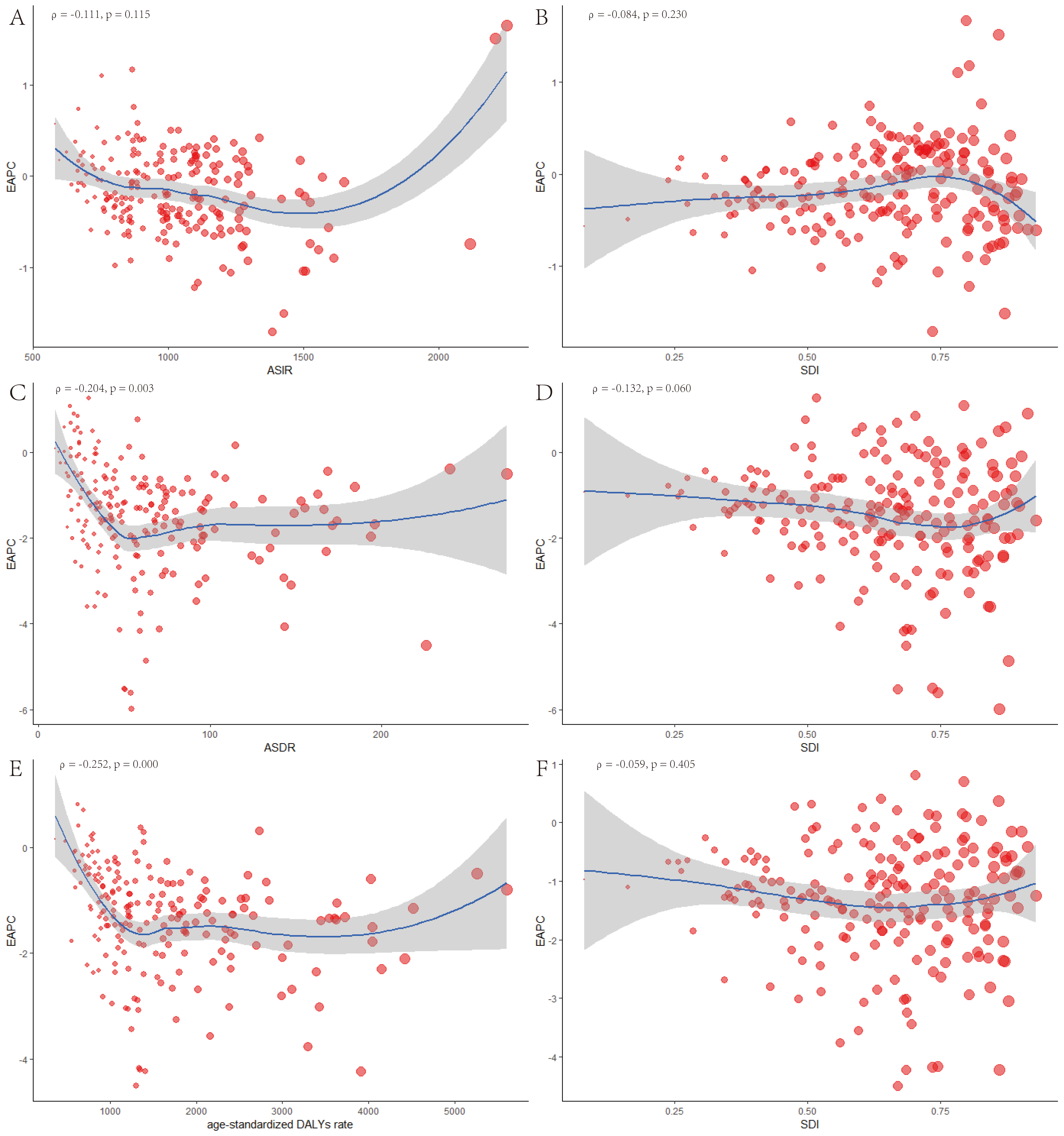
**

**Supplementary Figure 4. The correlation between EAPC and chronic obstructive pulmonary disease age-standardized rates in 1990 and SDI in 2019. The circles represent countries that were available on SDI data. The size of circle is increased with the cases of chronic respiratory diseases. The ρ indices Pearson’s correlation coefficient and p values were derived from Pearson’s correlation analysis.** (A) EAPC and age-standardized incidence rate. (B) EAPC and SDI in incidence. (C) EAPC and age-standardized death rate. (D) EAPC and SDI in death. (E) EAPC and age-standardized DALY rate. (F) EAPC and SDI in DALYs. Abbreviations: EAPC = estimated annual percentage change. SDI = socio-demographic index. DALY = disability adjusted life-year.


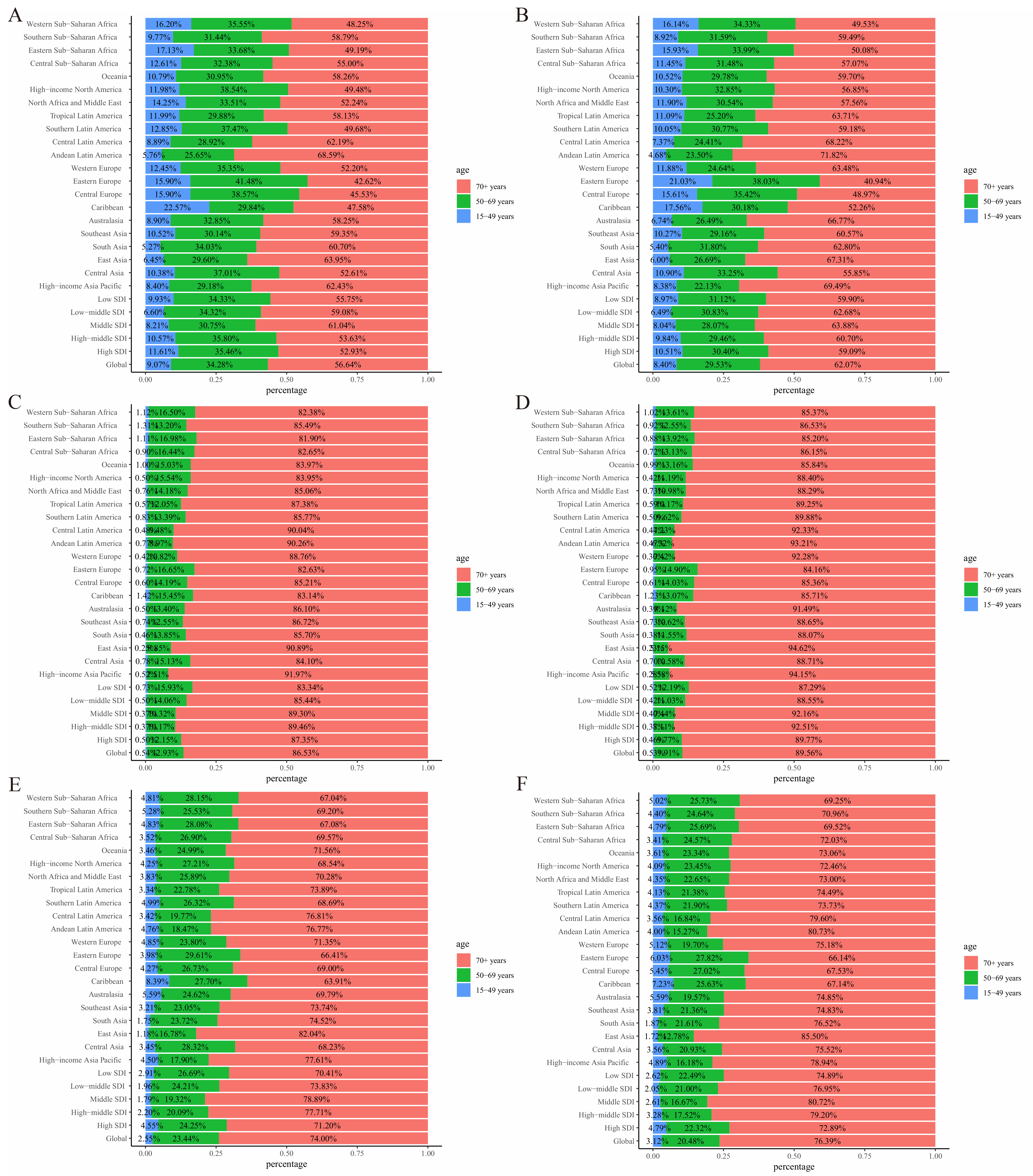


**Supplementary Figure 5. The incidence, death, and DALY rates of chronic obstructive pulmonary disease in different age groups.** (A) incidence in 1990. (B) incidence in 2019. (C) Death rate in 1990. (D) Death rate in 2019. (E) DALY rate in 1990. (F) DALY rate in 2019. Abbreviations: DALY = disability adjusted life-year.


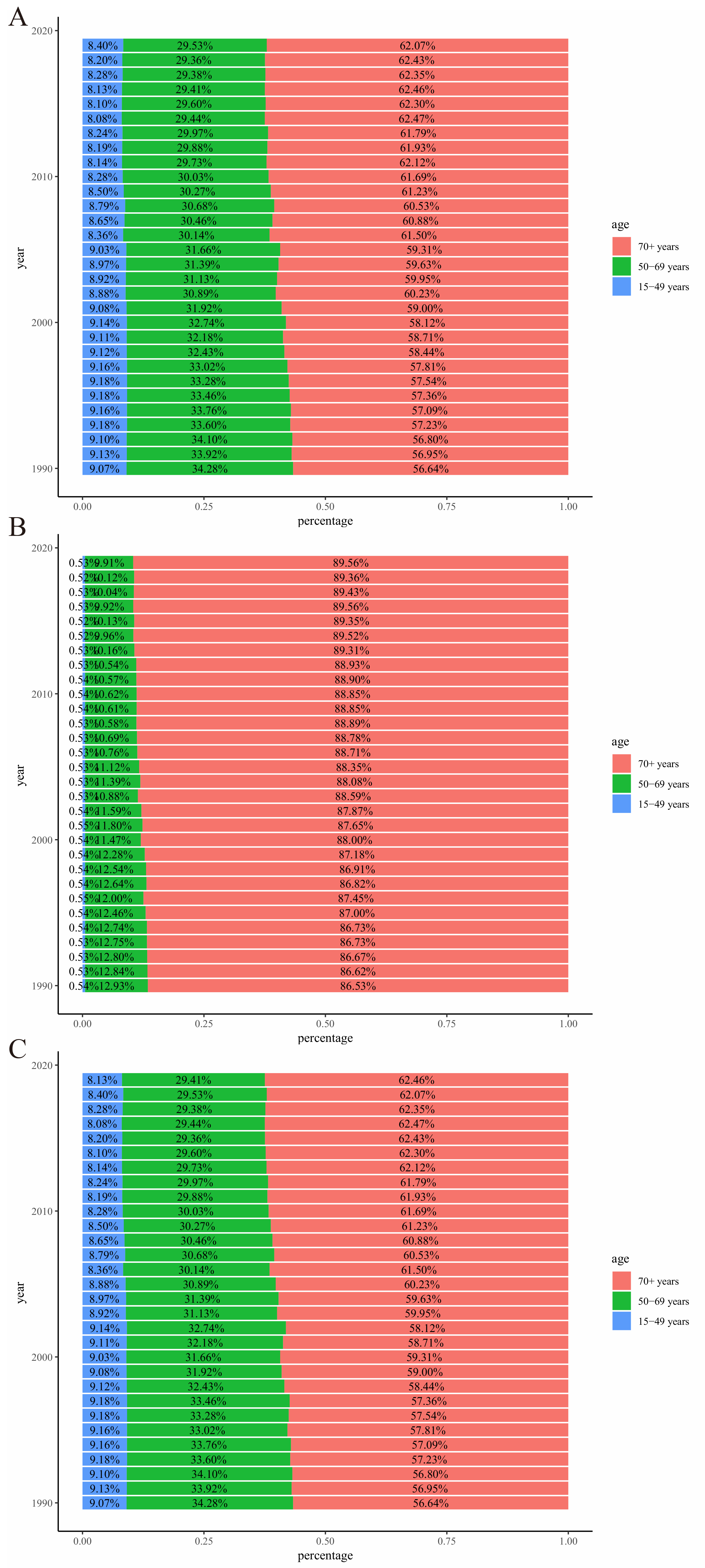


**Supplementary Figure 6.** The proportion of different ages in chronic obstructive pulmonary disease incidence (A) and death (B) and age-standardized DALY (C) by years. Abbreviations: DALY = disability adjusted life-year.


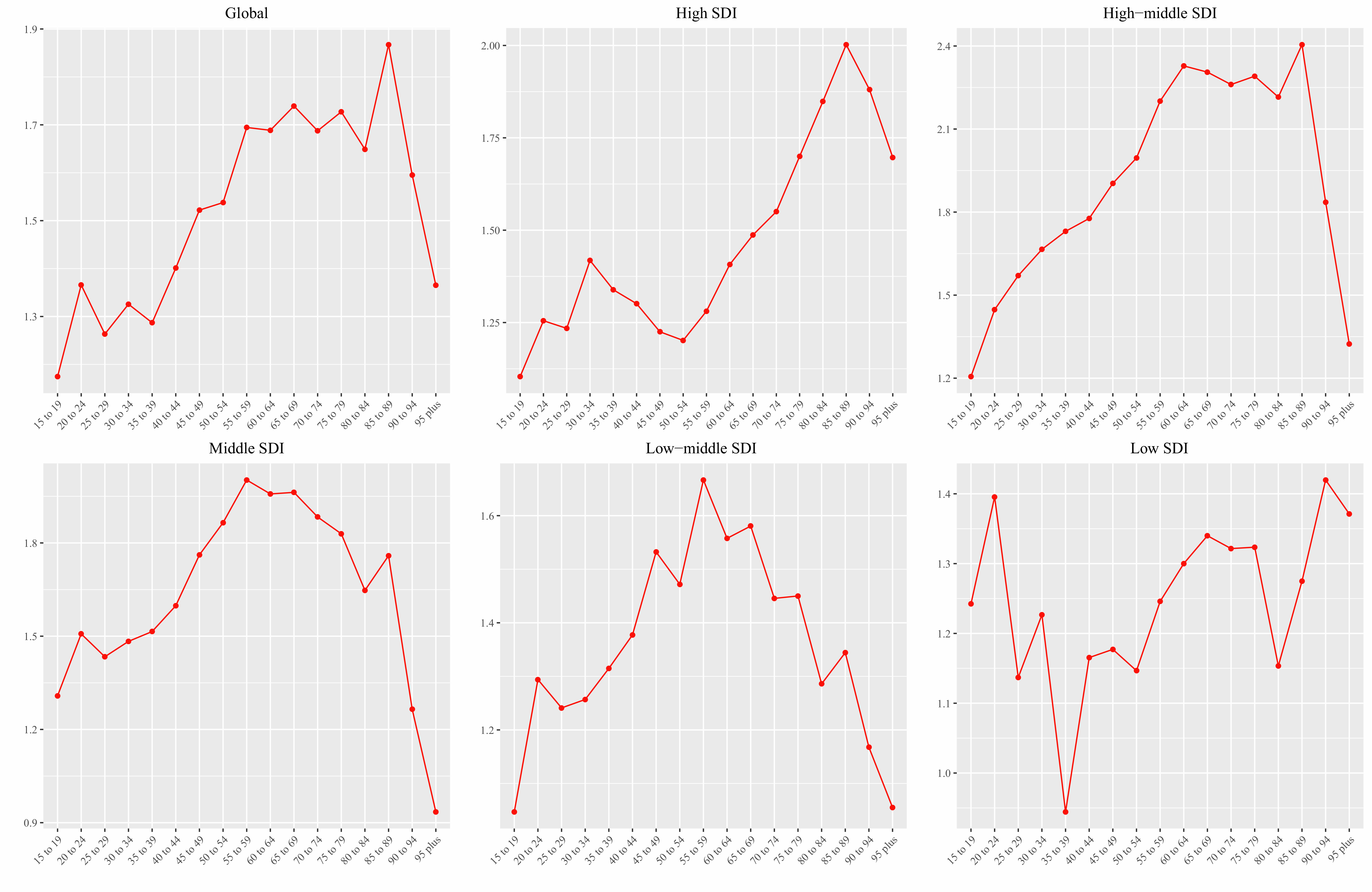


**Supplementary Figure 7. The ratio of male to female death among different age groups in 2019.** (A) Global. (B) High SDI. (C) High-middle SDI. (D) Middle SDI. (E) Middle-low SDI. (F) Low SDI. Abbreviations: SDI = socio-demographic index.


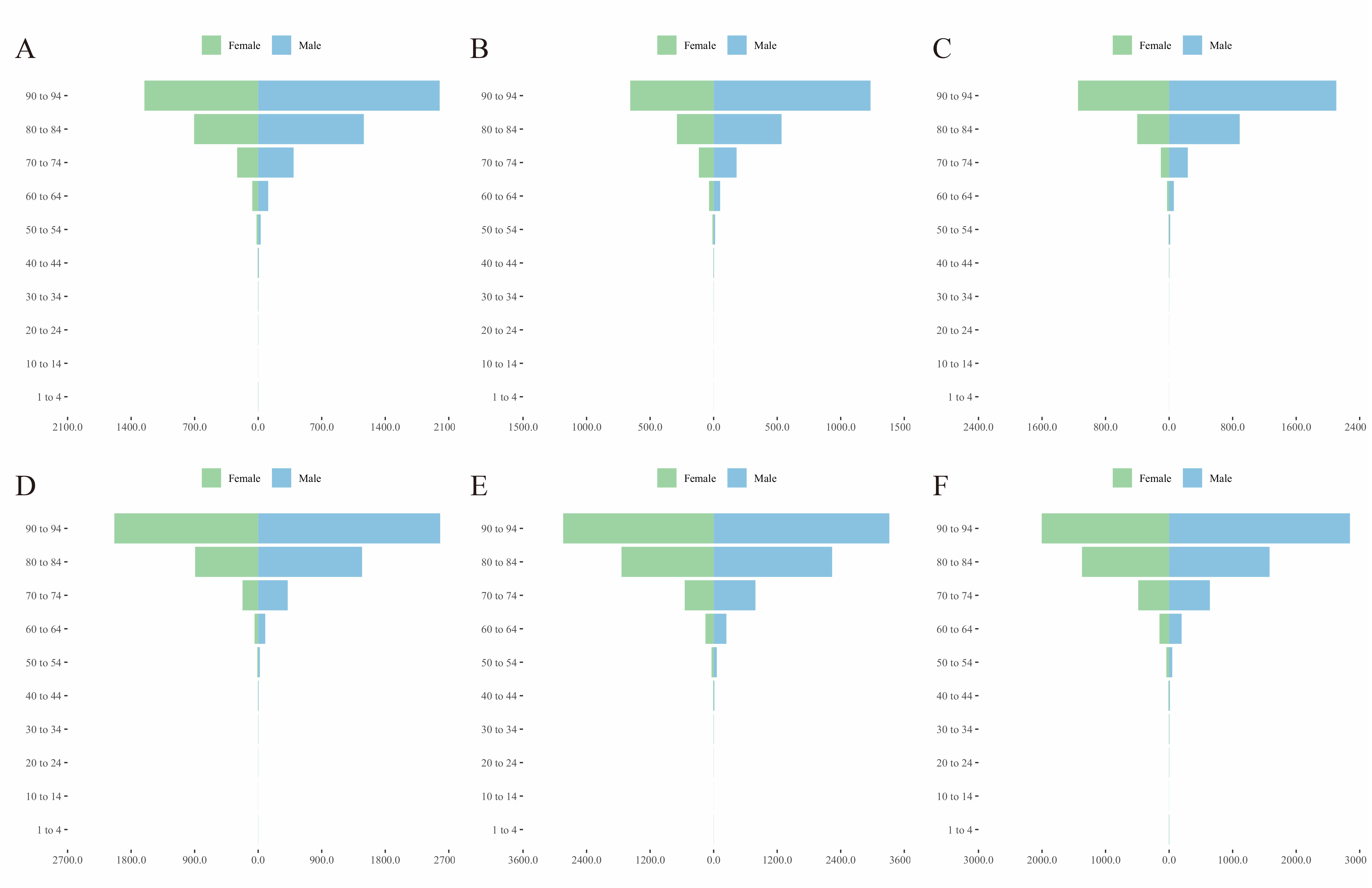


**Supplementary Figure 8.** Distribution of different ages in chronic obstructive pulmonary disease death in global (A), high SDI (B), high-middle SDI (C), middle SDI (D), middle-low SDI (E), low SDI (F). Abbreviations: SDI, socio-demographic index.


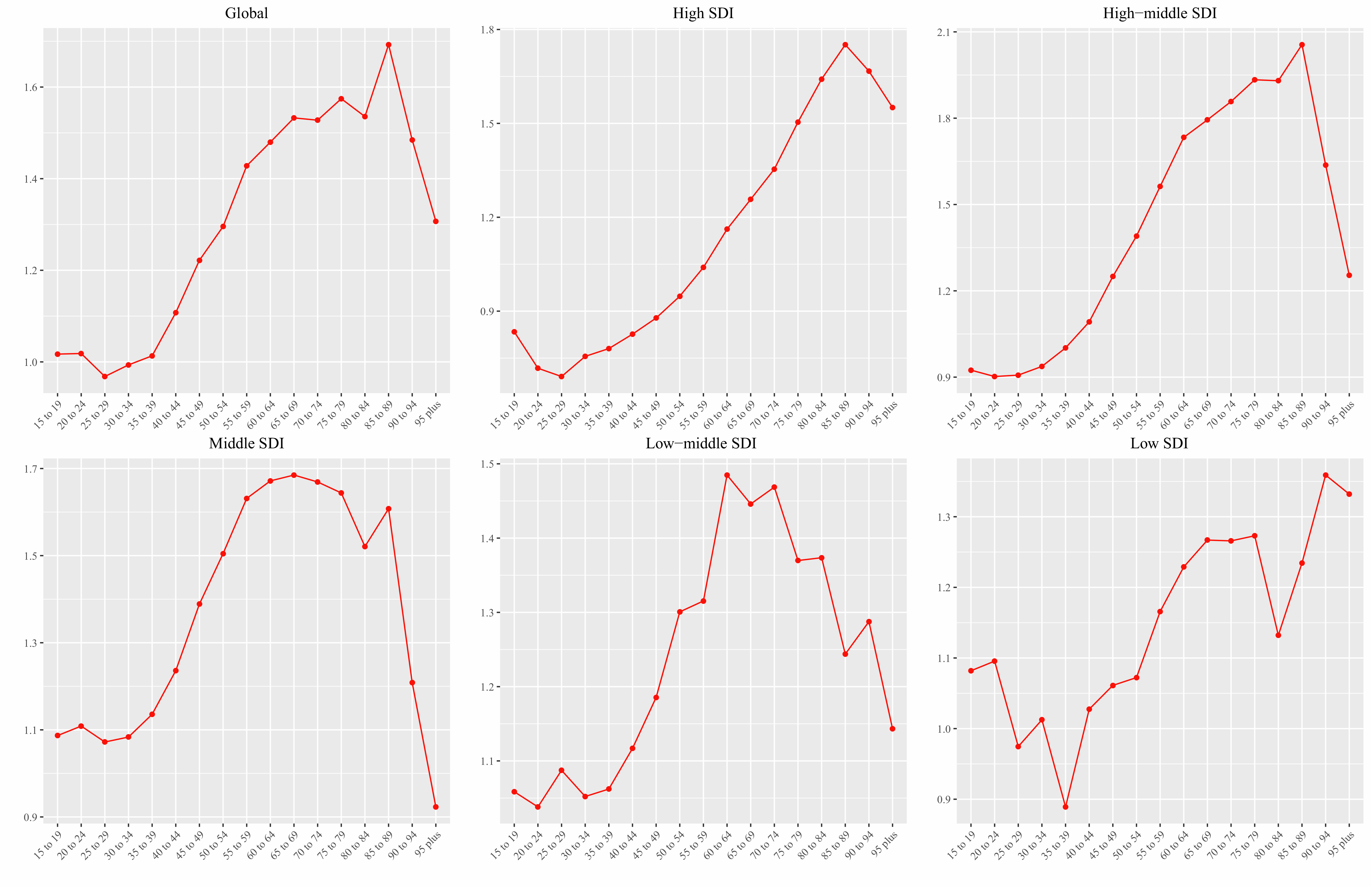


**Supplementary Figure 9. The ratio of male to female age standardized DALY rate among different age groups in 2019.** (A) Global. (B) High SDI. (C) High-middle SDI. (D) Middle SDI. (E) Middle-low SDI. (F) Low SDI. Abbreviations: SDI = socio-demographic index.

**
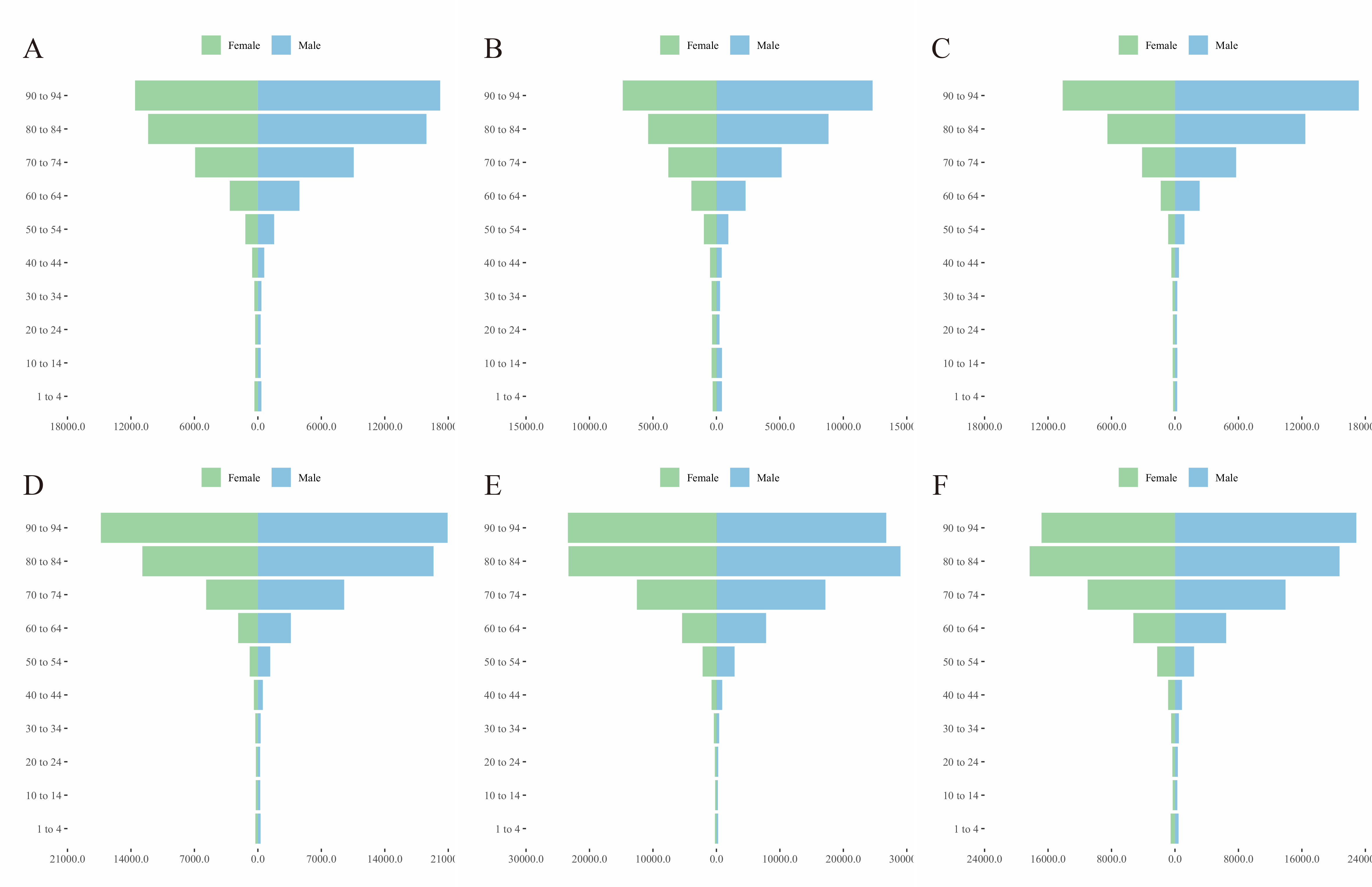
Supplementary Figure 10.** Distribution of different ages in chronic obstructive pulmonary disease DALYs in global (A), high SDI (B), high-middle SDI (C), middle SDI (D), middle-low SDI (E), low SDI (F). Abbreviations: Abbreviations: SDI, socio-demographic index.
