# Supplementary material for: Global, regional, and national burden of chronic obstructive pulmonary disease from 1990 to 2019
Source: Front Physiol. 2022 Aug 9;13:925132. doi: 10.3389/fphys.2022.925132 (PMC9396373; doi:10.3389/fphys.2022.925132)
Supplement: Supplementary file 2 [file Table2.DOCX]

**Supplementary Table 1. The top three and the bottom three countries of chronic obstructive pulmonary disease incidence, death, or DALY.**

| **Measure** | **sex** | **Top three countries** | | | **Bottom three countries** | | |
| --- | --- | --- | --- | --- | --- | --- | --- |
| **2019 ASR (per 100,000 people)** | | | | | | | |
| **ASIR** |  |  |  |  |  |  |  |
|  | both | Puerto Rico(1638.51) | Greenland(1785.53) | United States of America(2550.01) | Israel(619.51) | Turkmenistan(635.93) | Finland(639.08) |
|  | female | Puerto Rico(1690.43) | Greenland(1855.71) | United States of America(2622.86) | Seychelles(589.89) | Cambodia(602.36) | Georgia(605.56) |
|  | male | Peru(1602.85) | Greenland(1725.28) | United States of America(2483.75) | Netherlands(580.68) | Liberia(617.05) | Israel(621.09) |
| **ASDR** |  |  |  |  |  |  |  |
|  | both | Solomon Islands(145.87) | Papua New Guinea(209.49) | Nepal(231.20) | Montenegro(9.32) | Latvia(9.92) | Estonia(10.27) |
|  | female | Bhutan(131.78) | Papua New Guinea(206.44) | Nepal(224.42) | Montenegro(5.25) | Latvia(6.31) | Japan(6.40) |
|  | male | Solomon Islands(179.15) | Papua New Guinea(212.13) | Nepal(238.95) | Kuwait(14.76) | Montenegro(15.55) | Antigua and Barbuda(15.57) |
| **Age-standardized DALY rate** |  |  |  |  |  |  |  |
|  | both | Solomon Islands(3335.25) | Nepal(4339.27) | Papua New Guinea(4452.56) | Estonia(354.15) | Montenegro(374.22) | Latvia(390.72) |
|  | female | Kiribati(2762.42) | Nepal(4317.44) | Papua New Guinea(4522.15) | Estonia(279.98) | Lithuania(300.35) | Latvia(301.56) |
|  | male | Solomon Islands(3986.25) | Nepal(4357.25) | Papua New Guinea(4378.71) | Montenegro(463.31) | Estonia(482.75) | Kuwait(507.92) |
| **1990-2019 increase times** | | | | | | | |
| **Incidence (cases)** | | | | | | | |
|  | both | Afghanistan(3.27) | United Arab Emirates(4.93) | Qatar(5.95) | Ukraine(0.52) | Latvia(0.65) | Belarus(0.73) |
|  | female | Jordan(3.22) | United Arab Emirates(4.09) | Qatar(4.70) | Ukraine(0.50) | Latvia(0.71) | Belarus(0.72) |
|  | male | Afghanistan(3.49) | United Arab Emirates(5.44) | Qatar(6.91) | Ukraine(0.55) | Latvia(0.58) | Lithuania(0.74) |
| **Death (cases)** |  |  |  |  |  |  |  |
|  | both | Belize(3.34) | Venezuela (Bolivarian Republic of)(3.45) | United Arab Emirates(5.74) | Ukraine(0.36) | Belarus(0.37) | Tokelau(0.48) |
|  | female | Venezuela (Bolivarian Republic of)(3.41) | Andorra(3.57) | Nicaragua(3.59) | Ukraine(0.32) | Belarus(0.33) | Tokelau(0.47) |
|  | male | Qatar(3.70) | Belize(3.81) | United Arab Emirates(6.43) | Ukraine(0.39) | Belarus(0.40) | Tokelau(0.48) |
| **DALY (cases)** |  |  |  |  |  |  |  |
|  | both | Jordan(2.64) | Qatar(4.92) | United Arab Emirates(5.96) | Ukraine(0.40) | Belarus(0.43) | Latvia(0.49) |
|  | female | Kuwait(2.54) | Qatar(3.92) | United Arab Emirates(4.40) | Ukraine(0.36) | Belarus(0.39) | Tokelau(0.50) |
|  | male | Jordan(2.82) | Qatar(5.58) | United Arab Emirates(6.43) | Ukraine(0.43) | Latvia(0.44) | Belarus(0.45) |
| **EAPC** | | | | | | | |
| **Incidence** |  |  |  |  |  |  |  |
|  | both | Oman(1.10) | Saudi Arabia(1.17) | United States of America(1.51) | Ukraine(-1.71) | Japan(-1.51) | Russian Federation(-1.22) |
|  | female | Saudi Arabia(1.09) | Oman(1.16) | United States of America(1.64) | Ukraine(-1.70) | Uzbekistan(-1.33) | Russian Federation(-1.16) |
|  | male | Oman(1.05) | Saudi Arabia(1.26) | United States of America(1.35) | Japan(-1.93) | Ukraine(-1.73) | Russian Federation(-1.35) |
| **Death** |  |  |  |  |  |  |  |
|  | both | Norway(0.90) | Greece(1.09) | Nicaragua(1.26) | Singapore(-5.99) | Belarus(-5.61) | Turkmenistan(-5.52) |
|  | female | Sweden(1.61) | Norway(1.76) | Nicaragua(1.90) | Belarus(-6.10) | Republic of Korea(-5.65) | Ukraine(-5.64) |
|  | male | Greece(0.96) | Georgia(1.09) | Paraguay(1.27) | Singapore(-6.64) | Ukraine(-5.67) | Turkmenistan(-5.46) |
| **DALY** |  |  |  |  |  |  |  |
|  | both | Paraguay(0.41) | Greece(0.70) | Georgia(0.81) | Turkmenistan(-4.50) | Singapore(-4.23) | China(-4.22) |
|  | female | Lesotho(0.61) | United States of America(0.69) | Zimbabwe(0.96) | China(-4.60) | Turkmenistan(-4.26) | Maldives(-4.00) |
|  | male | Greece(0.79) | Paraguay(0.95) | Georgia(1.05) | Turkmenistan(-4.77) | Singapore(-4.77) | Ukraine(-4.54) |

**Supplementary Table 2. The top three and the bottom three regions of chronic obstructive pulmonary disease incidence, death, or DALY.**

| **Measure** | **sex** | **Top three regions** | | | **Bottom three regions** | | |
| --- | --- | --- | --- | --- | --- | --- | --- |
| **2019 ASIR (per 100,000 people)** | | | | | | | |
| **ASIR** | | | | | | | |
|  | both | Oceania(1291.17) | Andean Latin America(1547.13) | High-income North America(2445.90) | Western Europe(768.49) | East Asia(771.01) | Western sub-Saharan Africa(778.52) |
|  | female | Tropical Latin America(1285.90) | Andean Latin America(1566.64) | High-income North America(2505.26) | East Asia(694.18) | Southeast Asia(748.49) | Central Asia(753.90) |
|  | male | Oceania(1326.22) | Andean Latin America(1526.93) | High-income North America(2392.71) | Western Europe(747.89) | Western sub-Saharan Africa(763.05) | Central sub-Saharan Africa(825.49) |
| **ASDR** |  |  |  |  |  |  |  |
|  | both | East Asia(67.48) | South Asia(118.75) | Oceania(166.28) | High-income Asia Pacific(12.31) | Eastern Europe(16.23) | Central Europe(19.12) |
|  | female | Central sub-Saharan Africa(64.95) | South Asia(105.89) | Oceania(154.03) | High-income Asia Pacific(7.10) | Eastern Europe(8.88) | Central Europe(12.06) |
|  | male | East Asia(95.24) | South Asia(132.58) | Oceania(179.59) | High-income Asia Pacific(20.25) | Central Europe(30.05) | Eastern Europe(30.39) |
| **Age-standardized DALY rate** |  |  |  |  |  |  |  |
|  | both | Central sub-Saharan Africa(1625.00) | South Asia(2559.27) | Oceania(3677.62) | High-income Asia Pacific(468.37) | Eastern Europe(537.15) | Central Europe(678.01) |
|  | female | Central sub-Saharan Africa(1632.22) | South Asia(2290.52) | Oceania(3551.20) | High-income Asia Pacific(362.74) | Eastern Europe(370.51) | Central Europe(537.36) |
|  | male | Southeast Asia(1886.76) | South Asia(2839.00) | Oceania(3805.86) | High-income Asia Pacific(614.92) | Andean Latin America(733.94) | Eastern Europe(816.17) |
| **1990-2019 increase times** |  |  |  |  |  |  |  |
| **Incidence (cases)** | | | | | | | |
|  | both | Oceania(1.95) | Central sub-Saharan Africa(2.10) | Western sub-Saharan Africa(2.13) | Eastern Europe(0.69) | Central Europe(0.98) | High-income Asia Pacific(1.07) |
|  | female | South Asia(1.99) | Central sub-Saharan Africa(2.05) | Western sub-Saharan Africa(2.13) | Eastern Europe(0.68) | Central Europe(1.00) | High-income Asia Pacific(1.02) |
|  | male | Oceania(1.96) | Western sub-Saharan Africa(2.13) | Central sub-Saharan Africa(2.16) | Eastern Europe(0.70) | Central Europe(0.97) | Western Europe(1.03) |
| **Death (cases)** |  |  |  |  |  |  |  |
|  | both | Andean Latin America(2.06) | High-income North America(2.11) | Central Latin America(2.46) | Eastern Europe(0.55) | Central Europe(0.85) | East Asia(0.85) |
|  | female | High-income North America(2.42) | Southern Latin America(2.46) | Central Latin America(2.61) | Eastern Europe(0.51) | East Asia(0.73) | Central Europe(0.97) |
|  | male | High-income North America(1.87) | Andean Latin America(2.03) | Central Latin America(2.33) | Eastern Europe(0.58) | Central Europe(0.78) | East Asia(0.96) |
| **DALY (cases)** |  |  |  |  |  |  |  |
|  | both | Western sub-Saharan Africa(1.68) | High-income North America(1.73) | Oceania(1.84) | Eastern Europe(0.54) | East Asia(0.79) | Central Europe(0.82) |
|  | female | South Asia(1.82) | High-income North America(1.83) | Oceania(1.90) | Eastern Europe(0.51) | East Asia(0.73) | Central Europe(0.92) |
|  | male | Western sub-Saharan Africa(1.65) | North Africa and Middle East(1.66) | Oceania(1.79) | Eastern Europe(0.56) | Central Europe(0.76) | East Asia(0.84) |
| **EAPC** | | | | | | | |
| **Incidence** |  |  |  |  |  |  |  |
|  | both | North Africa and Middle East(0.09) | Southern Latin America(0.29) | High-income North America(1.41) | Eastern Europe(-1.33) | High-income Asia Pacific(-1.26) | Tropical Latin America(-1.01) |
|  | female | North Africa and Middle East(0.01) | Southern Latin America(0.44) | High-income North America(1.55) | Eastern Europe(-1.26) | Tropical Latin America(-0.98) | High-income Asia Pacific(-0.95) |
|  | male | Andean Latin America(0.16) | North Africa and Middle East(0.18) | High-income North America(1.24) | High-income Asia Pacific(-1.55) | Eastern Europe(-1.44) | Tropical Latin America(-1.02) |
| **Death** |  |  |  |  |  |  |  |
|  | both | Caribbean(-0.18) | Southern Latin America(-0.13) | High-income North America(0.38) | East Asia(-4.45) | Eastern Europe(-4.03) | High-income Asia Pacific(-2.59) |
|  | female | Caribbean(-0.27) | Southern Latin America(0.46) | High-income North America(0.98) | East Asia(-5.10) | Eastern Europe(-4.13) | High-income Asia Pacific(-3.13) |
|  | male | High-income North America(-0.38) | Andean Latin America(-0.10) | Caribbean(-0.06) | Eastern Europe(-4.24) | East Asia(-3.76) | Australasia(-2.47) |
| **DALY** |  |  |  |  |  |  |  |
|  | both | Caribbean(-0.39) | Southern Latin America(-0.36) | High-income North America(0.29) | East Asia(-4.16) | Eastern Europe(-3.36) | High-income Asia Pacific(-2.34) |
|  | female | Caribbean(-0.49) | Southern Latin America(0.11) | High-income North America(0.63) | East Asia(-4.52) | Eastern Europe(-2.99) | High-income Asia Pacific(-2.25) |
|  | male | Southern Latin America(-0.73) | Caribbean(-0.29) | High-income North America(-0.17) | East Asia(-3.79) | Eastern Europe(-3.73) | High-income Asia Pacific(-2.45) |

**Supplementary Table 3. The incidence cases and age-standardized incidence rate of chronic obstructive pulmonary disease in 1990 and 2019, and its temporal trends from 1990 to 2019.**

| **Nation** | **Sex** | **Incident Cases No. (95% UI)** | | **Change in absolute number (%)** | **ASIR per 100,000 No.(95% UI)** | | **1990-2019 EAPC No. (95%CI)** |
| --- | --- | --- | --- | --- | --- | --- | --- |
|  |  | **1990** | **2019** |  | **1990** | **2019** |  |
| Afghanistan | both | 110283.72(130109.96,93062.15) | 360801.19(440072.84,296578.62) | 2.27 | 972.16(1101.94,858.97) | 1019.17(1165.98,901.48) | 0.17(0.09,0.25) |
| Albania | both | 25248.87(29359.66,22107.16) | 29866.84(33621.58,26351.42) | 0.18 | 888.57(1005.16,792.98) | 955.32(1097.62,831.06) | 0.25(0.15,0.35) |
| Algeria | both | 195686.43(237658.53,163250.46) | 365668.17(431650.61,314237.36) | 0.87 | 813.26(937.18,710.71) | 929.14(1091.60,799.77) | 0.42(0.33,0.51) |
| American Samoa | both | 432.27(535.90,354.13) | 496.66(593.05,409.95) | 0.15 | 970.83(1126.44,837.11) | 941.01(1110.96,785.23) | 0.05(-0.13,0.24) |
| Andorra | both | 370.60(422.97,325.02) | 646.20(714.36,583.56) | 0.74 | 769.46(901.21,659.55) | 746.43(879.34,633.61) | -0.19(-0.21,-0.16) |
| Angola | both | 105899.17(128572.91,85864.40) | 254221.68(316297.99,204414.21) | 1.40 | 983.10(1132.57,857.48) | 857.02(987.08,744.86) | -0.62(-0.67,-0.57) |
| Antigua and Barbuda | both | 663.35(838.26,532.82) | 836.26(999.35,693.72) | 0.26 | 1055.61(1324.19,856.14) | 1141.32(1405.92,917.04) | 0.30(0.24,0.35) |
| Argentina | both | 368747.55(430251.61,323736.43) | 546399.16(636959.94,477296.13) | 0.48 | 1104.90(1280.90,971.95) | 1202.17(1436.70,1030.26) | 0.31(0.25,0.37) |
| Armenia | both | 27272.89(31350.70,23716.27) | 30266.44(33976.90,26375.06) | 0.11 | 881.54(996.33,775.60) | 888.37(1005.49,770.72) | 0.05(-0.04,0.13) |
| Australia | both | 206640.16(233585.80,182190.30) | 334143.38(376323.66,293273.82) | 0.62 | 1263.65(1444.45,1097.17) | 1162.79(1359.25,995.25) | -0.57(-0.72,-0.42) |
| Austria | both | 63217.29(70134.47,57294.72) | 70487.09(77277.09,64330.16) | 0.11 | 759.18(870.10,671.72) | 698.04(813.85,599.16) | -0.35(-0.41,-0.30) |
| Azerbaijan | both | 48122.06(56873.00,41583.81) | 70776.70(81072.85,62249.94) | 0.47 | 728.70(833.64,644.29) | 741.91(857.06,647.72) | 0.08(-0.07,0.24) |
| Bahrain | both | 4497.26(5445.22,3777.58) | 12828.24(14791.88,11223.31) | 1.85 | 1148.56(1308.10,1010.25) | 1142.04(1321.94,991.42) | -0.05(-0.11,0.02) |
| Bangladesh | both | 584819.36(648071.00,527612.27) | 1229420.84(1354127.21,1100359.13) | 1.10 | 864.34(935.54,789.40) | 884.17(973.64,789.84) | -0.12(-0.21,-0.03) |
| Barbados | both | 2822.00(3399.81,2385.64) | 3379.68(3938.91,2912.61) | 0.20 | 1168.62(1419.38,980.06) | 1296.79(1589.53,1056.81) | 0.41(0.36,0.47) |
| Belarus | both | 136839.16(155183.55,121558.61) | 99798.03(114917.58,87543.16) | -0.27 | 1230.61(1409.11,1078.53) | 963.59(1143.82,815.74) | -1.06(-1.21,-0.90) |
| Belgium | both | 92577.23(101879.15,84343.43) | 101925.54(110694.54,94133.87) | 0.10 | 831.96(945.07,745.78) | 732.69(841.44,636.82) | -0.39(-0.54,-0.23) |
| Belize | both | 2797.91(3492.44,2218.06) | 5271.82(6330.63,4357.04) | 0.88 | 1294.77(1556.96,1079.81) | 1364.47(1618.61,1142.18) | 0.11(0.06,0.15) |
| Benin | both | 39232.61(49548.15,31236.40) | 93622.00(117196.48,75306.51) | 1.39 | 800.96(924.36,701.73) | 753.78(870.56,654.52) | -0.32(-0.41,-0.23) |
| Bermuda | both | 649.95(770.39,550.48) | 851.83(983.55,728.35) | 0.31 | 1244.51(1510.53,1032.82) | 1371.86(1664.27,1117.25) | 0.36(0.31,0.40) |
| Bhutan | both | 3324.98(3698.05,2990.76) | 5626.04(6182.41,5078.83) | 0.69 | 888.26(956.87,821.23) | 918.64(1009.17,827.71) | 0.04(0.00,0.07) |
| Bolivia (Plurinational State of) | both | 105023.08(129695.44,82874.32) | 170324.74(208257.30,140358.50) | 0.62 | 1487.17(1748.45,1260.45) | 1488.64(1759.61,1254.44) | -0.18(-0.26,-0.10) |
| Bosnia and Herzegovina | both | 48040.19(55504.39,42317.01) | 44485.01(49791.68,39980.47) | -0.07 | 1104.45(1273.97,972.45) | 1151.17(1339.91,1007.93) | 0.22(0.17,0.27) |
| Botswana | both | 8975.02(10509.22,7707.12) | 18581.55(21913.94,15941.76) | 1.07 | 868.86(966.08,782.54) | 948.95(1093.09,830.19) | 0.23(0.13,0.33) |
| Brazil | both | 2242310.93(2860191.81,1748301.62) | 2410958.97(2946902.11,1948379.51) | 0.08 | 1501.67(1845.04,1220.04) | 1252.75(1566.43,980.03) | -1.05(-1.19,-0.91) |
| Brunei Darussalam | both | 2252.16(2721.01,1893.88) | 3667.29(4221.81,3204.95) | 0.63 | 1192.17(1367.66,1054.56) | 1081.15(1244.91,952.66) | -0.46(-0.51,-0.41) |
| Bulgaria | both | 97840.50(110087.98,86731.93) | 85538.12(95941.47,76212.70) | -0.13 | 1035.81(1187.85,911.97) | 1042.86(1203.87,906.76) | 0.01(-0.10,0.12) |
| Burkina Faso | both | 70407.36(88369.90,55330.34) | 165387.53(212638.37,130746.24) | 1.35 | 664.48(781.44,565.90) | 688.59(821.66,589.57) | 0.06(0.00,0.13) |
| Burundi | both | 70321.62(87238.04,56188.21) | 120893.38(151101.71,95687.35) | 0.72 | 1135.08(1324.94,970.36) | 958.39(1114.88,820.99) | -0.63(-0.66,-0.60) |
| Cambodia | both | 59352.71(73695.17,47742.85) | 102125.39(122298.56,87363.69) | 0.72 | 582.58(670.00,511.57) | 682.20(797.11,598.67) | 0.57(0.45,0.69) |
| Cameroon | both | 74186.07(92608.42,61016.01) | 195270.38(241988.91,158360.12) | 1.63 | 771.88(881.65,682.45) | 727.89(845.16,635.66) | -0.26(-0.38,-0.13) |
| Canada | both | 405590.42(462219.60,359688.99) | 663786.12(760807.45,580996.68) | 0.64 | 1487.65(1732.23,1294.61) | 1497.87(1754.50,1301.83) | 0.17(-0.04,0.38) |
| Cabo Verde | both | 2697.49(3330.17,2192.99) | 3680.24(4481.49,3118.88) | 0.36 | 730.07(851.28,634.18) | 689.09(832.97,587.38) | -0.23(-0.43,-0.03) |
| Central African Republic | both | 26619.59(32285.23,22053.33) | 44749.67(54179.59,37074.14) | 0.68 | 972.99(1106.59,856.11) | 889.14(1013.84,781.15) | -0.33(-0.34,-0.31) |
| Chad | both | 39860.00(49402.67,32295.73) | 109485.46(139646.07,86699.19) | 1.75 | 666.43(764.12,583.35) | 661.81(771.62,578.64) | -0.07(-0.20,0.05) |
| Chile | both | 124924.45(147972.35,107311.36) | 210644.60(238891.80,185583.94) | 0.69 | 1019.10(1192.30,891.85) | 1093.64(1283.92,947.73) | 0.19(0.13,0.25) |
| China | both | 8580786.93(10131160.05,7416100.07) | 11907502.35(13430463.79,10497328.21) | 0.39 | 863.43(998.05,756.52) | 767.50(906.86,657.12) | -0.51(-0.81,-0.22) |
| Colombia | both | 356178.09(440593.69,286340.27) | 491691.31(573454.80,424552.53) | 0.38 | 1119.32(1329.46,941.85) | 1061.52(1263.82,893.92) | -0.26(-0.40,-0.11) |
| Comoros | both | 5091.22(6337.66,4090.44) | 6457.58(7839.69,5320.32) | 0.27 | 992.16(1170.03,851.17) | 920.61(1094.87,775.36) | -0.32(-0.41,-0.24) |
| Congo | both | 24032.32(29528.41,19511.08) | 46614.91(56903.13,38289.13) | 0.94 | 992.12(1148.81,864.87) | 951.16(1108.19,819.34) | -0.21(-0.27,-0.14) |
| Costa Rica | both | 45074.85(55316.48,36421.86) | 66457.17(77162.75,57399.07) | 0.47 | 1506.94(1776.20,1279.76) | 1477.64(1754.64,1258.28) | -0.22(-0.39,-0.05) |
| Côte d'Ivoire | both | 96457.44(122446.60,77017.67) | 200427.48(250330.89,161472.10) | 1.08 | 800.86(933.18,698.81) | 777.34(917.71,665.13) | -0.18(-0.25,-0.10) |
| Croatia | both | 62295.88(70851.89,55214.90) | 64552.22(71875.39,58312.50) | 0.04 | 1176.11(1351.14,1027.34) | 1202.49(1388.12,1053.53) | 0.05(0.01,0.10) |
| Cuba | both | 129478.32(157436.66,107460.76) | 132094.60(152802.23,114626.82) | 0.02 | 1275.74(1570.36,1045.12) | 1346.74(1653.61,1092.59) | 0.17(0.14,0.20) |
| Cyprus | both | 6193.71(7107.44,5382.49) | 10688.37(11963.19,9529.02) | 0.73 | 814.93(945.57,700.89) | 801.18(939.21,686.00) | 0.04(-0.01,0.09) |
| Czechia | both | 93594.76(106319.33,82557.31) | 141984.58(159094.12,124635.22) | 0.52 | 874.13(1015.66,759.72) | 1063.34(1212.13,923.83) | 0.76(0.70,0.81) |
| Democratic Republic of the Congo | both | 341068.46(423468.32,273908.54) | 700746.51(862786.84,576422.58) | 1.05 | 866.19(994.81,748.42) | 862.66(989.99,757.33) | -0.06(-0.13,0.01) |
| Denmark | both | 45226.17(49681.34,41335.36) | 55022.52(59728.25,50562.56) | 0.22 | 828.22(954.53,729.08) | 791.58(920.09,679.20) | -0.23(-0.29,-0.17) |
| Djibouti | both | 5622.15(7086.10,4434.23) | 10974.89(13536.73,9028.58) | 0.95 | 1049.54(1234.01,893.33) | 935.16(1104.53,797.51) | -0.49(-0.58,-0.40) |
| Dominica | both | 936.18(1158.45,762.12) | 796.95(934.56,676.97) | -0.15 | 1176.85(1438.93,973.44) | 1281.74(1548.39,1054.88) | 0.30(0.20,0.39) |
| Dominican Republic | both | 72592.85(89199.40,57842.19) | 96450.08(118642.40,79380.37) | 0.33 | 873.30(1042.59,718.83) | 905.25(1113.24,744.59) | 0.29(0.13,0.44) |
| Ecuador | both | 138759.90(174462.30,107439.85) | 229588.57(276088.13,191153.84) | 0.65 | 1273.70(1536.37,1038.86) | 1406.21(1679.70,1178.97) | 0.27(0.03,0.51) |
| Egypt | both | 495048.50(600298.10,407980.41) | 934265.14(1099150.05,797348.76) | 0.89 | 909.23(1049.63,786.96) | 1042.40(1193.84,916.63) | 0.41(0.33,0.49) |
| El Salvador | both | 90871.17(114022.60,70791.36) | 79608.11(95078.98,67313.63) | -0.12 | 1526.14(1852.62,1244.55) | 1304.58(1558.70,1088.44) | -0.74(-0.88,-0.60) |
| Equatorial Guinea | both | 4142.71(5080.68,3324.13) | 11076.61(13775.80,8854.93) | 1.67 | 909.91(1035.51,788.58) | 906.33(1051.27,782.23) | 0.00(-0.06,0.05) |
| Eritrea | both | 33818.49(41587.82,27364.05) | 64405.73(79345.63,52380.73) | 0.90 | 1043.20(1211.20,896.27) | 972.52(1140.08,835.48) | -0.37(-0.44,-0.30) |
| Estonia | both | 13666.93(15379.71,12083.14) | 12223.48(13851.92,10767.64) | -0.11 | 827.45(938.32,721.41) | 797.37(928.29,683.12) | -0.30(-0.44,-0.16) |
| Ethiopia | both | 482072.71(610467.84,383712.47) | 838230.65(1084700.27,645672.41) | 0.74 | 855.98(1011.97,737.49) | 734.06(881.83,614.95) | -0.66(-0.79,-0.54) |
| Micronesia (Federated States of) | both | 965.54(1135.34,810.80) | 869.68(1016.77,741.48) | -0.10 | 1040.22(1159.60,928.03) | 957.86(1100.31,839.02) | -0.43(-0.58,-0.28) |
| Fiji | both | 6674.35(7820.21,5673.09) | 7142.17(8296.86,6219.27) | 0.07 | 1003.95(1130.75,890.42) | 826.46(949.48,724.96) | -0.90(-1.00,-0.79) |
| Finland | both | 33217.04(37384.64,29497.63) | 40188.84(44742.45,36085.82) | 0.21 | 664.46(779.38,573.02) | 639.08(750.98,543.10) | -0.16(-0.26,-0.05) |
| France | both | 533222.57(601713.70,480382.66) | 525293.97(585986.28,470367.73) | -0.01 | 862.33(1000.40,758.58) | 696.73(828.36,585.86) | -0.93(-1.00,-0.86) |
| Gabon | both | 8423.46(10231.12,6904.04) | 12983.58(15627.77,10762.39) | 0.54 | 857.34(983.04,737.08) | 801.35(937.20,687.13) | -0.35(-0.45,-0.25) |
| Georgia | both | 36800.58(41974.19,32486.11) | 30204.34(33820.25,27068.83) | -0.18 | 656.22(754.27,577.93) | 710.07(826.23,617.86) | 0.38(0.29,0.46) |
| Germany | both | 710613.56(776744.26,633336.13) | 693342.96(749549.49,641779.30) | -0.02 | 800.02(904.90,705.81) | 657.10(763.11,564.45) | -0.45(-0.61,-0.28) |
| Ghana | both | 90211.06(112957.49,72165.97) | 192385.87(233192.85,158448.09) | 1.13 | 594.54(691.40,510.72) | 646.56(754.78,558.39) | 0.18(0.09,0.26) |
| Greece | both | 76021.58(85552.11,67901.54) | 90533.55(97982.69,83269.92) | 0.19 | 676.01(792.65,590.37) | 690.51(805.08,590.04) | 0.23(0.16,0.30) |
| Greenland | both | 1002.79(1183.47,850.93) | 1049.36(1195.60,924.75) | 0.05 | 2115.46(2460.63,1808.10) | 1785.53(2075.43,1552.64) | -0.74(-0.84,-0.64) |
| Grenada | both | 1216.30(1528.29,959.91) | 1241.69(1492.78,1045.12) | 0.02 | 1252.01(1536.24,1017.44) | 1352.03(1666.10,1110.71) | 0.19(0.16,0.23) |
| Guam | both | 1403.42(1713.26,1160.06) | 1875.16(2214.10,1577.00) | 0.34 | 1188.83(1404.99,1008.32) | 1078.48(1283.67,904.38) | -0.42(-0.49,-0.34) |
| Guatemala | both | 115712.11(143596.81,90700.63) | 154762.12(190697.22,125681.34) | 0.34 | 1203.40(1418.99,1009.89) | 949.94(1136.79,799.13) | -1.01(-1.22,-0.80) |
| Guinea | both | 54898.62(69612.89,44553.09) | 106404.08(134646.75,85870.19) | 0.94 | 842.75(997.68,734.03) | 834.13(981.78,719.94) | -0.24(-0.34,-0.15) |
| Guinea-Bissau | both | 8437.18(10441.43,6845.77) | 14055.65(17196.11,11494.69) | 0.67 | 859.48(980.32,753.24) | 779.60(898.24,679.89) | -0.45(-0.57,-0.33) |
| Guyana | both | 9873.98(12218.98,7895.88) | 8954.58(10875.73,7361.78) | -0.09 | 1170.18(1407.68,969.98) | 1202.42(1460.65,990.48) | -0.06(-0.12,0.00) |
| Haiti | both | 111332.10(136343.99,88290.70) | 174852.86(212386.22,143434.08) | 0.57 | 1418.76(1681.99,1177.52) | 1357.42(1605.74,1144.00) | -0.25(-0.30,-0.20) |
| Honduras | both | 72638.68(90647.61,56484.98) | 106303.93(129419.03,87175.69) | 0.46 | 1289.15(1523.15,1081.42) | 1155.23(1355.10,979.82) | -0.60(-0.67,-0.53) |
| Hungary | both | 122301.49(136487.14,109421.73) | 136056.26(150573.32,122220.12) | 0.11 | 1081.78(1233.91,957.08) | 1128.17(1280.39,990.26) | 0.15(0.06,0.24) |
| Iceland | both | 2779.11(3245.63,2377.83) | 3481.96(3976.04,3071.70) | 0.25 | 1121.62(1327.84,939.28) | 972.94(1158.57,817.58) | -0.50(-0.65,-0.34) |
| India | both | 7375584.03(8372408.79,6475436.25) | 13942845.70(15653232.69,12291771.24) | 0.89 | 1256.92(1408.64,1109.63) | 1156.32(1298.01,1021.72) | -0.08(-0.26,0.10) |
| Indonesia | both | 1449027.92(1769660.70,1223191.05) | 1963623.84(2282282.00,1707921.48) | 0.36 | 863.11(1001.48,757.84) | 838.15(981.19,730.35) | -0.07(-0.15,0.02) |
| Iran (Islamic Republic of) | both | 545475.36(678388.54,438127.35) | 731231.27(845833.04,633930.96) | 0.34 | 948.83(1108.50,820.19) | 932.07(1091.46,799.65) | -0.22(-0.29,-0.15) |
| Iraq | both | 177568.24(218678.63,143576.08) | 349360.07(427457.82,290088.56) | 0.97 | 993.32(1146.39,859.03) | 886.28(1055.86,754.18) | -0.45(-0.51,-0.38) |
| Ireland | both | 37867.15(43210.32,33439.00) | 44874.81(49957.80,40083.32) | 0.19 | 1027.45(1172.25,902.86) | 846.40(974.22,728.03) | -0.74(-0.79,-0.68) |
| Israel | both | 35598.67(41758.03,30292.92) | 60919.12(70761.58,51666.41) | 0.71 | 715.30(833.64,617.06) | 619.51(730.60,518.53) | -0.59(-0.66,-0.52) |
| Italy | both | 513271.70(571744.49,457199.86) | 515349.15(562705.20,468849.12) | 0.00 | 773.49(890.45,677.38) | 678.87(806.99,561.85) | -0.62(-0.76,-0.47) |
| Jamaica | both | 32807.50(41083.94,26216.28) | 29547.87(35480.00,24759.78) | -0.10 | 1263.61(1551.24,1039.02) | 1190.58(1462.07,970.95) | -0.38(-0.44,-0.31) |
| Japan | both | 1872757.89(2124196.26,1646655.67) | 1862023.32(2150420.18,1608022.12) | -0.01 | 1425.77(1671.42,1234.55) | 1006.83(1207.66,852.01) | -1.51(-1.73,-1.29) |
| Jordan | both | 37222.69(46360.90,29699.70) | 118945.36(142778.97,98990.65) | 2.20 | 1097.62(1275.16,943.85) | 1178.33(1369.83,1012.09) | 0.23(0.20,0.26) |
| Kazakhstan | both | 102048.47(115942.60,90202.63) | 130951.61(147876.02,115847.31) | 0.28 | 686.48(767.19,616.88) | 720.61(811.61,639.79) | 0.26(0.06,0.46) |
| Kenya | both | 203708.76(262871.05,158271.30) | 374627.80(467533.22,300202.34) | 0.84 | 847.33(1000.11,726.23) | 784.82(932.12,669.63) | -0.37(-0.45,-0.29) |
| Kiribati | both | 861.12(996.75,740.95) | 1111.07(1267.76,975.52) | 0.29 | 1197.94(1340.78,1065.06) | 1021.75(1140.28,917.91) | -0.64(-0.68,-0.60) |
| Kuwait | both | 15639.96(19090.86,12797.57) | 39402.46(46026.38,33341.53) | 1.52 | 1020.26(1186.93,872.37) | 1089.59(1291.11,915.59) | 0.27(0.21,0.32) |
| Kyrgyzstan | both | 38332.37(45347.65,33284.17) | 49027.18(58632.64,41667.29) | 0.28 | 962.89(1083.88,866.09) | 812.30(940.46,707.20) | -0.69(-0.80,-0.58) |
| Lao People's Democratic Republic | both | 26995.83(32452.24,22538.98) | 41701.80(48144.57,36566.75) | 0.54 | 677.90(770.63,600.29) | 683.40(769.06,609.09) | -0.07(-0.15,0.00) |
| Latvia | both | 31358.78(35544.35,27665.79) | 20230.61(23117.03,17798.94) | -0.35 | 1095.41(1253.42,961.90) | 948.26(1121.37,804.07) | -0.17(-0.30,-0.04) |
| Lebanon | both | 32832.37(39786.35,27629.16) | 59704.43(69188.90,51948.47) | 0.82 | 1008.10(1178.38,871.82) | 1165.25(1352.91,1008.76) | 0.51(0.49,0.53) |
| Lesotho | both | 10288.41(11723.90,9128.74) | 12718.06(14142.79,11458.82) | 0.24 | 720.89(792.46,657.57) | 755.18(830.45,688.22) | 0.12(0.00,0.23) |
| Liberia | both | 15347.99(19434.61,12160.99) | 31337.53(39187.20,25167.35) | 1.04 | 699.59(828.50,595.27) | 665.35(790.63,567.71) | -0.28(-0.37,-0.18) |
| Libya | both | 39109.95(48049.39,31554.43) | 61082.67(69479.13,53615.26) | 0.56 | 967.75(1116.06,845.05) | 1032.08(1189.45,904.72) | 0.25(0.19,0.30) |
| Lithuania | both | 34147.77(38530.53,30422.89) | 25740.85(29225.55,22703.78) | -0.25 | 887.17(1016.31,781.21) | 829.37(972.83,699.76) | -0.12(-0.20,-0.04) |
| Luxembourg | both | 3508.51(4173.71,3133.30) | 5247.86(5805.91,4749.66) | 0.50 | 905.56(1086.91,775.09) | 802.19(934.44,684.98) | -0.58(-0.65,-0.51) |
| North Macedonia | both | 24963.36(29133.08,21213.68) | 27811.37(31150.67,24916.69) | 0.11 | 1261.04(1473.20,1075.19) | 1171.66(1351.18,1031.66) | -0.47(-0.60,-0.34) |
| Madagascar | both | 217007.20(275954.35,175255.52) | 325782.36(395072.44,266839.56) | 0.50 | 1508.38(1806.22,1278.43) | 1199.75(1387.11,1035.72) | -1.05(-1.14,-0.96) |
| Malawi | both | 93909.06(117941.77,73498.99) | 165644.26(208200.28,130715.98) | 0.76 | 883.66(1037.61,747.57) | 876.56(1037.56,740.77) | -0.07(-0.13,0.00) |
| Malaysia | both | 117793.24(138192.30,101333.42) | 205263.47(240937.01,178495.19) | 0.74 | 770.50(866.94,694.52) | 727.41(859.68,628.94) | -0.26(-0.31,-0.21) |
| Maldives | both | 2381.87(2932.60,1960.44) | 3756.82(4364.41,3249.41) | 0.58 | 1168.57(1319.48,1031.82) | 1015.89(1167.50,884.33) | -0.66(-0.72,-0.60) |
| Mali | both | 55223.97(67578.83,46052.56) | 146244.89(184521.85,118465.73) | 1.65 | 667.40(760.82,594.89) | 707.93(815.87,624.02) | 0.17(0.09,0.24) |
| Malta | both | 3204.65(3599.14,2842.23) | 3828.87(4231.59,3471.72) | 0.19 | 856.47(980.39,752.85) | 760.89(895.68,645.07) | -0.49(-0.52,-0.45) |
| Marshall Islands | both | 426.09(504.52,357.34) | 474.95(557.58,406.51) | 0.11 | 1065.06(1182.07,959.32) | 948.92(1085.24,833.89) | -0.52(-0.65,-0.39) |
| Mauritania | both | 21433.84(27429.22,16956.52) | 38186.87(48318.03,30707.22) | 0.78 | 1014.79(1206.80,873.27) | 953.86(1139.37,816.40) | -0.36(-0.46,-0.26) |
| Mauritius | both | 9185.19(10642.99,7971.07) | 12231.39(13893.29,10650.01) | 0.33 | 979.81(1116.57,861.50) | 903.34(1050.11,782.28) | -0.48(-0.54,-0.42) |
| Mexico | both | 889405.64(1108420.94,720426.61) | 1289300.79(1535699.70,1093920.68) | 0.45 | 1180.87(1377.62,1011.55) | 1108.97(1328.03,937.73) | -0.40(-0.56,-0.23) |
| Republic of Moldova | both | 44750.42(51256.00,39282.54) | 34111.59(39356.27,29926.74) | -0.24 | 1002.47(1153.17,876.74) | 875.05(1035.24,740.49) | -0.55(-0.64,-0.46) |
| Mongolia | both | 13841.35(16707.72,11615.97) | 21516.52(25277.95,18293.80) | 0.55 | 763.55(861.14,674.21) | 695.75(803.54,600.54) | -0.38(-0.46,-0.30) |
| Montenegro | both | 5370.23(6336.53,4595.48) | 6377.07(7396.02,5546.95) | 0.19 | 869.44(1021.01,742.52) | 960.63(1138.21,822.11) | 0.39(0.28,0.50) |
| Morocco | both | 177896.08(211359.24,149894.22) | 296905.67(344551.27,259000.85) | 0.67 | 738.33(842.46,644.66) | 876.42(1016.46,764.19) | 0.53(0.45,0.60) |
| Mozambique | both | 139247.88(175456.37,112606.85) | 299873.34(378803.07,236690.75) | 1.15 | 923.82(1088.93,789.79) | 937.82(1101.47,802.22) | -0.02(-0.06,0.03) |
| Myanmar | both | 260467.03(297563.99,229703.33) | 411483.87(453399.35,374413.84) | 0.58 | 787.03(861.77,719.96) | 836.77(918.98,762.80) | 0.19(0.14,0.23) |
| Namibia | both | 10013.67(11638.83,8729.84) | 16824.82(19478.47,14568.66) | 0.68 | 865.33(958.94,782.01) | 836.14(943.28,744.57) | -0.25(-0.35,-0.14) |
| Nepal | both | 101404.27(111188.86,92942.24) | 218389.33(239883.03,197171.79) | 1.15 | 848.59(918.20,780.23) | 913.60(1000.06,828.73) | 0.04(-0.05,0.12) |
| Netherlands | both | 101779.78(110254.94,94138.04) | 145207.04(154112.42,135510.82) | 0.43 | 640.02(714.50,578.78) | 641.57(712.27,576.37) | -0.09(-0.15,-0.03) |
| New Zealand | both | 52642.71(60776.92,45868.62) | 65884.45(75216.14,57487.07) | 0.25 | 1556.32(1826.65,1332.48) | 1261.44(1506.50,1070.01) | -0.81(-0.90,-0.73) |
| Nicaragua | both | 57986.44(74474.04,44807.42) | 62913.95(77960.14,51225.11) | 0.08 | 1192.63(1442.53,982.27) | 1037.52(1254.46,868.01) | -0.58(-0.72,-0.43) |
| Niger | both | 69789.50(88083.05,54726.30) | 180133.12(232495.09,140715.48) | 1.58 | 828.74(959.88,721.50) | 744.58(872.72,648.56) | -0.49(-0.59,-0.39) |
| Nigeria | both | 921696.19(1172386.55,736958.21) | 1864933.31(2408375.12,1475089.24) | 1.02 | 988.80(1170.75,852.00) | 839.62(1012.14,718.46) | -0.72(-0.83,-0.61) |
| Democratic People's Republic of Korea | both | 192095.70(227950.69,164562.64) | 266642.45(292019.75,243942.03) | 0.39 | 997.78(1145.08,881.24) | 986.32(1124.00,875.58) | -0.20(-0.26,-0.14) |
| Northern Mariana Islands | both | 425.08(507.57,358.90) | 457.39(529.89,384.08) | 0.08 | 1212.88(1418.85,1041.73) | 1105.18(1312.19,934.30) | -0.26(-0.35,-0.18) |
| Norway | both | 54805.83(61689.80,48358.03) | 61767.70(68952.89,55486.51) | 0.13 | 1231.60(1436.54,1062.66) | 1051.72(1234.99,897.54) | -0.60(-0.69,-0.50) |
| Oman | both | 14393.99(18112.32,11648.69) | 36962.00(44265.14,31076.62) | 1.57 | 753.22(891.11,639.10) | 1003.56(1190.89,852.45) | 1.10(1.00,1.20) |
| Pakistan | both | 660532.29(761376.22,580638.58) | 1133046.38(1292429.51,1005203.97) | 0.72 | 799.44(885.10,717.96) | 716.97(788.96,648.03) | -0.34(-0.39,-0.28) |
| Palestine | both | 20093.87(24908.20,16286.93) | 48647.53(58967.88,40151.27) | 1.42 | 1101.19(1267.21,947.21) | 1175.25(1364.80,1006.16) | 0.20(0.14,0.26) |
| Panama | both | 29831.87(36292.57,24388.13) | 51186.29(61018.19,43337.10) | 0.72 | 1221.53(1450.53,1032.83) | 1247.74(1489.52,1050.33) | -0.04(-0.08,0.01) |
| Papua New Guinea | both | 51979.45(59990.83,45269.88) | 114573.98(129813.61,101726.90) | 1.20 | 1524.85(1678.84,1381.45) | 1434.73(1569.22,1313.29) | -0.29(-0.34,-0.23) |
| Paraguay | both | 49461.56(64689.51,37478.62) | 79235.52(100437.04,62852.68) | 0.60 | 1035.60(1289.08,834.57) | 1201.21(1502.81,960.34) | 0.51(0.47,0.54) |
| Peru | both | 349445.26(433350.98,275578.50) | 526071.26(631662.09,435976.59) | 0.51 | 1570.23(1854.88,1295.75) | 1634.47(1971.10,1352.70) | -0.01(-0.10,0.08) |
| Philippines | both | 780983.78(1010027.07,619620.59) | 1093088.40(1356001.20,907810.40) | 0.40 | 1132.37(1385.48,948.63) | 994.02(1200.08,842.34) | -0.46(-0.52,-0.41) |
| Poland | both | 636639.70(723644.46,560491.26) | 566302.42(633112.11,507172.62) | -0.11 | 1613.87(1860.14,1418.27) | 1323.63(1563.77,1141.35) | -0.90(-0.99,-0.81) |
| Portugal | both | 102835.68(118326.58,90896.20) | 109546.94(119142.32,100526.67) | 0.07 | 970.67(1142.11,850.78) | 876.46(1023.88,743.91) | -0.38(-0.53,-0.23) |
| Puerto Rico | both | 58125.69(69731.59,47983.60) | 54199.68(61527.27,47668.43) | -0.07 | 1648.86(1979.26,1359.95) | 1638.51(1989.08,1361.88) | -0.06(-0.15,0.04) |
| Qatar | both | 3285.45(4018.29,2691.00) | 19538.77(22863.98,16628.50) | 4.95 | 920.45(1080.62,783.53) | 972.66(1147.20,836.27) | 0.13(0.09,0.18) |
| Romania | both | 321484.31(366650.36,284195.35) | 274949.29(310966.41,242567.94) | -0.14 | 1280.15(1463.72,1127.44) | 1187.96(1376.80,1030.30) | -0.32(-0.39,-0.26) |
| Russian Federation | both | 1726365.91(1983980.13,1520294.98) | 1303724.34(1496166.00,1141633.81) | -0.24 | 1096.51(1273.31,955.50) | 839.88(1000.90,712.08) | -1.22(-1.38,-1.05) |
| Rwanda | both | 134124.52(171205.29,107118.23) | 174929.85(215649.13,140489.22) | 0.30 | 1592.25(1891.82,1346.78) | 1370.48(1629.27,1156.51) | -0.57(-0.60,-0.53) |
| Saint Lucia | both | 1961.37(2416.01,1579.92) | 2067.79(2378.06,1777.37) | 0.05 | 1296.10(1549.83,1079.45) | 1358.25(1629.74,1123.34) | 0.05(-0.01,0.10) |
| Saint Vincent and the Grenadines | both | 1347.42(1719.98,1069.81) | 1164.71(1402.47,968.02) | -0.14 | 1086.44(1346.08,889.80) | 1150.60(1415.21,938.90) | 0.17(0.12,0.23) |
| Samoa | both | 1440.96(1717.06,1223.21) | 1756.31(2095.28,1475.96) | 0.22 | 980.12(1108.84,867.79) | 901.65(1047.77,783.89) | -0.40(-0.49,-0.30) |
| Sao Tome and Principe | both | 1358.02(1665.68,1115.68) | 1928.42(2276.39,1654.49) | 0.42 | 1089.94(1247.72,953.37) | 1076.99(1219.55,964.34) | -0.24(-0.30,-0.18) |
| Saudi Arabia | both | 109635.52(131619.96,90929.40) | 303531.64(349111.83,263104.45) | 1.77 | 867.11(984.82,755.24) | 1183.20(1357.63,1019.60) | 1.17(1.12,1.22) |
| Senegal | both | 51446.09(64144.91,41972.68) | 98125.06(120925.58,79978.75) | 0.91 | 688.94(792.27,606.98) | 678.76(790.38,586.94) | -0.11(-0.25,0.04) |
| Serbia | both | 86861.64(97901.78,77547.08) | 101429.98(111911.37,91809.81) | 0.17 | 883.00(1014.50,777.17) | 962.03(1095.11,847.01) | 0.44(0.39,0.49) |
| Seychelles | both | 439.47(523.69,373.56) | 657.92(758.59,578.20) | 0.50 | 623.47(727.34,543.23) | 682.45(808.37,587.12) | 0.26(0.22,0.30) |
| Sierra Leone | both | 29797.69(37311.43,24250.11) | 60061.18(74347.41,49191.06) | 1.02 | 780.68(905.88,677.32) | 745.02(866.85,646.16) | -0.23(-0.33,-0.13) |
| Singapore | both | 33759.48(37998.68,30044.48) | 56974.96(65487.05,49882.20) | 0.69 | 1277.68(1436.85,1138.76) | 980.17(1159.97,847.98) | -0.76(-0.94,-0.58) |
| Slovakia | both | 45242.36(51741.78,39568.93) | 60338.06(68265.77,52994.64) | 0.33 | 849.06(981.65,739.04) | 963.48(1103.02,836.11) | 0.47(0.41,0.53) |
| Slovenia | both | 25942.97(29327.49,23102.54) | 31783.24(35608.79,28380.62) | 0.23 | 1259.91(1442.73,1110.66) | 1230.36(1428.37,1068.23) | 0.01(-0.05,0.07) |
| Solomon Islands | both | 3009.87(3618.09,2470.06) | 5679.41(6976.12,4736.63) | 0.89 | 987.89(1110.43,875.91) | 975.99(1126.34,850.05) | -0.17(-0.33,-0.01) |
| Somalia | both | 87440.34(108688.81,68383.23) | 205915.33(260274.94,165190.24) | 1.35 | 1105.32(1292.65,937.77) | 948.62(1118.70,822.17) | -0.57(-0.64,-0.50) |
| South Africa | both | 415756.08(503557.51,333834.53) | 570530.99(674932.47,474997.12) | 0.37 | 1294.03(1507.57,1079.51) | 1134.54(1335.08,948.62) | -0.93(-1.32,-0.54) |
| Republic of Korea | both | 291449.51(341680.63,252614.33) | 441955.26(488856.39,400498.47) | 0.52 | 808.69(940.92,703.98) | 787.47(931.00,674.82) | -0.26(-0.32,-0.20) |
| South Sudan | both | 64420.16(80291.83,51826.07) | 91081.60(113673.74,72399.46) | 0.41 | 1033.26(1217.31,883.95) | 930.36(1097.87,794.59) | -0.43(-0.51,-0.36) |
| Spain | both | 306784.60(342762.33,277170.33) | 419332.76(454764.78,385995.85) | 0.37 | 720.86(828.32,635.72) | 737.32(859.19,635.20) | 0.21(0.11,0.31) |
| Sri Lanka | both | 113383.04(131604.29,98445.91) | 193526.27(214901.91,174045.99) | 0.71 | 761.29(859.68,677.35) | 863.62(984.63,767.88) | 0.37(0.35,0.39) |
| Sudan | both | 193598.41(241722.77,152855.20) | 383234.51(474127.22,311048.63) | 0.98 | 955.84(1117.94,814.35) | 1026.22(1198.52,882.35) | 0.13(0.07,0.20) |
| Suriname | both | 4543.48(5727.68,3646.70) | 6301.46(7638.94,5239.11) | 0.39 | 1112.86(1372.33,911.90) | 1163.95(1426.03,956.41) | 0.13(0.07,0.18) |
| Eswatini | both | 8048.06(9830.57,6636.02) | 10719.66(12605.55,9229.43) | 0.33 | 1164.86(1316.62,1039.86) | 1102.03(1248.44,988.06) | -0.36(-0.42,-0.30) |
| Sweden | both | 100864.37(113243.59,88796.65) | 105253.69(118312.47,94238.37) | 0.04 | 1077.14(1267.48,924.24) | 889.47(1065.64,746.01) | -0.59(-0.86,-0.32) |
| Switzerland | both | 60433.82(67180.33,54262.91) | 71197.09(78389.25,64153.32) | 0.18 | 854.28(995.73,738.63) | 741.50(884.65,627.98) | -0.61(-0.66,-0.57) |
| Syrian Arab Republic | both | 110780.44(136106.44,91244.91) | 135424.03(155968.06,117600.76) | 0.22 | 887.03(1012.62,780.29) | 992.69(1137.88,867.89) | 0.58(0.42,0.73) |
| Taiwan (Province of China) | both | 148404.11(177895.45,127403.02) | 187319.35(210504.63,165950.41) | 0.26 | 825.01(979.06,712.30) | 790.90(975.23,657.59) | -0.45(-0.59,-0.31) |
| Tajikistan | both | 42431.10(50482.26,35887.17) | 68024.77(80652.87,57309.60) | 0.60 | 993.92(1127.99,871.61) | 880.63(1006.75,768.84) | -0.47(-0.57,-0.36) |
| United Republic of Tanzania | both | 331675.19(424525.06,258254.89) | 700308.73(887526.10,548996.06) | 1.11 | 1105.09(1326.81,927.24) | 1112.10(1330.10,922.15) | 0.05(-0.02,0.12) |
| Thailand | both | 413925.08(480925.05,361118.20) | 517887.19(582025.57,462552.15) | 0.25 | 837.28(953.96,747.53) | 766.11(918.50,646.70) | -0.64(-0.77,-0.51) |
| Bahamas | both | 2873.33(3584.78,2327.35) | 3916.52(4663.92,3266.16) | 0.36 | 1141.08(1401.63,940.39) | 1218.82(1504.09,990.21) | 0.23(0.19,0.26) |
| Gambia | both | 7565.60(9520.85,6049.75) | 16134.12(19954.44,13011.77) | 1.13 | 787.76(907.80,690.99) | 755.74(881.70,659.13) | -0.22(-0.34,-0.10) |
| Timor-Leste | both | 6089.57(7541.06,4930.94) | 9891.22(11841.74,8404.30) | 0.62 | 757.68(874.42,659.67) | 784.15(896.53,694.07) | 0.09(-0.01,0.19) |
| Togo | both | 33093.05(42116.10,26103.58) | 65740.62(82081.84,53035.93) | 0.99 | 898.76(1053.72,777.45) | 849.28(1010.30,724.45) | -0.27(-0.34,-0.19) |
| Tonga | both | 988.35(1216.86,809.50) | 997.16(1197.00,830.47) | 0.01 | 1075.03(1254.29,933.31) | 990.77(1158.73,844.45) | -0.41(-0.52,-0.29) |
| Trinidad and Tobago | both | 12154.39(15034.06,9840.73) | 13508.87(15988.74,11375.00) | 0.11 | 981.37(1184.46,814.77) | 1092.85(1349.73,888.22) | 0.33(0.18,0.48) |
| Tunisia | both | 70956.48(84875.14,60021.32) | 115871.35(132943.87,101832.39) | 0.63 | 894.36(1030.24,784.78) | 1016.28(1183.42,886.11) | 0.40(0.35,0.44) |
| Turkey | both | 638624.85(752191.60,543821.61) | 1031952.92(1148185.91,927849.52) | 0.62 | 1147.99(1309.86,1014.97) | 1294.22(1466.28,1147.15) | 0.33(0.19,0.47) |
| Turkmenistan | both | 26622.82(32357.10,22514.75) | 31106.92(37219.87,25799.68) | 0.17 | 803.00(918.53,715.84) | 635.93(753.96,534.97) | -0.98(-1.24,-0.72) |
| Uganda | both | 223014.17(285792.37,173809.82) | 460042.86(589651.19,355012.70) | 1.06 | 1113.28(1315.23,943.31) | 1041.64(1246.03,877.96) | -0.34(-0.40,-0.29) |
| Ukraine | both | 790133.47(903192.68,697474.87) | 412669.13(474954.77,358810.61) | -0.48 | 1383.20(1608.77,1202.46) | 945.70(1147.41,781.30) | -1.71(-1.89,-1.52) |
| United Arab Emirates | both | 21291.40(25510.64,17851.73) | 104966.84(120958.21,91532.80) | 3.93 | 1335.32(1531.50,1159.34) | 1412.65(1622.22,1237.25) | 0.42(0.17,0.68) |
| United Kingdom | both | 723168.00(822331.82,632842.11) | 773974.89(868569.01,692482.65) | 0.07 | 1271.17(1497.58,1073.93) | 1073.13(1275.18,903.25) | -0.78(-0.87,-0.70) |
| United States of America | both | 5570049.36(6493878.02,4827879.74) | 9271837.10(10346925.12,8170487.96) | 0.66 | 2209.40(2663.02,1876.28) | 2550.01(2931.83,2228.81) | 1.51(1.02,2.01) |
| Uruguay | both | 35369.38(40905.09,31025.69) | 40694.24(46305.63,36371.95) | 0.15 | 1088.76(1271.84,945.39) | 1093.21(1291.28,949.28) | 0.03(0.01,0.05) |
| Uzbekistan | both | 192262.89(229108.97,163760.63) | 257478.33(303123.19,219592.51) | 0.34 | 1109.98(1253.96,974.53) | 874.26(1007.96,763.80) | -1.17(-1.30,-1.04) |
| Vanuatu | both | 1330.62(1575.11,1117.85) | 2526.30(3083.01,2029.69) | 0.90 | 995.48(1110.01,892.27) | 943.70(1112.85,786.90) | -0.41(-0.67,-0.14) |
| Venezuela (Bolivarian Republic of) | both | 216463.89(271739.15,171435.73) | 286231.10(337485.16,242006.09) | 0.32 | 1099.18(1329.57,913.41) | 1070.02(1279.92,888.79) | -0.22(-0.27,-0.16) |
| Viet nam | both | 421693.52(516010.64,347870.44) | 739901.88(842860.14,658481.42) | 0.75 | 665.34(777.23,582.96) | 832.43(962.09,737.21) | 0.74(0.59,0.88) |
| Virginia | both | 135912.05(158749.91,117110.90) | 252410.23(284346.29,222886.46) | 0.86 | 2251.28(2700.46,1906.44) | 2644.08(3021.60,2296.66) | 1.66(1.17,2.15) |
| Yemen | both | 137977.81(171455.81,111585.94) | 287143.76(350751.14,234288.89) | 1.08 | 1000.40(1141.10,878.25) | 995.18(1152.69,868.68) | -0.01(-0.07,0.05) |
| Zambia | both | 54912.34(68056.56,43997.58) | 127134.08(159050.94,100419.38) | 1.32 | 684.80(786.77,595.42) | 713.82(838.32,607.05) | 0.12(-0.07,0.30) |
| Zimbabwe | both | 58232.61(69403.16,49111.74) | 89091.24(103565.73,76777.33) | 0.53 | 697.04(782.51,626.74) | 713.74(798.92,639.06) | 0.02(-0.14,0.17) |
| Monaco | both | 273.56(297.10,249.89) | 352.55(382.71,324.15) | 0.29 | 754.59(890.68,646.35) | 740.62(869.80,628.08) | -0.05(-0.07,-0.04) |
| San Marino | both | 178.39(202.84,159.41) | 273.81(302.71,248.55) | 0.53 | 739.37(867.92,636.21) | 729.18(856.69,616.75) | -0.04(-0.05,-0.02) |
| Saint Kitts and Nevis | both | 522.94(649.62,419.09) | 638.20(758.35,540.10) | 0.22 | 1188.26(1449.90,968.92) | 1254.19(1553.62,1027.78) | 0.13(0.09,0.17) |
| Cook Islands | both | 205.97(248.33,173.61) | 224.88(254.65,194.37) | 0.09 | 1165.79(1357.87,1009.87) | 1151.80(1337.44,987.60) | 0.11(-0.01,0.23) |
| Nauru | both | 96.68(116.78,80.00) | 94.82(116.06,78.58) | -0.02 | 1109.18(1249.58,991.26) | 1093.90(1269.06,949.83) | -0.25(-0.47,-0.03) |
| Niue | both | 26.08(30.60,22.22) | 20.11(22.87,17.40) | -0.23 | 1095.11(1261.16,954.04) | 1125.80(1307.88,966.38) | 0.26(0.11,0.40) |
| Palau | both | 174.91(200.94,152.52) | 232.18(264.54,204.93) | 0.33 | 1304.50(1477.77,1154.85) | 1251.17(1432.71,1098.72) | -0.21(-0.27,-0.15) |
| Tokelau | both | 18.67(22.71,15.36) | 16.13(18.97,13.73) | -0.14 | 1077.07(1262.38,919.55) | 1122.83(1306.05,965.78) | 0.33(0.17,0.50) |
| Tuvalu | both | 89.89(103.10,78.51) | 121.26(142.42,103.38) | 0.35 | 1020.90(1137.57,916.81) | 1093.64(1277.68,933.90) | -0.01(-0.23,0.22) |
| Afghanistan | male | 47452.56(59210.75,38436.82) | 165716.26(207870.97,130407.53) | 2.49 | 791.52(917.21,683.37) | 853.08(992.09,733.82) | 0.28(0.17,0.39) |
| Albania | male | 13984.59(16454.28,12210.15) | 15847.16(17675.89,14251.17) | 0.13 | 1011.34(1141.06,905.96) | 1026.17(1169.18,900.99) | 0.03(-0.09,0.14) |
| Algeria | male | 96321.83(118455.91,79430.95) | 179790.10(215952.95,152110.02) | 0.87 | 784.34(908.45,684.46) | 901.34(1068.32,767.54) | 0.44(0.37,0.52) |
| American Samoa | male | 222.22(273.51,179.90) | 256.31(311.95,211.07) | 0.15 | 959.76(1122.57,826.78) | 959.57(1146.25,794.36) | -0.01(-0.17,0.15) |
| Andorra | male | 205.45(233.40,182.36) | 331.06(363.92,300.95) | 0.61 | 824.21(964.19,710.04) | 757.84(896.16,641.90) | -0.39(-0.42,-0.35) |
| Angola | male | 50240.05(63088.12,39853.08) | 126486.86(163190.33,99066.71) | 1.52 | 927.92(1085.95,794.73) | 846.48(994.44,727.50) | -0.46(-0.52,-0.40) |
| Antigua and Barbuda | male | 323.71(420.42,251.65) | 382.96(463.63,313.51) | 0.18 | 1055.65(1351.85,835.21) | 1097.72(1379.21,866.19) | 0.14(0.09,0.20) |
| Argentina | male | 183666.40(214500.00,158783.38) | 260365.01(310316.96,226248.57) | 0.42 | 1143.67(1320.32,1000.60) | 1211.81(1466.65,1040.36) | 0.15(0.09,0.21) |
| Armenia | male | 14196.71(16351.16,12263.45) | 14999.40(16862.32,13050.40) | 0.06 | 990.21(1113.73,872.61) | 964.11(1095.28,833.48) | -0.10(-0.18,-0.02) |
| Australia | male | 113617.97(128452.05,99075.18) | 175415.34(201114.62,151429.34) | 0.54 | 1440.23(1644.89,1242.25) | 1263.13(1495.64,1060.66) | -0.76(-0.91,-0.62) |
| Austria | male | 30243.39(34193.61,27128.86) | 33825.99(37370.32,30699.44) | 0.12 | 809.06(942.29,713.76) | 713.71(839.70,602.75) | -0.54(-0.59,-0.48) |
| Azerbaijan | male | 23584.98(28429.36,20156.85) | 35596.61(41026.74,31133.99) | 0.51 | 754.23(865.07,671.75) | 775.08(895.51,680.49) | 0.13(-0.04,0.30) |
| Bahrain | male | 2373.12(2901.27,1981.29) | 7080.71(8218.47,6108.11) | 1.98 | 1098.88(1269.14,960.82) | 1084.84(1263.40,928.20) | -0.06(-0.11,-0.01) |
| Bangladesh | male | 281621.51(311050.01,254579.26) | 621939.74(687191.15,551935.18) | 1.21 | 843.19(915.46,770.49) | 884.66(976.32,787.07) | 0.02(-0.08,0.11) |
| Barbados | male | 1344.26(1643.87,1121.00) | 1554.79(1834.82,1326.46) | 0.16 | 1174.55(1441.79,980.98) | 1273.87(1598.58,1017.36) | 0.31(0.26,0.37) |
| Belarus | male | 65711.99(75264.96,57864.57) | 48740.95(56083.12,42967.17) | -0.26 | 1354.76(1553.60,1189.44) | 1047.34(1258.21,898.05) | -1.10(-1.20,-1.01) |
| Belgium | male | 45895.52(50508.13,41324.34) | 49575.35(54027.88,45390.18) | 0.08 | 877.40(996.98,780.38) | 747.95(869.24,648.17) | -0.42(-0.57,-0.28) |
| Belize | male | 1468.83(1852.56,1153.75) | 2769.71(3348.58,2276.30) | 0.89 | 1352.38(1629.51,1126.65) | 1458.82(1747.03,1210.65) | 0.19(0.14,0.24) |
| Benin | male | 20040.72(25667.35,15748.32) | 46123.07(59316.09,35870.02) | 1.30 | 811.06(941.15,705.62) | 730.26(860.50,622.52) | -0.50(-0.58,-0.42) |
| Bermuda | male | 326.81(389.75,273.08) | 438.16(508.52,374.67) | 0.34 | 1316.05(1601.45,1073.88) | 1445.73(1752.95,1173.44) | 0.34(0.31,0.36) |
| Bhutan | male | 1464.60(1653.08,1310.28) | 2710.29(2986.34,2437.46) | 0.85 | 782.33(847.05,722.14) | 867.25(956.33,780.63) | 0.40(0.36,0.44) |
| Bolivia (Plurinational State of) | male | 52910.24(65994.15,40658.13) | 83219.90(104520.43,67759.15) | 0.57 | 1460.04(1729.18,1208.02) | 1448.23(1750.68,1210.06) | -0.19(-0.27,-0.11) |
| Bosnia and Herzegovina | male | 24948.84(29300.05,21920.43) | 22575.92(25272.90,20298.41) | -0.10 | 1202.67(1397.06,1056.34) | 1250.54(1466.80,1085.18) | 0.22(0.17,0.28) |
| Botswana | male | 4688.92(5540.73,3973.85) | 9283.25(11103.26,7837.58) | 0.98 | 1027.83(1136.88,923.69) | 1059.13(1217.41,921.50) | -0.06(-0.14,0.01) |
| Brazil | male | 1097185.70(1425425.26,843761.07) | 1145186.20(1424294.98,921883.69) | 0.04 | 1495.74(1856.49,1210.80) | 1228.76(1570.08,957.12) | -1.06(-1.17,-0.94) |
| Brunei Darussalam | male | 1209.12(1455.33,1018.17) | 1958.76(2241.71,1711.70) | 0.62 | 1288.97(1458.73,1149.80) | 1166.64(1341.53,1031.80) | -0.48(-0.54,-0.42) |
| Bulgaria | male | 49414.21(55840.18,44077.84) | 40770.85(45311.29,36484.55) | -0.17 | 1082.43(1250.20,951.91) | 1058.64(1226.98,917.19) | -0.09(-0.20,0.02) |
| Burkina Faso | male | 31962.44(41045.48,24613.78) | 76897.30(99212.49,59056.41) | 1.41 | 604.10(718.50,515.41) | 641.07(764.50,538.43) | 0.18(0.11,0.26) |
| Burundi | male | 35590.25(45549.44,27997.61) | 61120.93(77525.84,47608.97) | 0.72 | 1206.20(1414.49,1036.99) | 992.15(1174.08,846.35) | -0.73(-0.77,-0.69) |
| Cambodia | male | 31098.77(38928.91,24874.45) | 54626.26(66110.79,46434.43) | 0.76 | 652.10(748.52,573.16) | 784.85(909.82,693.12) | 0.71(0.59,0.83) |
| Cameroon | male | 36850.58(46857.51,29570.33) | 98635.26(124645.11,78185.92) | 1.68 | 754.15(873.90,659.98) | 724.06(846.57,624.82) | -0.18(-0.31,-0.06) |
| Canada | male | 209158.90(239340.69,184118.51) | 331363.82(383048.58,286419.51) | 0.58 | 1622.79(1891.19,1410.46) | 1571.28(1871.53,1348.64) | -0.03(-0.26,0.21) |
| Cabo Verde | male | 1347.09(1723.95,1062.48) | 1821.79(2239.68,1506.69) | 0.35 | 764.49(900.72,657.94) | 694.95(848.72,587.03) | -0.37(-0.57,-0.17) |
| Central African Republic | male | 13054.47(16225.33,10534.83) | 22140.70(27424.86,17824.56) | 0.70 | 966.36(1113.22,839.05) | 887.97(1028.98,771.85) | -0.33(-0.35,-0.31) |
| Chad | male | 21577.42(27535.75,17070.79) | 60272.67(77275.73,46838.23) | 1.79 | 715.91(840.96,618.07) | 692.25(824.45,595.90) | -0.17(-0.28,-0.06) |
| Chile | male | 63445.34(74975.46,54491.68) | 102452.98(119519.37,89029.28) | 0.61 | 1078.79(1243.65,940.22) | 1127.86(1349.40,966.53) | 0.06(-0.03,0.15) |
| China | male | 4627421.98(5539172.58,3951672.74) | 6449484.08(7347521.81,5660641.68) | 0.39 | 927.98(1077.85,809.07) | 849.32(1004.75,723.78) | -0.34(-0.62,-0.06) |
| Colombia | male | 181533.83(229034.62,143816.71) | 237640.78(283072.28,201810.65) | 0.31 | 1133.93(1366.01,947.25) | 1075.18(1299.58,900.14) | -0.29(-0.43,-0.14) |
| Comoros | male | 2595.09(3365.30,2043.49) | 3257.38(4045.14,2625.83) | 0.26 | 1027.09(1226.49,872.32) | 951.32(1144.22,794.65) | -0.31(-0.43,-0.19) |
| Congo | male | 12838.45(16256.80,10121.32) | 24462.74(30864.89,19406.99) | 0.91 | 1045.73(1231.06,896.74) | 964.00(1157.85,812.66) | -0.38(-0.46,-0.30) |
| Costa Rica | male | 21538.45(26913.86,17267.78) | 30613.51(36159.29,26065.18) | 0.42 | 1401.89(1677.87,1178.57) | 1437.37(1730.57,1197.57) | -0.07(-0.28,0.14) |
| Côte d'Ivoire | male | 44995.70(57425.85,35599.11) | 92685.35(117789.01,72378.94) | 1.06 | 741.66(866.76,646.17) | 695.63(831.24,589.64) | -0.32(-0.41,-0.23) |
| Croatia | male | 30393.37(34708.11,26914.26) | 31194.14(34545.28,28272.52) | 0.03 | 1256.78(1458.81,1105.27) | 1263.90(1460.19,1104.04) | -0.08(-0.13,-0.02) |
| Cuba | male | 60900.95(75680.32,48333.01) | 63815.06(75386.56,54640.52) | 0.05 | 1223.43(1542.35,959.25) | 1332.03(1665.51,1068.41) | 0.27(0.22,0.32) |
| Cyprus | male | 2925.57(3382.23,2551.78) | 5111.95(5693.00,4602.80) | 0.75 | 776.34(899.86,672.27) | 766.47(903.62,655.39) | 0.01(-0.05,0.07) |
| Czechia | male | 45568.44(51920.71,40437.70) | 71627.60(80417.85,62923.67) | 0.57 | 913.82(1063.09,798.93) | 1114.97(1279.08,961.52) | 0.76(0.68,0.83) |
| Democratic Republic of the Congo | male | 157545.30(201727.09,122394.49) | 331321.36(418954.23,266300.65) | 1.10 | 800.66(938.52,683.57) | 805.20(946.35,700.11) | -0.03(-0.11,0.05) |
| Denmark | male | 23779.76(26194.94,21763.04) | 26088.32(28673.85,23635.23) | 0.10 | 914.88(1056.95,798.76) | 793.90(932.04,675.86) | -0.46(-0.52,-0.40) |
| Djibouti | male | 3169.69(4060.37,2484.82) | 6013.42(7548.18,4892.62) | 0.90 | 1128.23(1348.43,958.58) | 977.44(1170.74,831.53) | -0.60(-0.70,-0.50) |
| Dominica | male | 480.78(611.24,385.08) | 421.25(499.82,352.82) | -0.12 | 1222.93(1526.18,1007.63) | 1354.64(1655.20,1103.59) | 0.41(0.31,0.51) |
| Dominican Republic | male | 33522.37(41646.07,26238.91) | 45237.39(57284.26,36284.82) | 0.35 | 800.53(970.43,651.65) | 848.70(1069.70,683.45) | 0.31(0.18,0.44) |
| Ecuador | male | 68457.62(87116.22,53057.76) | 113843.66(138865.77,93968.71) | 0.66 | 1248.87(1513.63,1014.98) | 1415.05(1702.18,1174.94) | 0.37(0.14,0.60) |
| Egypt | male | 246139.03(302009.59,201255.85) | 481923.03(572755.12,409985.67) | 0.96 | 878.28(1019.36,757.12) | 1023.40(1181.50,896.07) | 0.49(0.42,0.56) |
| El Salvador | male | 46290.75(59804.55,35479.66) | 36804.76(45100.62,29908.86) | -0.20 | 1514.03(1873.41,1212.01) | 1277.80(1542.49,1048.46) | -0.79(-0.95,-0.64) |
| Equatorial Guinea | male | 2022.42(2574.57,1564.30) | 5910.46(7614.07,4586.35) | 1.92 | 900.75(1058.37,767.50) | 862.70(1019.79,729.73) | -0.19(-0.27,-0.10) |
| Eritrea | male | 17608.44(22197.19,13851.94) | 33366.15(42096.82,26416.94) | 0.89 | 1120.96(1314.56,965.61) | 1039.12(1223.55,884.28) | -0.37(-0.44,-0.30) |
| Estonia | male | 6844.15(7711.94,6045.24) | 5840.00(6603.68,5173.42) | -0.15 | 921.05(1041.84,812.27) | 846.98(985.93,729.38) | -0.46(-0.59,-0.33) |
| Ethiopia | male | 241620.71(311428.66,187168.01) | 451263.39(597926.69,340606.56) | 0.87 | 864.86(1022.06,743.71) | 771.71(944.10,638.39) | -0.51(-0.67,-0.35) |
| Micronesia (Federated States of) | male | 514.85(610.97,431.78) | 442.51(513.95,381.49) | -0.14 | 1083.94(1217.50,970.94) | 976.58(1109.91,861.36) | -0.47(-0.57,-0.37) |
| Fiji | male | 3599.92(4274.81,3050.42) | 3858.74(4535.81,3334.64) | 0.07 | 1095.06(1245.98,974.78) | 908.66(1047.80,798.98) | -0.88(-0.99,-0.78) |
| Finland | male | 17166.33(19269.44,15355.08) | 20373.91(22485.03,18513.51) | 0.19 | 722.67(830.46,630.67) | 645.70(761.93,549.69) | -0.43(-0.52,-0.34) |
| France | male | 245148.24(276311.16,219116.90) | 229163.34(261548.17,201704.02) | -0.07 | 859.30(983.16,754.86) | 664.58(807.57,546.88) | -1.13(-1.23,-1.03) |
| Gabon | male | 4041.59(5052.08,3251.66) | 6440.78(7851.29,5279.04) | 0.59 | 841.77(983.06,726.43) | 819.01(962.81,699.90) | -0.20(-0.29,-0.10) |
| Georgia | male | 19630.52(22895.55,17357.21) | 15861.88(17899.88,14112.03) | -0.19 | 773.49(895.61,683.56) | 829.66(963.91,721.13) | 0.29(0.19,0.38) |
| Germany | male | 348346.10(393917.81,294761.88) | 319931.74(347591.75,292778.01) | -0.08 | 853.95(979.39,733.97) | 648.19(756.45,551.41) | -0.80(-0.95,-0.65) |
| Ghana | male | 45103.17(57961.81,35124.87) | 94983.87(117925.22,76723.98) | 1.11 | 582.99(689.99,497.21) | 662.10(775.43,566.47) | 0.33(0.24,0.42) |
| Greece | male | 34674.57(39313.59,30805.11) | 40510.32(44148.27,37113.21) | 0.17 | 646.59(761.42,557.84) | 663.73(783.17,562.86) | 0.25(0.19,0.32) |
| Greenland | male | 505.49(610.43,419.36) | 529.10(609.98,461.08) | 0.05 | 2091.40(2480.85,1757.04) | 1725.28(2058.03,1486.74) | -0.77(-0.87,-0.66) |
| Grenada | male | 599.16(779.00,466.00) | 588.91(726.75,479.37) | -0.02 | 1236.82(1546.59,998.64) | 1307.16(1658.39,1039.12) | 0.13(0.08,0.17) |
| Guam | male | 756.61(928.33,626.46) | 991.61(1182.23,831.51) | 0.31 | 1229.14(1460.93,1041.89) | 1133.06(1356.03,950.79) | -0.39(-0.46,-0.32) |
| Guatemala | male | 63517.31(80232.36,48823.45) | 76801.66(97672.63,61787.77) | 0.21 | 1272.52(1535.01,1044.73) | 963.84(1180.09,806.11) | -1.16(-1.38,-0.94) |
| Guinea | male | 27752.88(36143.72,22158.95) | 54053.85(70991.73,41951.36) | 0.95 | 831.23(998.30,714.68) | 837.15(997.98,717.82) | -0.21(-0.29,-0.12) |
| Guinea-Bissau | male | 4224.27(5363.38,3358.41) | 6752.59(8489.95,5327.45) | 0.60 | 880.11(1016.74,763.99) | 761.24(886.95,654.10) | -0.63(-0.76,-0.50) |
| Guyana | male | 4820.01(6253.95,3790.20) | 4449.86(5479.07,3593.14) | -0.08 | 1157.75(1449.09,940.25) | 1210.66(1485.37,984.21) | 0.04(-0.01,0.08) |
| Haiti | male | 52626.92(65429.64,40652.88) | 82826.13(101725.45,66782.07) | 0.57 | 1318.61(1590.80,1071.75) | 1284.17(1529.39,1071.81) | -0.19(-0.25,-0.12) |
| Honduras | male | 36779.37(46891.35,28169.47) | 50507.55(63755.91,39824.44) | 0.37 | 1213.74(1473.49,996.12) | 1067.41(1299.86,875.40) | -0.68(-0.77,-0.60) |
| Hungary | male | 63636.99(70857.44,57290.27) | 67263.14(74097.06,60707.27) | 0.06 | 1216.07(1386.49,1083.32) | 1213.91(1385.79,1067.00) | -0.04(-0.14,0.07) |
| Iceland | male | 1373.06(1660.00,1141.73) | 1668.00(1941.39,1455.12) | 0.21 | 1134.57(1394.05,929.75) | 962.68(1178.33,801.27) | -0.52(-0.68,-0.37) |
| India | male | 3885314.68(4460216.56,3393161.78) | 6983117.54(7846861.04,6143879.92) | 0.80 | 1252.69(1406.40,1108.51) | 1161.84(1302.81,1022.46) | 0.06(-0.15,0.28) |
| Indonesia | male | 800513.27(987711.29,669970.30) | 1142721.03(1337129.22,989694.23) | 0.43 | 949.36(1102.47,828.96) | 982.08(1157.34,850.12) | 0.20(0.10,0.30) |
| Iran (Islamic Republic of) | male | 290331.52(365900.90,230056.26) | 376211.19(438955.12,324274.35) | 0.30 | 972.37(1133.83,829.59) | 947.98(1112.07,805.01) | -0.25(-0.31,-0.18) |
| Iraq | male | 96103.48(120622.13,76381.78) | 171896.93(213349.29,140358.48) | 0.79 | 1045.34(1220.49,899.46) | 846.98(1017.74,709.62) | -0.83(-0.91,-0.76) |
| Ireland | male | 18454.31(21353.19,15975.96) | 21310.85(24046.75,18848.14) | 0.15 | 1029.01(1182.70,892.24) | 830.80(972.28,705.66) | -0.82(-0.89,-0.76) |
| Israel | male | 17902.17(21447.22,14882.73) | 29837.12(35328.42,24843.94) | 0.67 | 729.95(858.75,618.02) | 621.09(740.29,513.75) | -0.68(-0.73,-0.63) |
| Italy | male | 262758.06(292277.19,235326.37) | 244128.43(266850.38,221580.76) | -0.07 | 840.96(961.26,744.05) | 672.39(793.86,564.65) | -0.94(-1.10,-0.77) |
| Jamaica | male | 16344.95(20901.98,12855.90) | 15174.21(18441.11,12656.69) | -0.07 | 1274.96(1591.70,1033.85) | 1246.70(1550.51,1012.80) | -0.26(-0.33,-0.19) |
| Japan | male | 963647.39(1091283.64,844906.10) | 1001954.21(1161589.73,855873.37) | 0.04 | 1554.09(1798.74,1349.58) | 1063.99(1257.37,903.71) | -1.93(-2.14,-1.72) |
| Jordan | male | 19602.47(24874.21,15437.70) | 62196.37(75832.62,50956.18) | 2.17 | 1072.50(1250.58,920.70) | 1171.30(1385.79,989.63) | 0.27(0.22,0.32) |
| Kazakhstan | male | 48431.22(55182.87,43037.06) | 65975.59(74968.45,58443.27) | 0.36 | 756.18(838.80,689.71) | 803.70(903.82,716.82) | 0.29(0.10,0.48) |
| Kenya | male | 107428.70(141666.11,81399.87) | 201785.49(256938.26,158588.84) | 0.88 | 874.28(1042.98,740.07) | 842.37(1014.26,711.77) | -0.18(-0.25,-0.11) |
| Kiribati | male | 426.66(500.66,362.27) | 543.41(639.13,470.92) | 0.27 | 1264.30(1421.85,1115.56) | 1089.86(1231.38,972.03) | -0.62(-0.67,-0.57) |
| Kuwait | male | 8846.59(10767.44,7231.28) | 20242.96(23955.26,17036.93) | 1.29 | 1006.90(1200.19,838.44) | 1049.98(1270.35,875.71) | 0.14(0.09,0.19) |
| Kyrgyzstan | male | 18901.86(22646.24,16203.61) | 25122.65(30167.05,21355.81) | 0.33 | 1035.42(1172.98,930.08) | 878.18(1016.60,772.10) | -0.67(-0.74,-0.60) |
| Lao People's Democratic Republic | male | 15285.89(18690.30,12524.70) | 22854.66(27023.43,19657.81) | 0.50 | 754.39(866.13,658.53) | 749.31(857.69,664.54) | -0.13(-0.21,-0.06) |
| Latvia | male | 16343.78(18645.05,14306.31) | 9505.16(10794.49,8403.06) | -0.42 | 1281.85(1468.89,1121.46) | 1010.71(1198.98,857.76) | -0.54(-0.67,-0.41) |
| Lebanon | male | 16544.70(20270.63,13563.24) | 28106.66(33080.03,24160.32) | 0.70 | 990.34(1166.71,846.71) | 1134.28(1331.14,975.15) | 0.49(0.47,0.50) |
| Lesotho | male | 5086.96(5771.15,4526.91) | 6064.85(6721.40,5477.37) | 0.19 | 822.30(894.29,752.26) | 821.86(895.68,755.69) | -0.09(-0.22,0.04) |
| Liberia | male | 7062.14(9047.32,5515.09) | 15091.97(19424.67,11778.29) | 1.14 | 628.55(754.20,529.10) | 617.05(757.70,511.89) | -0.12(-0.24,0.00) |
| Libya | male | 20267.68(25202.12,16368.35) | 30674.03(34971.70,26739.04) | 0.51 | 963.63(1116.65,834.41) | 1026.26(1187.00,886.83) | 0.23(0.18,0.28) |
| Lithuania | male | 16938.18(19197.66,15130.68) | 12546.30(14127.19,11145.84) | -0.26 | 977.32(1120.46,869.61) | 901.11(1061.20,771.22) | -0.17(-0.25,-0.08) |
| Luxembourg | male | 1861.98(2422.67,1620.53) | 2565.66(2878.90,2303.40) | 0.38 | 1037.51(1318.55,879.60) | 830.48(977.07,703.99) | -0.96(-1.03,-0.89) |
| North Macedonia | male | 11942.41(14118.51,9990.70) | 13725.26(15407.45,12315.48) | 0.15 | 1232.99(1463.52,1034.84) | 1182.64(1368.09,1032.70) | -0.38(-0.51,-0.25) |
| Madagascar | male | 118932.08(155803.73,93349.57) | 163099.53(205379.26,129539.70) | 0.37 | 1599.14(1981.55,1331.56) | 1207.02(1419.19,1027.27) | -1.28(-1.39,-1.16) |
| Malawi | male | 38201.17(48617.45,29492.03) | 72317.20(91861.74,56404.54) | 0.89 | 764.13(897.21,650.15) | 821.47(972.57,696.84) | 0.28(0.21,0.35) |
| Malaysia | male | 61636.53(73413.29,52625.54) | 105684.75(124785.36,91890.66) | 0.71 | 821.01(925.33,736.17) | 734.46(867.83,635.17) | -0.49(-0.54,-0.43) |
| Maldives | male | 1162.06(1429.76,956.52) | 2052.67(2403.75,1747.22) | 0.77 | 1084.91(1239.09,955.18) | 997.71(1161.00,844.87) | -0.47(-0.54,-0.41) |
| Mali | male | 28433.39(35562.07,23018.31) | 76208.54(96577.46,60534.86) | 1.68 | 677.04(776.99,597.96) | 717.17(835.02,623.16) | 0.16(0.08,0.24) |
| Malta | male | 1585.35(1791.39,1393.69) | 1905.78(2100.40,1736.61) | 0.20 | 882.70(1008.35,770.40) | 752.36(883.49,635.40) | -0.61(-0.64,-0.59) |
| Marshall Islands | male | 235.29(281.79,197.51) | 252.72(293.12,218.10) | 0.07 | 1189.40(1323.21,1073.28) | 1000.87(1131.47,887.37) | -0.69(-0.79,-0.58) |
| Mauritania | male | 11061.65(14475.72,8416.69) | 19262.73(24988.40,15162.40) | 0.74 | 1016.84(1230.64,852.01) | 940.91(1145.69,792.17) | -0.41(-0.51,-0.31) |
| Mauritius | male | 4770.36(5543.73,4125.69) | 6193.10(7055.47,5411.30) | 0.30 | 1066.18(1206.40,944.44) | 960.32(1111.16,830.12) | -0.55(-0.61,-0.49) |
| Mexico | male | 431549.79(548264.38,342701.21) | 609508.56(736603.52,509582.12) | 0.41 | 1131.79(1330.20,964.60) | 1088.29(1312.73,912.21) | -0.29(-0.45,-0.12) |
| Republic of Moldova | male | 22448.14(26044.80,19704.28) | 16688.25(19092.54,14782.47) | -0.26 | 1087.17(1251.97,965.18) | 928.70(1100.97,793.81) | -0.62(-0.69,-0.55) |
| Mongolia | male | 6983.85(8551.07,5819.82) | 10871.12(12850.93,9176.34) | 0.56 | 778.78(877.85,687.66) | 738.50(847.17,640.72) | -0.21(-0.28,-0.14) |
| Montenegro | male | 2646.64(3161.25,2236.89) | 3110.00(3594.06,2716.34) | 0.18 | 889.42(1056.35,753.80) | 980.35(1160.36,831.04) | 0.38(0.26,0.49) |
| Morocco | male | 87445.23(104318.88,72703.75) | 147359.70(172299.38,127277.59) | 0.69 | 720.68(824.80,626.33) | 876.33(1021.20,759.41) | 0.63(0.56,0.69) |
| Mozambique | male | 67238.62(86412.27,53317.47) | 153367.50(198094.89,119505.02) | 1.28 | 938.72(1114.23,801.09) | 1020.46(1208.52,869.40) | 0.30(0.27,0.32) |
| Myanmar | male | 142611.74(164865.13,124152.53) | 205761.84(230222.85,186072.98) | 0.44 | 861.02(945.33,782.43) | 898.89(994.48,822.20) | 0.14(0.07,0.20) |
| Namibia | male | 4792.64(5601.81,4169.08) | 8153.71(9387.30,7095.52) | 0.70 | 920.66(1015.90,833.85) | 923.44(1027.57,825.35) | -0.10(-0.22,0.03) |
| Nepal | male | 46985.19(51748.55,42633.79) | 91865.41(100999.97,82817.70) | 0.96 | 770.88(835.08,702.54) | 812.93(890.38,735.92) | 0.04(-0.05,0.13) |
| Netherlands | male | 49267.26(53008.10,45665.51) | 64709.55(68903.18,60534.40) | 0.31 | 661.91(730.36,601.29) | 580.68(647.23,521.71) | -0.56(-0.61,-0.50) |
| New Zealand | male | 27581.56(32215.36,23668.90) | 34109.05(39443.89,29447.97) | 0.24 | 1691.78(1998.22,1436.75) | 1350.97(1631.49,1128.13) | -0.86(-0.93,-0.80) |
| Nicaragua | male | 28096.73(36974.00,20945.56) | 30192.61(37677.98,24006.21) | 0.07 | 1133.07(1401.82,916.34) | 1004.30(1221.63,829.96) | -0.54(-0.67,-0.41) |
| Niger | male | 35241.70(45004.04,27344.59) | 92213.67(121313.83,70510.74) | 1.62 | 817.46(957.32,704.45) | 735.98(872.85,631.51) | -0.46(-0.58,-0.34) |
| Nigeria | male | 465860.58(602154.19,364946.45) | 941471.07(1249477.05,718186.33) | 1.02 | 976.40(1182.81,826.47) | 824.82(1019.04,690.47) | -0.76(-0.89,-0.64) |
| Democratic People's Republic of Korea | male | 92303.87(112634.47,77936.91) | 119089.43(133301.32,107146.20) | 0.29 | 1068.29(1235.04,941.48) | 1004.19(1156.27,882.40) | -0.26(-0.29,-0.22) |
| Northern Mariana Islands | male | 228.05(275.81,189.76) | 240.74(282.42,204.09) | 0.06 | 1208.53(1435.97,1029.09) | 1127.58(1350.95,949.95) | -0.14(-0.24,-0.04) |
| Norway | male | 28096.79(31828.11,24925.71) | 29615.23(33419.61,26456.20) | 0.05 | 1303.22(1524.50,1132.94) | 1034.21(1224.80,877.20) | -0.85(-0.97,-0.74) |
| Oman | male | 8156.87(10324.12,6578.93) | 21851.00(25965.68,18337.68) | 1.68 | 762.86(913.64,642.52) | 1006.71(1198.08,847.94) | 1.05(0.96,1.15) |
| Pakistan | male | 359953.20(414600.25,314629.75) | 559199.37(631873.48,495865.90) | 0.55 | 823.05(914.27,736.10) | 716.04(789.04,644.94) | -0.46(-0.52,-0.40) |
| Palestine | male | 9970.01(12569.48,7886.24) | 23747.95(29267.23,19325.75) | 1.38 | 1048.52(1205.98,906.41) | 1094.39(1286.02,929.23) | 0.09(0.02,0.15) |
| Panama | male | 15264.52(18883.08,12289.19) | 25035.84(30152.91,20720.67) | 0.64 | 1222.96(1470.01,1022.93) | 1224.99(1473.39,1016.99) | -0.12(-0.17,-0.07) |
| Papua New Guinea | male | 27111.04(31847.88,23287.36) | 60447.33(69234.96,53289.11) | 1.23 | 1537.17(1720.03,1385.15) | 1454.12(1601.89,1316.20) | -0.28(-0.34,-0.23) |
| Paraguay | male | 21318.52(27678.73,16230.49) | 34090.41(43423.61,26760.45) | 0.60 | 901.60(1109.18,730.82) | 1048.70(1319.21,834.88) | 0.48(0.39,0.57) |
| Peru | male | 167152.31(212455.05,129865.07) | 255584.30(309632.14,213014.25) | 0.53 | 1490.47(1810.65,1217.17) | 1602.85(1937.32,1330.85) | 0.17(0.10,0.25) |
| Philippines | male | 409869.54(530333.78,324968.23) | 590375.72(733105.25,487192.00) | 0.44 | 1176.53(1434.79,980.32) | 1084.10(1300.57,923.58) | -0.24(-0.29,-0.19) |
| Poland | male | 322292.42(368810.85,283325.06) | 274298.62(308321.34,244291.45) | -0.15 | 1740.12(1985.68,1536.76) | 1366.53(1615.48,1175.12) | -1.07(-1.21,-0.93) |
| Portugal | male | 49410.49(58120.79,43664.31) | 49577.23(54294.81,45279.84) | 0.00 | 989.35(1180.13,862.95) | 871.97(1022.50,739.39) | -0.48(-0.61,-0.35) |
| Puerto Rico | male | 27481.29(33487.01,22320.78) | 23575.97(27108.03,20501.27) | -0.14 | 1598.34(1960.69,1302.48) | 1576.73(1934.29,1286.96) | -0.06(-0.12,0.00) |
| Qatar | male | 1851.13(2239.04,1539.63) | 12799.01(14833.48,10894.04) | 5.91 | 857.41(1013.61,725.27) | 912.79(1085.03,775.09) | 0.17(0.13,0.22) |
| Romania | male | 156568.88(179422.51,137554.69) | 137406.27(155359.16,119841.58) | -0.12 | 1319.29(1512.34,1158.43) | 1254.73(1453.14,1082.00) | -0.25(-0.33,-0.17) |
| Russian Federation | male | 799961.32(927179.51,704523.97) | 609606.27(703463.84,534606.16) | -0.24 | 1158.90(1336.50,1019.29) | 865.36(1027.35,739.51) | -1.35(-1.51,-1.19) |
| Rwanda | male | 62712.65(82428.15,48367.87) | 78754.52(99439.48,61999.20) | 0.26 | 1576.16(1901.64,1318.83) | 1321.31(1572.95,1114.99) | -0.65(-0.70,-0.60) |
| Saint Lucia | male | 1034.92(1307.77,814.23) | 1074.41(1243.84,917.12) | 0.04 | 1392.33(1693.36,1137.57) | 1445.92(1729.00,1191.99) | -0.02(-0.09,0.06) |
| Saint Vincent and the Grenadines | male | 681.74(885.47,529.32) | 601.99(728.32,501.78) | -0.12 | 1108.22(1399.31,897.03) | 1176.45(1464.55,949.42) | 0.16(0.10,0.22) |
| Samoa | male | 779.48(934.13,658.44) | 907.62(1095.90,762.39) | 0.16 | 1033.11(1173.14,914.21) | 914.37(1062.66,795.64) | -0.58(-0.67,-0.48) |
| Sao Tome and Principe | male | 694.84(886.55,550.65) | 960.60(1153.84,801.27) | 0.38 | 1099.00(1286.85,946.01) | 1097.33(1260.12,971.65) | -0.19(-0.25,-0.14) |
| Saudi Arabia | male | 59592.73(71784.55,49012.64) | 162271.55(189161.27,139927.79) | 1.72 | 810.04(928.17,705.17) | 1121.06(1309.90,956.77) | 1.26(1.21,1.32) |
| Senegal | male | 23266.22(29605.00,18674.74) | 45180.84(56581.66,36274.08) | 0.94 | 651.46(748.86,570.75) | 628.75(735.92,542.43) | -0.20(-0.37,-0.02) |
| Serbia | male | 43520.83(49089.15,38817.71) | 49830.33(55106.72,45009.01) | 0.14 | 940.32(1092.91,824.90) | 983.45(1128.10,860.70) | 0.24(0.19,0.28) |
| Seychelles | male | 241.67(289.60,204.45) | 379.58(439.12,334.59) | 0.57 | 720.33(837.88,632.79) | 777.82(917.54,673.68) | 0.20(0.14,0.27) |
| Sierra Leone | male | 15449.95(19651.14,12448.44) | 29331.69(37114.86,23256.53) | 0.90 | 802.28(953.90,690.46) | 714.38(849.34,611.77) | -0.51(-0.61,-0.40) |
| Singapore | male | 18505.03(20962.91,16274.59) | 32994.10(38228.03,28995.15) | 0.78 | 1464.96(1659.32,1289.44) | 1101.05(1296.09,951.26) | -0.92(-1.05,-0.80) |
| Slovakia | male | 22239.27(25591.58,19477.93) | 29626.13(33444.87,26150.62) | 0.33 | 885.46(1030.73,770.47) | 1013.36(1169.25,883.74) | 0.49(0.44,0.54) |
| Slovenia | male | 13239.19(15001.84,11851.45) | 15666.46(17510.92,13985.39) | 0.18 | 1409.30(1607.34,1249.34) | 1277.56(1493.04,1103.07) | -0.29(-0.34,-0.25) |
| Solomon Islands | male | 1609.59(1969.42,1328.70) | 3021.55(3764.94,2509.50) | 0.88 | 1006.46(1143.63,886.76) | 1016.33(1188.59,878.99) | -0.13(-0.31,0.05) |
| Somalia | male | 46709.44(60144.91,35999.48) | 104854.28(138612.13,81474.19) | 1.24 | 1156.31(1366.92,978.58) | 983.27(1182.77,841.58) | -0.60(-0.67,-0.53) |
| South Africa | male | 197789.39(239396.92,159769.26) | 278239.14(329234.64,232625.50) | 0.41 | 1379.69(1585.03,1172.72) | 1216.04(1422.27,1029.14) | -0.90(-1.24,-0.55) |
| Republic of Korea | male | 148804.75(174388.12,128631.75) | 240417.84(265560.59,218028.32) | 0.62 | 894.89(1038.08,789.43) | 874.54(1032.94,757.40) | -0.29(-0.37,-0.22) |
| South Sudan | male | 34393.20(43760.79,27263.98) | 47087.45(60504.93,36433.74) | 0.37 | 1060.87(1262.88,900.82) | 947.28(1130.19,805.34) | -0.45(-0.53,-0.37) |
| Spain | male | 157474.25(176582.51,141957.66) | 207171.31(225776.91,191444.06) | 0.32 | 790.37(913.36,702.59) | 792.67(923.29,684.33) | 0.16(0.06,0.26) |
| Sri Lanka | male | 60557.84(70183.54,52615.76) | 102365.23(114564.02,92170.05) | 0.69 | 814.15(924.69,723.35) | 972.61(1103.44,869.70) | 0.59(0.55,0.63) |
| Sudan | male | 99874.36(126986.76,76157.91) | 194432.39(244215.62,156670.97) | 0.95 | 919.61(1093.74,770.16) | 990.90(1176.36,839.07) | 0.17(0.11,0.23) |
| Suriname | male | 2248.42(2878.63,1780.77) | 3141.05(3890.26,2595.48) | 0.40 | 1109.67(1381.98,902.88) | 1176.30(1468.99,962.75) | 0.20(0.16,0.24) |
| Eswatini | male | 4112.68(5099.60,3355.92) | 5647.89(6689.52,4780.36) | 0.37 | 1312.21(1481.97,1175.66) | 1273.75(1431.57,1132.84) | -0.26(-0.33,-0.18) |
| Sweden | male | 46999.91(52950.54,41491.77) | 47774.37(54571.80,41934.37) | 0.02 | 1033.48(1218.24,888.04) | 830.77(1014.65,679.20) | -0.76(-1.07,-0.46) |
| Switzerland | male | 32283.39(36195.92,28778.08) | 33158.61(37116.83,29603.22) | 0.03 | 958.98(1114.77,829.31) | 731.01(885.73,606.63) | -1.10(-1.15,-1.04) |
| Syrian Arab Republic | male | 51674.13(63556.67,42368.05) | 60858.35(70372.70,52321.73) | 0.18 | 789.01(907.35,694.64) | 897.14(1026.77,784.02) | 0.60(0.45,0.75) |
| Taiwan (Province of China) | male | 84062.62(101544.64,71965.92) | 101144.38(114922.41,89015.60) | 0.20 | 897.12(1075.70,769.53) | 878.35(1089.63,721.84) | -0.30(-0.43,-0.18) |
| Tajikistan | male | 21176.45(25606.94,17899.46) | 35272.24(42306.48,29410.71) | 0.67 | 1032.54(1171.64,906.11) | 922.37(1060.32,801.78) | -0.43(-0.58,-0.29) |
| United Republic of Tanzania | male | 165134.66(218180.63,125359.32) | 339840.21(448312.84,257169.16) | 1.06 | 1121.38(1377.18,927.96) | 1107.62(1346.52,909.85) | -0.04(-0.08,0.01) |
| Thailand | male | 220595.06(256932.61,190692.23) | 282490.06(319282.98,252118.95) | 0.28 | 911.00(1038.18,812.54) | 861.71(1029.53,726.24) | -0.49(-0.61,-0.37) |
| Bahamas | male | 1398.90(1787.96,1104.29) | 1793.38(2180.89,1483.20) | 0.28 | 1130.30(1411.56,915.34) | 1183.54(1479.90,948.42) | 0.15(0.12,0.19) |
| Gambia | male | 3873.25(4922.68,3028.97) | 7924.12(10096.72,6216.60) | 1.05 | 790.63(925.26,685.01) | 733.70(873.56,629.76) | -0.34(-0.47,-0.21) |
| Timor-Leste | male | 3072.93(3884.64,2466.84) | 5233.01(6291.86,4355.29) | 0.70 | 751.71(878.01,650.71) | 821.37(941.77,722.27) | 0.34(0.25,0.43) |
| Togo | male | 16259.36(21273.22,12463.35) | 32352.79(41273.77,25373.32) | 0.99 | 892.47(1054.08,768.74) | 847.60(1016.18,719.49) | -0.23(-0.31,-0.16) |
| Tonga | male | 527.33(657.02,429.94) | 519.84(632.39,434.74) | -0.01 | 1144.46(1343.20,988.96) | 1051.15(1232.98,911.95) | -0.43(-0.53,-0.33) |
| Trinidad and Tobago | male | 6218.27(7912.71,4928.79) | 6323.33(7502.23,5308.60) | 0.02 | 995.16(1232.71,816.22) | 1042.50(1295.35,839.64) | 0.28(0.19,0.37) |
| Tunisia | male | 37496.27(45926.16,31307.30) | 59410.50(68042.49,51786.94) | 0.58 | 922.88(1071.46,807.64) | 1057.95(1231.86,922.48) | 0.44(0.36,0.51) |
| Turkey | male | 294455.93(347477.46,246500.69) | 484731.93(538777.46,437110.29) | 0.65 | 1043.75(1192.29,913.09) | 1251.64(1423.56,1110.25) | 0.61(0.50,0.73) |
| Turkmenistan | male | 13268.16(16340.22,11095.47) | 15799.94(19240.62,13011.82) | 0.19 | 830.33(953.85,737.62) | 647.90(771.81,544.40) | -1.05(-1.29,-0.81) |
| Uganda | male | 128032.48(167910.42,97266.34) | 261614.29(346261.88,197157.09) | 1.04 | 1267.62(1518.74,1053.77) | 1201.08(1462.29,1004.66) | -0.28(-0.31,-0.24) |
| Ukraine | male | 374986.23(434168.06,328566.52) | 206046.81(238360.35,179387.67) | -0.45 | 1522.78(1779.93,1318.15) | 1052.10(1287.41,874.11) | -1.73(-1.91,-1.55) |
| United Arab Emirates | male | 13278.60(15766.31,11241.83) | 72232.59(82977.19,62939.74) | 4.44 | 1329.76(1539.38,1152.21) | 1372.00(1590.94,1192.30) | 0.11(-0.04,0.25) |
| United Kingdom | male | 322420.43(371277.28,283721.16) | 339223.07(383929.13,301581.01) | 0.05 | 1197.68(1428.59,1007.98) | 974.55(1175.36,813.17) | -0.96(-1.07,-0.85) |
| United States of America | male | 2598984.41(3081538.63,2223106.32) | 4290524.12(4851530.75,3739988.82) | 0.65 | 2183.49(2653.21,1831.16) | 2483.75(2894.87,2136.55) | 1.35(0.90,1.80) |
| Uruguay | male | 19464.22(22625.12,17080.78) | 20668.03(23731.97,18392.44) | 0.06 | 1246.18(1456.09,1086.97) | 1199.31(1420.39,1036.27) | -0.18(-0.23,-0.13) |
| Uzbekistan | male | 90767.17(108114.92,77009.22) | 128332.86(152371.89,109169.55) | 0.41 | 1108.66(1258.14,976.20) | 923.63(1061.11,805.87) | -0.96(-1.07,-0.85) |
| Vanuatu | male | 717.07(864.19,602.50) | 1303.98(1667.87,1017.52) | 0.82 | 1018.13(1144.85,911.44) | 941.86(1151.51,771.84) | -0.46(-0.72,-0.20) |
| Venezuela (Bolivarian Republic of) | male | 112166.96(143657.09,87064.37) | 139106.74(167258.96,115826.63) | 0.24 | 1118.69(1371.98,914.55) | 1069.65(1293.83,881.09) | -0.30(-0.35,-0.25) |
| Viet nam | male | 230566.21(284771.78,187849.56) | 414951.64(475267.54,369449.29) | 0.80 | 780.11(897.38,684.73) | 984.69(1128.39,874.19) | 0.80(0.64,0.97) |
| Virginia | male | 62558.89(74925.24,53131.60) | 115663.08(131670.26,101130.84) | 0.85 | 2209.06(2698.10,1841.76) | 2553.20(2977.33,2190.56) | 1.49(1.05,1.93) |
| Yemen | male | 67390.67(84142.28,52982.82) | 143915.53(179137.35,115106.33) | 1.14 | 931.93(1066.76,812.06) | 972.21(1128.85,840.70) | 0.20(0.15,0.25) |
| Zambia | male | 27232.10(34349.79,21438.71) | 66168.77(84487.86,51648.94) | 1.43 | 685.76(799.31,592.12) | 754.02(891.29,637.89) | 0.34(0.17,0.52) |
| Zimbabwe | male | 27372.11(32930.86,23017.71) | 39481.75(46569.47,33653.97) | 0.44 | 689.68(768.55,624.82) | 689.93(772.44,620.26) | -0.11(-0.29,0.07) |
| Monaco | male | 138.88(152.73,122.51) | 171.53(186.72,157.58) | 0.24 | 818.28(954.38,699.49) | 752.07(882.88,637.98) | -0.29(-0.31,-0.26) |
| San Marino | male | 90.86(102.59,81.05) | 129.04(143.42,116.45) | 0.42 | 775.38(908.14,665.87) | 722.95(861.05,609.94) | -0.25(-0.26,-0.24) |
| Saint Kitts and Nevis | male | 249.62(321.25,193.22) | 299.58(363.65,249.84) | 0.20 | 1140.45(1435.15,913.55) | 1220.11(1527.25,984.19) | 0.20(0.16,0.24) |
| Cook Islands | male | 110.93(135.41,93.54) | 114.66(131.13,99.66) | 0.03 | 1199.49(1406.59,1042.16) | 1190.45(1394.52,1021.54) | 0.17(0.03,0.31) |
| Nauru | male | 52.32(63.57,43.67) | 47.92(59.77,38.61) | -0.08 | 1172.65(1318.01,1055.66) | 1114.64(1308.84,961.09) | -0.36(-0.54,-0.17) |
| Niue | male | 12.62(15.25,10.69) | 9.98(11.42,8.64) | -0.21 | 1096.72(1286.14,961.41) | 1157.83(1345.30,995.60) | 0.18(0.04,0.32) |
| Palau | male | 99.05(113.37,87.15) | 138.25(159.13,120.85) | 0.40 | 1474.89(1669.95,1316.37) | 1390.28(1582.87,1223.44) | -0.31(-0.36,-0.26) |
| Tokelau | male | 9.34(11.74,7.49) | 8.51(10.14,7.20) | -0.09 | 1113.28(1343.09,939.03) | 1158.60(1360.77,995.36) | 0.34(0.19,0.50) |
| Tuvalu | male | 44.17(51.43,38.16) | 62.79(74.67,52.87) | 0.42 | 1058.46(1182.51,951.74) | 1129.98(1326.85,964.48) | -0.04(-0.26,0.18) |
| Afghanistan | female | 62831.15(73594.68,53857.01) | 195084.93(232563.49,165630.83) | 2.10 | 1141.37(1277.07,1015.39) | 1182.12(1336.11,1060.60) | 0.09(0.04,0.15) |
| Albania | female | 11264.28(13125.40,9800.15) | 14019.68(16066.30,12063.27) | 0.24 | 785.69(895.61,689.40) | 892.12(1037.59,764.92) | 0.45(0.35,0.56) |
| Algeria | female | 99364.60(120219.73,82999.05) | 185878.08(218619.67,160260.66) | 0.87 | 840.28(969.61,735.07) | 957.05(1117.54,829.17) | 0.40(0.29,0.50) |
| American Samoa | female | 210.05(261.87,167.22) | 240.34(285.02,197.09) | 0.14 | 978.56(1147.74,814.11) | 917.55(1074.48,755.30) | 0.12(-0.12,0.35) |
| Andorra | female | 165.15(193.58,141.35) | 315.15(351.93,281.01) | 0.91 | 713.46(847.89,600.82) | 734.99(865.26,618.79) | 0.06(0.04,0.08) |
| Angola | female | 55659.12(67100.57,45454.26) | 127734.83(154910.01,105129.76) | 1.29 | 1036.66(1183.89,900.27) | 862.26(980.80,755.90) | -0.79(-0.85,-0.73) |
| Antigua and Barbuda | female | 339.64(420.31,277.67) | 453.30(537.96,382.27) | 0.33 | 1054.98(1307.56,866.21) | 1178.48(1449.79,947.32) | 0.43(0.37,0.49) |
| Argentina | female | 185081.15(217832.99,160883.90) | 286034.15(330157.31,248265.18) | 0.55 | 1076.25(1265.58,933.79) | 1196.29(1405.27,1023.83) | 0.46(0.38,0.54) |
| Armenia | female | 13076.18(15060.87,11310.99) | 15267.04(17071.52,13356.79) | 0.17 | 795.84(907.34,695.18) | 824.66(938.67,713.21) | 0.18(0.07,0.28) |
| Australia | female | 93022.18(107464.60,81938.32) | 158728.04(178624.07,141563.19) | 0.71 | 1105.72(1306.96,957.06) | 1064.79(1244.41,923.94) | -0.38(-0.55,-0.21) |
| Austria | female | 32973.90(36569.10,29587.44) | 36661.10(40127.98,33433.58) | 0.11 | 722.49(826.76,632.20) | 683.27(795.95,586.27) | -0.21(-0.28,-0.13) |
| Azerbaijan | female | 24537.08(28686.04,21310.07) | 35180.10(40210.35,30849.40) | 0.43 | 706.52(808.76,620.14) | 714.91(824.74,619.92) | 0.05(-0.09,0.19) |
| Bahrain | female | 2124.14(2544.26,1786.20) | 5747.54(6606.87,5025.68) | 1.71 | 1234.89(1396.38,1092.07) | 1256.84(1445.39,1099.18) | 0.05(-0.04,0.13) |
| Bangladesh | female | 303197.84(344605.91,269822.10) | 607481.10(673712.04,544596.86) | 1.00 | 880.99(957.11,799.77) | 878.74(974.84,784.44) | -0.27(-0.36,-0.19) |
| Barbados | female | 1477.74(1746.47,1253.99) | 1824.89(2104.85,1568.53) | 0.23 | 1165.08(1402.00,977.18) | 1315.50(1604.20,1079.46) | 0.49(0.43,0.56) |
| Belarus | female | 71127.16(80363.20,63416.64) | 51057.08(59327.59,44523.05) | -0.28 | 1145.80(1312.24,1002.40) | 898.84(1067.55,755.91) | -1.05(-1.27,-0.83) |
| Belgium | female | 46681.71(51950.98,42375.79) | 52350.19(56960.64,47970.38) | 0.12 | 795.26(910.73,700.67) | 717.80(825.59,619.00) | -0.39(-0.55,-0.22) |
| Belize | female | 1329.08(1657.56,1053.70) | 2502.11(3031.19,2047.04) | 0.88 | 1236.24(1480.34,1030.89) | 1264.41(1514.35,1047.72) | 0.00(-0.04,0.05) |
| Benin | female | 19191.89(23819.51,15716.02) | 47498.93(57922.85,39126.64) | 1.47 | 786.08(904.10,689.20) | 775.09(884.09,682.48) | -0.13(-0.23,-0.04) |
| Bermuda | female | 323.15(383.59,273.80) | 413.68(474.26,353.06) | 0.28 | 1186.79(1452.58,975.92) | 1308.59(1588.61,1068.61) | 0.36(0.29,0.42) |
| Bhutan | female | 1860.38(2070.54,1673.28) | 2915.75(3229.42,2636.62) | 0.57 | 998.08(1080.09,921.09) | 975.08(1074.57,881.88) | -0.27(-0.32,-0.21) |
| Bolivia (Plurinational State of) | female | 52112.84(63917.09,42172.55) | 87104.84(104377.94,72235.33) | 0.67 | 1506.46(1759.83,1279.85) | 1524.90(1786.35,1290.91) | -0.16(-0.26,-0.07) |
| Bosnia and Herzegovina | female | 23091.35(26462.38,20204.19) | 21909.09(24788.50,19391.47) | -0.05 | 1018.34(1165.39,891.04) | 1061.06(1223.78,923.70) | 0.20(0.14,0.25) |
| Botswana | female | 4286.09(5036.10,3666.19) | 9298.29(10845.28,7993.06) | 1.17 | 747.73(838.94,667.15) | 880.60(1007.55,769.38) | 0.63(0.50,0.77) |
| Brazil | female | 1145125.24(1447557.45,899370.21) | 1265772.77(1529471.46,1028284.97) | 0.11 | 1510.12(1844.65,1228.88) | 1282.47(1593.25,1005.68) | -1.03(-1.21,-0.84) |
| Brunei Darussalam | female | 1043.05(1266.77,867.99) | 1708.53(2010.01,1475.87) | 0.64 | 1104.05(1298.18,962.19) | 1005.54(1173.23,874.96) | -0.41(-0.47,-0.34) |
| Bulgaria | female | 48426.29(55263.05,42058.00) | 44767.28(50889.45,39339.71) | -0.08 | 997.90(1150.93,867.29) | 1036.45(1210.16,897.72) | 0.10(0.00,0.21) |
| Burkina Faso | female | 38444.92(48012.60,30623.96) | 88490.23(111860.57,71488.36) | 1.30 | 716.37(835.96,611.70) | 731.24(869.90,631.68) | -0.02(-0.09,0.04) |
| Burundi | female | 34731.37(42764.61,27752.57) | 59772.45(74032.29,47564.09) | 0.72 | 1076.70(1255.95,911.92) | 925.23(1073.48,789.14) | -0.56(-0.60,-0.53) |
| Cambodia | female | 28253.94(35194.90,22823.67) | 47499.13(56384.03,40615.56) | 0.68 | 526.65(609.97,454.07) | 602.36(707.55,522.59) | 0.44(0.31,0.56) |
| Cameroon | female | 37335.49(45101.07,31324.66) | 96635.13(118118.66,79433.75) | 1.59 | 786.22(887.10,696.42) | 731.48(838.33,641.90) | -0.31(-0.44,-0.19) |
| Canada | female | 196431.51(225873.41,172393.66) | 332422.30(377937.46,290929.25) | 0.69 | 1377.40(1637.71,1192.54) | 1427.35(1654.59,1227.73) | 0.33(0.16,0.51) |
| Cabo Verde | female | 1350.40(1635.54,1119.75) | 1858.45(2238.81,1584.00) | 0.38 | 702.22(810.24,610.59) | 684.58(819.11,584.60) | -0.11(-0.32,0.09) |
| Central African Republic | female | 13565.13(16291.64,11146.20) | 22608.97(26946.64,19028.71) | 0.67 | 974.18(1105.62,851.29) | 884.90(993.22,783.33) | -0.33(-0.35,-0.31) |
| Chad | female | 18282.58(21972.57,15162.84) | 49212.79(61414.53,39674.26) | 1.69 | 616.20(693.92,546.12) | 628.61(721.13,551.46) | 0.03(-0.10,0.17) |
| Chile | female | 61479.11(72528.30,52606.46) | 108191.62(122283.03,95028.06) | 0.76 | 967.54(1129.58,834.38) | 1062.24(1244.41,914.50) | 0.30(0.25,0.35) |
| China | female | 3953364.95(4636334.93,3454756.78) | 5458018.27(6145231.59,4847601.47) | 0.38 | 803.97(921.67,709.18) | 689.41(804.04,596.20) | -0.73(-1.05,-0.41) |
| Colombia | female | 174644.26(213186.00,141627.03) | 254050.53(293103.16,219826.64) | 0.45 | 1101.98(1298.55,938.00) | 1045.39(1231.70,879.33) | -0.22(-0.36,-0.08) |
| Comoros | female | 2496.12(3071.64,2010.18) | 3200.20(3836.29,2642.64) | 0.28 | 958.55(1123.82,824.02) | 898.68(1062.69,759.70) | -0.31(-0.36,-0.26) |
| Congo | female | 11193.88(13477.25,9307.07) | 22152.18(26331.02,18643.25) | 0.98 | 936.16(1068.62,820.95) | 935.85(1066.36,826.40) | -0.02(-0.08,0.04) |
| Costa Rica | female | 23536.40(28467.45,19055.01) | 35843.66(41237.97,31229.03) | 0.52 | 1606.97(1878.51,1359.61) | 1507.75(1769.13,1285.63) | -0.37(-0.51,-0.24) |
| Côte d'Ivoire | female | 51461.74(66018.29,41447.54) | 107742.14(134253.11,87535.18) | 1.09 | 862.75(1011.76,746.54) | 866.53(1014.36,742.51) | -0.03(-0.09,0.03) |
| Croatia | female | 31902.51(36516.79,27743.87) | 33358.08(37506.16,29646.74) | 0.05 | 1116.30(1292.97,969.05) | 1150.09(1329.80,1009.11) | 0.14(0.09,0.18) |
| Cuba | female | 68577.37(83916.51,57105.48) | 68279.54(78378.60,59487.99) | 0.00 | 1327.93(1615.95,1113.22) | 1364.46(1655.05,1117.93) | 0.08(0.04,0.13) |
| Cyprus | female | 3268.14(3790.40,2813.16) | 5576.42(6322.06,4892.86) | 0.71 | 861.54(1013.64,734.27) | 836.09(1000.98,710.60) | 0.02(-0.03,0.07) |
| Czechia | female | 48026.31(54839.37,41861.89) | 70356.98(79618.97,61051.89) | 0.46 | 849.68(989.41,732.89) | 1026.99(1177.91,889.95) | 0.74(0.70,0.77) |
| Democratic Republic of the Congo | female | 183523.16(223734.53,149797.48) | 369425.15(441506.36,307643.20) | 1.01 | 926.86(1061.63,809.68) | 911.91(1031.99,801.67) | -0.10(-0.16,-0.04) |
| Denmark | female | 21446.41(23725.62,19237.03) | 28934.19(31315.66,26731.76) | 0.35 | 755.20(867.82,657.68) | 788.32(912.16,680.89) | -0.05(-0.19,0.09) |
| Djibouti | female | 2452.47(3082.59,1939.67) | 4961.47(6031.66,4122.85) | 1.02 | 970.37(1135.67,826.05) | 887.17(1047.57,753.95) | -0.40(-0.48,-0.32) |
| Dominica | female | 455.40(551.65,373.94) | 375.70(441.13,318.26) | -0.18 | 1142.95(1376.36,946.10) | 1210.58(1464.01,1000.47) | 0.13(0.04,0.22) |
| Dominican Republic | female | 39070.48(47931.33,31247.23) | 51212.69(61667.59,42740.17) | 0.31 | 941.27(1125.37,775.72) | 961.91(1161.06,800.38) | 0.30(0.12,0.47) |
| Ecuador | female | 70302.28(88557.96,54493.98) | 115744.91(137687.17,96969.66) | 0.65 | 1298.48(1574.59,1051.52) | 1397.70(1657.58,1172.80) | 0.17(-0.08,0.43) |
| Egypt | female | 248909.47(302369.91,205566.34) | 452342.11(530001.38,387717.63) | 0.82 | 941.67(1088.39,812.49) | 1064.99(1220.37,927.89) | 0.35(0.25,0.45) |
| El Salvador | female | 44580.42(55058.64,35402.96) | 42803.34(49956.70,36762.14) | -0.04 | 1526.58(1824.60,1267.86) | 1314.16(1550.51,1123.81) | -0.70(-0.84,-0.57) |
| Equatorial Guinea | female | 2120.29(2527.94,1742.98) | 5166.15(6249.29,4277.79) | 1.44 | 913.98(1029.69,798.27) | 927.81(1061.21,809.97) | 0.08(0.03,0.13) |
| Eritrea | female | 16210.05(19484.76,13106.30) | 31039.58(37560.96,25252.62) | 0.91 | 988.69(1131.65,846.82) | 925.81(1078.82,796.29) | -0.37(-0.44,-0.31) |
| Estonia | female | 6822.78(7772.76,5967.28) | 6383.48(7273.90,5547.27) | -0.06 | 763.01(878.32,654.75) | 763.31(886.67,656.16) | -0.17(-0.33,0.00) |
| Ethiopia | female | 240452.00(303523.45,193487.63) | 386967.25(486553.09,305082.43) | 0.61 | 845.32(1004.21,726.61) | 693.31(825.51,586.41) | -0.83(-0.93,-0.72) |
| Micronesia (Federated States of) | female | 450.69(526.85,377.20) | 427.17(512.42,353.74) | -0.05 | 995.04(1105.33,885.96) | 943.09(1113.32,798.40) | -0.36(-0.55,-0.16) |
| Fiji | female | 3074.43(3603.03,2614.70) | 3283.42(3788.26,2840.65) | 0.07 | 918.71(1040.44,808.87) | 756.88(868.63,657.15) | -0.88(-0.99,-0.76) |
| Finland | female | 16050.71(18369.79,13821.86) | 19814.93(22523.30,17337.90) | 0.23 | 634.77(759.23,536.15) | 641.41(770.23,535.64) | 0.04(-0.08,0.16) |
| France | female | 288074.33(323843.81,258024.65) | 296130.63(329091.60,264717.22) | 0.03 | 866.94(1021.11,752.27) | 727.47(860.73,616.67) | -0.74(-0.81,-0.68) |
| Gabon | female | 4381.87(5248.98,3616.60) | 6542.80(7777.83,5429.18) | 0.49 | 871.76(992.34,756.27) | 785.09(907.73,673.12) | -0.49(-0.59,-0.38) |
| Georgia | female | 17170.06(19369.23,15189.55) | 14342.46(15930.97,12822.09) | -0.16 | 563.58(640.20,494.07) | 605.56(701.77,525.93) | 0.44(0.34,0.53) |
| Germany | female | 362267.46(398082.94,327953.31) | 373411.23(404306.52,344405.02) | 0.03 | 766.28(869.10,671.69) | 667.01(783.98,568.78) | -0.16(-0.38,0.06) |
| Ghana | female | 45107.88(55351.65,36897.92) | 97402.00(116673.12,82017.87) | 1.16 | 605.63(697.91,525.80) | 634.07(742.34,550.32) | 0.04(-0.05,0.13) |
| Greece | female | 41347.01(46706.99,36648.92) | 50023.23(54230.23,45736.61) | 0.21 | 704.34(824.96,609.15) | 715.93(843.66,615.97) | 0.21(0.14,0.28) |
| Greenland | female | 497.30(589.12,414.73) | 520.26(591.74,458.95) | 0.05 | 2195.34(2562.47,1857.06) | 1855.71(2138.56,1625.92) | -0.80(-0.91,-0.68) |
| Grenada | female | 617.14(762.11,492.43) | 652.77(775.40,554.58) | 0.06 | 1271.13(1543.86,1039.66) | 1400.21(1705.45,1170.34) | 0.23(0.18,0.28) |
| Guam | female | 646.81(781.60,531.04) | 883.55(1040.09,734.02) | 0.37 | 1147.49(1342.58,956.49) | 1025.96(1216.16,853.56) | -0.44(-0.52,-0.35) |
| Guatemala | female | 52194.80(63740.38,41420.22) | 77960.46(93608.34,63791.94) | 0.49 | 1130.64(1319.22,950.64) | 935.29(1101.17,785.62) | -0.84(-1.03,-0.65) |
| Guinea | female | 27145.74(33771.89,22227.23) | 52350.23(64927.48,43070.82) | 0.93 | 848.43(989.45,738.40) | 825.55(960.67,715.26) | -0.29(-0.39,-0.18) |
| Guinea-Bissau | female | 4212.90(5110.45,3446.65) | 7303.06(8865.38,6032.91) | 0.73 | 836.41(954.30,731.02) | 796.46(916.18,694.98) | -0.26(-0.37,-0.15) |
| Guyana | female | 5053.96(6167.60,4076.99) | 4504.72(5471.71,3742.78) | -0.11 | 1181.46(1408.25,980.91) | 1192.34(1445.10,989.04) | -0.15(-0.23,-0.08) |
| Haiti | female | 58705.18(70983.57,47216.31) | 92026.73(110734.08,75746.93) | 0.57 | 1508.08(1784.34,1256.69) | 1419.92(1676.70,1198.62) | -0.30(-0.35,-0.25) |
| Honduras | female | 35859.31(44223.11,28204.75) | 55796.38(66188.45,47015.10) | 0.56 | 1357.19(1586.61,1140.24) | 1227.40(1422.93,1056.09) | -0.54(-0.60,-0.49) |
| Hungary | female | 58664.50(66120.75,51989.94) | 68793.12(76612.35,61203.28) | 0.17 | 981.38(1122.21,855.87) | 1069.04(1223.66,935.70) | 0.32(0.25,0.38) |
| Iceland | female | 1406.05(1640.55,1223.29) | 1813.96(2052.58,1603.48) | 0.29 | 1108.42(1300.61,948.02) | 983.25(1164.71,833.94) | -0.47(-0.63,-0.31) |
| India | female | 3490269.35(3924101.52,3067970.37) | 6959728.16(7813317.93,6133479.01) | 0.99 | 1261.70(1413.21,1113.60) | 1147.08(1287.72,1012.89) | -0.25(-0.41,-0.09) |
| Indonesia | female | 648514.65(781105.98,550828.18) | 820902.81(948646.05,720419.15) | 0.27 | 778.34(901.16,683.28) | 698.95(812.47,610.17) | -0.39(-0.46,-0.33) |
| Iran (Islamic Republic of) | female | 255143.84(314014.00,208070.15) | 355020.08(411175.80,309913.14) | 0.39 | 924.30(1073.87,805.01) | 915.39(1063.45,797.94) | -0.19(-0.27,-0.11) |
| Iraq | female | 81464.75(99645.63,66534.74) | 177463.14(216177.52,148254.30) | 1.18 | 941.99(1087.84,820.20) | 925.33(1092.98,794.49) | -0.06(-0.12,0.00) |
| Ireland | female | 19412.84(21972.93,17129.85) | 23563.96(26202.01,21190.55) | 0.21 | 1026.45(1163.01,895.84) | 859.67(995.97,744.38) | -0.66(-0.72,-0.60) |
| Israel | female | 17696.51(20859.61,15085.67) | 31082.00(35702.51,26704.24) | 0.76 | 698.68(816.44,599.38) | 613.68(719.24,516.30) | -0.51(-0.61,-0.41) |
| Italy | female | 250513.64(282009.26,221204.60) | 271220.72(297854.85,243754.05) | 0.08 | 718.08(834.00,621.04) | 689.68(829.73,562.89) | -0.31(-0.46,-0.17) |
| Jamaica | female | 16462.55(20305.64,13118.46) | 14373.66(17418.68,11957.89) | -0.13 | 1252.09(1513.93,1018.71) | 1133.38(1410.51,919.21) | -0.50(-0.59,-0.42) |
| Japan | female | 909110.50(1040194.55,792578.18) | 860069.11(986923.96,744787.19) | -0.05 | 1335.39(1577.86,1146.65) | 967.13(1182.34,808.46) | -1.06(-1.39,-0.74) |
| Jordan | female | 17620.22(21998.83,14211.32) | 56748.99(67105.19,47498.79) | 2.22 | 1127.92(1310.95,968.20) | 1192.32(1382.16,1018.06) | 0.19(0.15,0.23) |
| Kazakhstan | female | 53617.25(60963.62,47223.36) | 64976.03(74122.11,57057.06) | 0.21 | 658.64(744.86,584.25) | 665.93(758.60,583.33) | 0.17(-0.05,0.38) |
| Kenya | female | 96280.06(121432.36,75920.74) | 172842.31(211188.54,141611.53) | 0.80 | 820.38(957.74,712.73) | 732.27(857.99,630.57) | -0.54(-0.64,-0.45) |
| Kiribati | female | 434.46(505.18,373.13) | 567.66(645.40,494.48) | 0.31 | 1153.08(1308.85,1019.40) | 986.45(1103.66,874.27) | -0.60(-0.63,-0.57) |
| Kuwait | female | 6793.37(8174.42,5610.50) | 19159.50(22523.28,15990.62) | 1.82 | 1076.34(1244.51,930.42) | 1154.19(1365.40,963.21) | 0.31(0.26,0.37) |
| Kyrgyzstan | female | 19430.51(22888.40,16927.16) | 23904.53(28560.97,20209.68) | 0.23 | 913.94(1038.51,816.44) | 760.95(888.09,655.14) | -0.73(-0.89,-0.58) |
| Lao People's Democratic Republic | female | 11709.94(13876.11,9915.96) | 18847.13(21443.84,16661.78) | 0.61 | 602.38(675.99,540.88) | 618.66(691.38,554.55) | 0.01(-0.06,0.09) |
| Latvia | female | 15015.00(17007.76,13131.56) | 10725.45(12394.30,9316.69) | -0.29 | 956.53(1094.80,833.83) | 897.85(1063.87,754.64) | 0.15(0.02,0.29) |
| Lebanon | female | 16287.67(19704.37,13826.22) | 31597.77(36036.82,27762.63) | 0.94 | 1023.82(1196.39,890.88) | 1189.99(1373.07,1038.50) | 0.52(0.49,0.55) |
| Lesotho | female | 5201.45(6004.80,4552.31) | 6653.21(7465.68,5951.92) | 0.28 | 657.82(731.28,590.45) | 724.30(803.42,653.95) | 0.34(0.24,0.43) |
| Liberia | female | 8285.86(10371.58,6631.65) | 16245.56(19725.71,13425.38) | 0.96 | 767.00(903.07,654.58) | 715.95(833.08,623.86) | -0.37(-0.44,-0.30) |
| Libya | female | 18842.26(23184.09,15274.39) | 30408.64(34795.35,26878.65) | 0.61 | 981.06(1127.51,857.71) | 1038.57(1200.14,909.48) | 0.23(0.17,0.29) |
| Lithuania | female | 17209.59(19560.36,15099.57) | 13194.55(15122.55,11563.28) | -0.23 | 829.05(949.16,722.16) | 775.66(918.21,645.39) | -0.14(-0.21,-0.07) |
| Luxembourg | female | 1646.53(1824.73,1479.08) | 2682.20(2951.18,2429.27) | 0.63 | 780.03(900.74,671.76) | 771.43(893.95,661.43) | -0.15(-0.22,-0.09) |
| North Macedonia | female | 13020.96(15140.42,11001.39) | 14086.12(15973.86,12519.22) | 0.08 | 1288.85(1494.01,1099.03) | 1174.23(1356.05,1030.12) | -0.52(-0.65,-0.40) |
| Madagascar | female | 98075.11(122237.62,78211.50) | 162682.83(194915.11,134508.02) | 0.66 | 1410.43(1668.72,1197.66) | 1194.82(1375.39,1029.53) | -0.78(-0.85,-0.71) |
| Malawi | female | 55707.90(69945.40,43625.34) | 93327.06(116746.42,73831.91) | 0.68 | 1002.32(1189.74,838.31) | 940.62(1121.01,790.34) | -0.31(-0.37,-0.25) |
| Malaysia | female | 56156.71(66075.85,47980.99) | 99578.72(116155.38,86053.25) | 0.77 | 722.66(821.11,643.38) | 720.79(846.31,620.42) | -0.04(-0.11,0.03) |
| Maldives | female | 1219.81(1514.00,989.33) | 1704.15(1965.97,1478.62) | 0.40 | 1272.89(1438.20,1127.09) | 1046.39(1187.99,912.71) | -0.85(-0.92,-0.78) |
| Mali | female | 26790.58(32589.47,22758.29) | 70036.35(86133.82,58032.82) | 1.61 | 653.88(743.53,584.13) | 695.68(791.75,620.75) | 0.18(0.11,0.25) |
| Malta | female | 1619.30(1846.24,1410.25) | 1923.09(2176.64,1708.44) | 0.19 | 843.42(977.50,731.07) | 774.89(927.36,651.86) | -0.39(-0.43,-0.34) |
| Marshall Islands | female | 190.80(230.09,158.72) | 222.24(269.12,183.04) | 0.16 | 943.94(1059.18,832.84) | 893.63(1059.43,752.41) | -0.36(-0.54,-0.18) |
| Mauritania | female | 10372.19(12798.59,8417.11) | 18924.14(23297.05,15491.70) | 0.82 | 1010.77(1174.50,872.93) | 961.06(1130.01,831.51) | -0.33(-0.43,-0.23) |
| Mauritius | female | 4414.83(5132.95,3829.14) | 6038.29(7013.47,5188.15) | 0.37 | 906.84(1040.72,792.11) | 853.57(994.97,727.42) | -0.43(-0.48,-0.37) |
| Mexico | female | 457855.85(559082.22,377905.69) | 679792.23(800438.75,580415.47) | 0.48 | 1226.08(1421.61,1050.16) | 1123.26(1329.76,955.39) | -0.51(-0.68,-0.34) |
| Republic of Moldova | female | 22302.28(25602.79,19529.31) | 17423.35(20414.93,14965.00) | -0.22 | 934.25(1078.41,810.02) | 829.68(988.19,695.48) | -0.50(-0.61,-0.38) |
| Mongolia | female | 6857.49(8166.44,5732.99) | 10645.40(12535.84,9116.61) | 0.55 | 753.72(852.62,665.70) | 662.69(773.41,572.91) | -0.53(-0.62,-0.45) |
| Montenegro | female | 2723.59(3195.02,2323.44) | 3267.08(3802.40,2793.91) | 0.20 | 858.09(1010.89,725.89) | 946.77(1117.20,806.46) | 0.38(0.27,0.49) |
| Morocco | female | 90450.85(107243.27,76153.48) | 149545.97(171892.94,131034.55) | 0.65 | 755.57(863.19,656.09) | 877.16(1009.53,768.57) | 0.43(0.35,0.52) |
| Mozambique | female | 72009.26(89151.18,57846.93) | 146505.84(181473.51,116660.43) | 1.03 | 910.82(1073.51,774.20) | 870.80(1019.39,736.92) | -0.30(-0.37,-0.22) |
| Myanmar | female | 117855.29(133695.67,104751.17) | 205722.03(225013.52,186340.91) | 0.75 | 715.79(779.48,658.96) | 776.22(850.00,704.42) | 0.24(0.22,0.26) |
| Namibia | female | 5221.03(6168.16,4512.03) | 8671.11(10157.41,7468.06) | 0.66 | 829.71(930.67,744.84) | 782.52(892.87,689.21) | -0.36(-0.44,-0.27) |
| Nepal | female | 54419.08(59759.90,49685.29) | 126523.92(140175.44,114129.47) | 1.32 | 928.50(1013.39,849.75) | 1004.70(1107.85,906.23) | 0.00(-0.09,0.08) |
| Netherlands | female | 52512.53(57795.14,47884.84) | 80497.50(86370.14,74821.82) | 0.53 | 628.40(712.93,554.59) | 702.79(789.80,625.47) | 0.30(0.23,0.37) |
| New Zealand | female | 25061.15(28745.41,21786.55) | 31775.40(36015.00,28079.54) | 0.27 | 1441.09(1702.09,1233.20) | 1174.03(1389.37,996.21) | -0.79(-0.91,-0.67) |
| Nicaragua | female | 29889.71(37697.16,23449.72) | 32721.34(39620.39,27105.46) | 0.09 | 1249.49(1494.75,1037.20) | 1063.15(1264.24,898.50) | -0.63(-0.79,-0.47) |
| Niger | female | 34547.79(43422.22,27398.50) | 87919.44(111513.11,70051.21) | 1.54 | 839.32(967.44,731.14) | 750.19(867.21,656.37) | -0.52(-0.61,-0.44) |
| Nigeria | female | 455835.60(568272.88,371448.43) | 923462.23(1154843.33,755137.47) | 1.03 | 1016.95(1192.12,888.00) | 844.90(993.16,735.63) | -0.78(-0.88,-0.68) |
| Democratic People's Republic of Korea | female | 99791.84(117861.13,86196.22) | 147553.02(160366.60,135976.71) | 0.48 | 945.26(1096.96,829.54) | 978.33(1097.24,877.61) | -0.14(-0.24,-0.04) |
| Northern Mariana Islands | female | 197.04(236.57,163.83) | 216.65(253.05,179.74) | 0.10 | 1218.45(1426.59,1044.82) | 1081.90(1278.37,913.31) | -0.39(-0.47,-0.31) |
| Norway | female | 26709.04(30082.87,23417.03) | 32152.46(35661.11,28918.04) | 0.20 | 1169.59(1364.85,1002.46) | 1071.22(1260.57,915.04) | -0.35(-0.42,-0.28) |
| Oman | female | 6237.12(7811.28,5044.63) | 15111.00(18290.84,12672.82) | 1.42 | 765.39(902.71,651.09) | 1030.68(1207.97,881.40) | 1.16(1.03,1.29) |
| Pakistan | female | 300579.09(350906.53,262477.38) | 573847.01(663314.90,505178.71) | 0.91 | 769.11(851.52,688.69) | 717.09(792.10,647.41) | -0.18(-0.24,-0.12) |
| Palestine | female | 10123.86(12399.27,8343.46) | 24899.58(29814.66,20692.81) | 1.46 | 1143.02(1323.60,977.97) | 1252.11(1454.25,1063.53) | 0.32(0.26,0.39) |
| Panama | female | 14567.35(17546.40,11935.50) | 26150.45(30720.06,22028.58) | 0.80 | 1219.76(1434.98,1033.23) | 1268.30(1502.18,1063.34) | 0.03(-0.03,0.10) |
| Papua New Guinea | female | 24868.41(28485.43,21550.18) | 54126.66(61094.71,48066.88) | 1.18 | 1512.85(1656.19,1361.46) | 1412.44(1550.77,1288.37) | -0.30(-0.35,-0.24) |
| Paraguay | female | 28143.04(36881.87,21374.27) | 45145.11(57820.82,35897.37) | 0.60 | 1175.39(1472.15,940.47) | 1364.98(1744.31,1086.47) | 0.55(0.45,0.64) |
| Peru | female | 182292.95(224257.62,144722.59) | 270486.95(324844.18,224144.71) | 0.48 | 1648.58(1940.82,1364.53) | 1665.90(2010.59,1377.65) | -0.19(-0.31,-0.06) |
| Philippines | female | 371114.24(482226.39,293316.66) | 502712.68(623044.84,416890.55) | 0.35 | 1088.34(1339.61,905.97) | 912.59(1109.90,771.83) | -0.68(-0.76,-0.60) |
| Poland | female | 314347.28(357348.23,275876.22) | 292003.80(326299.71,260909.03) | -0.07 | 1525.07(1758.47,1328.49) | 1302.91(1537.98,1121.13) | -0.74(-0.81,-0.68) |
| Portugal | female | 53425.19(61735.69,46165.43) | 59969.71(65640.69,54600.11) | 0.12 | 954.88(1123.71,819.80) | 880.26(1036.23,739.73) | -0.29(-0.48,-0.10) |
| Puerto Rico | female | 30644.40(36315.15,25339.60) | 30623.71(34889.99,26938.85) | 0.00 | 1691.16(2009.39,1392.47) | 1690.43(2036.97,1422.75) | -0.05(-0.18,0.07) |
| Qatar | female | 1434.32(1795.16,1146.97) | 6739.75(8104.58,5668.96) | 3.70 | 1068.43(1254.65,924.50) | 1151.86(1337.06,997.57) | 0.24(0.20,0.29) |
| Romania | female | 164915.42(189093.68,144355.61) | 137543.02(155704.28,121277.69) | -0.17 | 1254.29(1443.58,1097.83) | 1136.61(1327.22,988.58) | -0.41(-0.47,-0.34) |
| Russian Federation | female | 926404.59(1059960.72,814182.88) | 694118.07(804681.49,603558.57) | -0.25 | 1076.07(1252.57,927.92) | 826.48(981.68,694.64) | -1.16(-1.34,-0.98) |
| Rwanda | female | 71411.87(89486.61,57382.28) | 96175.33(118277.47,78282.98) | 0.35 | 1623.89(1926.05,1372.80) | 1437.33(1707.00,1206.62) | -0.47(-0.51,-0.44) |
| Saint Lucia | female | 926.46(1126.92,748.40) | 993.38(1148.63,851.49) | 0.07 | 1208.87(1435.85,1006.54) | 1272.47(1526.70,1051.03) | 0.09(0.05,0.14) |
| Saint Vincent and the Grenadines | female | 665.68(828.17,530.78) | 562.73(678.87,467.10) | -0.15 | 1070.89(1306.11,878.65) | 1125.02(1381.18,917.46) | 0.16(0.10,0.21) |
| Samoa | female | 661.48(781.25,557.19) | 848.69(1014.67,708.23) | 0.28 | 926.60(1053.23,814.23) | 888.44(1040.27,761.88) | -0.20(-0.31,-0.10) |
| Sao Tome and Principe | female | 663.18(798.26,550.29) | 967.82(1127.35,836.55) | 0.46 | 1081.29(1225.80,949.61) | 1062.92(1199.19,952.03) | -0.25(-0.32,-0.18) |
| Saudi Arabia | female | 50042.79(60637.75,42105.52) | 141260.08(162970.12,122720.22) | 1.82 | 961.66(1099.69,825.08) | 1300.09(1504.24,1109.68) | 1.09(1.03,1.16) |
| Senegal | female | 28179.88(34902.86,22973.58) | 52944.22(64737.05,43633.16) | 0.88 | 721.93(833.19,631.76) | 728.58(849.57,631.48) | -0.01(-0.13,0.12) |
| Serbia | female | 43340.82(49341.61,38252.33) | 51599.65(57268.23,46370.97) | 0.19 | 841.68(964.13,738.23) | 943.67(1065.06,831.45) | 0.60(0.54,0.67) |
| Seychelles | female | 197.80(236.99,167.10) | 278.34(324.88,241.72) | 0.41 | 536.97(633.29,463.88) | 589.89(709.84,497.80) | 0.28(0.21,0.34) |
| Sierra Leone | female | 14347.74(17514.50,11686.00) | 30729.49(37439.39,25497.53) | 1.14 | 755.42(872.62,652.87) | 776.31(895.99,680.94) | 0.07(-0.03,0.17) |
| Singapore | female | 15254.45(17293.61,13473.34) | 23980.86(27578.47,20568.70) | 0.57 | 1110.65(1257.75,980.22) | 858.35(1017.51,730.08) | -0.61(-0.85,-0.37) |
| Slovakia | female | 23003.09(26335.03,20008.12) | 30711.92(34963.13,26865.32) | 0.34 | 820.78(948.58,709.81) | 928.33(1069.20,801.24) | 0.47(0.40,0.54) |
| Slovenia | female | 12703.78(14532.71,11095.69) | 16116.78(18225.34,14276.95) | 0.27 | 1163.71(1349.50,1011.13) | 1198.74(1402.02,1039.87) | 0.23(0.15,0.31) |
| Solomon Islands | female | 1400.28(1678.49,1146.02) | 2657.86(3253.50,2200.71) | 0.90 | 959.76(1088.54,842.28) | 930.69(1086.93,808.96) | -0.19(-0.33,-0.05) |
| Somalia | female | 40730.90(49529.54,32394.19) | 101061.05(123768.18,82961.11) | 1.48 | 1059.85(1223.60,900.17) | 934.16(1088.59,811.49) | -0.49(-0.55,-0.43) |
| South Africa | female | 217966.69(265534.32,173238.58) | 292291.85(347020.96,243157.06) | 0.34 | 1244.73(1479.18,1023.61) | 1090.46(1296.62,902.89) | -0.95(-1.38,-0.52) |
| Republic of Korea | female | 142644.76(168446.49,121787.81) | 201537.42(226001.58,181809.90) | 0.41 | 746.18(874.25,640.20) | 707.92(855.68,602.15) | -0.29(-0.38,-0.20) |
| South Sudan | female | 30026.96(37485.23,24211.90) | 43994.15(54428.20,35524.41) | 0.47 | 1002.66(1173.26,858.26) | 904.80(1064.52,772.48) | -0.45(-0.53,-0.36) |
| Spain | female | 149310.35(166889.32,133423.09) | 212161.45(231345.21,194138.76) | 0.42 | 655.98(758.77,567.62) | 679.78(797.69,583.61) | 0.23(0.12,0.33) |
| Sri Lanka | female | 52825.20(61707.13,45616.27) | 91161.04(102132.36,81237.35) | 0.73 | 707.81(802.55,626.55) | 772.79(892.63,680.34) | 0.20(0.17,0.23) |
| Sudan | female | 93724.05(115548.43,76206.41) | 188802.12(231114.55,154205.43) | 1.01 | 990.47(1151.31,851.22) | 1062.71(1234.21,919.53) | 0.10(0.03,0.17) |
| Suriname | female | 2295.05(2840.07,1854.96) | 3160.40(3821.40,2607.35) | 0.38 | 1122.76(1360.45,924.41) | 1154.29(1415.02,938.84) | 0.04(-0.04,0.12) |
| Eswatini | female | 3935.38(4804.72,3222.13) | 5071.77(5961.13,4383.17) | 0.29 | 1055.20(1201.15,931.16) | 982.22(1118.56,869.37) | -0.42(-0.49,-0.36) |
| Sweden | female | 53864.46(61531.64,46547.48) | 57479.32(64413.04,51401.94) | 0.07 | 1129.35(1341.38,959.64) | 950.11(1139.31,806.15) | -0.46(-0.71,-0.21) |
| Switzerland | female | 28150.43(31582.00,25030.35) | 38038.48(42236.43,34246.46) | 0.35 | 762.72(891.50,650.12) | 753.09(901.79,637.22) | -0.13(-0.18,-0.08) |
| Syrian Arab Republic | female | 59106.31(72592.13,48489.65) | 74565.68(85925.19,64590.22) | 0.26 | 991.18(1137.26,867.31) | 1079.09(1241.67,940.12) | 0.50(0.35,0.66) |
| Taiwan (Province of China) | female | 64341.49(76417.54,55124.90) | 86174.97(97672.23,75608.29) | 0.34 | 743.90(875.96,642.21) | 707.74(856.56,590.35) | -0.56(-0.73,-0.40) |
| Tajikistan | female | 21254.65(25153.84,17886.91) | 32752.53(38857.41,27598.92) | 0.54 | 968.01(1113.30,839.09) | 843.19(965.29,728.87) | -0.54(-0.61,-0.47) |
| United Republic of Tanzania | female | 166540.54(211493.31,130246.30) | 360468.52(448398.17,284875.85) | 1.16 | 1088.61(1301.53,913.11) | 1116.77(1324.02,929.57) | 0.14(0.04,0.23) |
| Thailand | female | 193330.03(225232.20,167371.20) | 235397.13(264549.62,209288.59) | 0.22 | 768.64(884.11,679.11) | 678.21(817.92,572.45) | -0.80(-0.95,-0.66) |
| Bahamas | female | 1474.43(1840.68,1210.68) | 2123.15(2514.57,1773.85) | 0.44 | 1147.05(1402.92,956.79) | 1245.62(1524.85,1013.45) | 0.29(0.26,0.33) |
| Gambia | female | 3692.35(4565.80,3002.37) | 8210.00(10024.77,6797.74) | 1.22 | 786.42(901.90,690.95) | 777.38(893.59,681.71) | -0.11(-0.22,0.00) |
| Timor-Leste | female | 3016.65(3735.31,2443.86) | 4658.20(5563.02,3959.15) | 0.54 | 763.46(880.25,661.53) | 747.14(858.33,659.92) | -0.17(-0.27,-0.06) |
| Togo | female | 16833.69(21285.34,13479.84) | 33387.83(41345.63,27295.06) | 0.98 | 902.67(1044.53,778.54) | 853.12(1009.16,730.33) | -0.28(-0.37,-0.18) |
| Tonga | female | 461.02(566.72,377.99) | 477.33(583.22,392.23) | 0.04 | 1006.71(1171.88,871.52) | 934.11(1129.30,783.82) | -0.37(-0.51,-0.24) |
| Trinidad and Tobago | female | 5936.12(7138.78,4901.91) | 7185.54(8630.92,5949.11) | 0.21 | 968.23(1146.29,818.30) | 1143.92(1436.01,915.88) | 0.38(0.13,0.63) |
| Tunisia | female | 33460.20(39955.45,28434.90) | 56460.85(64805.30,49647.63) | 0.69 | 864.23(997.48,758.87) | 974.43(1132.99,845.35) | 0.36(0.30,0.42) |
| Turkey | female | 344168.92(408179.26,295619.99) | 547221.00(610666.25,491239.41) | 0.59 | 1250.03(1449.57,1106.11) | 1341.15(1519.88,1187.41) | 0.10(-0.07,0.26) |
| Turkmenistan | female | 13354.67(16102.72,11412.85) | 15306.97(18303.84,12726.42) | 0.15 | 784.18(892.52,695.75) | 622.48(739.97,520.80) | -0.96(-1.24,-0.68) |
| Uganda | female | 94981.69(118204.16,75252.87) | 198428.57(249015.75,156414.01) | 1.09 | 963.50(1122.12,823.05) | 898.81(1064.41,765.25) | -0.38(-0.46,-0.30) |
| Ukraine | female | 415147.24(468394.62,364053.45) | 206622.32(238939.46,178089.78) | -0.50 | 1272.44(1474.20,1110.10) | 854.04(1032.67,699.48) | -1.70(-1.91,-1.49) |
| United Arab Emirates | female | 8012.80(9887.43,6555.81) | 32734.24(38539.07,28087.17) | 3.09 | 1394.24(1605.94,1194.48) | 1556.83(1787.83,1363.34) | 0.80(0.43,1.16) |
| United Kingdom | female | 400747.56(455825.18,349775.54) | 434751.82(485108.08,390374.21) | 0.08 | 1348.96(1599.42,1134.08) | 1171.34(1392.03,990.61) | -0.64(-0.75,-0.52) |
| United States of America | female | 2971064.95(3424508.38,2585465.99) | 4981312.98(5579734.92,4409511.86) | 0.68 | 2249.99(2675.08,1932.23) | 2622.86(2996.68,2292.15) | 1.64(1.10,2.19) |
| Uruguay | female | 15905.16(18348.53,13782.48) | 20026.21(22628.36,17750.20) | 0.26 | 950.79(1110.03,813.91) | 997.19(1177.98,851.94) | 0.27(0.23,0.31) |
| Uzbekistan | female | 101495.72(120754.69,86035.39) | 129145.47(152167.70,110104.83) | 0.27 | 1118.71(1268.78,975.45) | 842.22(975.26,727.47) | -1.33(-1.47,-1.19) |
| Vanuatu | female | 613.54(730.37,514.06) | 1222.32(1478.02,994.44) | 0.99 | 967.35(1082.98,859.77) | 944.39(1107.43,793.25) | -0.34(-0.61,-0.07) |
| Venezuela (Bolivarian Republic of) | female | 104296.92(129510.84,83510.26) | 147124.37(172176.71,125600.49) | 0.41 | 1077.96(1287.31,894.06) | 1066.03(1267.67,892.28) | -0.13(-0.20,-0.07) |
| Viet nam | female | 191127.31(232727.29,159030.84) | 324950.24(367954.95,287791.58) | 0.70 | 572.87(668.41,497.15) | 695.88(808.17,606.93) | 0.58(0.46,0.71) |
| Virginia | female | 73353.16(84800.42,63643.75) | 136747.15(154815.33,121262.00) | 0.86 | 2313.81(2737.66,1975.14) | 2743.11(3137.60,2397.26) | 1.78(1.23,2.33) |
| Yemen | female | 70587.14(87318.43,57887.89) | 143228.23(173232.62,118769.25) | 1.03 | 1066.91(1213.28,942.49) | 1016.96(1170.40,889.78) | -0.20(-0.28,-0.13) |
| Zambia | female | 27680.24(33803.02,22570.08) | 60965.31(75777.38,49013.85) | 1.20 | 678.97(776.08,596.20) | 678.98(796.00,577.30) | -0.06(-0.25,0.14) |
| Zimbabwe | female | 30860.51(37193.22,25927.11) | 49609.49(57384.54,42855.63) | 0.61 | 706.58(803.84,626.62) | 739.17(831.98,659.08) | 0.13(0.00,0.27) |
| Monaco | female | 134.67(148.39,122.50) | 181.02(198.84,164.48) | 0.34 | 696.40(839.60,582.94) | 730.78(865.03,612.61) | 0.19(0.17,0.22) |
| San Marino | female | 87.53(100.86,77.16) | 144.77(161.15,129.87) | 0.65 | 705.47(843.72,595.13) | 729.72(861.41,614.07) | 0.15(0.13,0.17) |
| Saint Kitts and Nevis | female | 273.32(331.90,223.44) | 338.63(398.71,287.42) | 0.24 | 1230.23(1474.14,1020.97) | 1284.44(1569.47,1062.23) | 0.07(0.02,0.12) |
| Cook Islands | female | 95.04(113.82,79.24) | 110.22(124.13,94.73) | 0.16 | 1129.37(1310.13,967.87) | 1114.17(1290.96,954.59) | 0.05(-0.05,0.15) |
| Nauru | female | 44.36(54.22,36.32) | 46.90(57.99,38.24) | 0.06 | 1040.75(1185.94,916.08) | 1073.23(1252.35,911.38) | -0.11(-0.37,0.15) |
| Niue | female | 13.46(15.77,11.43) | 10.13(11.50,8.73) | -0.25 | 1097.38(1271.42,938.03) | 1096.29(1263.97,934.44) | 0.33(0.15,0.50) |
| Palau | female | 75.86(89.73,63.32) | 93.93(106.99,79.65) | 0.24 | 1135.84(1312.00,961.20) | 1103.27(1281.41,937.04) | -0.09(-0.18,0.00) |
| Tokelau | female | 9.33(11.24,7.70) | 7.62(8.99,6.45) | -0.18 | 1041.24(1215.01,883.76) | 1085.07(1259.52,922.86) | 0.31(0.13,0.49) |
| Tuvalu | female | 45.72(52.28,40.15) | 58.48(67.80,49.35) | 0.28 | 990.62(1110.97,884.01) | 1059.55(1220.91,899.43) | 0.00(-0.24,0.24) |

**Supplementary Table 4. The deaths and age-standardized death rate of chronic obstructive pulmonary disease in 1990 and 2019, and its temporal trends from 1990 to 2019.**

| **Nation** | **Sex** | **Death Cases No. (95% UI)** | | **Change in absolute number (%)** | **ASDR per 100,000 No.(95% UI)** | | **1990-2019 EAPC No. (95%CI)** |
| --- | --- | --- | --- | --- | --- | --- | --- |
|  |  | **1990** | **2019** |  | **1990** | **2019** |  |
| Afghanistan | both | 5945.35(7128.01,4808.86) | 7082.19(8699.00,5443.95) | 0.19 | 94.84(112.96,78.02) | 67.82(81.33,51.99) | -1.34(-1.60,-1.07) |
| Albania | both | 1006.04(1096.32,695.08) | 814.64(1053.12,607.71) | -0.19 | 59.10(64.70,39.61) | 19.54(25.16,14.61) | -4.17(-4.65,-3.69) |
| Algeria | both | 4410.01(5596.24,3377.91) | 7527.83(9346.53,6030.39) | 0.71 | 53.82(66.94,41.75) | 30.36(37.49,24.50) | -1.94(-2.03,-1.85) |
| American Samoa | both | 16.81(18.99,14.52) | 22.95(27.78,19.31) | 0.37 | 96.52(109.26,82.70) | 57.84(69.95,48.68) | -1.95(-2.04,-1.87) |
| Andorra | both | 15.53(20.54,12.00) | 39.42(48.69,30.83) | 1.54 | 35.77(46.21,27.88) | 25.66(32.13,20.11) | -1.18(-1.34,-1.03) |
| Angola | both | 2793.92(3626.10,2084.38) | 3933.85(5045.51,2885.66) | 0.41 | 82.55(118.87,59.96) | 46.92(62.07,35.19) | -2.17(-2.34,-2.00) |
| Antigua and Barbuda | both | 6.69(7.54,5.93) | 10.55(12.65,8.64) | 0.58 | 11.61(13.08,10.33) | 11.93(14.20,9.71) | 0.01(-0.20,0.22) |
| Argentina | both | 9893.85(11384.68,9153.62) | 19348.12(21811.34,16774.76) | 0.96 | 33.02(38.60,30.24) | 34.85(39.29,30.28) | -0.04(-0.29,0.20) |
| Armenia | both | 1077.93(1154.04,901.92) | 1323.02(1581.22,1016.27) | 0.23 | 48.39(52.16,39.39) | 33.77(40.44,25.81) | -1.38(-1.60,-1.17) |
| Australia | both | 6965.95(7402.21,6488.04) | 11202.86(12660.90,9284.03) | 0.61 | 35.97(38.30,33.28) | 24.18(27.12,20.30) | -1.56(-1.80,-1.32) |
| Austria | both | 2524.09(2897.93,2338.55) | 3405.86(3824.66,2914.12) | 0.35 | 20.33(23.30,18.81) | 16.92(18.79,14.47) | -0.47(-0.62,-0.32) |
| Azerbaijan | both | 1934.10(2169.39,1728.30) | 2005.94(3035.50,1556.46) | 0.04 | 41.91(47.59,37.35) | 31.94(49.48,23.85) | -1.47(-1.95,-0.99) |
| Bahrain | both | 85.78(98.36,74.26) | 161.18(206.87,128.98) | 0.88 | 78.35(88.92,67.14) | 37.15(45.41,30.47) | -2.83(-3.38,-2.27) |
| Bangladesh | both | 61136.68(78435.75,52166.05) | 70892.81(117827.71,53727.05) | 0.16 | 147.55(198.33,125.64) | 62.02(106.80,47.21) | -3.11(-3.45,-2.76) |
| Barbados | both | 39.81(45.12,35.01) | 62.81(75.73,50.31) | 0.58 | 12.90(14.62,11.42) | 12.96(15.57,10.40) | -0.26(-0.43,-0.08) |
| Belarus | both | 6648.08(7315.68,4906.28) | 2437.92(3910.01,1838.32) | -0.63 | 53.71(59.38,39.04) | 14.98(24.05,11.29) | -5.61(-6.13,-5.09) |
| Belgium | both | 6493.88(7016.74,6017.19) | 7400.33(8256.18,6282.30) | 0.14 | 41.08(44.29,38.01) | 27.63(30.62,23.71) | -1.77(-1.91,-1.63) |
| Belize | both | 22.02(24.56,19.43) | 73.60(86.62,61.54) | 2.34 | 22.42(25.37,19.47) | 28.82(33.98,24.06) | 0.58(0.07,1.09) |
| Benin | both | 1340.34(1588.99,1082.14) | 1887.95(2448.73,1457.65) | 0.41 | 69.20(82.02,55.90) | 42.45(53.78,34.03) | -1.47(-1.63,-1.30) |
| Bermuda | both | 10.94(12.30,9.79) | 18.13(21.94,14.87) | 0.66 | 19.04(21.48,16.91) | 13.21(15.97,10.89) | -1.33(-1.42,-1.23) |
| Bhutan | both | 299.65(394.03,217.85) | 570.96(781.29,434.49) | 0.91 | 162.86(216.77,120.78) | 123.17(164.64,94.18) | -0.99(-1.01,-0.97) |
| Bolivia (Plurinational State of) | both | 1855.87(2221.37,1475.28) | 3577.63(4444.95,2812.37) | 0.93 | 62.07(73.06,51.29) | 51.07(63.07,40.15) | -0.61(-0.67,-0.55) |
| Bosnia and Herzegovina | both | 1162.31(1244.23,997.47) | 1204.21(1486.99,952.98) | 0.04 | 36.31(39.07,30.79) | 20.97(25.73,16.59) | -2.17(-2.37,-1.98) |
| Botswana | both | 463.35(649.55,334.75) | 649.04(846.65,480.67) | 0.40 | 95.12(132.98,68.85) | 58.45(74.00,43.86) | -2.07(-2.41,-1.73) |
| Brazil | both | 40253.50(42288.01,36784.22) | 76550.65(85615.68,68782.25) | 0.90 | 56.04(59.18,50.36) | 34.38(38.59,30.76) | -2.06(-2.24,-1.88) |
| Brunei Darussalam | both | 72.51(82.04,52.71) | 92.98(107.06,81.29) | 0.28 | 128.47(147.90,85.53) | 64.50(72.90,54.57) | -2.51(-2.70,-2.32) |
| Bulgaria | both | 3785.93(4056.36,3436.44) | 3035.83(3809.41,2404.86) | -0.20 | 35.16(37.74,31.47) | 20.51(25.64,16.24) | -2.34(-2.58,-2.11) |
| Burkina Faso | both | 1658.08(1988.73,1350.24) | 2713.92(3291.39,2231.81) | 0.64 | 41.99(50.35,34.73) | 31.75(38.01,26.44) | -1.03(-1.18,-0.87) |
| Burundi | both | 2249.11(2805.99,1632.56) | 2399.21(3214.50,1769.40) | 0.07 | 94.50(116.48,69.75) | 61.07(79.19,45.92) | -1.74(-1.83,-1.65) |
| Cambodia | both | 2737.60(3164.49,2257.25) | 4959.28(5791.38,3936.04) | 0.81 | 73.54(85.76,59.91) | 53.76(62.11,42.74) | -1.14(-1.18,-1.11) |
| Cameroon | both | 2629.50(3138.81,2115.86) | 4165.72(5526.26,3053.28) | 0.58 | 67.97(80.56,55.12) | 40.02(51.76,30.06) | -1.64(-1.73,-1.56) |
| Canada | both | 9319.26(10076.41,8589.83) | 18385.19(20998.56,14506.24) | 0.97 | 28.77(31.03,26.44) | 24.22(27.56,19.52) | -0.96(-1.10,-0.82) |
| Cabo Verde | both | 133.46(151.68,86.16) | 106.71(144.84,86.22) | -0.20 | 55.50(63.02,36.06) | 25.59(34.64,20.74) | -2.96(-3.62,-2.29) |
| Central African Republic | both | 1074.31(1444.28,765.88) | 1578.55(2341.66,1054.29) | 0.47 | 109.22(161.94,73.88) | 91.58(146.99,58.78) | -0.60(-0.65,-0.55) |
| Chad | both | 1886.30(2492.66,1454.24) | 2760.97(3485.92,2143.10) | 0.46 | 69.78(92.40,53.61) | 52.82(65.42,40.86) | -0.78(-0.86,-0.71) |
| Chile | both | 2780.63(2956.01,2444.13) | 6389.12(7227.39,4942.51) | 1.30 | 32.28(34.53,28.09) | 26.87(30.37,20.81) | -0.24(-0.50,0.02) |
| China | both | 1301224.12(1458960.69,955215.85) | 1085272.61(1320157.12,929796.86) | -0.17 | 226.43(251.43,170.16) | 67.98(83.43,57.80) | -4.51(-4.72,-4.29) |
| Colombia | both | 6210.74(6637.58,5413.61) | 17772.26(22724.21,12951.74) | 1.86 | 41.82(44.92,36.49) | 31.90(40.81,23.40) | -1.27(-1.47,-1.07) |
| Comoros | both | 128.51(167.77,80.42) | 167.14(207.85,131.34) | 0.30 | 62.14(79.47,41.96) | 38.28(47.36,30.52) | -1.83(-1.98,-1.69) |
| Congo | both | 789.19(1089.40,568.41) | 996.94(1305.13,723.47) | 0.26 | 90.30(132.36,63.54) | 51.39(68.20,37.85) | -2.09(-2.19,-1.98) |
| Costa Rica | both | 476.75(525.86,419.56) | 1424.60(1793.72,1085.69) | 1.99 | 29.57(32.79,25.90) | 27.59(34.87,21.10) | -0.95(-1.49,-0.42) |
| Côte d'Ivoire | both | 2305.47(2763.87,1790.15) | 3642.29(4612.47,2763.26) | 0.58 | 67.46(80.40,52.91) | 40.43(49.50,32.05) | -1.59(-1.69,-1.50) |
| Croatia | both | 1282.45(1409.63,1173.32) | 1877.63(2321.91,1466.75) | 0.46 | 21.97(24.10,19.97) | 19.53(24.14,15.31) | -0.10(-0.22,0.02) |
| Cuba | both | 1866.16(1988.94,1712.39) | 4586.19(5602.99,3548.80) | 1.46 | 18.66(19.98,17.09) | 23.29(28.42,17.90) | 0.69(0.53,0.85) |
| Cyprus | both | 326.91(374.46,269.99) | 541.09(626.35,400.29) | 0.66 | 57.95(66.57,46.17) | 32.53(37.96,22.62) | -2.43(-2.70,-2.16) |
| Czechia | both | 3192.55(3760.14,2968.32) | 4062.32(4917.71,3294.82) | 0.27 | 23.64(28.00,21.87) | 18.40(22.28,14.90) | 0.21(-0.31,0.74) |
| Democratic Republic of the Congo | both | 10993.22(15008.22,7446.68) | 19997.35(32273.71,12899.88) | 0.82 | 86.78(126.81,58.21) | 71.96(121.72,45.13) | -0.60(-0.76,-0.43) |
| Denmark | both | 3264.59(3486.28,3025.83) | 4572.46(5186.48,3609.67) | 0.40 | 38.24(40.64,35.28) | 36.00(40.80,28.40) | -0.55(-0.81,-0.29) |
| Djibouti | both | 60.30(83.83,39.55) | 145.65(209.39,97.43) | 1.42 | 48.45(63.64,32.72) | 31.66(44.06,21.13) | -1.63(-1.69,-1.57) |
| Dominica | both | 17.31(20.67,15.38) | 21.86(26.67,17.25) | 0.26 | 23.28(27.77,20.69) | 24.28(29.67,19.03) | 0.24(0.19,0.30) |
| Dominican Republic | both | 915.00(1028.57,773.66) | 1936.60(2515.99,1436.21) | 1.12 | 24.32(27.38,20.63) | 22.19(28.70,16.46) | 0.46(0.17,0.74) |
| Ecuador | both | 1712.70(1859.83,1424.22) | 3670.04(4523.14,2838.26) | 1.14 | 34.95(37.58,30.21) | 29.81(36.57,22.53) | 0.25(-0.18,0.67) |
| Egypt | both | 15224.74(16935.18,13669.10) | 22560.48(29260.76,16449.02) | 0.48 | 58.83(68.33,53.37) | 43.58(55.42,32.27) | -0.83(-0.94,-0.72) |
| El Salvador | both | 1041.83(1134.16,884.73) | 1513.77(1880.53,1115.64) | 0.45 | 33.90(37.23,29.33) | 23.10(28.72,17.08) | -1.34(-1.60,-1.08) |
| Equatorial Guinea | both | 159.53(223.34,108.99) | 156.85(262.00,103.19) | -0.02 | 93.43(142.33,61.07) | 42.84(74.23,28.57) | -3.08(-3.35,-2.81) |
| Eritrea | both | 704.08(907.61,474.91) | 1116.51(1437.42,783.50) | 0.59 | 71.55(94.29,49.99) | 49.95(62.92,34.38) | -1.29(-1.34,-1.24) |
| Estonia | both | 332.24(368.61,307.84) | 303.38(387.96,238.13) | -0.09 | 16.60(18.52,15.35) | 10.27(13.09,8.01) | -1.74(-1.90,-1.59) |
| Ethiopia | both | 13499.17(15547.81,10819.39) | 13727.34(15812.65,11712.64) | 0.02 | 71.35(84.83,58.57) | 38.43(44.17,32.63) | -2.35(-2.45,-2.25) |
| Micronesia (Federated States of) | both | 67.91(84.20,52.74) | 56.77(73.10,42.24) | -0.16 | 171.54(219.17,133.45) | 108.37(136.73,84.05) | -1.71(-1.84,-1.58) |
| Fiji | both | 399.56(495.36,319.80) | 412.45(533.87,321.31) | 0.03 | 143.05(177.69,115.79) | 73.44(92.71,58.69) | -2.92(-3.23,-2.60) |
| Finland | both | 1253.12(1405.43,1162.82) | 2043.23(2285.82,1736.40) | 0.63 | 17.27(19.48,15.97) | 14.60(16.30,12.50) | -0.56(-0.65,-0.46) |
| France | both | 22049.88(24041.49,19530.45) | 22060.48(26355.34,18180.36) | 0.00 | 24.91(27.12,21.98) | 12.76(15.11,10.79) | -2.66(-2.91,-2.41) |
| Gabon | both | 305.82(385.87,219.98) | 308.18(399.91,218.32) | 0.01 | 63.62(82.04,44.58) | 37.08(47.01,26.20) | -1.90(-1.94,-1.86) |
| Georgia | both | 1196.34(1595.56,1056.36) | 1221.56(1675.19,974.51) | 0.02 | 22.53(30.48,19.89) | 19.36(26.07,15.50) | 0.85(0.23,1.47) |
| Germany | both | 39887.83(45488.92,36878.51) | 45165.49(51730.33,39407.71) | 0.13 | 30.30(34.48,27.96) | 20.75(23.49,18.24) | -1.25(-1.67,-0.83) |
| Ghana | both | 3075.53(3784.29,1929.34) | 5938.02(7459.49,3587.35) | 0.93 | 55.71(68.48,35.32) | 42.08(51.96,25.74) | -0.60(-0.75,-0.46) |
| Greece | both | 2626.71(2862.75,2385.28) | 6399.32(7428.30,4869.23) | 1.44 | 18.25(19.85,16.37) | 20.39(23.24,16.17) | 1.09(0.66,1.51) |
| Greenland | both | 23.78(26.91,19.15) | 32.00(37.96,23.97) | 0.35 | 92.77(105.60,73.00) | 56.34(66.46,40.93) | -2.22(-2.45,-1.98) |
| Grenada | both | 17.75(20.08,15.54) | 19.05(21.70,16.45) | 0.07 | 22.34(25.35,19.59) | 19.10(21.73,16.42) | -0.54(-0.80,-0.28) |
| Guam | both | 30.49(34.25,26.98) | 53.04(64.79,43.29) | 0.74 | 60.45(68.12,52.33) | 28.73(35.23,23.49) | -2.82(-3.08,-2.56) |
| Guatemala | both | 1627.11(1818.79,1382.19) | 2529.12(3208.85,1987.94) | 0.55 | 45.88(51.28,40.55) | 27.19(34.14,21.66) | -2.20(-2.49,-1.90) |
| Guinea | both | 2214.70(2769.76,1775.74) | 2787.32(3445.51,2134.48) | 0.26 | 70.92(89.72,56.81) | 53.06(65.19,41.08) | -0.63(-0.81,-0.44) |
| Guinea-Bissau | both | 370.04(451.82,292.96) | 385.65(485.05,296.84) | 0.04 | 96.12(115.38,76.33) | 59.15(73.57,45.80) | -1.38(-1.60,-1.17) |
| Guyana | both | 90.25(104.18,77.06) | 118.57(147.61,91.79) | 0.31 | 25.58(29.55,22.02) | 21.57(26.57,16.89) | -0.67(-0.81,-0.53) |
| Haiti | both | 2420.02(3185.18,1641.86) | 3419.91(4792.39,2265.55) | 0.41 | 72.44(105.25,44.93) | 54.34(77.46,35.00) | -0.83(-0.89,-0.76) |
| Honduras | both | 1273.71(1463.89,1035.84) | 3536.33(4627.64,2229.31) | 1.78 | 57.71(68.10,43.75) | 68.62(88.94,41.59) | 0.77(0.54,1.00) |
| Hungary | both | 5385.40(6150.83,5065.33) | 5935.53(7093.26,4887.85) | 0.10 | 39.10(44.73,36.58) | 29.70(35.39,24.36) | -0.60(-1.00,-0.19) |
| Iceland | both | 69.51(79.61,61.67) | 110.81(127.41,89.21) | 0.59 | 23.04(26.32,20.52) | 17.50(20.02,14.24) | -1.21(-1.33,-1.08) |
| India | both | 601995.22(683143.60,491310.12) | 1168381.11(1363608.59,899260.42) | 0.94 | 195.96(226.00,159.48) | 125.11(145.25,95.20) | -1.68(-1.83,-1.54) |
| Indonesia | both | 65596.00(74089.21,56281.35) | 100930.01(113830.80,85012.11) | 0.54 | 83.86(96.81,71.34) | 61.93(70.18,52.48) | -0.95(-1.09,-0.80) |
| Iran (Islamic Republic of) | both | 8206.72(9874.04,7168.93) | 16834.62(18193.27,14588.04) | 1.05 | 42.64(52.57,36.06) | 26.93(29.14,23.24) | -1.59(-1.65,-1.52) |
| Iraq | both | 2101.12(2584.13,1713.68) | 3292.30(4153.25,2576.71) | 0.57 | 28.16(35.34,22.82) | 17.32(21.98,13.82) | -2.16(-2.35,-1.96) |
| Ireland | both | 2300.85(2428.78,2043.38) | 2547.96(2886.91,2134.92) | 0.11 | 56.83(60.09,50.44) | 32.41(36.70,27.25) | -2.40(-2.67,-2.12) |
| Israel | both | 1327.53(1429.04,1221.76) | 2026.88(2481.65,1763.18) | 0.53 | 29.35(31.79,26.79) | 16.18(19.79,14.17) | -2.23(-2.37,-2.09) |
| Italy | both | 24262.65(25174.94,22611.18) | 31345.33(34246.18,26440.44) | 0.29 | 27.24(28.33,25.05) | 16.76(18.17,14.51) | -1.72(-1.81,-1.64) |
| Jamaica | both | 345.15(391.78,310.07) | 641.67(800.46,494.50) | 0.86 | 18.45(21.14,16.60) | 20.73(26.08,15.93) | 0.11(-0.41,0.64) |
| Japan | both | 29862.30(31160.17,27075.99) | 55793.67(63876.36,45377.85) | 0.87 | 19.46(20.39,17.52) | 11.41(12.91,9.61) | -2.00(-2.16,-1.85) |
| Jordan | both | 396.59(468.79,324.55) | 856.00(1027.91,700.02) | 1.16 | 38.78(46.17,31.73) | 17.79(21.48,14.57) | -3.34(-3.65,-3.03) |
| Kazakhstan | both | 6905.06(7411.64,6041.16) | 9772.39(11527.45,8198.03) | 0.42 | 60.49(65.11,52.52) | 68.18(80.18,56.63) | -0.11(-0.78,0.57) |
| Kenya | both | 3501.49(5300.68,2572.85) | 7704.71(10390.59,5821.98) | 1.20 | 47.87(73.98,34.90) | 42.90(57.47,32.45) | -0.12(-0.31,0.06) |
| Kiribati | both | 57.66(69.53,47.37) | 72.92(90.15,57.09) | 0.26 | 184.25(230.00,152.41) | 144.10(176.67,112.54) | -0.81(-0.88,-0.75) |
| Kuwait | both | 81.39(91.48,70.32) | 229.56(277.30,184.37) | 1.82 | 17.21(19.64,14.38) | 12.22(14.91,9.69) | -1.02(-1.28,-0.75) |
| Kyrgyzstan | both | 2657.72(2936.99,2017.39) | 1513.47(1888.69,1282.92) | -0.43 | 92.09(102.10,69.32) | 41.08(51.25,34.89) | -3.48(-4.18,-2.78) |
| Lao People's Democratic Republic | both | 2562.98(3122.95,1992.83) | 2663.07(3360.15,2092.24) | 0.04 | 135.08(165.81,106.06) | 76.70(96.08,61.34) | -2.23(-2.32,-2.13) |
| Latvia | both | 749.75(802.88,695.06) | 433.00(599.06,349.84) | -0.42 | 21.20(22.64,19.63) | 9.92(13.53,7.94) | -2.55(-2.91,-2.19) |
| Lebanon | both | 694.75(864.89,564.07) | 1185.21(1498.59,917.94) | 0.71 | 37.81(47.38,30.60) | 23.35(29.69,18.13) | -1.57(-1.63,-1.51) |
| Lesotho | both | 999.76(1437.92,674.02) | 1115.96(1526.16,787.35) | 0.12 | 114.66(164.91,77.92) | 104.40(141.45,74.25) | 0.15(-0.07,0.37) |
| Liberia | both | 436.67(520.79,361.08) | 534.44(715.02,398.06) | 0.22 | 40.93(49.57,34.27) | 29.75(39.56,23.04) | -1.22(-1.37,-1.06) |
| Libya | both | 558.89(721.67,433.15) | 1172.90(1477.19,881.61) | 1.10 | 33.34(43.46,25.32) | 26.61(33.35,19.92) | -0.68(-0.87,-0.49) |
| Lithuania | both | 1473.15(1567.53,1255.89) | 778.73(1009.90,631.10) | -0.47 | 32.82(34.93,27.97) | 12.21(15.80,9.90) | -3.60(-3.77,-3.42) |
| Luxembourg | both | 176.39(194.64,162.93) | 221.38(259.79,182.57) | 0.26 | 32.59(36.01,29.99) | 19.91(23.25,16.54) | -1.91(-2.00,-1.83) |
| North Macedonia | both | 658.63(725.64,555.56) | 652.38(835.67,514.86) | -0.01 | 42.89(47.91,35.31) | 24.72(31.66,19.85) | -2.00(-2.15,-1.85) |
| Madagascar | both | 4165.09(4705.07,3657.11) | 6029.57(7697.73,4600.64) | 0.45 | 78.35(90.30,68.98) | 65.01(82.25,49.59) | -0.86(-1.05,-0.67) |
| Malawi | both | 1815.67(2165.30,1459.77) | 2402.23(2842.48,1966.71) | 0.32 | 50.14(58.85,40.90) | 37.21(43.72,30.74) | -1.16(-1.28,-1.04) |
| Malaysia | both | 5101.29(6029.28,4196.21) | 7473.12(10018.31,5862.09) | 0.46 | 65.33(78.15,53.95) | 34.38(46.19,27.00) | -3.28(-3.76,-2.81) |
| Maldives | both | 96.24(112.55,73.36) | 122.96(146.79,101.55) | 0.28 | 143.31(164.18,113.82) | 51.64(61.43,42.42) | -4.06(-4.27,-3.85) |
| Mali | both | 2894.00(3419.87,2199.19) | 4573.53(5877.32,3388.96) | 0.58 | 74.45(87.60,57.59) | 56.29(70.70,43.08) | -0.93(-1.02,-0.84) |
| Malta | both | 106.17(116.52,96.17) | 157.24(180.58,131.85) | 0.48 | 26.29(28.86,23.72) | 15.40(17.65,12.97) | -2.05(-2.15,-1.95) |
| Marshall Islands | both | 22.16(30.27,17.63) | 26.41(35.12,19.09) | 0.19 | 155.34(214.45,124.04) | 104.71(136.15,77.56) | -1.31(-1.39,-1.23) |
| Mauritania | both | 526.13(643.93,379.86) | 524.70(669.58,401.89) | 0.00 | 57.35(70.01,41.27) | 28.76(36.13,22.50) | -2.16(-2.37,-1.95) |
| Mauritius | both | 450.09(482.27,397.57) | 537.35(678.17,429.16) | 0.19 | 73.83(79.48,64.51) | 34.90(44.16,28.14) | -2.77(-2.98,-2.56) |
| Mexico | both | 16909.84(17646.45,15104.18) | 38524.31(43747.72,32632.09) | 1.28 | 49.17(51.63,43.38) | 36.93(41.88,31.20) | -1.17(-1.30,-1.05) |
| Republic of Moldova | both | 1830.32(1957.72,1500.73) | 971.39(1200.33,822.75) | -0.47 | 46.98(50.47,37.85) | 16.75(20.71,14.20) | -4.13(-4.65,-3.61) |
| Mongolia | both | 526.36(622.36,411.73) | 453.13(607.84,356.96) | -0.14 | 56.55(66.64,44.27) | 27.81(34.98,22.73) | -3.22(-3.59,-2.85) |
| Montenegro | both | 53.94(63.29,44.43) | 84.98(99.79,68.94) | 0.58 | 9.76(11.52,7.91) | 9.32(10.91,7.48) | 0.08(-0.04,0.20) |
| Morocco | both | 4982.24(6516.97,3970.68) | 9373.44(11681.96,7232.59) | 0.88 | 43.72(59.43,34.60) | 37.27(46.80,29.08) | -0.60(-0.77,-0.43) |
| Mozambique | both | 2450.96(2992.43,1959.99) | 3758.84(4746.17,2982.79) | 0.53 | 44.61(54.55,36.64) | 38.91(49.16,31.11) | -0.43(-0.55,-0.31) |
| Myanmar | both | 37654.05(46073.12,27629.72) | 43284.40(50184.91,33261.44) | 0.15 | 193.65(233.07,139.34) | 115.56(133.27,88.11) | -1.96(-2.07,-1.85) |
| Namibia | both | 658.59(905.20,469.91) | 790.97(1012.49,613.90) | 0.20 | 102.83(142.00,74.53) | 64.34(82.23,49.72) | -1.81(-2.04,-1.59) |
| Nepal | both | 19609.41(23556.79,15062.76) | 40799.55(48382.74,30907.21) | 1.08 | 273.02(326.19,213.16) | 231.20(270.35,175.79) | -0.50(-0.66,-0.34) |
| Netherlands | both | 6933.76(7449.41,6377.65) | 10766.17(12239.88,8585.77) | 0.55 | 33.47(35.95,30.75) | 28.62(32.43,22.80) | -0.87(-1.07,-0.67) |
| New Zealand | both | 1541.77(1658.85,1440.98) | 2396.17(2686.42,2024.65) | 0.55 | 39.27(42.28,36.49) | 28.28(31.59,24.04) | -1.57(-1.75,-1.38) |
| Nicaragua | both | 430.19(481.90,379.51) | 1342.92(1549.44,1011.24) | 2.12 | 29.16(33.87,25.49) | 40.05(46.03,29.96) | 1.26(0.89,1.63) |
| Niger | both | 1972.70(2504.63,1518.20) | 3618.96(4954.19,2581.56) | 0.83 | 74.46(98.42,57.15) | 52.11(70.27,37.99) | -1.01(-1.20,-0.83) |
| Nigeria | both | 17748.63(21947.31,14084.93) | 24505.92(30356.44,19594.92) | 0.38 | 45.48(56.61,36.02) | 33.92(41.73,27.06) | -0.84(-0.96,-0.72) |
| Democratic People's Republic of Korea | both | 19506.70(25359.26,14226.37) | 31704.02(37876.55,25210.16) | 0.63 | 174.31(231.33,126.70) | 112.77(136.52,90.31) | -1.60(-1.77,-1.44) |
| Northern Mariana Islands | both | 10.31(12.43,8.62) | 17.37(21.01,14.80) | 0.68 | 81.71(94.96,70.41) | 49.27(58.79,42.20) | -1.71(-1.78,-1.65) |
| Norway | both | 1523.06(1784.67,1413.64) | 2770.71(3026.39,2116.92) | 0.82 | 20.52(23.97,19.06) | 25.85(28.18,19.70) | 0.90(0.64,1.16) |
| Oman | both | 184.89(248.28,137.74) | 243.74(281.30,191.08) | 0.32 | 38.53(51.86,28.77) | 25.45(29.63,19.45) | -0.95(-1.22,-0.68) |
| Pakistan | both | 64996.66(82333.57,52750.79) | 82757.82(102124.46,67880.21) | 0.27 | 130.64(167.50,105.33) | 100.39(122.70,83.09) | -1.08(-1.33,-0.83) |
| Palestine | both | 342.22(448.24,265.13) | 487.91(568.52,405.34) | 0.43 | 44.35(58.60,34.47) | 26.93(31.45,22.32) | -1.94(-2.08,-1.81) |
| Panama | both | 349.31(391.69,312.03) | 876.66(1098.42,661.03) | 1.51 | 24.17(27.41,21.33) | 20.31(25.48,15.31) | -1.05(-1.35,-0.76) |
| Papua New Guinea | both | 3338.04(4000.84,2738.95) | 7154.08(9108.31,5538.75) | 1.14 | 240.12(286.42,190.92) | 209.49(259.45,162.01) | -0.39(-0.45,-0.33) |
| Paraguay | both | 430.54(552.21,374.83) | 1147.83(1464.33,878.08) | 1.67 | 20.67(27.05,17.70) | 22.12(28.21,16.90) | 0.52(0.29,0.76) |
| Peru | both | 3320.13(4232.22,2825.32) | 6961.17(8989.86,5132.37) | 1.10 | 27.00(36.91,22.75) | 21.31(27.63,15.64) | -0.17(-0.45,0.10) |
| Philippines | both | 20035.10(22255.69,17365.64) | 33228.91(41083.32,27251.61) | 0.66 | 82.95(93.82,72.70) | 50.85(64.72,41.71) | -1.63(-1.78,-1.49) |
| Poland | both | 14579.29(15101.45,13622.34) | 11348.62(13769.22,9487.84) | -0.22 | 34.65(35.99,32.14) | 15.37(18.69,12.87) | -2.78(-2.97,-2.60) |
| Portugal | both | 4624.35(5029.39,4278.56) | 7051.85(8062.20,5903.21) | 0.52 | 36.33(39.27,33.31) | 23.69(26.93,20.21) | -1.66(-1.82,-1.51) |
| Puerto Rico | both | 963.78(1052.71,862.97) | 1832.10(2280.56,1391.04) | 0.90 | 28.05(30.70,24.87) | 21.92(27.59,16.99) | -1.60(-1.93,-1.26) |
| Qatar | both | 21.46(30.23,17.01) | 66.19(99.20,48.76) | 2.08 | 32.84(47.51,25.42) | 22.97(32.41,17.71) | -1.22(-1.49,-0.96) |
| Romania | both | 14278.08(15297.80,11364.35) | 8106.34(10100.52,6606.59) | -0.43 | 59.46(64.04,46.15) | 20.77(25.97,16.82) | -3.76(-4.01,-3.51) |
| Russian Federation | both | 57982.19(62626.19,47061.33) | 39390.41(46787.63,33694.55) | -0.32 | 34.55(37.36,27.49) | 16.70(19.87,14.31) | -3.28(-3.62,-2.95) |
| Rwanda | both | 2761.43(3390.39,2064.25) | 2519.69(3363.40,1928.29) | -0.09 | 97.56(117.55,74.50) | 50.27(66.02,39.13) | -2.94(-3.21,-2.67) |
| Saint Lucia | both | 29.69(32.69,26.97) | 58.69(69.71,47.94) | 0.98 | 35.91(39.66,32.55) | 28.94(34.31,23.69) | -1.29(-1.56,-1.03) |
| Saint Vincent and the Grenadines | both | 10.71(12.30,9.47) | 20.07(23.04,16.98) | 0.87 | 15.20(17.44,13.41) | 16.12(18.48,13.65) | 0.01(-0.19,0.21) |
| Samoa | both | 104.38(135.66,82.97) | 104.83(131.69,85.28) | 0.00 | 138.21(180.52,110.64) | 82.52(103.26,67.10) | -1.87(-2.01,-1.73) |
| Sao Tome and Principe | both | 59.64(69.42,48.54) | 78.65(97.70,59.45) | 0.32 | 102.65(118.29,84.86) | 88.33(108.64,66.73) | -0.52(-0.62,-0.41) |
| Saudi Arabia | both | 2561.05(3533.90,1938.22) | 3723.31(4532.17,2953.14) | 0.45 | 55.43(75.66,41.43) | 31.74(37.64,25.73) | -2.00(-2.08,-1.91) |
| Senegal | both | 1950.31(2345.54,1499.30) | 2762.95(3432.65,2158.19) | 0.42 | 65.76(79.08,50.94) | 41.77(50.91,33.46) | -1.22(-1.49,-0.95) |
| Serbia | both | 3395.21(3964.97,3019.21) | 3865.05(4773.20,3107.41) | 0.14 | 34.01(40.16,30.13) | 25.15(31.12,20.29) | -1.17(-1.35,-0.98) |
| Seychelles | both | 26.29(28.79,22.62) | 30.51(34.78,26.20) | 0.16 | 46.90(51.38,40.37) | 32.67(37.18,27.98) | -1.48(-1.61,-1.34) |
| Sierra Leone | both | 1186.07(1461.27,911.34) | 1478.62(1907.66,1108.48) | 0.25 | 65.07(79.24,50.50) | 44.74(56.56,34.38) | -0.92(-1.10,-0.74) |
| Singapore | both | 958.46(1022.35,824.08) | 749.92(995.86,626.93) | -0.22 | 54.18(58.02,46.25) | 10.58(14.07,8.82) | -5.99(-6.32,-5.65) |
| Slovakia | both | 1052.00(1532.09,946.37) | 1146.70(1483.86,901.15) | 0.09 | 18.39(26.51,16.52) | 12.56(16.20,9.90) | -0.89(-1.06,-0.71) |
| Slovenia | both | 672.95(837.05,527.39) | 638.62(800.88,497.73) | -0.05 | 28.40(35.13,22.30) | 12.71(16.07,9.90) | -3.60(-3.96,-3.25) |
| Solomon Islands | both | 186.81(239.81,142.55) | 346.68(418.20,275.40) | 0.86 | 168.90(217.12,131.64) | 145.87(169.97,118.53) | -0.45(-0.50,-0.39) |
| Somalia | both | 2125.61(3041.81,1435.35) | 3992.27(6427.71,2675.20) | 0.88 | 89.31(133.51,62.19) | 66.97(109.05,45.16) | -0.92(-0.99,-0.85) |
| South Africa | both | 12236.05(14044.11,10581.77) | 17826.87(20124.83,16467.08) | 0.46 | 62.79(72.98,53.59) | 46.60(51.97,42.43) | -1.35(-1.93,-0.77) |
| Republic of Korea | both | 11476.80(12517.66,8397.69) | 15506.39(17472.24,12625.84) | 0.35 | 62.49(69.62,41.58) | 19.16(21.58,15.35) | -4.87(-5.23,-4.50) |
| South Sudan | both | 1396.10(1780.32,917.38) | 1330.77(1813.80,952.44) | -0.05 | 58.97(75.45,40.41) | 40.87(53.86,29.40) | -1.30(-1.41,-1.20) |
| Spain | both | 22694.26(24151.62,20391.43) | 36560.14(42491.33,29179.84) | 0.61 | 42.29(45.18,37.58) | 29.05(33.19,24.12) | -1.53(-1.68,-1.39) |
| Sri Lanka | both | 9541.74(10390.62,8298.27) | 13596.93(17633.98,9535.51) | 0.42 | 113.98(124.21,99.12) | 65.33(83.62,46.00) | -1.24(-1.50,-0.98) |
| Sudan | both | 6244.48(8346.17,4004.61) | 7190.93(9718.31,4822.51) | 0.15 | 72.74(100.40,45.49) | 45.18(60.99,30.73) | -1.72(-1.84,-1.61) |
| Suriname | both | 63.21(72.70,56.44) | 116.69(139.22,96.53) | 0.85 | 25.40(29.09,22.74) | 20.97(24.94,17.35) | -0.86(-1.14,-0.57) |
| Eswatini | both | 251.15(345.50,184.56) | 319.48(417.24,238.25) | 0.27 | 97.63(135.29,71.75) | 65.53(83.65,49.90) | -1.17(-1.51,-0.82) |
| Sweden | both | 2481.45(2978.21,2270.74) | 4477.79(5046.44,3398.38) | 0.80 | 15.11(18.05,13.86) | 17.85(20.03,13.56) | 0.59(0.45,0.74) |
| Switzerland | both | 2536.84(2743.80,2300.49) | 3074.26(3503.83,2563.07) | 0.21 | 22.84(24.69,20.73) | 14.94(16.87,12.58) | -1.59(-1.70,-1.47) |
| Syrian Arab Republic | both | 2223.86(2795.24,1803.99) | 3249.08(4429.61,2417.02) | 0.46 | 45.74(59.76,36.85) | 35.02(46.91,26.60) | -1.23(-1.39,-1.06) |
| Taiwan (Province of China) | both | 5150.41(5518.27,4562.29) | 9968.91(12655.63,7858.46) | 0.94 | 45.04(48.76,39.14) | 24.42(31.06,19.16) | -2.45(-2.65,-2.25) |
| Tajikistan | both | 1682.47(1851.59,1436.06) | 1669.25(2395.28,1337.36) | -0.01 | 61.52(67.64,51.88) | 48.85(72.69,38.61) | -0.83(-0.97,-0.69) |
| United Republic of Tanzania | both | 4189.95(4866.92,3519.93) | 7001.10(8305.02,5758.76) | 0.67 | 41.44(48.02,35.07) | 31.42(36.63,25.67) | -0.92(-0.94,-0.90) |
| Thailand | both | 20374.26(23500.80,16503.20) | 23924.78(31299.86,17913.15) | 0.17 | 70.22(81.16,56.47) | 24.83(32.40,18.64) | -4.12(-4.32,-3.91) |
| Bahamas | both | 23.58(26.65,20.91) | 50.84(62.85,40.71) | 1.16 | 16.36(18.53,14.42) | 14.58(18.06,11.67) | -0.48(-0.57,-0.40) |
| Gambia | both | 196.07(254.95,142.83) | 423.88(535.75,325.24) | 1.16 | 62.56(79.47,46.95) | 48.78(61.02,37.97) | -0.77(-0.98,-0.56) |
| Timor-Leste | both | 244.29(310.52,184.31) | 515.69(651.23,391.01) | 1.11 | 99.34(127.46,74.60) | 77.66(98.03,59.67) | -1.07(-1.26,-0.89) |
| Togo | both | 741.46(881.36,590.72) | 1331.91(1729.40,985.20) | 0.80 | 65.75(78.37,51.99) | 43.17(55.37,33.10) | -1.23(-1.37,-1.08) |
| Tonga | both | 36.46(42.24,30.54) | 41.97(50.39,33.70) | 0.15 | 79.50(92.07,66.40) | 54.91(65.80,44.13) | -1.37(-1.56,-1.19) |
| Trinidad and Tobago | both | 200.75(217.96,187.51) | 271.22(360.45,200.63) | 0.35 | 25.67(27.97,23.78) | 15.58(20.59,11.61) | -1.86(-2.04,-1.68) |
| Tunisia | both | 1380.99(1833.42,1118.64) | 2649.25(3506.54,1962.57) | 0.92 | 34.31(45.45,27.61) | 24.02(31.61,17.97) | -1.30(-1.35,-1.25) |
| Turkey | both | 18823.79(21342.72,15855.27) | 32980.77(40822.56,22668.26) | 0.75 | 60.16(68.57,50.41) | 40.64(50.33,28.01) | -0.75(-1.10,-0.39) |
| Turkmenistan | both | 843.22(911.67,652.69) | 442.20(806.45,323.06) | -0.48 | 50.03(54.39,38.30) | 13.26(25.01,9.64) | -5.52(-6.07,-4.98) |
| Uganda | both | 4163.77(5909.84,2791.17) | 5480.31(7142.08,4037.28) | 0.32 | 68.16(96.71,45.78) | 44.02(56.25,33.00) | -1.84(-1.97,-1.71) |
| Ukraine | both | 33452.17(36237.11,26048.30) | 12181.14(18471.32,9741.58) | -0.64 | 49.76(54.11,38.14) | 15.96(24.16,12.78) | -5.51(-6.05,-4.97) |
| United Arab Emirates | both | 309.96(416.62,231.71) | 1777.87(2382.56,1216.16) | 4.74 | 77.58(104.08,61.14) | 45.50(58.31,35.60) | -1.99(-2.41,-1.57) |
| United Kingdom | both | 37656.90(43240.90,35477.72) | 51878.55(55228.57,43732.81) | 0.38 | 39.59(45.62,37.28) | 36.54(38.77,30.82) | -0.29(-0.35,-0.23) |
| United States of America | both | 106097.57(124040.82,98990.18) | 224987.73(240467.74,181616.38) | 1.12 | 31.61(36.81,29.57) | 37.72(40.16,30.84) | 0.50(0.31,0.69) |
| Uruguay | both | 1290.67(1538.13,1173.70) | 2154.63(2388.68,1888.04) | 0.67 | 33.38(40.06,30.34) | 35.61(39.19,31.35) | 0.08(-0.18,0.35) |
| Uzbekistan | both | 4929.39(5378.84,3868.13) | 3836.51(5646.20,3034.95) | -0.22 | 46.84(51.27,36.58) | 28.98(47.54,23.23) | -2.67(-3.24,-2.10) |
| Vanuatu | both | 89.64(123.36,62.71) | 172.00(227.71,128.60) | 0.92 | 166.47(227.78,119.00) | 121.24(156.46,92.27) | -1.33(-1.45,-1.22) |
| Venezuela (Bolivarian Republic of) | both | 2045.71(2303.22,1856.31) | 7062.18(9045.26,5420.84) | 2.45 | 23.29(26.98,20.73) | 26.08(33.27,20.01) | -0.03(-0.27,0.21) |
| Viet nam | both | 26078.41(33159.57,9882.78) | 36229.40(45192.44,14126.81) | 0.39 | 73.93(93.74,27.59) | 47.62(58.89,18.73) | -1.52(-1.55,-1.48) |
| Virginia | both | 2384.68(2744.32,2220.14) | 5302.62(6287.36,4184.92) | 1.22 | 33.56(38.70,31.18) | 36.12(42.79,28.71) | -0.04(-0.29,0.21) |
| Yemen | both | 3340.81(4736.40,2294.15) | 5737.54(7400.39,4432.82) | 0.72 | 79.73(116.73,53.62) | 52.51(66.84,41.42) | -1.61(-1.71,-1.51) |
| Zambia | both | 1569.56(1885.79,1283.34) | 2388.06(2919.19,1918.79) | 0.52 | 57.54(70.37,47.35) | 41.65(50.26,33.77) | -1.31(-1.51,-1.10) |
| Zimbabwe | both | 1819.89(2072.73,1559.61) | 2891.11(3774.41,1999.77) | 0.59 | 53.07(60.13,45.66) | 49.07(63.36,34.59) | 0.13(-0.05,0.30) |
| Monaco | both | 15.29(19.17,12.21) | 19.77(23.23,15.86) | 0.29 | 18.69(23.27,14.83) | 17.04(20.12,13.67) | -0.09(-0.23,0.06) |
| San Marino | both | 5.15(6.26,4.32) | 10.51(14.27,7.34) | 1.04 | 15.82(19.14,13.19) | 13.39(18.35,9.26) | -0.26(-0.46,-0.07) |
| Saint Kitts and Nevis | both | 8.93(10.15,7.90) | 10.95(13.09,9.18) | 0.23 | 24.76(28.21,21.88) | 20.20(24.03,17.06) | -0.49(-0.64,-0.34) |
| Cook Islands | both | 7.61(8.69,6.47) | 7.50(9.17,6.22) | -0.01 | 71.88(82.58,60.06) | 32.74(39.98,27.05) | -2.87(-3.01,-2.73) |
| Nauru | both | 4.53(5.64,3.56) | 2.93(3.68,2.12) | -0.35 | 149.05(179.52,121.07) | 98.54(120.02,73.89) | -1.42(-1.73,-1.11) |
| Niue | both | 2.25(2.76,1.79) | 1.21(1.52,0.92) | -0.46 | 95.82(117.27,76.13) | 58.07(73.18,44.19) | -1.94(-2.02,-1.86) |
| Palau | both | 12.41(15.53,9.95) | 16.64(20.55,13.23) | 0.34 | 153.00(193.39,123.52) | 107.93(130.08,87.06) | -1.14(-1.21,-1.06) |
| Tokelau | both | 1.60(1.98,1.31) | 0.76(0.96,0.59) | -0.52 | 124.05(153.40,101.53) | 63.50(79.32,49.21) | -2.40(-2.46,-2.35) |
| Tuvalu | both | 9.57(12.03,7.62) | 7.44(9.59,5.71) | -0.22 | 167.79(215.64,132.25) | 87.49(110.57,67.74) | -2.32(-2.42,-2.22) |
| Afghanistan | male | 3283.82(3961.66,2606.52) | 3291.32(4005.04,2524.12) | 0.00 | 100.74(120.38,81.96) | 66.42(79.38,52.00) | -1.58(-1.83,-1.32) |
| Albania | male | 646.19(707.56,468.68) | 535.53(701.19,402.96) | -0.17 | 96.80(106.50,67.35) | 29.67(38.62,22.39) | -4.50(-5.05,-3.94) |
| Algeria | male | 2577.08(3474.96,1890.37) | 4368.23(5670.32,3343.98) | 0.70 | 61.32(80.27,45.61) | 33.18(42.61,25.49) | -2.21(-2.29,-2.12) |
| American Samoa | male | 10.87(12.28,9.41) | 13.59(16.14,11.56) | 0.25 | 128.50(145.56,110.99) | 74.00(87.63,64.06) | -2.09(-2.18,-2.00) |
| Andorra | male | 11.98(15.92,9.06) | 26.73(33.09,20.76) | 1.23 | 58.15(74.20,45.07) | 38.99(48.30,30.33) | -1.36(-1.49,-1.23) |
| Angola | male | 1347.41(1728.27,987.52) | 1771.37(2217.82,1384.28) | 0.31 | 83.49(104.20,61.86) | 47.85(58.57,38.02) | -2.11(-2.22,-1.99) |
| Antigua and Barbuda | male | 4.17(4.84,3.60) | 5.98(7.41,4.81) | 0.44 | 19.85(22.97,17.17) | 15.57(19.20,12.53) | -0.75(-1.13,-0.36) |
| Argentina | male | 6252.75(6754.06,5806.41) | 10576.21(12056.48,9132.34) | 0.69 | 50.08(54.32,46.10) | 48.29(55.31,41.39) | -0.40(-0.66,-0.14) |
| Armenia | male | 691.38(739.12,643.48) | 718.73(857.93,589.56) | 0.04 | 77.82(83.54,71.86) | 46.14(54.89,38.12) | -2.27(-2.48,-2.07) |
| Australia | male | 4501.27(4750.21,4228.46) | 6208.41(7069.63,5360.33) | 0.38 | 59.39(62.99,55.30) | 30.72(34.95,26.53) | -2.47(-2.77,-2.16) |
| Austria | male | 1503.65(1602.41,1407.06) | 1933.35(2182.94,1692.55) | 0.29 | 36.38(38.77,33.91) | 23.79(26.91,20.85) | -1.36(-1.51,-1.22) |
| Azerbaijan | male | 1048.48(1144.87,944.49) | 1145.24(1640.29,889.50) | 0.09 | 61.16(66.92,55.26) | 40.84(60.30,31.10) | -1.91(-2.53,-1.29) |
| Bahrain | male | 50.83(59.19,43.21) | 92.64(120.35,72.58) | 0.82 | 96.32(110.69,81.97) | 41.01(49.99,32.44) | -3.14(-3.76,-2.51) |
| Bangladesh | male | 42867.07(51291.24,36985.87) | 44639.98(74931.26,33836.38) | 0.04 | 185.67(234.26,159.82) | 73.75(126.04,56.37) | -3.34(-3.72,-2.96) |
| Barbados | male | 23.84(27.38,20.46) | 37.37(45.67,29.52) | 0.57 | 19.55(22.44,16.74) | 17.88(21.77,14.16) | -0.83(-1.11,-0.56) |
| Belarus | male | 3709.13(3993.10,3435.20) | 1477.79(1913.01,1142.05) | -0.60 | 92.68(100.03,85.38) | 28.16(36.14,22.10) | -5.35(-5.96,-4.75) |
| Belgium | male | 4456.22(4736.42,4180.99) | 4345.79(4928.04,3800.59) | -0.02 | 77.03(82.06,71.54) | 40.47(45.74,35.47) | -2.78(-2.97,-2.59) |
| Belize | male | 13.74(15.75,11.89) | 52.39(62.39,43.75) | 2.81 | 29.44(34.25,25.26) | 41.48(49.17,34.33) | 0.76(0.14,1.39) |
| Benin | male | 792.60(957.43,608.79) | 1045.52(1412.50,770.61) | 0.32 | 86.81(105.56,66.19) | 53.48(69.21,39.82) | -1.43(-1.58,-1.28) |
| Bermuda | male | 6.91(7.85,6.06) | 11.96(14.65,9.62) | 0.73 | 30.69(34.80,26.80) | 21.83(26.62,17.54) | -0.98(-1.06,-0.90) |
| Bhutan | male | 138.15(199.14,96.05) | 270.90(424.57,186.07) | 0.96 | 167.13(240.31,119.61) | 114.72(178.16,80.22) | -1.23(-1.27,-1.18) |
| Bolivia (Plurinational State of) | male | 946.88(1172.12,717.77) | 1804.27(2308.95,1337.84) | 0.91 | 71.60(88.07,55.12) | 55.72(71.61,41.24) | -0.80(-0.87,-0.73) |
| Bosnia and Herzegovina | male | 669.14(724.00,605.27) | 746.72(926.38,589.50) | 0.12 | 50.39(54.60,44.94) | 31.51(38.79,25.20) | -1.80(-2.01,-1.59) |
| Botswana | male | 285.85(387.92,209.65) | 389.05(511.53,285.11) | 0.36 | 140.57(190.58,105.24) | 89.11(115.10,66.66) | -2.09(-2.57,-1.60) |
| Brazil | male | 23877.90(24853.72,22604.18) | 40792.86(43550.52,37262.48) | 0.71 | 74.14(77.78,68.92) | 43.62(46.84,39.45) | -2.19(-2.37,-2.02) |
| Brunei Darussalam | male | 42.91(49.21,32.25) | 50.98(60.24,42.98) | 0.19 | 204.13(232.00,144.17) | 105.13(121.03,88.87) | -1.92(-2.35,-1.48) |
| Bulgaria | male | 2519.68(2744.82,2303.83) | 1852.71(2316.69,1457.68) | -0.26 | 51.46(56.24,46.94) | 31.39(38.89,24.71) | -2.12(-2.33,-1.90) |
| Burkina Faso | male | 882.39(1062.70,707.88) | 1412.15(1742.25,1113.50) | 0.60 | 49.48(59.50,40.57) | 39.51(47.70,31.59) | -0.83(-1.01,-0.65) |
| Burundi | male | 1281.18(1621.86,888.63) | 1417.73(1892.77,1012.38) | 0.11 | 125.23(156.40,88.53) | 76.39(99.49,55.35) | -1.91(-2.00,-1.82) |
| Cambodia | male | 1664.35(1973.30,1360.49) | 2972.82(3509.27,2351.28) | 0.79 | 108.11(132.05,86.96) | 82.92(97.28,66.44) | -0.92(-0.96,-0.89) |
| Cameroon | male | 1531.36(1888.48,1184.39) | 2433.13(3180.58,1674.96) | 0.59 | 83.99(102.81,64.10) | 50.65(64.04,36.72) | -1.58(-1.70,-1.47) |
| Canada | male | 5939.82(6279.08,5544.83) | 9847.80(11213.91,8405.20) | 0.66 | 47.35(50.21,43.67) | 30.75(35.01,26.18) | -1.95(-2.12,-1.78) |
| Cabo Verde | male | 72.44(84.82,46.27) | 56.19(79.75,47.35) | -0.22 | 72.07(83.49,46.08) | 33.50(47.41,28.27) | -3.43(-4.17,-2.69) |
| Central African Republic | male | 559.42(720.57,393.27) | 789.95(1056.27,515.66) | 0.41 | 123.14(162.87,86.44) | 101.06(128.87,66.45) | -0.70(-0.73,-0.67) |
| Chad | male | 1052.44(1522.85,767.45) | 1665.51(2169.16,1161.96) | 0.58 | 82.19(121.34,59.59) | 61.38(78.96,43.49) | -0.85(-0.92,-0.79) |
| Chile | male | 1629.71(1749.31,1508.72) | 3260.79(3695.86,2812.49) | 1.00 | 42.60(45.86,38.91) | 32.87(37.24,28.01) | -0.46(-0.73,-0.18) |
| China | male | 665056.15(771944.41,500796.67) | 628314.65(759702.44,518961.46) | -0.06 | 272.74(310.39,212.15) | 97.78(115.76,82.16) | -3.75(-3.93,-3.56) |
| Colombia | male | 3488.87(3741.21,3182.07) | 9077.84(11741.02,6785.02) | 1.60 | 49.26(53.39,44.06) | 37.53(48.70,28.00) | -1.31(-1.58,-1.04) |
| Comoros | male | 78.80(104.37,48.86) | 93.91(120.21,72.01) | 0.19 | 83.95(109.90,55.90) | 50.32(63.88,38.74) | -1.94(-2.11,-1.78) |
| Congo | male | 400.47(511.74,275.73) | 487.76(628.44,283.93) | 0.22 | 103.12(130.52,73.31) | 54.10(67.32,30.67) | -2.51(-2.65,-2.36) |
| Costa Rica | male | 246.27(275.02,218.51) | 734.40(923.57,569.68) | 1.98 | 32.09(35.96,28.19) | 32.80(41.28,25.48) | -0.64(-1.22,-0.06) |
| Côte d'Ivoire | male | 1563.94(1905.01,1191.78) | 2333.94(3021.17,1706.60) | 0.49 | 88.30(106.31,68.00) | 52.28(65.61,39.01) | -1.77(-1.84,-1.69) |
| Croatia | male | 830.65(903.29,750.91) | 1140.48(1425.52,887.21) | 0.37 | 39.29(42.70,35.42) | 32.01(39.77,25.24) | -0.46(-0.64,-0.29) |
| Cuba | male | 1051.53(1123.99,977.48) | 2553.52(3138.34,2030.85) | 1.43 | 21.63(23.20,20.01) | 28.37(34.84,22.60) | 0.79(0.60,0.98) |
| Cyprus | male | 180.41(209.78,152.67) | 311.40(361.29,242.55) | 0.73 | 65.93(75.89,54.72) | 41.42(48.35,30.78) | -1.88(-2.21,-1.55) |
| Czechia | male | 2162.65(2306.97,2018.15) | 2452.41(2994.33,1971.96) | 0.13 | 42.98(45.76,40.00) | 27.35(33.20,22.06) | -0.39(-0.98,0.20) |
| Democratic Republic of the Congo | male | 5360.78(6944.23,3607.51) | 8529.57(11381.87,5209.48) | 0.59 | 89.84(114.34,61.00) | 71.92(95.30,44.09) | -0.79(-0.91,-0.66) |
| Denmark | male | 1898.51(2030.84,1767.92) | 2267.68(2590.00,1958.45) | 0.19 | 55.15(58.96,51.11) | 42.00(48.09,36.20) | -1.27(-1.45,-1.08) |
| Djibouti | male | 35.86(53.74,22.89) | 92.13(141.16,58.50) | 1.57 | 64.83(90.46,42.50) | 40.37(60.23,26.96) | -1.82(-1.88,-1.75) |
| Dominica | male | 10.93(13.62,9.51) | 15.01(18.67,11.54) | 0.37 | 38.00(47.82,33.31) | 37.53(46.36,29.45) | -0.10(-0.22,0.02) |
| Dominican Republic | male | 494.87(572.28,423.56) | 1083.34(1502.54,800.82) | 1.19 | 26.61(30.67,22.99) | 26.43(36.14,19.79) | 0.81(0.56,1.06) |
| Ecuador | male | 945.08(1029.73,819.05) | 2064.09(2608.75,1554.92) | 1.18 | 38.66(42.02,33.32) | 36.52(45.69,26.78) | 0.62(0.22,1.02) |
| Egypt | male | 8378.02(9367.91,7472.72) | 13852.23(18590.86,9278.62) | 0.65 | 64.56(72.26,57.86) | 45.96(62.28,30.72) | -1.07(-1.20,-0.94) |
| El Salvador | male | 514.48(564.26,452.98) | 672.53(840.33,505.47) | 0.31 | 36.75(40.41,32.19) | 25.16(31.64,18.82) | -1.34(-1.59,-1.09) |
| Equatorial Guinea | male | 81.48(109.97,53.27) | 75.47(101.85,42.54) | -0.07 | 110.31(147.41,73.14) | 49.63(65.79,27.35) | -3.22(-3.57,-2.86) |
| Eritrea | male | 381.00(522.45,216.96) | 570.93(767.76,389.85) | 0.50 | 103.93(143.83,56.50) | 65.98(85.61,45.16) | -1.70(-1.80,-1.60) |
| Estonia | male | 205.47(221.28,189.54) | 168.86(214.61,131.38) | -0.18 | 31.23(33.57,28.80) | 17.65(22.37,13.82) | -2.00(-2.18,-1.82) |
| Ethiopia | male | 8020.35(9915.30,6243.85) | 8396.90(10153.07,6771.83) | 0.05 | 88.21(111.93,67.93) | 47.55(57.12,38.63) | -2.26(-2.32,-2.20) |
| Micronesia (Federated States of) | male | 39.34(50.56,29.52) | 30.48(39.39,22.12) | -0.23 | 206.83(272.65,156.42) | 126.87(157.40,98.74) | -1.82(-1.94,-1.71) |
| Fiji | male | 252.86(306.79,201.05) | 256.68(324.77,200.63) | 0.02 | 196.53(236.10,158.30) | 110.29(133.84,88.95) | -2.71(-3.09,-2.33) |
| Finland | male | 868.37(927.74,808.29) | 1307.74(1493.38,1127.91) | 0.51 | 35.38(37.97,32.69) | 22.91(26.17,19.77) | -1.54(-1.66,-1.42) |
| France | male | 12856.43(14058.68,11761.83) | 12296.88(14651.17,10327.02) | -0.04 | 42.34(46.51,38.44) | 18.92(22.46,16.03) | -3.12(-3.37,-2.87) |
| Gabon | male | 157.34(195.01,119.93) | 163.69(238.24,101.69) | 0.04 | 78.01(94.74,59.79) | 46.07(65.47,28.85) | -1.85(-1.91,-1.80) |
| Georgia | male | 640.62(723.72,565.23) | 706.54(872.70,563.68) | 0.10 | 34.40(38.37,30.63) | 30.90(37.94,24.73) | 1.09(0.41,1.77) |
| Germany | male | 24671.34(26072.04,23266.56) | 25592.25(29319.40,22353.34) | 0.04 | 56.11(59.34,52.72) | 28.53(32.49,25.07) | -2.39(-2.87,-1.90) |
| Ghana | male | 1997.78(2538.52,1078.34) | 4219.30(5412.75,2157.53) | 1.11 | 80.34(102.54,43.36) | 71.87(90.13,36.13) | 0.18(-0.05,0.41) |
| Greece | male | 1478.60(1607.79,1349.35) | 3419.93(3944.94,2856.69) | 1.31 | 23.55(25.56,21.60) | 25.46(29.04,21.56) | 0.96(0.61,1.32) |
| Greenland | male | 9.07(10.43,7.57) | 14.63(17.31,11.58) | 0.61 | 80.29(90.88,65.49) | 52.10(60.94,40.81) | -1.49(-1.57,-1.41) |
| Grenada | male | 10.98(12.61,9.41) | 11.12(13.09,9.25) | 0.01 | 34.98(40.35,29.98) | 27.38(32.08,23.08) | -0.74(-1.52,0.04) |
| Guam | male | 19.28(22.20,16.62) | 31.64(38.77,25.69) | 0.64 | 78.27(89.47,67.03) | 39.16(47.97,31.95) | -2.46(-2.73,-2.20) |
| Guatemala | male | 884.32(1009.31,767.15) | 1259.11(1598.24,991.70) | 0.42 | 49.56(57.37,42.32) | 32.95(40.64,26.62) | -1.77(-2.08,-1.46) |
| Guinea | male | 1169.60(1561.68,871.70) | 1569.66(1988.25,1129.79) | 0.34 | 76.72(105.67,56.17) | 61.71(77.93,44.92) | -0.30(-0.48,-0.12) |
| Guinea-Bissau | male | 236.27(299.68,176.93) | 220.37(289.13,153.31) | -0.07 | 129.13(159.57,95.91) | 77.40(98.46,55.39) | -1.47(-1.68,-1.26) |
| Guyana | male | 59.06(70.65,48.91) | 76.40(96.34,57.74) | 0.29 | 36.57(43.47,30.63) | 31.23(38.97,23.98) | -0.60(-0.78,-0.43) |
| Haiti | male | 1400.46(1856.60,788.45) | 1952.24(2740.98,1092.67) | 0.39 | 91.95(119.82,51.92) | 67.68(95.14,37.89) | -0.89(-0.98,-0.80) |
| Honduras | male | 634.88(761.89,497.08) | 1805.03(2577.70,1118.84) | 1.84 | 63.04(77.85,45.99) | 75.06(107.61,44.93) | 0.89(0.64,1.14) |
| Hungary | male | 3408.53(3608.08,3227.76) | 3242.74(3956.72,2656.23) | -0.05 | 65.16(68.79,61.57) | 43.50(52.73,35.68) | -1.05(-1.46,-0.63) |
| Iceland | male | 33.28(37.12,29.62) | 52.85(61.39,44.99) | 0.59 | 26.18(29.18,23.18) | 19.54(22.65,16.66) | -1.20(-1.36,-1.04) |
| India | male | 349606.45(405699.97,275001.42) | 616328.33(753429.18,473986.41) | 0.76 | 218.69(255.75,168.59) | 138.48(168.15,105.78) | -1.62(-1.76,-1.47) |
| Indonesia | male | 38541.69(44541.21,31497.57) | 65023.02(77307.02,52994.75) | 0.69 | 108.93(126.48,88.90) | 90.08(105.17,74.43) | -0.51(-0.62,-0.39) |
| Iran (Islamic Republic of) | male | 4845.12(6048.27,4272.34) | 10071.03(10864.80,8774.83) | 1.08 | 49.39(62.69,42.91) | 31.42(33.98,27.59) | -1.50(-1.56,-1.45) |
| Iraq | male | 1322.79(1664.60,1035.40) | 1991.63(2445.41,1559.73) | 0.51 | 37.97(47.24,29.75) | 22.87(27.92,18.16) | -2.34(-2.57,-2.10) |
| Ireland | male | 1396.17(1479.83,1314.14) | 1346.31(1541.72,1159.12) | -0.04 | 85.27(89.98,79.78) | 40.46(46.33,34.82) | -3.08(-3.37,-2.79) |
| Israel | male | 749.16(807.33,698.00) | 1060.25(1216.69,920.91) | 0.42 | 36.12(38.79,33.45) | 19.85(22.74,17.25) | -2.20(-2.36,-2.03) |
| Italy | male | 16557.20(17037.44,15817.59) | 18447.14(19825.40,16424.94) | 0.11 | 49.45(51.21,46.37) | 26.31(28.26,23.45) | -2.27(-2.34,-2.19) |
| Jamaica | male | 221.38(247.25,198.95) | 471.02(590.78,359.24) | 1.13 | 26.68(29.81,23.98) | 33.74(42.18,25.78) | 0.60(-0.06,1.27) |
| Japan | male | 18960.68(19554.59,17934.85) | 35886.17(38800.37,30689.49) | 0.89 | 32.08(33.28,29.90) | 18.93(20.39,16.42) | -1.99(-2.09,-1.89) |
| Jordan | male | 224.42(278.21,178.95) | 561.63(709.21,434.92) | 1.50 | 44.65(55.10,35.63) | 22.03(27.92,16.97) | -2.94(-3.15,-2.74) |
| Kazakhstan | male | 4011.67(4332.57,3688.30) | 5810.77(7083.02,4820.68) | 0.45 | 104.01(112.32,95.94) | 112.12(136.87,95.17) | -0.32(-0.99,0.36) |
| Kenya | male | 1876.36(3167.35,1238.02) | 4270.17(5623.06,3096.44) | 1.28 | 55.92(96.49,36.12) | 56.28(73.57,41.10) | 0.24(-0.04,0.52) |
| Kiribati | male | 32.01(38.60,25.94) | 37.61(47.84,28.83) | 0.17 | 237.82(281.81,193.22) | 178.16(218.62,139.88) | -1.02(-1.07,-0.97) |
| Kuwait | male | 47.56(54.81,40.80) | 161.74(203.74,125.14) | 2.40 | 19.10(22.12,15.93) | 14.76(18.67,11.23) | -0.84(-1.13,-0.55) |
| Kyrgyzstan | male | 1426.97(1553.74,1304.99) | 852.92(995.50,725.81) | -0.40 | 141.87(153.91,129.19) | 58.60(68.26,50.34) | -3.84(-4.57,-3.11) |
| Lao People's Democratic Republic | male | 1536.65(1920.66,1128.09) | 1693.16(2170.00,1264.87) | 0.10 | 183.27(228.09,136.49) | 107.72(135.04,81.71) | -2.06(-2.13,-1.98) |
| Latvia | male | 472.05(504.52,442.76) | 232.60(301.32,180.71) | -0.51 | 40.18(42.95,37.51) | 16.87(21.69,13.18) | -2.92(-3.30,-2.55) |
| Lebanon | male | 392.88(523.54,296.10) | 711.45(896.13,475.22) | 0.81 | 44.87(60.80,34.05) | 31.49(39.50,21.02) | -0.92(-1.09,-0.75) |
| Lesotho | male | 543.55(788.45,354.36) | 607.42(818.10,429.90) | 0.12 | 162.57(228.62,107.17) | 147.57(193.23,108.06) | -0.18(-0.37,0.02) |
| Liberia | male | 224.11(271.63,177.08) | 256.23(339.18,183.62) | 0.14 | 40.89(49.31,32.61) | 29.17(38.36,20.87) | -1.24(-1.38,-1.09) |
| Libya | male | 337.95(479.68,254.74) | 717.62(951.28,511.80) | 1.12 | 39.82(57.57,29.86) | 32.57(43.05,23.55) | -0.64(-0.83,-0.45) |
| Lithuania | male | 988.58(1047.34,929.57) | 479.66(584.18,388.28) | -0.51 | 61.01(64.67,57.30) | 23.01(27.83,18.76) | -3.49(-3.69,-3.28) |
| Luxembourg | male | 113.72(123.83,103.99) | 117.56(142.56,97.19) | 0.03 | 58.75(63.82,53.65) | 26.66(32.22,22.01) | -3.02(-3.13,-2.92) |
| North Macedonia | male | 397.22(439.50,350.87) | 399.93(497.63,321.03) | 0.01 | 54.37(60.61,47.80) | 33.42(40.97,27.30) | -1.80(-1.99,-1.62) |
| Madagascar | male | 2448.45(2798.24,2131.91) | 3267.90(4211.05,2457.70) | 0.33 | 95.80(110.83,84.18) | 79.96(101.90,57.90) | -0.80(-0.99,-0.61) |
| Malawi | male | 978.58(1190.64,804.08) | 1380.66(1654.27,1112.91) | 0.41 | 62.97(77.55,51.96) | 54.23(63.72,44.55) | -0.63(-0.85,-0.42) |
| Malaysia | male | 3251.13(3996.06,2633.23) | 4888.33(6421.74,3778.04) | 0.50 | 87.11(107.62,68.13) | 45.14(59.31,35.02) | -3.63(-4.23,-3.03) |
| Maldives | male | 48.29(58.45,34.62) | 62.76(74.51,50.83) | 0.30 | 131.37(157.63,96.24) | 49.68(59.51,40.46) | -3.84(-4.05,-3.63) |
| Mali | male | 1581.25(1908.01,1201.23) | 2646.74(3445.21,1928.71) | 0.67 | 83.53(99.53,65.77) | 63.72(81.57,47.90) | -0.81(-0.97,-0.65) |
| Malta | male | 81.44(90.23,73.06) | 113.35(131.91,95.00) | 0.39 | 50.56(56.43,45.23) | 27.16(31.55,22.71) | -2.34(-2.45,-2.22) |
| Marshall Islands | male | 13.03(19.79,10.10) | 14.96(20.15,10.96) | 0.15 | 197.86(305.70,154.46) | 115.78(155.23,87.29) | -1.67(-1.84,-1.50) |
| Mauritania | male | 274.66(341.80,209.94) | 276.55(353.69,212.04) | 0.01 | 66.03(82.26,51.04) | 30.38(38.26,23.56) | -2.49(-2.75,-2.23) |
| Mauritius | male | 277.24(297.86,257.03) | 304.89(374.75,244.76) | 0.10 | 108.55(117.05,99.23) | 47.96(58.56,39.01) | -3.03(-3.21,-2.85) |
| Mexico | male | 9461.14(9826.55,8849.30) | 20551.05(24377.88,16918.98) | 1.17 | 56.44(58.94,51.56) | 43.09(51.00,35.52) | -1.19(-1.32,-1.06) |
| Republic of Moldova | male | 1145.43(1219.95,1074.73) | 594.43(695.05,506.71) | -0.48 | 74.38(79.64,69.53) | 27.35(31.84,23.50) | -4.03(-4.51,-3.55) |
| Mongolia | male | 293.55(354.64,231.05) | 263.18(358.04,204.64) | -0.10 | 76.31(90.10,61.10) | 38.10(48.71,30.70) | -3.40(-3.83,-2.97) |
| Montenegro | male | 35.47(40.82,29.33) | 56.94(68.42,45.79) | 0.61 | 16.42(18.92,13.15) | 15.55(18.48,12.26) | 0.00(-0.15,0.16) |
| Morocco | male | 2754.86(4046.93,2098.41) | 5551.95(7189.50,4032.95) | 1.02 | 51.31(80.46,38.68) | 45.54(59.77,33.65) | -0.67(-1.05,-0.28) |
| Mozambique | male | 1397.72(1835.63,1105.30) | 2368.14(2974.03,1840.32) | 0.69 | 59.32(78.51,47.10) | 60.69(76.41,47.65) | 0.20(-0.02,0.43) |
| Myanmar | male | 21783.07(27177.79,17101.18) | 25550.61(29575.28,21866.20) | 0.17 | 252.41(316.73,201.67) | 168.69(192.24,144.10) | -1.49(-1.57,-1.42) |
| Namibia | male | 418.32(527.06,303.48) | 483.08(592.47,385.52) | 0.15 | 150.66(186.24,109.89) | 100.27(120.68,81.29) | -1.55(-1.76,-1.33) |
| Nepal | male | 9815.18(11966.20,7107.29) | 19902.18(23692.11,14191.08) | 1.03 | 280.15(337.67,198.48) | 238.95(282.53,171.62) | -0.43(-0.62,-0.24) |
| Netherlands | male | 4781.61(5097.27,4420.20) | 5889.96(6717.21,5113.88) | 0.23 | 63.82(68.24,58.33) | 38.32(43.67,33.42) | -2.26(-2.49,-2.03) |
| New Zealand | male | 927.83(985.33,873.89) | 1275.31(1448.87,1101.48) | 0.37 | 59.50(63.17,55.42) | 34.09(38.69,29.46) | -2.48(-2.70,-2.27) |
| Nicaragua | male | 255.77(294.85,224.31) | 716.41(857.94,551.37) | 1.80 | 45.33(53.08,39.30) | 50.50(59.58,38.73) | 0.59(0.33,0.84) |
| Niger | male | 1127.96(1574.12,800.42) | 1882.52(2765.13,1212.01) | 0.67 | 87.58(130.90,61.57) | 57.48(83.87,37.78) | -1.13(-1.38,-0.87) |
| Nigeria | male | 9969.27(13111.15,6881.99) | 13900.66(18832.35,10047.27) | 0.39 | 56.08(73.17,39.56) | 41.13(54.92,29.83) | -0.90(-1.01,-0.79) |
| Democratic People's Republic of Korea | male | 8117.68(10411.66,5862.09) | 14586.47(17255.02,10866.26) | 0.80 | 223.59(276.70,170.55) | 159.86(188.84,121.13) | -1.17(-1.33,-1.02) |
| Northern Mariana Islands | male | 6.87(8.34,5.78) | 11.51(14.05,9.87) | 0.68 | 100.46(117.13,86.19) | 66.78(79.88,57.68) | -1.34(-1.39,-1.29) |
| Norway | male | 929.96(967.94,884.19) | 1436.79(1538.91,1300.15) | 0.54 | 31.51(32.83,29.80) | 31.56(33.80,28.57) | 0.07(-0.20,0.35) |
| Oman | male | 110.42(165.38,78.15) | 148.36(177.99,114.26) | 0.34 | 49.18(74.31,36.03) | 31.47(37.66,23.06) | -1.05(-1.34,-0.77) |
| Pakistan | male | 48001.90(61401.24,37225.69) | 57405.63(74211.36,43793.15) | 0.20 | 172.72(223.64,132.60) | 136.97(172.43,106.32) | -0.95(-1.22,-0.68) |
| Palestine | male | 204.29(303.23,155.64) | 297.40(347.64,246.05) | 0.46 | 60.96(91.73,47.11) | 39.67(46.53,33.17) | -1.64(-1.77,-1.52) |
| Panama | male | 198.17(220.78,177.67) | 438.09(562.30,331.51) | 1.21 | 28.05(31.44,24.77) | 22.41(28.78,16.96) | -1.47(-1.86,-1.08) |
| Papua New Guinea | male | 1752.46(2185.52,1348.99) | 3686.68(4696.34,2787.57) | 1.10 | 256.70(316.35,201.08) | 212.13(261.55,158.83) | -0.60(-0.65,-0.54) |
| Paraguay | male | 242.13(343.64,204.83) | 762.48(981.69,573.23) | 2.15 | 26.22(38.11,21.93) | 33.04(42.00,24.79) | 1.27(1.04,1.50) |
| Peru | male | 1858.77(2492.97,1558.54) | 3728.29(4881.82,2670.58) | 1.01 | 31.27(45.02,26.03) | 24.43(32.02,17.50) | 0.01(-0.30,0.32) |
| Philippines | male | 12282.29(14008.94,10574.42) | 22099.52(28262.68,17233.67) | 0.80 | 105.24(120.72,91.16) | 77.02(97.75,60.27) | -0.93(-1.08,-0.77) |
| Poland | male | 10420.61(10760.47,10048.86) | 7062.06(8724.58,5668.05) | -0.32 | 65.98(68.27,62.77) | 25.66(31.44,20.72) | -3.28(-3.43,-3.12) |
| Portugal | male | 2870.60(3082.36,2675.78) | 3892.34(4496.53,3336.89) | 0.36 | 58.40(63.04,54.07) | 34.87(39.98,30.08) | -1.98(-2.11,-1.84) |
| Puerto Rico | male | 526.03(579.44,476.87) | 901.45(1143.03,693.54) | 0.71 | 33.94(37.50,30.72) | 26.34(33.57,20.23) | -1.54(-1.87,-1.22) |
| Qatar | male | 12.63(19.07,9.34) | 46.69(69.32,34.26) | 2.70 | 35.32(55.10,26.32) | 21.64(32.21,16.31) | -1.73(-2.18,-1.27) |
| Romania | male | 8998.81(9569.23,8383.76) | 5193.47(6305.43,4232.82) | -0.42 | 85.98(91.34,79.47) | 33.45(40.41,27.24) | -3.40(-3.61,-3.19) |
| Russian Federation | male | 35201.72(37528.31,34248.30) | 24752.87(29574.90,20435.28) | -0.30 | 70.59(75.12,67.94) | 31.68(37.70,26.46) | -3.54(-3.90,-3.18) |
| Rwanda | male | 1528.73(1977.13,1070.10) | 1314.87(1707.94,960.25) | -0.14 | 129.04(163.52,91.53) | 69.50(88.95,50.75) | -2.86(-3.14,-2.57) |
| Saint Lucia | male | 19.79(22.48,17.61) | 41.44(49.77,33.13) | 1.09 | 61.65(69.54,54.87) | 47.64(56.84,38.52) | -1.49(-1.78,-1.20) |
| Saint Vincent and the Grenadines | male | 6.79(7.87,5.79) | 14.64(16.91,12.21) | 1.16 | 24.41(28.05,21.01) | 24.46(28.00,20.59) | -0.17(-0.34,0.00) |
| Samoa | male | 60.62(76.21,47.35) | 54.69(70.98,45.43) | -0.10 | 177.79(222.00,139.89) | 93.27(120.45,78.36) | -2.30(-2.45,-2.16) |
| Sao Tome and Principe | male | 32.70(38.89,26.32) | 45.06(60.49,32.86) | 0.38 | 128.95(149.94,105.84) | 107.95(144.82,79.09) | -0.46(-0.55,-0.38) |
| Saudi Arabia | male | 1369.81(2192.66,1003.70) | 2200.56(2707.12,1716.46) | 0.61 | 55.27(89.03,40.91) | 31.98(38.48,25.38) | -1.97(-2.09,-1.86) |
| Senegal | male | 1208.93(1499.89,890.76) | 1582.15(2036.56,1203.93) | 0.31 | 83.25(103.92,61.82) | 51.03(64.24,39.60) | -1.34(-1.61,-1.07) |
| Serbia | male | 2167.83(2618.77,1846.72) | 2381.92(2938.63,1916.06) | 0.10 | 48.53(59.12,41.66) | 35.41(43.52,28.77) | -1.12(-1.31,-0.93) |
| Seychelles | male | 16.53(18.51,13.77) | 18.48(21.88,15.61) | 0.12 | 77.00(85.93,64.31) | 47.52(55.60,39.95) | -2.04(-2.26,-1.83) |
| Sierra Leone | male | 737.52(906.03,545.44) | 804.93(1092.50,556.94) | 0.09 | 79.90(97.68,59.49) | 50.74(67.65,35.35) | -1.21(-1.40,-1.01) |
| Singapore | male | 680.26(722.11,638.71) | 471.39(544.62,404.37) | -0.31 | 96.87(102.80,89.81) | 15.57(17.86,13.29) | -6.64(-6.95,-6.33) |
| Slovakia | male | 694.47(999.14,626.33) | 720.12(917.93,559.26) | 0.04 | 30.70(44.03,27.62) | 20.91(26.42,16.41) | -0.83(-1.05,-0.61) |
| Slovenia | male | 475.34(593.69,368.03) | 396.13(510.31,304.48) | -0.17 | 56.47(70.13,44.56) | 21.98(28.05,17.13) | -4.14(-4.54,-3.75) |
| Solomon Islands | male | 123.45(163.84,93.69) | 215.65(260.79,171.60) | 0.75 | 202.07(273.52,158.05) | 179.15(209.09,146.03) | -0.35(-0.41,-0.28) |
| Somalia | male | 1221.25(1905.22,785.50) | 2093.57(3422.19,1318.22) | 0.71 | 119.64(196.69,81.06) | 90.60(151.26,59.12) | -0.93(-0.98,-0.89) |
| South Africa | male | 6645.62(7814.61,5872.84) | 9927.63(10932.10,9100.70) | 0.49 | 86.10(102.95,75.81) | 68.01(74.96,61.81) | -1.22(-1.88,-0.56) |
| Republic of Korea | male | 5815.02(6230.73,5059.26) | 9010.29(10074.86,7639.88) | 0.55 | 90.78(98.50,76.84) | 31.24(35.06,25.50) | -4.37(-4.72,-4.03) |
| South Sudan | male | 932.51(1203.83,614.90) | 899.45(1228.53,593.85) | -0.04 | 80.18(104.70,55.07) | 55.03(74.36,36.74) | -1.36(-1.47,-1.25) |
| Spain | male | 15139.15(15955.36,14104.19) | 21824.37(24963.05,18455.39) | 0.44 | 73.33(77.61,67.48) | 46.57(52.85,39.67) | -1.83(-1.99,-1.66) |
| Sri Lanka | male | 5741.39(6341.59,5062.06) | 7366.03(9696.61,5154.29) | 0.28 | 134.77(149.09,119.04) | 84.48(109.44,59.76) | -0.83(-1.15,-0.52) |
| Sudan | male | 3838.88(5513.22,2092.60) | 4666.84(6596.21,2815.51) | 0.22 | 88.54(128.75,48.09) | 55.03(76.80,33.79) | -1.74(-1.87,-1.61) |
| Suriname | male | 35.35(40.92,31.03) | 67.61(82.27,54.98) | 0.91 | 32.34(36.88,28.38) | 28.13(34.25,22.99) | -0.76(-1.11,-0.40) |
| Eswatini | male | 152.07(190.24,110.86) | 199.46(253.81,155.79) | 0.31 | 152.33(191.76,111.68) | 111.33(136.20,88.85) | -0.91(-1.21,-0.61) |
| Sweden | male | 1464.33(1552.70,1367.78) | 2159.60(2386.61,1916.38) | 0.47 | 22.43(23.79,20.92) | 20.01(22.08,17.82) | -0.52(-0.66,-0.38) |
| Switzerland | male | 1693.05(1831.86,1535.39) | 1726.73(2007.41,1471.41) | 0.02 | 40.87(44.39,36.92) | 20.54(23.82,17.63) | -2.60(-2.73,-2.48) |
| Syrian Arab Republic | male | 1257.72(1586.53,995.62) | 1908.84(2606.67,1381.88) | 0.52 | 48.89(64.44,38.17) | 37.48(52.35,27.98) | -1.20(-1.37,-1.03) |
| Taiwan (Province of China) | male | 3368.33(3618.66,3100.40) | 6790.43(8459.94,5391.75) | 1.02 | 58.77(63.46,53.87) | 37.49(46.81,29.72) | -1.78(-1.98,-1.59) |
| Tajikistan | male | 861.86(949.33,773.59) | 919.31(1251.57,737.84) | 0.07 | 76.77(84.47,68.25) | 59.64(84.14,47.22) | -0.78(-1.07,-0.49) |
| United Republic of Tanzania | male | 2395.76(2819.24,1971.42) | 3845.39(4626.59,3025.25) | 0.61 | 52.83(62.18,43.63) | 39.74(47.31,30.71) | -1.01(-1.07,-0.96) |
| Thailand | male | 12275.57(14347.43,10038.42) | 15888.02(20415.72,11811.91) | 0.29 | 96.47(112.83,80.07) | 38.54(49.33,28.77) | -3.55(-3.78,-3.32) |
| Bahamas | male | 14.40(16.75,12.39) | 31.59(39.66,24.68) | 1.19 | 25.88(30.06,22.12) | 21.55(26.86,16.88) | -0.78(-0.90,-0.66) |
| Gambia | male | 120.33(158.34,84.95) | 238.98(311.46,178.30) | 0.99 | 79.16(102.44,58.59) | 59.70(76.30,44.74) | -0.87(-1.07,-0.67) |
| Timor-Leste | male | 126.49(169.49,84.03) | 327.30(438.73,230.54) | 1.59 | 112.68(157.71,76.62) | 100.53(132.57,72.55) | -0.51(-0.71,-0.31) |
| Togo | male | 411.62(500.21,313.55) | 722.45(971.90,499.19) | 0.76 | 79.99(96.56,60.88) | 57.89(75.54,41.63) | -0.87(-0.99,-0.75) |
| Tonga | male | 25.55(30.28,20.70) | 28.31(34.11,22.94) | 0.11 | 124.51(147.74,101.04) | 89.37(106.96,73.06) | -1.28(-1.53,-1.02) |
| Trinidad and Tobago | male | 121.68(131.31,112.92) | 174.74(228.81,128.49) | 0.44 | 35.88(38.69,33.01) | 22.16(28.85,16.47) | -1.79(-1.96,-1.62) |
| Tunisia | male | 843.11(1160.98,655.02) | 1618.53(2207.95,1139.26) | 0.92 | 42.33(57.77,32.93) | 31.67(42.99,22.56) | -1.09(-1.15,-1.02) |
| Turkey | male | 11602.27(13768.25,9359.08) | 20387.22(25158.24,14040.79) | 0.76 | 80.35(95.82,64.62) | 56.00(68.90,38.88) | -0.51(-0.84,-0.17) |
| Turkmenistan | male | 431.41(461.61,404.13) | 239.78(307.52,187.97) | -0.44 | 65.86(70.97,60.85) | 18.06(22.70,14.48) | -5.46(-5.98,-4.94) |
| Uganda | male | 2615.65(3990.19,1735.44) | 3256.92(4265.18,2224.52) | 0.25 | 94.39(146.41,62.01) | 66.23(86.08,45.95) | -1.58(-1.73,-1.44) |
| Ukraine | male | 19934.02(21169.46,18689.63) | 7788.47(9600.63,6178.47) | -0.61 | 92.87(98.81,86.68) | 29.40(35.89,23.48) | -5.67(-6.24,-5.09) |
| United Arab Emirates | male | 244.34(347.83,172.76) | 1570.60(2137.03,1043.31) | 5.43 | 83.45(124.91,65.34) | 50.56(68.13,39.05) | -2.05(-2.37,-1.73) |
| United Kingdom | male | 22755.98(23246.90,21993.65) | 26866.67(28198.49,24832.72) | 0.18 | 65.57(67.24,62.67) | 44.53(46.73,41.16) | -1.37(-1.53,-1.21) |
| United States of America | male | 59390.76(60990.19,56950.19) | 112521.05(117707.85,104079.43) | 0.89 | 45.19(46.50,43.12) | 44.25(46.33,40.84) | -0.23(-0.33,-0.12) |
| Uruguay | male | 889.14(972.61,810.69) | 1275.47(1425.76,1119.62) | 0.43 | 55.96(61.08,51.10) | 55.36(61.80,48.64) | -0.27(-0.62,0.07) |
| Uzbekistan | male | 2596.71(2821.58,2376.86) | 2053.26(2570.92,1640.55) | -0.21 | 62.65(67.95,56.81) | 34.74(41.51,28.73) | -3.21(-3.86,-2.56) |
| Vanuatu | male | 58.98(78.29,38.77) | 108.02(141.87,76.86) | 0.83 | 198.09(262.34,134.40) | 145.38(188.07,105.30) | -1.26(-1.36,-1.17) |
| Venezuela (Bolivarian Republic of) | male | 1076.07(1179.95,972.56) | 3756.12(4850.45,2817.18) | 2.49 | 26.80(29.54,23.95) | 31.72(40.98,24.05) | -0.06(-0.50,0.38) |
| Viet nam | male | 14878.42(19219.90,5365.42) | 22434.23(28470.78,7492.90) | 0.51 | 113.65(146.57,40.83) | 79.79(100.61,26.63) | -1.20(-1.24,-1.17) |
| Virginia | male | 1320.61(1416.63,1234.03) | 2641.08(3320.57,2041.76) | 1.00 | 49.00(52.64,45.55) | 42.63(53.28,33.13) | -0.85(-1.05,-0.66) |
| Yemen | male | 1783.37(2523.77,1005.54) | 3216.25(4326.42,2376.50) | 0.80 | 99.62(148.19,55.85) | 61.59(81.40,46.14) | -1.79(-1.88,-1.70) |
| Zambia | male | 897.12(1137.20,723.83) | 1420.68(1716.78,1124.74) | 0.58 | 68.77(88.59,54.47) | 56.59(67.41,45.51) | -0.83(-1.08,-0.58) |
| Zimbabwe | male | 1070.05(1274.80,893.56) | 1547.62(1900.78,1225.25) | 0.45 | 69.80(82.59,59.08) | 64.60(78.74,51.63) | -0.33(-0.52,-0.14) |
| Monaco | male | 9.40(11.36,7.59) | 11.04(13.17,9.10) | 0.17 | 30.56(36.66,24.77) | 22.60(26.89,18.63) | -0.95(-0.98,-0.93) |
| San Marino | male | 2.77(3.33,2.29) | 5.16(6.94,3.59) | 0.86 | 20.77(24.94,17.16) | 15.76(21.41,10.94) | -0.85(-1.03,-0.67) |
| Saint Kitts and Nevis | male | 4.62(5.49,3.92) | 6.46(7.90,5.27) | 0.40 | 31.26(37.04,26.64) | 27.15(32.20,22.75) | -0.42(-0.56,-0.27) |
| Cook Islands | male | 3.98(4.57,3.36) | 3.70(4.40,3.11) | -0.07 | 74.82(86.24,62.89) | 33.92(40.34,28.46) | -2.99(-3.17,-2.81) |
| Nauru | male | 2.88(3.62,2.20) | 1.71(2.30,1.26) | -0.41 | 182.87(224.34,144.66) | 120.57(155.95,92.97) | -1.50(-1.77,-1.23) |
| Niue | male | 1.10(1.37,0.89) | 0.66(0.84,0.54) | -0.40 | 126.75(158.20,103.50) | 76.31(97.30,62.31) | -1.95(-2.04,-1.86) |
| Palau | male | 7.57(9.28,6.22) | 10.38(12.95,8.33) | 0.37 | 192.51(234.61,159.03) | 135.77(165.09,111.49) | -1.15(-1.23,-1.07) |
| Tokelau | male | 0.74(0.95,0.57) | 0.35(0.43,0.29) | -0.52 | 123.65(160.05,95.65) | 60.28(74.37,49.36) | -2.56(-2.60,-2.52) |
| Tuvalu | male | 4.80(5.97,3.62) | 3.85(5.02,3.02) | -0.20 | 209.29(261.43,156.62) | 98.29(126.75,77.14) | -2.68(-2.81,-2.55) |
| Afghanistan | female | 2661.53(3412.45,1954.46) | 3790.87(4919.92,2734.52) | 0.42 | 87.89(113.75,63.72) | 69.05(89.19,48.30) | -1.05(-1.34,-0.76) |
| Albania | female | 359.86(410.33,203.94) | 279.11(387.72,186.32) | -0.22 | 35.58(40.81,19.89) | 12.06(16.67,8.09) | -4.15(-4.48,-3.81) |
| Algeria | female | 1832.93(2490.00,1100.97) | 3159.61(4137.38,2419.01) | 0.72 | 44.54(61.01,26.36) | 27.24(35.86,19.89) | -1.48(-1.59,-1.37) |
| American Samoa | female | 5.94(7.57,4.65) | 9.35(12.43,7.07) | 0.58 | 67.89(86.22,52.52) | 44.20(58.77,33.22) | -1.67(-1.79,-1.55) |
| Andorra | female | 3.56(5.18,2.40) | 12.69(16.70,9.03) | 2.57 | 16.91(23.68,11.45) | 14.68(19.41,10.43) | -0.49(-0.76,-0.22) |
| Angola | female | 1446.51(2241.44,863.42) | 2162.48(3036.80,1321.06) | 0.49 | 81.12(148.14,46.99) | 45.97(68.04,28.11) | -2.20(-2.42,-1.98) |
| Antigua and Barbuda | female | 2.53(3.04,2.04) | 4.57(5.64,3.35) | 0.81 | 7.13(8.49,5.88) | 9.26(11.41,6.72) | 0.63(0.47,0.78) |
| Argentina | female | 3641.10(5024.09,3177.60) | 8771.91(10305.95,6938.02) | 1.41 | 21.22(29.43,18.39) | 25.82(30.04,20.63) | 0.54(0.30,0.78) |
| Armenia | female | 386.55(439.86,218.37) | 604.29(768.23,322.83) | 0.56 | 29.86(34.20,16.56) | 25.40(32.25,13.57) | -0.31(-0.57,-0.04) |
| Australia | female | 2464.67(2840.96,2181.23) | 4994.45(5994.68,3454.98) | 1.03 | 22.01(25.20,19.40) | 19.27(22.72,13.51) | -0.68(-0.85,-0.51) |
| Austria | female | 1020.43(1363.21,887.21) | 1472.51(1741.08,1106.19) | 0.44 | 12.40(16.47,10.87) | 12.07(14.04,8.79) | 0.09(-0.08,0.25) |
| Azerbaijan | female | 885.62(1088.60,740.03) | 860.70(1509.33,548.32) | -0.03 | 30.69(38.15,25.83) | 25.46(45.25,14.80) | -1.21(-1.59,-0.83) |
| Bahrain | female | 34.95(41.91,27.78) | 68.54(95.37,53.72) | 0.96 | 63.85(76.68,48.57) | 33.83(45.23,26.81) | -2.50(-2.99,-2.02) |
| Bangladesh | female | 18269.61(29521.22,13610.87) | 26252.83(51657.85,18175.28) | 0.44 | 99.84(171.91,72.90) | 48.85(98.85,33.56) | -2.52(-2.85,-2.20) |
| Barbados | female | 15.96(19.51,13.17) | 25.44(32.12,19.39) | 0.59 | 8.82(10.73,7.34) | 9.31(11.69,7.09) | 0.38(0.23,0.53) |
| Belarus | female | 2938.94(3457.02,1282.05) | 960.12(2112.33,634.67) | -0.67 | 34.95(41.14,15.17) | 8.30(18.58,5.51) | -6.10(-6.69,-5.51) |
| Belgium | female | 2037.66(2498.22,1757.90) | 3054.53(3652.39,2320.47) | 0.50 | 20.56(24.74,17.80) | 18.91(22.17,14.18) | -0.47(-0.59,-0.35) |
| Belize | female | 8.28(10.37,6.83) | 21.21(25.83,16.90) | 1.56 | 16.09(20.78,13.21) | 16.15(19.81,12.79) | 0.01(-0.30,0.32) |
| Benin | female | 547.74(700.02,411.44) | 842.43(1168.49,621.73) | 0.54 | 53.53(69.10,40.53) | 34.36(46.98,25.72) | -1.30(-1.47,-1.12) |
| Bermuda | female | 4.03(4.92,3.31) | 6.17(8.02,4.54) | 0.53 | 11.87(14.53,9.71) | 7.37(9.49,5.52) | -2.11(-2.34,-1.88) |
| Bhutan | female | 161.50(220.59,109.28) | 300.07(387.16,222.10) | 0.86 | 160.48(221.95,108.31) | 131.78(168.94,97.46) | -0.77(-0.83,-0.72) |
| Bolivia (Plurinational State of) | female | 908.99(1152.74,648.84) | 1773.36(2259.31,1276.85) | 0.95 | 55.03(67.98,42.67) | 47.17(59.95,33.64) | -0.46(-0.54,-0.38) |
| Bosnia and Herzegovina | female | 493.16(552.13,357.64) | 457.49(595.71,327.59) | -0.07 | 26.94(30.45,19.33) | 13.69(17.88,9.87) | -2.74(-2.97,-2.51) |
| Botswana | female | 177.50(307.40,107.56) | 259.99(372.37,164.23) | 0.46 | 64.91(111.34,40.10) | 40.35(56.49,25.32) | -1.71(-1.87,-1.55) |
| Brazil | female | 16375.60(17866.71,13621.95) | 35757.78(43428.99,30581.34) | 1.18 | 41.92(46.32,34.87) | 27.71(33.63,23.71) | -1.79(-1.96,-1.62) |
| Brunei Darussalam | female | 29.60(35.47,16.73) | 42.00(49.54,35.06) | 0.42 | 93.22(112.19,49.33) | 49.00(57.00,38.45) | -2.82(-3.26,-2.38) |
| Bulgaria | female | 1266.25(1411.79,943.67) | 1183.12(1665.65,886.56) | -0.07 | 22.27(24.99,16.49) | 13.12(18.28,9.87) | -2.41(-2.68,-2.14) |
| Burkina Faso | female | 775.69(978.02,582.43) | 1301.76(1704.16,998.35) | 0.68 | 35.43(45.79,26.77) | 26.21(34.30,20.73) | -1.11(-1.25,-0.97) |
| Burundi | female | 967.94(1288.01,531.14) | 981.48(1531.01,655.57) | 0.01 | 70.58(94.75,40.72) | 47.35(71.23,32.71) | -1.64(-1.75,-1.54) |
| Cambodia | female | 1073.25(1336.83,753.55) | 1986.46(2443.18,1410.94) | 0.85 | 49.71(61.53,34.44) | 36.12(44.40,25.27) | -1.24(-1.30,-1.17) |
| Cameroon | female | 1098.15(1370.33,807.00) | 1732.59(2402.21,1159.71) | 0.58 | 53.89(67.59,39.52) | 31.52(42.91,21.57) | -1.62(-1.71,-1.53) |
| Canada | female | 3379.44(3936.35,2941.98) | 8537.39(10423.91,5204.35) | 1.53 | 17.60(20.57,15.33) | 19.62(23.59,12.62) | 0.03(-0.19,0.25) |
| Cabo Verde | female | 61.02(73.40,35.39) | 50.52(73.10,32.93) | -0.17 | 43.84(52.53,25.45) | 20.08(28.95,13.29) | -2.56(-3.19,-1.93) |
| Central African Republic | female | 514.89(822.53,293.15) | 788.59(1415.72,428.09) | 0.53 | 96.72(174.66,51.49) | 83.61(164.20,42.78) | -0.48(-0.56,-0.40) |
| Chad | female | 833.87(1143.86,589.47) | 1095.46(1457.28,800.41) | 0.31 | 59.00(82.13,41.18) | 43.69(59.24,31.89) | -0.82(-0.93,-0.71) |
| Chile | female | 1150.93(1267.87,838.71) | 3128.34(3770.34,1784.87) | 1.72 | 24.43(27.10,17.62) | 22.52(27.00,13.03) | 0.04(-0.21,0.29) |
| China | female | 636167.96(736502.92,417646.82) | 456957.96(619555.74,357118.31) | -0.28 | 198.17(228.23,132.67) | 50.09(67.86,38.99) | -5.18(-5.41,-4.94) |
| Colombia | female | 2721.87(2994.15,2048.51) | 8694.42(11282.28,5629.54) | 2.19 | 35.09(38.99,26.66) | 27.43(35.74,17.94) | -1.12(-1.26,-0.98) |
| Comoros | female | 49.71(68.82,27.52) | 73.23(95.86,53.19) | 0.47 | 43.76(58.64,25.57) | 29.49(38.42,21.36) | -1.50(-1.61,-1.38) |
| Congo | female | 388.72(647.21,215.74) | 509.18(750.27,293.79) | 0.31 | 80.57(146.59,44.23) | 49.11(77.42,27.95) | -1.74(-1.86,-1.61) |
| Costa Rica | female | 230.48(266.90,189.83) | 690.20(873.45,476.29) | 1.99 | 27.28(31.61,22.35) | 23.39(29.62,16.22) | -1.24(-1.75,-0.73) |
| Côte d'Ivoire | female | 741.53(969.58,535.40) | 1308.36(1754.92,965.67) | 0.76 | 46.05(59.47,34.41) | 29.51(39.38,22.51) | -1.07(-1.26,-0.87) |
| Croatia | female | 451.81(565.43,392.41) | 737.15(942.08,501.96) | 0.63 | 12.50(15.79,10.79) | 12.09(15.44,8.47) | 0.32(0.17,0.48) |
| Cuba | female | 814.63(907.37,688.44) | 2032.67(2587.61,1210.80) | 1.50 | 15.92(17.83,13.55) | 19.22(24.49,11.35) | 0.66(0.52,0.79) |
| Cyprus | female | 146.50(181.34,91.98) | 229.69(276.96,132.40) | 0.57 | 50.85(63.45,29.16) | 25.44(30.78,13.48) | -2.58(-2.98,-2.18) |
| Czechia | female | 1029.91(1556.57,901.69) | 1609.92(2010.81,1186.34) | 0.56 | 12.37(18.31,10.92) | 12.11(15.14,8.82) | 0.83(0.43,1.24) |
| Democratic Republic of the Congo | female | 5632.45(9434.13,3057.43) | 11467.78(22390.92,6146.16) | 1.04 | 83.66(154.55,42.81) | 71.60(147.08,37.28) | -0.45(-0.66,-0.24) |
| Denmark | female | 1366.09(1534.21,1189.89) | 2304.77(2780.81,1382.74) | 0.69 | 28.08(31.02,23.15) | 32.06(38.45,18.87) | 0.01(-0.38,0.40) |
| Djibouti | female | 24.44(38.80,12.23) | 53.52(83.96,18.31) | 1.19 | 35.68(52.36,17.83) | 23.53(35.88,7.51) | -1.55(-1.60,-1.50) |
| Dominica | female | 6.38(8.57,5.37) | 6.85(8.61,4.30) | 0.07 | 14.29(19.03,12.07) | 13.95(17.57,8.74) | 0.16(0.04,0.27) |
| Dominican Republic | female | 420.13(488.89,328.12) | 853.26(1180.55,552.60) | 1.03 | 22.07(25.56,17.11) | 18.43(25.22,11.89) | 0.09(-0.33,0.51) |
| Ecuador | female | 767.62(861.05,564.31) | 1605.95(1964.22,1113.70) | 1.09 | 31.71(35.16,24.69) | 24.24(29.81,16.53) | -0.15(-0.61,0.32) |
| Egypt | female | 6846.73(8049.49,5816.17) | 8708.25(12942.34,5855.22) | 0.27 | 53.04(67.15,45.28) | 42.48(62.01,29.35) | -0.42(-0.53,-0.30) |
| El Salvador | female | 527.35(599.91,412.82) | 841.24(1085.74,576.51) | 0.60 | 31.55(36.22,25.62) | 21.61(28.01,15.05) | -1.30(-1.59,-1.00) |
| Equatorial Guinea | female | 78.05(134.36,43.63) | 81.39(196.55,42.90) | 0.04 | 80.74(156.43,42.36) | 38.30(97.99,19.96) | -2.93(-3.12,-2.74) |
| Eritrea | female | 323.09(474.77,187.59) | 545.58(762.27,308.77) | 0.69 | 54.58(82.06,33.59) | 41.45(57.16,22.87) | -0.89(-0.94,-0.85) |
| Estonia | female | 126.77(161.26,113.03) | 134.52(185.02,102.38) | 0.06 | 9.54(11.92,8.53) | 6.44(8.87,4.92) | -1.44(-1.59,-1.30) |
| Ethiopia | female | 5478.82(7051.82,3383.80) | 5330.44(6841.00,4173.56) | -0.03 | 55.48(74.22,36.72) | 29.01(36.55,22.41) | -2.58(-2.75,-2.40) |
| Micronesia (Federated States of) | female | 28.57(37.87,20.46) | 26.29(36.79,15.91) | -0.08 | 142.03(195.07,100.90) | 94.39(127.63,59.65) | -1.50(-1.64,-1.36) |
| Fiji | female | 146.70(209.05,106.68) | 155.77(211.20,112.30) | 0.06 | 99.30(141.74,72.24) | 50.62(67.90,37.03) | -2.74(-2.97,-2.51) |
| Finland | female | 384.75(530.31,334.83) | 735.49(875.16,535.57) | 0.91 | 8.25(11.11,7.20) | 8.92(10.53,6.16) | 0.37(0.26,0.48) |
| France | female | 9193.45(10521.44,7129.20) | 9763.60(13196.30,7301.85) | 0.06 | 15.66(17.75,12.27) | 8.68(12.19,6.73) | -2.40(-2.65,-2.14) |
| Gabon | female | 148.48(211.30,80.23) | 144.49(195.39,73.90) | -0.03 | 53.55(78.45,29.53) | 30.54(40.88,15.91) | -1.97(-2.08,-1.87) |
| Georgia | female | 555.72(927.75,460.30) | 515.01(912.58,367.76) | -0.07 | 16.64(27.43,13.87) | 12.20(21.00,8.93) | 0.12(-0.44,0.69) |
| Germany | female | 15216.49(20570.76,13355.14) | 19573.24(23797.33,15922.66) | 0.29 | 17.62(23.30,15.60) | 15.20(17.93,12.42) | -0.37(-0.72,-0.03) |
| Ghana | female | 1077.75(1367.71,730.43) | 1718.72(2199.70,1178.51) | 0.59 | 35.59(46.14,25.25) | 21.77(27.54,14.94) | -1.70(-1.74,-1.66) |
| Greece | female | 1148.11(1327.25,977.77) | 2979.40(3756.78,1636.84) | 1.60 | 14.22(16.46,11.90) | 16.44(20.36,9.60) | 1.25(0.72,1.77) |
| Greenland | female | 14.71(17.34,10.93) | 17.37(21.44,11.44) | 0.18 | 103.66(121.82,74.64) | 61.29(75.61,39.34) | -2.58(-2.97,-2.20) |
| Grenada | female | 6.77(8.47,5.61) | 7.93(9.54,6.31) | 0.17 | 14.31(17.61,11.91) | 14.13(17.05,11.30) | 0.08(-0.15,0.31) |
| Guam | female | 11.20(13.30,9.10) | 21.40(27.41,16.69) | 0.91 | 45.45(55.65,34.84) | 20.77(26.67,16.09) | -3.19(-3.63,-2.75) |
| Guatemala | female | 742.79(875.06,537.69) | 1270.01(1803.84,978.51) | 0.71 | 42.36(50.47,35.68) | 23.99(33.52,18.53) | -2.29(-2.59,-1.98) |
| Guinea | female | 1045.10(1331.70,771.36) | 1217.66(1651.22,872.70) | 0.17 | 65.44(83.82,47.12) | 45.12(60.82,32.49) | -0.98(-1.18,-0.78) |
| Guinea-Bissau | female | 133.77(173.40,93.93) | 165.28(220.55,122.14) | 0.24 | 66.70(86.61,46.98) | 46.07(62.34,34.03) | -0.99(-1.18,-0.79) |
| Guyana | female | 31.19(38.19,25.49) | 42.17(55.86,31.27) | 0.35 | 16.24(20.16,13.25) | 14.00(18.40,10.28) | -0.72(-0.89,-0.54) |
| Haiti | female | 1019.56(1585.61,645.14) | 1467.67(2563.51,871.13) | 0.44 | 57.30(107.59,32.48) | 42.41(78.95,24.38) | -0.89(-0.98,-0.80) |
| Honduras | female | 638.83(770.31,498.62) | 1731.31(2511.31,975.11) | 1.71 | 53.00(68.01,40.43) | 62.95(89.91,34.74) | 0.64(0.26,1.01) |
| Hungary | female | 1976.87(2665.79,1776.49) | 2692.79(3322.87,2111.41) | 0.36 | 23.68(31.51,21.27) | 21.37(26.30,16.71) | 0.05(-0.34,0.43) |
| Iceland | female | 36.23(45.73,30.73) | 57.96(69.93,42.26) | 0.60 | 20.92(26.27,17.96) | 15.93(18.98,11.55) | -1.28(-1.45,-1.11) |
| India | female | 252388.77(307456.61,193765.84) | 552052.78(689463.53,371593.40) | 1.19 | 172.54(215.26,132.99) | 113.04(141.42,75.21) | -1.69(-1.86,-1.52) |
| Indonesia | female | 27054.31(34069.12,20943.84) | 35906.99(44017.64,28045.91) | 0.33 | 63.28(83.15,47.98) | 40.53(49.74,31.65) | -1.51(-1.72,-1.30) |
| Iran (Islamic Republic of) | female | 3361.60(4272.47,2545.14) | 6763.59(7882.95,5355.93) | 1.01 | 36.15(47.85,27.19) | 22.39(26.11,17.47) | -1.73(-1.87,-1.59) |
| Iraq | female | 778.33(1077.67,546.03) | 1300.67(1805.68,985.83) | 0.67 | 19.50(28.72,13.57) | 12.63(18.60,9.70) | -1.78(-1.92,-1.64) |
| Ireland | female | 904.68(985.67,673.26) | 1201.65(1460.82,865.61) | 0.33 | 38.74(42.36,29.13) | 26.78(32.06,19.49) | -1.71(-1.96,-1.46) |
| Israel | female | 578.37(651.45,504.49) | 966.63(1364.27,780.90) | 0.67 | 23.72(26.60,20.26) | 13.19(18.61,10.73) | -2.26(-2.38,-2.15) |
| Italy | female | 7705.45(8595.57,6662.50) | 12898.19(14649.16,9412.89) | 0.67 | 14.24(15.85,12.19) | 10.89(12.34,8.40) | -0.83(-0.90,-0.76) |
| Jamaica | female | 123.78(162.34,104.21) | 170.65(270.72,123.44) | 0.38 | 11.91(15.85,9.98) | 9.55(15.59,6.89) | -1.34(-1.67,-1.01) |
| Japan | female | 10901.62(11711.95,8768.57) | 19907.50(25884.10,14425.06) | 0.83 | 11.88(12.81,9.48) | 6.40(8.38,4.90) | -2.31(-2.49,-2.13) |
| Jordan | female | 172.17(213.38,114.24) | 294.37(385.28,229.86) | 0.71 | 33.23(41.72,22.58) | 13.08(17.17,10.25) | -4.11(-4.68,-3.54) |
| Kazakhstan | female | 2893.40(3217.25,2032.83) | 3961.61(4760.35,2760.34) | 0.37 | 39.18(43.94,27.91) | 44.66(53.57,31.27) | 0.01(-0.72,0.75) |
| Kenya | female | 1625.14(2591.51,986.20) | 3434.54(5923.45,2161.03) | 1.11 | 40.81(66.45,24.79) | 33.94(57.46,21.60) | -0.28(-0.44,-0.12) |
| Kiribati | female | 25.65(34.37,19.57) | 35.31(47.19,24.73) | 0.38 | 147.32(227.59,110.54) | 122.85(165.44,82.64) | -0.53(-0.62,-0.45) |
| Kuwait | female | 33.83(39.90,26.22) | 67.83(93.17,51.66) | 1.00 | 15.76(19.04,11.96) | 8.35(11.26,6.24) | -1.85(-2.16,-1.54) |
| Kyrgyzstan | female | 1230.74(1433.10,641.07) | 660.54(1000.59,528.56) | -0.46 | 65.30(75.86,33.88) | 29.89(44.79,23.90) | -3.27(-3.93,-2.61) |
| Lao People's Democratic Republic | female | 1026.34(1352.15,714.35) | 969.90(1422.08,701.58) | -0.05 | 98.89(134.43,69.26) | 51.53(76.78,37.36) | -2.57(-2.68,-2.45) |
| Latvia | female | 277.69(312.01,236.90) | 200.40(328.87,144.27) | -0.28 | 11.76(13.19,9.88) | 6.31(10.38,4.56) | -2.02(-2.29,-1.74) |
| Lebanon | female | 301.87(396.63,221.63) | 473.76(721.50,320.94) | 0.57 | 31.61(41.93,23.07) | 16.87(25.70,11.49) | -2.41(-2.49,-2.32) |
| Lesotho | female | 456.22(812.94,262.45) | 508.54(768.15,285.45) | 0.11 | 87.31(155.78,50.57) | 79.87(119.65,45.91) | 0.54(0.17,0.90) |
| Liberia | female | 212.56(279.12,166.52) | 278.21(426.71,190.23) | 0.31 | 41.05(56.86,32.29) | 30.44(45.77,21.47) | -1.19(-1.35,-1.03) |
| Libya | female | 220.94(297.23,154.92) | 455.28(604.50,327.80) | 1.06 | 26.73(36.11,18.71) | 20.61(27.21,15.21) | -0.71(-0.97,-0.44) |
| Lithuania | female | 484.57(545.86,291.31) | 299.06(489.61,229.52) | -0.38 | 16.94(19.07,10.16) | 6.59(10.84,5.08) | -3.58(-3.88,-3.28) |
| Luxembourg | female | 62.67(80.55,54.34) | 103.81(129.36,76.52) | 0.66 | 18.63(23.53,16.26) | 15.37(19.02,11.49) | -0.73(-0.85,-0.61) |
| North Macedonia | female | 261.41(313.58,182.96) | 252.44(359.24,190.06) | -0.03 | 32.76(40.01,22.97) | 17.90(25.23,13.50) | -2.10(-2.25,-1.95) |
| Madagascar | female | 1716.64(2076.75,1428.43) | 2761.66(3813.21,2009.88) | 0.61 | 60.27(77.70,50.19) | 52.90(71.59,38.38) | -0.70(-0.89,-0.50) |
| Malawi | female | 837.09(1086.08,566.03) | 1021.57(1277.45,783.28) | 0.22 | 39.90(49.24,28.96) | 26.94(33.51,20.40) | -1.45(-1.53,-1.37) |
| Malaysia | female | 1850.15(2220.95,1341.26) | 2584.78(3952.01,1922.27) | 0.40 | 45.19(55.13,33.08) | 23.84(37.26,17.63) | -2.84(-3.17,-2.51) |
| Maldives | female | 47.95(60.11,29.63) | 60.20(73.64,47.27) | 0.26 | 157.21(191.52,104.67) | 53.74(66.27,42.02) | -4.35(-4.61,-4.09) |
| Mali | female | 1312.76(1621.73,882.16) | 1926.79(2912.87,1172.66) | 0.47 | 65.99(81.09,45.37) | 48.42(69.97,30.38) | -1.14(-1.18,-1.10) |
| Malta | female | 24.74(30.33,20.96) | 43.89(54.81,32.93) | 0.77 | 10.59(12.94,9.00) | 7.33(9.09,5.60) | -1.49(-1.61,-1.36) |
| Marshall Islands | female | 9.14(12.43,4.14) | 11.45(16.65,5.75) | 0.25 | 122.72(166.53,57.15) | 92.34(132.01,47.13) | -1.11(-1.26,-0.95) |
| Mauritania | female | 251.47(332.41,135.09) | 248.15(336.39,173.08) | -0.01 | 50.80(67.19,27.38) | 27.06(36.76,18.72) | -1.93(-2.10,-1.77) |
| Mauritius | female | 172.85(192.06,126.16) | 232.46(336.27,176.54) | 0.34 | 50.21(56.31,36.50) | 25.82(37.50,19.63) | -2.49(-2.76,-2.23) |
| Mexico | female | 7448.69(7890.19,5936.24) | 17973.26(21592.67,14328.52) | 1.41 | 42.27(45.12,32.55) | 31.80(38.15,25.16) | -1.09(-1.22,-0.96) |
| Republic of Moldova | female | 684.89(778.81,377.85) | 376.96(568.00,298.03) | -0.45 | 29.93(34.20,16.69) | 10.17(15.41,8.05) | -4.34(-4.93,-3.75) |
| Mongolia | female | 232.81(290.67,160.68) | 189.95(268.64,144.70) | -0.18 | 43.82(54.65,30.28) | 21.24(28.15,16.69) | -3.08(-3.41,-2.75) |
| Montenegro | female | 18.47(25.86,13.37) | 28.04(37.81,19.87) | 0.52 | 5.60(7.89,4.00) | 5.25(7.02,3.71) | -0.03(-0.26,0.19) |
| Morocco | female | 2227.38(3016.17,1081.82) | 3821.49(5131.42,2658.51) | 0.72 | 37.26(50.93,19.22) | 29.49(39.93,20.43) | -0.62(-0.76,-0.48) |
| Mozambique | female | 1053.24(1441.41,697.39) | 1390.70(1944.13,1020.63) | 0.32 | 32.91(42.93,23.83) | 24.74(34.37,18.33) | -1.05(-1.16,-0.95) |
| Myanmar | female | 15870.98(21075.10,8253.63) | 17733.79(21291.42,9790.89) | 0.12 | 149.26(194.68,75.65) | 80.79(96.58,44.64) | -2.39(-2.55,-2.24) |
| Namibia | female | 240.27(425.20,150.35) | 307.89(474.75,209.23) | 0.28 | 68.58(122.54,42.56) | 41.95(63.97,28.98) | -1.97(-2.20,-1.73) |
| Nepal | female | 9794.23(12538.93,6662.16) | 20897.38(25633.15,14488.65) | 1.13 | 268.57(344.80,189.31) | 224.42(274.28,154.19) | -0.58(-0.71,-0.45) |
| Netherlands | female | 2152.15(2584.25,1847.38) | 4876.22(6016.05,2861.72) | 1.27 | 16.77(20.08,14.45) | 22.54(27.50,13.10) | 0.84(0.54,1.13) |
| New Zealand | female | 613.95(723.63,548.07) | 1120.86(1307.71,811.10) | 0.83 | 27.33(31.79,24.35) | 24.09(27.88,17.54) | -0.78(-1.00,-0.56) |
| Nicaragua | female | 174.41(208.40,145.34) | 626.50(744.00,389.28) | 2.59 | 19.24(24.19,16.05) | 32.59(38.66,20.30) | 1.90(1.28,2.51) |
| Niger | female | 844.74(1101.84,593.30) | 1736.44(2514.41,1172.36) | 1.06 | 62.94(86.18,44.54) | 47.27(68.76,32.08) | -0.90(-1.02,-0.78) |
| Nigeria | female | 7779.36(10857.17,5684.21) | 10605.27(14395.10,7424.47) | 0.36 | 37.67(53.42,27.57) | 27.13(36.03,19.49) | -0.99(-1.09,-0.89) |
| Democratic People's Republic of Korea | female | 11389.01(16207.14,7206.82) | 17117.55(22389.86,13136.51) | 0.50 | 152.98(219.81,98.02) | 90.35(118.33,69.58) | -1.95(-2.14,-1.76) |
| Northern Mariana Islands | female | 3.44(4.35,2.54) | 5.86(7.86,4.69) | 0.70 | 61.41(77.49,46.02) | 33.61(44.36,26.36) | -2.10(-2.18,-2.01) |
| Norway | female | 593.10(836.75,522.75) | 1333.92(1522.65,722.84) | 1.25 | 13.56(18.28,12.13) | 21.90(24.97,11.14) | 1.76(1.46,2.06) |
| Oman | female | 74.47(109.33,48.49) | 95.37(120.08,63.27) | 0.28 | 31.26(45.85,20.24) | 20.36(26.08,12.86) | -1.08(-1.33,-0.83) |
| Pakistan | female | 16994.76(25395.61,11899.75) | 25352.20(33695.59,19771.35) | 0.49 | 78.05(118.74,53.60) | 63.35(84.29,49.62) | -0.89(-1.09,-0.69) |
| Palestine | female | 137.93(189.03,89.11) | 190.51(242.39,149.11) | 0.38 | 31.76(43.26,20.78) | 18.80(24.23,14.95) | -2.06(-2.20,-1.91) |
| Panama | female | 151.14(189.37,129.70) | 438.57(561.24,318.52) | 1.90 | 20.36(26.32,17.29) | 18.54(23.79,13.61) | -0.46(-0.69,-0.23) |
| Papua New Guinea | female | 1585.57(1976.78,1243.84) | 3467.40(4530.18,2525.26) | 1.19 | 225.49(285.40,170.48) | 206.44(265.35,149.13) | -0.22(-0.28,-0.15) |
| Paraguay | female | 188.41(237.85,159.06) | 385.35(530.64,269.76) | 1.05 | 16.19(20.96,13.49) | 13.24(18.35,9.23) | -0.67(-0.92,-0.42) |
| Peru | female | 1461.35(2015.63,1206.51) | 3232.88(4244.75,2167.96) | 1.21 | 23.05(34.49,18.79) | 18.50(24.29,12.33) | -0.36(-0.61,-0.10) |
| Philippines | female | 7752.81(8985.86,6261.85) | 11129.39(16232.05,8578.96) | 0.44 | 62.89(75.21,51.97) | 31.73(48.86,24.38) | -2.41(-2.58,-2.24) |
| Poland | female | 4158.68(4472.99,3445.32) | 4286.56(6171.92,3315.38) | 0.03 | 16.32(17.58,13.34) | 9.22(13.00,7.14) | -1.87(-2.06,-1.67) |
| Portugal | female | 1753.74(2066.92,1522.72) | 3159.51(3853.32,2269.73) | 0.80 | 23.04(26.60,19.84) | 16.42(19.96,12.64) | -1.30(-1.48,-1.12) |
| Puerto Rico | female | 437.75(501.79,347.49) | 930.64(1201.85,609.69) | 1.13 | 23.29(26.75,18.35) | 18.67(23.87,12.95) | -1.59(-1.94,-1.23) |
| Qatar | female | 8.83(12.41,6.49) | 19.50(30.90,13.99) | 1.21 | 31.33(46.09,22.53) | 27.89(40.51,21.13) | -0.11(-0.30,0.07) |
| Romania | female | 5279.27(5965.98,2565.04) | 2912.87(4390.30,2209.99) | -0.45 | 39.83(45.29,18.89) | 11.89(17.99,9.09) | -4.30(-4.61,-3.99) |
| Russian Federation | female | 22780.47(25383.53,12508.23) | 14637.54(20747.41,11956.02) | -0.36 | 20.01(22.34,10.94) | 9.14(13.06,7.45) | -3.34(-3.62,-3.05) |
| Rwanda | female | 1232.70(1649.01,729.58) | 1204.82(2018.38,835.32) | -0.02 | 74.82(98.06,46.76) | 39.40(64.30,28.13) | -2.70(-2.92,-2.48) |
| Saint Lucia | female | 9.90(11.75,8.53) | 17.25(21.54,13.08) | 0.74 | 20.63(24.51,17.73) | 15.26(19.09,11.55) | -1.63(-1.94,-1.31) |
| Saint Vincent and the Grenadines | female | 3.92(4.93,3.22) | 5.42(6.64,4.18) | 0.38 | 9.73(12.20,7.97) | 8.64(10.58,6.64) | -0.36(-0.68,-0.04) |
| Samoa | female | 43.76(67.49,30.69) | 50.15(67.31,35.95) | 0.15 | 107.53(165.07,75.88) | 73.83(98.70,53.59) | -1.40(-1.53,-1.26) |
| Sao Tome and Principe | female | 26.93(31.98,20.94) | 33.59(46.15,19.00) | 0.25 | 85.12(100.22,66.37) | 71.44(97.16,39.91) | -0.72(-0.86,-0.58) |
| Saudi Arabia | female | 1191.24(1821.30,802.28) | 1522.76(2022.01,1147.83) | 0.28 | 55.94(84.99,36.93) | 31.13(40.89,22.75) | -2.08(-2.17,-1.99) |
| Senegal | female | 741.39(936.92,540.61) | 1180.80(1530.70,902.88) | 0.59 | 49.11(61.73,36.43) | 33.81(43.53,26.13) | -0.94(-1.20,-0.67) |
| Serbia | female | 1227.38(1603.43,1005.24) | 1483.13(1891.87,1118.60) | 0.21 | 22.97(30.27,18.78) | 17.34(22.37,13.15) | -1.15(-1.34,-0.96) |
| Seychelles | female | 9.76(11.47,7.90) | 12.04(14.53,9.06) | 0.23 | 28.27(33.04,22.90) | 22.29(26.96,16.85) | -0.84(-0.96,-0.72) |
| Sierra Leone | female | 448.56(590.86,323.78) | 673.68(920.51,491.16) | 0.50 | 49.95(65.85,36.26) | 39.48(53.77,29.13) | -0.42(-0.59,-0.25) |
| Singapore | female | 278.21(316.30,161.98) | 278.53(484.21,209.58) | 0.00 | 27.29(31.09,15.97) | 6.87(11.97,5.20) | -4.93(-5.31,-4.54) |
| Slovakia | female | 357.53(590.33,293.59) | 426.59(601.36,324.22) | 0.19 | 10.52(17.12,8.66) | 7.61(10.50,5.79) | -0.75(-0.89,-0.61) |
| Slovenia | female | 197.61(252.87,135.44) | 242.48(316.47,168.91) | 0.23 | 13.16(16.88,9.01) | 7.44(9.68,5.29) | -2.66(-2.98,-2.34) |
| Solomon Islands | female | 63.36(88.83,44.69) | 131.02(163.87,98.51) | 1.07 | 124.79(182.51,86.15) | 110.46(134.98,80.18) | -0.36(-0.41,-0.31) |
| Somalia | female | 904.36(1644.69,498.34) | 1898.70(3834.94,1071.42) | 1.10 | 65.29(123.67,37.56) | 53.49(105.77,30.31) | -0.51(-0.61,-0.41) |
| South Africa | female | 5590.43(7194.65,4329.19) | 7899.25(9604.39,6972.98) | 0.41 | 48.05(63.29,36.89) | 34.01(41.03,29.75) | -1.38(-1.89,-0.88) |
| Republic of Korea | female | 5661.78(6488.05,2987.29) | 6496.10(7850.04,4799.43) | 0.15 | 49.44(57.69,24.66) | 12.54(15.18,9.25) | -5.65(-6.07,-5.23) |
| South Sudan | female | 463.59(652.59,243.98) | 431.32(679.42,262.25) | -0.07 | 37.73(52.35,21.53) | 25.27(39.50,15.87) | -1.39(-1.54,-1.24) |
| Spain | female | 7555.11(8500.00,5813.48) | 14735.77(18872.11,9076.04) | 0.95 | 23.11(26.09,17.65) | 17.74(22.12,11.90) | -1.07(-1.19,-0.96) |
| Sri Lanka | female | 3800.35(4371.38,2949.61) | 6230.90(8374.17,3881.67) | 0.64 | 93.22(107.04,71.59) | 51.97(69.79,31.93) | -1.41(-1.64,-1.18) |
| Sudan | female | 2405.61(3680.43,1431.52) | 2524.09(3862.03,1654.03) | 0.05 | 56.66(95.49,31.18) | 33.68(53.34,21.66) | -1.87(-1.96,-1.78) |
| Suriname | female | 27.85(35.63,23.84) | 49.08(60.46,37.60) | 0.76 | 20.26(25.67,17.44) | 15.70(19.35,11.98) | -1.00(-1.20,-0.79) |
| Eswatini | female | 99.08(171.45,60.44) | 120.03(179.26,73.78) | 0.21 | 65.39(113.92,39.68) | 41.72(61.10,26.00) | -1.30(-1.65,-0.94) |
| Sweden | female | 1017.11(1453.19,880.63) | 2318.19(2788.53,1332.80) | 1.28 | 10.64(14.50,9.38) | 16.36(19.44,9.17) | 1.61(1.41,1.82) |
| Switzerland | female | 843.79(988.19,699.46) | 1347.53(1634.96,977.47) | 0.60 | 12.11(14.03,10.18) | 11.08(13.22,7.92) | -0.30(-0.39,-0.21) |
| Syrian Arab Republic | female | 966.14(1317.86,691.90) | 1340.24(1904.36,997.91) | 0.39 | 42.20(60.74,29.69) | 33.54(47.74,25.66) | -1.10(-1.28,-0.92) |
| Taiwan (Province of China) | female | 1782.08(2009.72,1293.36) | 3178.48(4872.50,2262.71) | 0.78 | 32.27(36.83,23.87) | 13.95(21.46,9.94) | -3.45(-3.72,-3.18) |
| Tajikistan | female | 820.60(946.11,618.06) | 749.94(1196.22,558.10) | -0.09 | 51.41(59.09,38.42) | 40.98(68.14,29.59) | -0.95(-1.18,-0.71) |
| United Republic of Tanzania | female | 1794.19(2208.78,1387.52) | 3155.70(4154.04,2383.95) | 0.76 | 32.19(39.06,24.69) | 25.14(32.24,19.37) | -0.68(-0.77,-0.59) |
| Thailand | female | 8098.70(10295.95,4798.62) | 8036.76(11416.00,5686.73) | -0.01 | 50.96(64.90,30.08) | 14.59(20.72,10.35) | -5.02(-5.29,-4.76) |
| Bahamas | female | 9.19(11.02,7.54) | 19.25(24.74,14.73) | 1.10 | 10.77(13.06,8.73) | 9.65(12.40,7.33) | -0.41(-0.52,-0.31) |
| Gambia | female | 75.74(107.90,51.25) | 184.91(282.91,130.12) | 1.44 | 48.46(67.35,33.64) | 40.02(60.44,28.70) | -0.57(-0.79,-0.35) |
| Timor-Leste | female | 117.81(156.73,78.13) | 188.38(284.81,129.20) | 0.60 | 87.65(127.93,59.54) | 55.41(86.16,39.37) | -1.95(-2.17,-1.73) |
| Togo | female | 329.84(424.73,245.87) | 609.46(888.80,439.63) | 0.85 | 54.15(70.11,40.12) | 34.24(49.20,25.08) | -1.32(-1.55,-1.08) |
| Tonga | female | 10.91(14.04,8.53) | 13.66(18.40,8.86) | 0.25 | 43.72(56.60,34.23) | 30.52(41.38,19.75) | -1.19(-1.33,-1.06) |
| Trinidad and Tobago | female | 79.07(91.74,69.23) | 96.47(152.98,68.26) | 0.22 | 18.47(21.62,16.23) | 10.24(15.99,7.26) | -2.18(-2.45,-1.91) |
| Tunisia | female | 537.88(787.72,386.02) | 1030.72(1507.98,724.68) | 0.92 | 27.06(39.92,19.21) | 17.56(25.68,12.30) | -1.48(-1.52,-1.45) |
| Turkey | female | 7221.52(8718.09,5341.45) | 12593.54(16107.79,7397.84) | 0.74 | 43.01(52.49,31.11) | 28.15(35.93,16.53) | -1.02(-1.48,-0.54) |
| Turkmenistan | female | 411.82(464.98,236.16) | 202.41(531.33,128.68) | -0.51 | 40.55(45.74,23.50) | 10.41(28.04,6.56) | -5.54(-6.10,-4.97) |
| Uganda | female | 1548.12(2419.57,900.21) | 2223.39(3392.60,1486.49) | 0.44 | 46.19(70.15,26.65) | 30.20(45.83,20.32) | -1.72(-1.84,-1.60) |
| Ukraine | female | 13518.15(15617.67,6683.11) | 4392.67(10285.17,3004.82) | -0.68 | 29.83(34.59,14.82) | 8.55(20.01,5.84) | -5.64(-6.14,-5.15) |
| United Arab Emirates | female | 65.62(96.35,42.98) | 207.27(285.38,145.16) | 2.16 | 66.31(102.86,43.81) | 31.73(43.51,23.35) | -2.29(-3.30,-1.28) |
| United Kingdom | female | 14900.91(20137.50,13458.03) | 25011.88(27541.99,17192.27) | 0.68 | 25.89(33.94,23.53) | 30.77(33.58,20.98) | 0.55(0.41,0.69) |
| United States of America | female | 46706.81(62786.90,41675.89) | 112466.68(124675.62,71152.95) | 1.41 | 23.44(30.91,21.18) | 33.04(36.20,21.49) | 1.07(0.78,1.36) |
| Uruguay | female | 401.53(621.63,337.70) | 879.16(1046.22,697.77) | 1.19 | 17.94(27.42,15.23) | 23.01(26.93,18.02) | 0.92(0.77,1.08) |
| Uzbekistan | female | 2332.69(2690.48,1332.14) | 1783.25(3392.61,1329.29) | -0.24 | 36.95(42.83,21.16) | 24.91(53.59,18.28) | -2.25(-2.77,-1.72) |
| Vanuatu | female | 30.65(51.64,18.66) | 63.98(97.24,42.82) | 1.09 | 126.93(218.90,77.14) | 94.84(145.10,64.17) | -1.30(-1.44,-1.15) |
| Venezuela (Bolivarian Republic of) | female | 969.64(1203.72,839.95) | 3306.06(4344.13,2402.85) | 2.41 | 20.35(26.31,17.24) | 21.60(28.49,15.69) | 0.01(-0.17,0.19) |
| Viet nam | female | 11199.99(16289.32,4115.78) | 13795.17(19121.64,4205.83) | 0.23 | 51.30(74.96,18.58) | 29.18(40.43,8.88) | -1.98(-2.02,-1.94) |
| Virginia | female | 1064.08(1383.70,942.59) | 2661.54(3389.64,1678.91) | 1.50 | 24.91(31.99,22.16) | 31.67(40.33,20.24) | 0.56(0.22,0.90) |
| Yemen | female | 1557.44(2576.26,931.82) | 2521.30(3354.11,1811.87) | 0.62 | 67.34(118.02,38.77) | 44.24(59.39,31.82) | -1.67(-1.81,-1.54) |
| Zambia | female | 672.44(874.33,484.19) | 967.38(1234.49,731.33) | 0.44 | 45.09(56.12,34.58) | 29.90(37.88,22.25) | -1.57(-1.66,-1.48) |
| Zimbabwe | female | 749.84(891.42,545.90) | 1343.49(1991.85,694.67) | 0.79 | 40.94(48.48,30.09) | 39.24(57.35,20.97) | 0.98(0.45,1.51) |
| Monaco | female | 5.89(8.85,4.24) | 8.73(10.91,6.36) | 0.48 | 11.54(17.46,8.36) | 12.85(16.28,9.42) | 0.77(0.45,1.09) |
| San Marino | female | 2.38(3.12,1.87) | 5.35(7.55,3.50) | 1.25 | 12.46(16.13,9.85) | 11.45(16.56,7.49) | 0.24(-0.01,0.49) |
| Saint Kitts and Nevis | female | 4.31(5.20,3.58) | 4.49(5.67,3.40) | 0.04 | 20.89(25.08,17.37) | 15.15(19.13,11.51) | -0.57(-0.79,-0.35) |
| Cook Islands | female | 3.63(4.45,2.77) | 3.80(4.83,2.93) | 0.05 | 68.47(83.64,51.91) | 31.43(40.17,24.03) | -2.72(-2.89,-2.56) |
| Nauru | female | 1.65(2.17,1.15) | 1.22(1.75,0.62) | -0.26 | 112.88(141.68,82.04) | 77.44(105.12,42.42) | -1.26(-1.59,-0.93) |
| Niue | female | 1.15(1.52,0.72) | 0.55(0.77,0.30) | -0.52 | 76.65(100.28,48.59) | 45.40(64.15,24.77) | -2.03(-2.11,-1.94) |
| Palau | female | 4.84(6.89,3.46) | 6.27(7.93,4.68) | 0.30 | 119.16(171.31,85.27) | 82.37(102.33,62.32) | -1.18(-1.29,-1.07) |
| Tokelau | female | 0.87(1.13,0.66) | 0.41(0.54,0.28) | -0.53 | 123.84(161.05,93.22) | 66.71(87.73,46.12) | -2.25(-2.32,-2.18) |
| Tuvalu | female | 4.77(6.54,3.49) | 3.59(4.90,2.22) | -0.25 | 143.67(200.97,103.75) | 78.70(105.71,49.29) | -2.15(-2.25,-2.06) |

**Supplementary Table 5. The DALYs and age-standardized DALY rate of chronic obstructive pulmonary disease in 1990 and 2019, and its temporal trends from 1990 to 2019.**

| **Nation** | **Sex** | **DALY Cases No. (95% UI)** | | **Change in absolute number (%)** | **Age-standardized DALY rate per 100,000 No.(95% UI)** | | **1990-2019 EAPC No. (95%CI)** |
| --- | --- | --- | --- | --- | --- | --- | --- |
|  |  | **1990** | **2019** |  | **1990** | **2019** |  |
| Afghanistan | both | 191922.97(226504.12,160093.31) | 306360.72(369966.64,250838.08) | 0.60 | 2417.04(2856.97,2022.96) | 1754.03(2077.07,1422.52) | -1.28(-1.52,-1.04) |
| Albania | both | 24581.98(27109.14,19698.96) | 19951.39(24255.18,16192.67) | -0.19 | 1179.92(1288.31,906.55) | 527.32(642.15,430.71) | -3.03(-3.41,-2.64) |
| Algeria | both | 165365.27(199552.90,135559.56) | 267334.90(317521.99,226065.12) | 0.62 | 1175.93(1417.90,960.21) | 788.59(929.80,668.69) | -1.43(-1.51,-1.35) |
| American Samoa | both | 556.18(628.26,490.76) | 646.36(770.98,551.75) | 0.16 | 2119.32(2360.45,1866.76) | 1370.04(1643.94,1177.28) | -1.65(-1.73,-1.57) |
| Andorra | both | 545.69(669.26,443.92) | 1034.13(1217.88,860.58) | 0.90 | 1031.39(1251.41,851.66) | 830.99(993.13,679.80) | -0.83(-0.92,-0.75) |
| Angola | both | 128386.45(166379.32,97462.15) | 189114.06(226499.08,153309.08) | 0.47 | 2194.06(2779.55,1705.30) | 1248.50(1527.07,1016.23) | -2.17(-2.32,-2.03) |
| Antigua and Barbuda | both | 282.36(356.96,227.34) | 435.27(534.12,353.75) | 0.54 | 477.73(595.16,391.06) | 501.45(628.48,398.66) | 0.11(0.04,0.19) |
| Argentina | both | 341073.51(392041.69,298946.06) | 529348.06(602799.51,461244.15) | 0.55 | 1057.00(1211.30,927.60) | 1031.28(1194.09,893.04) | -0.27(-0.40,-0.14) |
| Armenia | both | 28690.74(31103.41,25356.68) | 28248.44(32467.18,24105.96) | -0.02 | 1089.63(1174.05,949.69) | 727.73(836.92,619.41) | -1.67(-1.83,-1.51) |
| Australia | both | 248170.52(283528.35,215374.96) | 328077.11(374682.79,283932.78) | 0.32 | 1345.83(1561.33,1152.48) | 945.42(1138.96,786.39) | -1.56(-1.70,-1.41) |
| Austria | both | 80705.55(93342.12,69820.23) | 96018.67(109715.06,82911.93) | 0.19 | 763.56(909.30,646.99) | 651.41(777.17,543.85) | -0.51(-0.56,-0.47) |
| Azerbaijan | both | 58854.71(64424.58,53533.41) | 65300.34(85406.89,54934.95) | 0.11 | 1092.61(1190.98,996.32) | 758.76(1026.08,636.60) | -1.89(-2.27,-1.50) |
| Bahrain | both | 3496.35(4029.29,3047.95) | 7571.41(9052.07,6382.09) | 1.17 | 1719.55(1932.05,1532.53) | 875.79(1030.56,751.56) | -2.65(-3.04,-2.27) |
| Bangladesh | both | 1730648.37(2053532.88,1505307.22) | 1942679.02(2790908.01,1593316.13) | 0.12 | 3434.42(4181.58,2989.00) | 1499.65(2192.86,1228.50) | -3.01(-3.20,-2.82) |
| Barbados | both | 1536.37(1850.01,1261.61) | 2058.77(2481.79,1703.17) | 0.34 | 570.34(699.11,461.06) | 576.69(731.58,457.07) | -0.09(-0.18,-0.01) |
| Belarus | both | 166779.89(184151.46,137666.10) | 70913.89(94487.15,57600.21) | -0.57 | 1336.23(1481.99,1099.21) | 517.73(669.25,413.95) | -4.17(-4.53,-3.81) |
| Belgium | both | 172780.66(191834.09,156122.44) | 178071.33(198308.87,157983.61) | 0.03 | 1208.83(1371.05,1073.49) | 878.54(1011.86,763.04) | -1.30(-1.39,-1.22) |
| Belize | both | 1246.95(1529.77,1021.91) | 3128.31(3732.62,2620.13) | 1.51 | 804.34(931.21,689.78) | 935.94(1092.21,793.63) | 0.25(-0.05,0.56) |
| Benin | both | 49496.96(57766.06,41197.58) | 86780.31(107611.53,70382.68) | 0.75 | 1841.12(2124.84,1542.65) | 1244.48(1517.17,1030.23) | -1.22(-1.34,-1.09) |
| Bermuda | both | 380.69(445.47,326.26) | 472.98(560.78,399.02) | 0.24 | 650.54(772.73,550.43) | 544.71(681.55,435.41) | -0.67(-0.73,-0.61) |
| Bhutan | both | 8995.84(11532.80,6734.74) | 13059.13(17103.87,10397.47) | 0.45 | 3547.71(4520.09,2710.15) | 2436.44(3190.00,1953.52) | -1.34(-1.37,-1.31) |
| Bolivia (Plurinational State of) | both | 86366.34(108920.47,63561.56) | 98267.21(118545.88,79670.55) | 0.14 | 1619.83(1905.41,1317.49) | 1063.53(1274.71,870.16) | -1.48(-1.59,-1.36) |
| Bosnia and Herzegovina | both | 37307.18(41834.30,32453.68) | 33918.74(40118.13,28376.43) | -0.09 | 956.12(1062.25,834.57) | 662.32(786.31,551.63) | -1.48(-1.59,-1.37) |
| Botswana | both | 15417.06(20251.95,11745.57) | 24044.20(29776.76,19002.18) | 0.56 | 2340.52(3114.17,1789.23) | 1589.19(1956.25,1266.81) | -1.75(-2.07,-1.43) |
| Brazil | both | 1436484.75(1671650.61,1254143.23) | 2042183.89(2300990.12,1835929.41) | 0.42 | 1387.29(1538.71,1257.26) | 915.42(1043.39,813.67) | -1.85(-2.01,-1.69) |
| Brunei Darussalam | both | 2279.72(2587.00,1886.56) | 3170.70(3657.05,2760.50) | 0.39 | 2401.96(2691.40,1859.45) | 1292.76(1451.17,1146.28) | -2.29(-2.40,-2.17) |
| Bulgaria | both | 111059.01(121998.04,100060.81) | 83661.44(99652.73,70106.61) | -0.25 | 975.71(1082.37,876.51) | 692.69(818.51,575.69) | -1.48(-1.63,-1.33) |
| Burkina Faso | both | 72471.74(86608.60,59100.68) | 139722.30(168459.88,114072.69) | 0.93 | 1183.41(1370.14,997.84) | 992.47(1155.76,844.37) | -0.67(-0.80,-0.53) |
| Burundi | both | 96798.34(120785.35,71432.94) | 109117.02(144243.72,85848.24) | 0.13 | 2696.00(3261.29,2050.63) | 1675.53(2118.80,1324.13) | -1.85(-1.94,-1.76) |
| Cambodia | both | 96700.36(111358.36,81426.88) | 150282.58(172225.14,125987.44) | 0.55 | 1732.93(1972.20,1475.95) | 1264.58(1439.33,1062.21) | -1.16(-1.22,-1.11) |
| Cameroon | both | 100022.06(116073.53,85164.83) | 195153.97(240339.53,155381.28) | 0.95 | 1804.48(2091.54,1519.81) | 1208.68(1491.33,975.49) | -1.28(-1.34,-1.22) |
| Canada | both | 269100.30(302712.27,239674.34) | 446332.32(499639.89,388800.82) | 0.66 | 867.97(993.29,763.01) | 744.36(860.93,640.90) | -0.65(-0.71,-0.59) |
| Cabo Verde | both | 3923.53(4394.84,2849.71) | 3658.90(4414.00,3161.54) | -0.07 | 1552.88(1724.71,1102.67) | 779.55(939.70,675.18) | -2.45(-2.97,-1.93) |
| Central African Republic | both | 42124.62(52084.72,32681.79) | 63886.57(84358.01,47596.30) | 0.52 | 2805.54(3690.39,2101.05) | 2323.42(3319.25,1683.25) | -0.65(-0.70,-0.59) |
| Chad | both | 64860.07(80819.09,52433.82) | 118887.17(144239.34,97016.49) | 0.83 | 1813.07(2315.09,1456.23) | 1433.32(1745.24,1165.05) | -0.67(-0.73,-0.62) |
| Chile | both | 90726.24(104593.56,78355.31) | 160293.00(184273.86,136185.56) | 0.77 | 867.05(982.71,756.80) | 721.04(845.92,609.75) | -0.51(-0.60,-0.42) |
| China | both | 28992086.01(32387822.62,22016544.87) | 22520549.05(26416880.47,19719778.94) | -0.22 | 3910.13(4343.33,2991.37) | 1269.95(1473.89,1114.00) | -4.22(-4.39,-4.06) |
| Colombia | both | 221546.31(259032.49,192564.08) | 388120.21(468216.50,318755.76) | 0.75 | 1051.50(1167.54,935.26) | 755.57(916.63,620.82) | -1.46(-1.59,-1.33) |
| Comoros | both | 5011.04(6448.62,3118.77) | 6125.42(7319.66,5017.58) | 0.22 | 1687.06(2125.52,1108.38) | 1117.08(1338.07,929.59) | -1.61(-1.77,-1.44) |
| Congo | both | 30064.67(37233.95,23633.01) | 41549.57(50462.91,32629.39) | 0.38 | 2296.31(2988.05,1751.80) | 1366.85(1657.67,1078.72) | -1.96(-2.06,-1.85) |
| Costa Rica | both | 19792.77(24528.24,16009.83) | 35890.26(43173.49,29337.71) | 0.81 | 868.95(1017.37,742.05) | 736.98(891.75,601.24) | -1.05(-1.39,-0.71) |
| Côte d'Ivoire | both | 103761.92(122857.21,84357.23) | 176610.72(213455.66,141402.63) | 0.70 | 1782.18(2095.61,1463.58) | 1180.16(1416.73,969.00) | -1.34(-1.41,-1.27) |
| Croatia | both | 43882.19(50018.13,38231.63) | 48168.27(56092.98,40609.19) | 0.10 | 739.08(849.86,635.60) | 632.04(747.77,523.86) | -0.52(-0.56,-0.48) |
| Cuba | both | 81811.67(98774.07,67823.11) | 130376.81(154175.23,105524.37) | 0.59 | 777.63(940.95,645.23) | 852.63(1038.33,683.81) | 0.21(0.12,0.31) |
| Cyprus | both | 8999.65(10499.00,7591.71) | 14573.70(17063.72,12168.73) | 0.62 | 1235.10(1425.41,1042.66) | 864.13(1027.72,706.84) | -1.47(-1.61,-1.32) |
| Czechia | both | 93750.49(104445.78,85082.22) | 113490.28(130423.65,96193.74) | 0.21 | 731.97(820.93,658.15) | 628.40(728.31,528.72) | 0.03(-0.28,0.34) |
| Democratic Republic of the Congo | both | 443579.89(558208.74,331043.05) | 750175.01(1015026.03,563758.11) | 0.69 | 2102.43(2817.96,1518.79) | 1741.07(2575.29,1252.30) | -0.62(-0.75,-0.48) |
| Denmark | both | 91405.61(100479.74,81770.96) | 107983.68(120280.27,91084.04) | 0.18 | 1223.06(1369.10,1076.41) | 1029.97(1167.13,867.05) | -0.89(-1.09,-0.70) |
| Djibouti | both | 3757.33(5001.76,2856.07) | 7748.08(9966.04,6028.47) | 1.06 | 1423.01(1817.38,1057.09) | 991.33(1272.10,769.16) | -1.43(-1.49,-1.36) |
| Dominica | both | 588.69(707.84,496.09) | 645.55(768.75,524.15) | 0.10 | 797.06(948.72,673.31) | 831.40(1007.70,674.39) | 0.14(0.05,0.23) |
| Dominican Republic | both | 49586.58(58988.78,40659.79) | 67305.61(83321.72,52758.94) | 0.36 | 796.05(912.39,681.21) | 679.24(834.95,532.55) | -0.10(-0.35,0.16) |
| Ecuador | both | 81139.86(98798.43,63435.84) | 97494.01(118125.00,80360.51) | 0.20 | 943.15(1082.32,794.22) | 644.03(774.65,535.17) | -0.90(-1.33,-0.48) |
| Egypt | both | 594519.72(666379.35,524693.15) | 884313.49(1070558.80,708469.16) | 0.49 | 1589.94(1751.93,1440.74) | 1270.71(1541.90,1014.44) | -0.62(-0.70,-0.54) |
| El Salvador | both | 53701.88(65597.91,42946.71) | 41373.16(50576.33,33438.18) | -0.23 | 1157.09(1346.20,980.65) | 668.58(817.76,539.10) | -1.98(-2.27,-1.68) |
| Equatorial Guinea | both | 5957.92(7632.57,4535.66) | 7194.83(9638.72,5420.14) | 0.21 | 2381.51(3250.74,1736.71) | 1103.59(1607.34,826.80) | -3.01(-3.29,-2.73) |
| Eritrea | both | 37984.72(47686.76,26949.76) | 56263.75(69334.02,44944.13) | 0.48 | 2190.23(2720.81,1610.23) | 1486.04(1810.97,1145.96) | -1.39(-1.43,-1.35) |
| Estonia | both | 10554.93(11569.81,9504.66) | 7682.99(9161.84,6425.48) | -0.27 | 551.06(609.58,491.78) | 354.15(425.71,293.97) | -1.77(-1.94,-1.61) |
| Ethiopia | both | 594894.50(692924.04,476396.55) | 583464.51(687520.36,497646.18) | -0.02 | 2022.89(2298.69,1689.55) | 1004.10(1141.00,879.60) | -2.68(-2.78,-2.58) |
| Micronesia (Federated States of) | both | 2270.64(2731.72,1840.28) | 1827.45(2315.73,1341.69) | -0.20 | 4041.83(4939.22,3233.56) | 2495.98(3120.58,1902.41) | -1.78(-1.92,-1.64) |
| Fiji | both | 12980.59(15857.02,10558.22) | 12115.12(15187.23,9744.68) | -0.07 | 3107.18(3785.36,2537.09) | 1638.61(2043.92,1326.91) | -2.68(-2.92,-2.44) |
| Finland | both | 45526.44(52946.74,39129.03) | 59869.34(69114.65,51556.34) | 0.32 | 711.85(851.53,602.61) | 618.38(755.88,511.65) | -0.43(-0.47,-0.40) |
| France | both | 644104.92(765278.55,544033.49) | 580693.01(684073.75,490980.64) | -0.10 | 865.31(1057.94,717.70) | 554.08(691.92,444.88) | -1.75(-1.91,-1.59) |
| Gabon | both | 10197.70(12229.93,8018.20) | 11585.59(14008.49,9247.07) | 0.14 | 1611.35(1942.56,1248.04) | 996.96(1208.80,794.31) | -1.72(-1.77,-1.68) |
| Georgia | both | 36240.84(42939.37,32217.54) | 32826.92(39908.50,27856.33) | -0.09 | 621.73(738.61,553.53) | 623.69(747.17,529.29) | 0.81(0.43,1.19) |
| Germany | both | 1181831.93(1327524.30,1057003.60) | 1217222.26(1348838.68,1083445.14) | 0.03 | 1023.23(1168.36,902.10) | 749.37(856.59,650.76) | -0.85(-1.09,-0.61) |
| Ghana | both | 121680.53(146473.09,82827.39) | 239465.16(288740.93,170900.90) | 0.97 | 1455.82(1750.96,999.14) | 1192.88(1431.63,834.42) | -0.36(-0.49,-0.24) |
| Greece | both | 94187.87(109089.35,80621.05) | 151294.28(169122.99,130379.33) | 0.61 | 692.36(824.23,582.91) | 723.94(843.16,610.80) | 0.70(0.47,0.93) |
| Greenland | both | 833.38(949.03,712.27) | 954.86(1101.87,794.16) | 0.15 | 2405.96(2700.57,2043.29) | 1517.20(1749.89,1259.38) | -2.06(-2.25,-1.87) |
| Grenada | both | 670.75(810.39,554.94) | 782.20(941.01,653.70) | 0.17 | 824.18(972.63,693.24) | 754.76(917.92,622.74) | -0.30(-0.40,-0.19) |
| Guam | both | 1104.19(1256.41,965.82) | 1509.79(1770.32,1288.16) | 0.37 | 1337.99(1495.38,1184.06) | 837.35(979.48,712.17) | -1.82(-1.98,-1.66) |
| Guatemala | both | 91696.83(106949.06,72527.26) | 82743.90(100856.61,67098.19) | -0.10 | 1323.22(1492.29,1142.04) | 622.87(759.79,507.02) | -2.89(-3.36,-2.41) |
| Guinea | both | 80069.45(94695.76,67037.66) | 115230.55(139979.01,93275.77) | 0.44 | 1921.76(2298.83,1600.86) | 1540.77(1839.41,1264.49) | -0.47(-0.63,-0.31) |
| Guinea-Bissau | both | 14436.71(17365.08,11740.76) | 17097.06(20746.77,13894.58) | 0.18 | 2656.75(3195.94,2176.25) | 1701.01(2046.35,1381.12) | -1.29(-1.48,-1.10) |
| Guyana | both | 5008.39(6143.75,4090.15) | 5538.41(6819.63,4391.11) | 0.11 | 872.81(1023.33,739.91) | 795.91(974.81,635.68) | -0.39(-0.46,-0.31) |
| Haiti | both | 126401.31(162758.53,86503.16) | 156415.09(200311.73,113031.82) | 0.24 | 2304.34(2922.25,1696.67) | 1633.86(2135.55,1183.35) | -1.01(-1.11,-0.91) |
| Honduras | both | 63037.09(74940.35,49732.38) | 99588.33(126628.99,74639.38) | 0.58 | 1659.67(1883.01,1403.08) | 1498.78(1919.43,1071.53) | -0.27(-0.39,-0.15) |
| Hungary | both | 148034.86(164796.58,136758.68) | 159071.02(182155.42,137391.09) | 0.07 | 1092.48(1213.87,999.47) | 947.13(1099.23,815.59) | -0.31(-0.57,-0.06) |
| Iceland | both | 2686.37(3183.23,2267.89) | 3672.25(4295.97,3122.10) | 0.37 | 977.39(1170.11,820.23) | 767.73(936.22,634.88) | -0.95(-1.08,-0.82) |
| India | both | 17098713.25(18978574.09,14491558.82) | 29220636.47(33209302.98,24324295.41) | 0.71 | 4044.09(4546.31,3399.27) | 2678.84(3047.93,2208.00) | -1.50(-1.59,-1.41) |
| Indonesia | both | 2335847.94(2573682.87,2062937.60) | 3181366.02(3576092.01,2768328.03) | 0.36 | 2058.88(2280.67,1811.97) | 1512.61(1687.81,1319.37) | -0.96(-1.05,-0.86) |
| Iran (Islamic Republic of) | both | 365428.54(425585.21,315782.94) | 587911.35(661391.85,521418.31) | 0.61 | 1113.20(1275.37,983.72) | 794.09(886.50,705.19) | -1.20(-1.24,-1.17) |
| Iraq | both | 106189.57(126870.93,87979.63) | 176571.95(214791.39,142692.59) | 0.66 | 946.97(1126.30,804.13) | 585.93(699.67,485.28) | -2.03(-2.18,-1.88) |
| Ireland | both | 64325.01(72333.71,56141.57) | 65744.94(74881.32,57264.70) | 0.02 | 1601.67(1819.78,1391.26) | 969.44(1127.54,829.83) | -2.03(-2.18,-1.89) |
| Israel | both | 43068.15(49997.49,37291.47) | 64746.76(76230.66,55570.30) | 0.50 | 895.85(1037.58,777.46) | 601.45(717.34,510.63) | -1.53(-1.65,-1.41) |
| Italy | both | 676663.82(761842.81,602867.95) | 668768.38(744429.51,592133.06) | -0.01 | 818.90(936.68,717.73) | 538.61(635.23,454.14) | -1.68(-1.80,-1.57) |
| Jamaica | both | 16691.12(20334.22,13734.07) | 21039.02(25548.66,17077.79) | 0.26 | 760.43(901.62,640.49) | 741.27(906.47,597.89) | -0.40(-0.70,-0.09) |
| Japan | both | 1203935.13(1429728.91,1018865.99) | 1291053.01(1448930.85,1128961.06) | 0.07 | 802.54(967.96,666.83) | 453.68(548.18,377.58) | -2.37(-2.56,-2.17) |
| Jordan | both | 19489.80(23472.24,16198.38) | 51394.41(62913.26,41508.28) | 1.64 | 1051.06(1214.80,893.42) | 632.36(747.82,530.80) | -2.19(-2.38,-2.00) |
| Kazakhstan | both | 190127.49(204355.41,170415.46) | 242742.85(281892.60,209317.84) | 0.28 | 1473.59(1583.94,1317.08) | 1468.09(1693.92,1261.48) | -0.58(-1.07,-0.08) |
| Kenya | both | 153497.17(197372.01,123473.85) | 330427.06(416175.10,269943.23) | 1.15 | 1295.37(1794.06,1017.78) | 1176.36(1487.63,957.51) | -0.11(-0.28,0.05) |
| Kiribati | both | 2029.61(2388.40,1700.68) | 2428.69(2979.02,1952.27) | 0.20 | 4520.24(5400.78,3759.20) | 3237.78(3956.69,2610.18) | -1.16(-1.22,-1.10) |
| Kuwait | both | 6473.44(8223.52,5195.75) | 15859.54(19931.45,12442.92) | 1.45 | 595.51(697.73,501.65) | 470.22(575.58,383.06) | -0.75(-0.87,-0.63) |
| Kyrgyzstan | both | 68012.64(74355.92,56837.88) | 44911.31(53271.30,38755.75) | -0.34 | 2164.99(2357.10,1791.48) | 970.61(1152.83,847.42) | -3.56(-4.10,-3.02) |
| Lao People's Democratic Republic | both | 91297.53(112879.12,71047.35) | 84578.74(102747.04,69439.48) | -0.07 | 3398.25(4076.63,2733.25) | 1830.87(2206.29,1507.08) | -2.36(-2.44,-2.28) |
| Latvia | both | 24359.07(27359.38,21719.51) | 11943.93(14646.53,10091.19) | -0.51 | 735.43(839.61,646.66) | 390.72(483.54,320.13) | -2.22(-2.51,-1.92) |
| Lebanon | both | 25423.24(30184.48,21229.51) | 44044.18(51407.04,36700.65) | 0.73 | 1053.00(1241.34,886.82) | 850.36(992.94,708.08) | -0.64(-0.71,-0.57) |
| Lesotho | both | 29104.42(40315.99,21004.33) | 35313.95(45969.32,26312.16) | 0.21 | 2728.92(3813.15,1963.29) | 2607.47(3409.82,1964.63) | 0.31(0.10,0.52) |
| Liberia | both | 18433.31(22312.60,15054.56) | 25994.11(33146.59,20577.22) | 0.41 | 1215.79(1422.74,1037.37) | 898.33(1121.09,723.65) | -1.12(-1.23,-1.01) |
| Libya | both | 23960.75(28953.65,19677.97) | 48709.85(57385.29,40075.78) | 1.03 | 967.42(1157.98,794.64) | 882.12(1036.09,730.68) | -0.26(-0.37,-0.15) |
| Lithuania | both | 40018.67(43651.44,35097.31) | 19809.23(23647.97,16838.28) | -0.51 | 914.05(1001.67,800.73) | 423.71(510.45,353.00) | -2.82(-2.98,-2.66) |
| Luxembourg | both | 5580.22(6450.99,4858.71) | 6909.82(8100.26,5878.36) | 0.24 | 1126.21(1321.33,963.29) | 787.26(952.64,652.59) | -1.46(-1.53,-1.39) |
| North Macedonia | both | 20215.02(23348.73,17496.92) | 20062.78(24067.69,16658.09) | -0.01 | 1122.86(1287.27,969.96) | 721.70(866.69,597.09) | -1.77(-1.88,-1.66) |
| Madagascar | both | 225171.15(260405.28,195309.59) | 310879.38(378781.59,251714.91) | 0.38 | 2548.77(2875.95,2271.12) | 1931.12(2324.51,1584.73) | -1.14(-1.24,-1.03) |
| Malawi | both | 88967.24(109066.29,71313.04) | 117158.91(139292.87,97770.89) | 0.32 | 1451.86(1668.23,1222.93) | 1109.58(1277.91,941.12) | -1.06(-1.15,-0.96) |
| Malaysia | both | 159245.94(179175.67,136870.66) | 243721.39(295877.37,204325.64) | 0.53 | 1589.83(1807.18,1359.52) | 923.05(1122.18,779.27) | -2.55(-2.85,-2.26) |
| Maldives | both | 3766.13(4515.10,2882.19) | 3931.91(4498.90,3422.40) | 0.04 | 3295.06(3753.12,2643.55) | 1251.62(1428.30,1093.22) | -3.77(-3.99,-3.55) |
| Mali | both | 104713.81(120526.84,87109.74) | 185662.62(231442.73,146047.86) | 0.77 | 2117.31(2446.72,1648.62) | 1666.14(2063.31,1286.29) | -0.83(-0.93,-0.73) |
| Malta | both | 3866.22(4566.52,3278.18) | 4922.68(5695.57,4167.04) | 0.27 | 944.24(1120.60,799.33) | 674.06(827.54,547.47) | -1.35(-1.40,-1.29) |
| Marshall Islands | both | 762.51(996.77,632.81) | 931.90(1204.38,699.46) | 0.22 | 3607.81(4816.12,2957.83) | 2437.95(3132.91,1843.70) | -1.35(-1.42,-1.28) |
| Mauritania | both | 20763.70(24605.73,16461.94) | 25241.14(30321.01,20275.89) | 0.22 | 1671.97(1971.37,1311.74) | 953.71(1132.57,781.65) | -1.83(-2.02,-1.65) |
| Mauritius | both | 14010.08(15205.24,12687.84) | 14833.37(17740.22,12561.77) | 0.06 | 1800.01(1940.25,1627.79) | 959.50(1145.42,811.31) | -2.35(-2.49,-2.21) |
| Mexico | both | 539408.58(617994.26,478216.67) | 921454.96(1050775.93,805715.87) | 0.71 | 1045.51(1130.53,956.27) | 808.02(918.80,707.27) | -1.05(-1.15,-0.94) |
| Republic of Moldova | both | 54404.35(59367.62,47048.09) | 27287.30(32011.78,23471.70) | -0.50 | 1245.52(1357.83,1069.54) | 529.53(623.45,451.13) | -3.44(-3.77,-3.12) |
| Mongolia | both | 17186.78(20015.15,13526.03) | 16174.89(20590.58,13254.52) | -0.06 | 1331.79(1543.38,1090.99) | 672.17(841.33,555.91) | -3.07(-3.37,-2.77) |
| Montenegro | both | 2222.14(2703.77,1830.33) | 2994.86(3559.07,2477.57) | 0.35 | 364.47(444.60,300.80) | 374.22(459.92,302.81) | 0.15(0.10,0.20) |
| Morocco | both | 172070.34(209674.16,141596.59) | 297875.92(353539.83,247894.29) | 0.73 | 1082.32(1337.96,904.65) | 965.09(1141.42,805.93) | -0.47(-0.55,-0.39) |
| Mozambique | both | 122092.00(153147.44,96499.29) | 199281.91(239082.66,162627.46) | 0.63 | 1321.68(1568.84,1117.44) | 1193.89(1410.93,992.18) | -0.26(-0.32,-0.19) |
| Myanmar | both | 1126818.19(1359950.69,893020.74) | 1120191.22(1292963.36,921733.39) | -0.01 | 4420.30(5345.80,3432.26) | 2535.23(2902.55,2080.64) | -2.11(-2.22,-2.00) |
| Namibia | both | 19574.58(25343.73,14624.40) | 24113.06(29790.74,19504.80) | 0.23 | 2449.73(3228.20,1837.77) | 1597.14(1966.11,1299.12) | -1.66(-1.86,-1.45) |
| Nepal | both | 510477.56(609172.19,404326.71) | 905564.11(1063112.75,714069.24) | 0.77 | 5600.28(6661.39,4407.04) | 4339.27(5078.79,3410.62) | -0.80(-0.97,-0.63) |
| Netherlands | both | 206629.90(237410.42,181190.27) | 291490.45(329971.68,249107.75) | 0.41 | 1093.86(1285.22,946.83) | 976.53(1135.57,815.97) | -0.57(-0.70,-0.44) |
| New Zealand | both | 57478.96(67609.03,49252.86) | 63773.73(71425.85,56092.61) | 0.11 | 1535.21(1829.50,1303.38) | 947.62(1101.44,813.70) | -2.01(-2.14,-1.87) |
| Nicaragua | both | 25930.60(32308.20,20646.41) | 37819.11(44772.67,30981.00) | 0.46 | 856.12(987.85,737.55) | 824.65(955.90,678.40) | -0.08(-0.25,0.09) |
| Niger | both | 90144.59(110149.85,71839.16) | 170826.98(219775.64,132507.64) | 0.90 | 2017.40(2505.16,1613.68) | 1425.37(1841.05,1114.92) | -1.10(-1.27,-0.92) |
| Nigeria | both | 745808.50(886421.04,617825.83) | 1176176.01(1399369.42,972553.16) | 0.58 | 1323.09(1569.69,1105.61) | 966.64(1134.86,804.95) | -1.06(-1.11,-1.01) |
| Democratic People's Republic of Korea | both | 492235.43(607475.35,373486.49) | 720735.47(834076.87,597373.06) | 0.46 | 3444.24(4285.68,2634.15) | 2369.28(2733.79,1964.03) | -1.39(-1.51,-1.27) |
| Northern Mariana Islands | both | 437.79(519.81,359.04) | 515.94(619.08,441.53) | 0.18 | 1875.40(2165.69,1642.43) | 1166.72(1374.94,1009.36) | -1.62(-1.66,-1.57) |
| Norway | both | 58177.15(68756.94,48807.74) | 73970.95(83737.97,60675.62) | 0.27 | 981.69(1202.82,805.77) | 889.16(1049.00,718.54) | -0.41(-0.54,-0.28) |
| Oman | both | 8126.22(10228.80,6543.00) | 16301.97(20686.43,13092.22) | 1.01 | 913.67(1173.12,721.46) | 683.99(800.27,569.69) | -0.73(-0.87,-0.59) |
| Pakistan | both | 1678454.60(2015330.91,1419797.69) | 2466318.53(2917860.98,2104167.43) | 0.47 | 2847.06(3463.67,2384.80) | 2228.12(2641.90,1901.41) | -1.00(-1.22,-0.78) |
| Palestine | both | 12579.11(15554.44,10209.79) | 22688.26(27095.25,18987.32) | 0.80 | 1146.34(1421.36,938.80) | 768.90(877.97,661.91) | -1.56(-1.67,-1.45) |
| Panama | both | 15166.86(18218.97,12684.08) | 24815.86(29883.41,20028.41) | 0.64 | 766.51(888.65,672.00) | 596.38(718.21,480.84) | -1.12(-1.25,-0.99) |
| Papua New Guinea | both | 110460.71(132701.71,91952.08) | 227960.88(289239.48,181012.03) | 1.06 | 5253.32(6225.41,4384.20) | 4452.56(5534.57,3566.00) | -0.49(-0.56,-0.43) |
| Paraguay | both | 20351.43(25785.98,16530.84) | 40774.39(50703.10,32439.34) | 1.00 | 626.18(755.77,535.51) | 676.13(828.95,541.35) | 0.41(0.27,0.56) |
| Peru | both | 180500.96(219116.08,148121.76) | 198154.85(246443.41,156432.29) | 0.10 | 920.39(1088.71,783.36) | 607.61(758.05,478.38) | -1.20(-1.37,-1.03) |
| Philippines | both | 822277.84(929023.16,722434.11) | 1304851.45(1507924.65,1126317.30) | 0.59 | 2083.82(2306.83,1858.20) | 1526.09(1767.27,1326.46) | -0.98(-1.08,-0.88) |
| Poland | both | 514264.98(590217.46,447900.51) | 378423.91(440435.04,323377.14) | -0.26 | 1211.04(1400.49,1046.79) | 650.55(782.91,541.22) | -2.30(-2.47,-2.14) |
| Portugal | both | 154174.21(180963.91,132584.42) | 167667.82(194772.76,145032.80) | 0.09 | 1234.74(1471.14,1047.71) | 860.68(1063.77,706.23) | -1.31(-1.41,-1.20) |
| Puerto Rico | both | 37074.52(44825.87,30797.83) | 42567.73(51182.87,35116.44) | 0.15 | 1032.67(1254.03,856.90) | 832.26(1035.07,658.67) | -1.19(-1.40,-0.98) |
| Qatar | both | 1686.40(2046.47,1380.41) | 8290.96(10538.22,6537.00) | 3.92 | 874.35(1100.32,731.70) | 616.18(760.84,512.26) | -1.32(-1.48,-1.17) |
| Romania | both | 366550.32(400441.11,317074.99) | 223367.19(265123.36,189887.84) | -0.39 | 1392.70(1527.60,1188.92) | 713.51(851.18,601.51) | -2.40(-2.59,-2.22) |
| Russian Federation | both | 1769632.15(1961919.32,1520141.32) | 1110123.96(1280630.70,973894.29) | -0.37 | 1016.24(1135.89,866.32) | 534.80(621.45,465.43) | -2.94(-3.22,-2.66) |
| Rwanda | both | 138387.09(169594.12,107693.72) | 128813.56(162327.35,102493.53) | -0.07 | 2989.74(3553.83,2395.21) | 1572.67(1948.23,1290.79) | -2.80(-3.02,-2.58) |
| Saint Lucia | both | 1203.91(1426.50,1017.11) | 1827.92(2126.25,1536.08) | 0.52 | 1093.55(1249.78,951.62) | 954.60(1128.33,793.38) | -0.79(-0.92,-0.65) |
| Saint Vincent and the Grenadines | both | 575.47(731.17,460.78) | 726.99(863.01,607.74) | 0.26 | 593.12(714.73,492.68) | 610.30(731.12,502.46) | -0.07(-0.17,0.03) |
| Samoa | both | 2983.65(3843.01,2384.93) | 2815.26(3520.73,2259.34) | -0.06 | 3068.12(3951.06,2469.57) | 1840.93(2277.71,1493.79) | -1.85(-1.98,-1.73) |
| Sao Tome and Principe | both | 1964.80(2278.80,1629.70) | 2747.26(3281.10,2226.82) | 0.40 | 2594.99(2968.34,2162.70) | 2295.20(2720.41,1849.34) | -0.52(-0.64,-0.39) |
| Saudi Arabia | both | 87020.63(110234.64,68644.87) | 181823.55(214761.33,152169.93) | 1.09 | 1215.53(1598.42,950.91) | 864.42(998.32,740.51) | -1.22(-1.26,-1.17) |
| Senegal | both | 72389.93(84503.20,58186.00) | 106791.06(128947.93,87089.38) | 0.48 | 1708.16(2007.53,1377.95) | 1158.57(1377.87,957.28) | -1.07(-1.33,-0.81) |
| Serbia | both | 96148.81(108583.71,85697.31) | 102235.80(120279.18,86318.28) | 0.06 | 901.21(1024.60,802.27) | 720.34(845.25,610.25) | -0.94(-1.07,-0.81) |
| Seychelles | both | 702.36(770.92,621.33) | 950.19(1060.96,841.17) | 0.35 | 1182.94(1290.33,1041.78) | 909.81(1017.67,805.02) | -1.13(-1.24,-1.03) |
| Sierra Leone | both | 41776.91(50832.55,33167.67) | 64602.40(79811.40,51089.18) | 0.55 | 1729.06(2077.61,1381.10) | 1305.50(1607.99,1052.30) | -0.68(-0.85,-0.52) |
| Singapore | both | 31581.35(35904.29,27406.71) | 30287.53(36181.00,25413.21) | -0.04 | 1410.44(1567.27,1236.58) | 447.81(541.34,368.68) | -4.23(-4.52,-3.95) |
| Slovakia | both | 33221.45(42386.50,29195.70) | 37384.76(44630.57,31285.67) | 0.13 | 579.56(726.19,505.63) | 476.27(569.09,394.83) | -0.44(-0.54,-0.33) |
| Slovenia | both | 20063.82(23905.19,16557.29) | 17806.89(21158.08,14868.99) | -0.11 | 863.78(1029.94,710.20) | 505.38(613.37,407.20) | -2.32(-2.51,-2.13) |
| Solomon Islands | both | 6510.93(8114.16,5154.51) | 11721.07(14034.07,9641.97) | 0.80 | 4028.39(5090.34,3174.83) | 3335.25(3951.13,2738.79) | -0.59(-0.64,-0.55) |
| Somalia | both | 103475.73(138014.33,73130.56) | 194652.91(280217.73,143795.09) | 0.88 | 2513.10(3481.53,1837.87) | 1863.13(2767.97,1356.91) | -0.97(-1.05,-0.89) |
| South Africa | both | 479227.04(534390.85,426106.14) | 623481.14(700309.96,561739.31) | 0.30 | 1880.02(2092.35,1677.76) | 1345.32(1499.14,1214.69) | -1.45(-1.90,-1.01) |
| Republic of Korea | both | 323117.32(361754.53,273813.00) | 421654.58(477126.53,367032.54) | 0.30 | 1203.64(1332.47,961.40) | 559.69(651.98,476.76) | -3.05(-3.21,-2.88) |
| South Sudan | both | 65003.34(83047.68,46298.36) | 65323.51(83601.87,51925.53) | 0.00 | 1708.18(2104.18,1266.90) | 1179.60(1501.67,927.38) | -1.33(-1.45,-1.20) |
| Spain | both | 536663.84(587746.50,490051.43) | 677880.68(757799.27,594318.60) | 0.26 | 1037.80(1153.47,939.03) | 752.27(863.05,648.90) | -1.30(-1.38,-1.23) |
| Sri Lanka | both | 257720.94(279937.54,227927.81) | 327288.70(405214.81,255635.14) | 0.27 | 2338.14(2527.79,2057.33) | 1393.98(1714.16,1090.84) | -1.55(-1.68,-1.42) |
| Sudan | both | 239122.89(309440.29,171884.81) | 288028.68(365973.42,218411.75) | 0.20 | 1866.38(2410.56,1321.64) | 1206.90(1549.02,905.47) | -1.59(-1.67,-1.51) |
| Suriname | both | 2688.63(3229.13,2238.35) | 4272.57(5067.84,3553.87) | 0.59 | 834.49(969.07,713.01) | 741.60(882.15,614.33) | -0.61(-0.76,-0.45) |
| Eswatini | both | 9669.49(12233.46,7644.24) | 12686.84(15713.98,10224.69) | 0.31 | 2567.68(3346.08,2024.54) | 1865.46(2300.50,1513.24) | -0.95(-1.26,-0.64) |
| Sweden | both | 112147.52(136021.26,92087.61) | 134876.52(156084.33,112001.92) | 0.20 | 903.35(1137.70,718.67) | 785.78(962.57,633.87) | -0.58(-0.68,-0.48) |
| Switzerland | both | 83693.77(96772.34,72360.02) | 92008.08(106709.53,79093.22) | 0.10 | 902.79(1080.07,761.49) | 656.39(802.01,537.07) | -1.25(-1.32,-1.18) |
| Syrian Arab Republic | both | 92338.21(109945.15,77640.49) | 121632.75(148875.52,100656.68) | 0.32 | 1230.64(1467.65,1033.36) | 987.36(1210.57,817.04) | -0.88(-1.02,-0.74) |
| Taiwan (Province of China) | both | 159745.16(178089.18,141383.10) | 200315.38(242314.25,167988.12) | 0.25 | 1054.82(1157.29,941.41) | 584.07(710.32,491.26) | -2.36(-2.49,-2.23) |
| Tajikistan | both | 47585.24(51647.45,42705.75) | 58893.73(74022.50,49440.37) | 0.24 | 1522.24(1647.89,1349.94) | 1098.22(1473.98,914.19) | -1.33(-1.50,-1.15) |
| United Republic of Tanzania | both | 221521.63(261604.59,185317.49) | 402768.59(491766.23,327202.07) | 0.82 | 1295.95(1471.33,1123.31) | 1061.49(1228.74,895.07) | -0.64(-0.68,-0.60) |
| Thailand | both | 663555.83(746388.83,575080.04) | 712557.58(856109.94,588733.34) | 0.07 | 1760.37(1980.29,1521.24) | 790.13(944.73,651.65) | -3.25(-3.43,-3.07) |
| Bahamas | both | 1328.91(1657.85,1092.06) | 2211.11(2689.50,1821.84) | 0.66 | 634.76(749.99,539.75) | 602.20(746.09,491.01) | -0.26(-0.31,-0.20) |
| Gambia | both | 8132.13(10235.36,6320.36) | 16790.53(20479.65,13418.46) | 1.06 | 1663.05(2068.73,1293.18) | 1354.49(1634.15,1087.06) | -0.68(-0.88,-0.49) |
| Timor-Leste | both | 10784.17(14335.39,7960.85) | 15501.26(18953.51,12040.29) | 0.44 | 2429.20(2982.08,1905.75) | 1780.70(2175.63,1406.14) | -1.27(-1.50,-1.04) |
| Togo | both | 32840.71(38497.12,27566.52) | 62476.16(76319.84,50227.34) | 0.90 | 1810.00(2107.14,1508.91) | 1305.42(1593.16,1067.30) | -0.98(-1.10,-0.87) |
| Tonga | both | 1079.18(1246.97,920.08) | 1066.89(1282.28,880.99) | -0.01 | 1796.27(2062.85,1541.00) | 1278.57(1523.03,1051.84) | -1.26(-1.41,-1.11) |
| Trinidad and Tobago | both | 7897.32(9093.12,6962.40) | 8976.79(11325.76,7099.38) | 0.14 | 779.45(872.79,702.49) | 583.86(732.26,457.87) | -1.17(-1.34,-1.00) |
| Tunisia | both | 52078.02(63684.83,43700.47) | 89905.42(108784.46,73277.74) | 0.73 | 912.04(1118.80,773.70) | 755.24(910.36,615.25) | -0.74(-0.77,-0.70) |
| Turkey | both | 640771.05(728334.51,552746.03) | 991743.41(1153651.91,817494.75) | 0.55 | 1606.03(1789.80,1403.96) | 1171.86(1368.26,962.55) | -0.89(-1.06,-0.73) |
| Turkmenistan | both | 29319.59(32381.13,24112.52) | 19111.84(27118.79,15424.65) | -0.35 | 1298.80(1409.21,1064.06) | 444.13(662.39,356.39) | -4.50(-4.92,-4.09) |
| Uganda | both | 191489.97(251050.85,142007.29) | 289724.81(360361.38,229299.87) | 0.51 | 1855.93(2507.30,1354.36) | 1290.38(1564.41,1037.07) | -1.58(-1.71,-1.45) |
| Ukraine | both | 908328.03(990022.53,765428.11) | 364900.22(474322.49,305131.77) | -0.60 | 1347.43(1486.14,1127.91) | 575.38(733.55,476.25) | -4.19(-4.62,-3.75) |
| United Arab Emirates | both | 19134.87(24141.15,15017.99) | 114082.28(144088.55,83258.32) | 4.96 | 2129.25(2671.37,1751.40) | 1523.29(1828.38,1249.50) | -1.25(-1.49,-1.01) |
| United Kingdom | both | 1175412.98(1346134.56,1031507.98) | 1285742.68(1444486.18,1113883.44) | 0.09 | 1484.80(1763.28,1260.16) | 1186.87(1391.89,1004.04) | -0.93(-0.98,-0.88) |
| United States of America | both | 4042277.02(4568024.63,3568362.22) | 7003710.18(7768072.13,6141549.25) | 0.73 | 1356.65(1568.64,1179.70) | 1447.79(1654.94,1244.52) | 0.37(0.31,0.42) |
| Uruguay | both | 38593.74(43782.20,34176.24) | 50256.97(55705.41,44652.02) | 0.30 | 1055.78(1207.46,931.50) | 1031.17(1167.96,898.50) | -0.19(-0.31,-0.07) |
| Uzbekistan | both | 163057.66(183275.69,136019.29) | 167293.11(212306.43,138561.81) | 0.03 | 1281.60(1421.38,1058.13) | 754.56(1032.19,626.44) | -2.86(-3.36,-2.36) |
| Vanuatu | both | 2903.09(3870.72,2092.68) | 5321.49(6809.54,4093.43) | 0.83 | 3722.66(4977.16,2699.70) | 2773.85(3561.04,2115.77) | -1.31(-1.45,-1.17) |
| Venezuela (Bolivarian Republic of) | both | 101302.30(123650.84,84035.42) | 199668.79(243276.30,162613.79) | 0.97 | 715.63(820.98,626.90) | 714.07(870.03,579.89) | -0.35(-0.51,-0.20) |
| Viet nam | both | 666814.11(817378.62,354562.35) | 1006628.63(1204734.32,595014.43) | 0.51 | 1568.66(1931.88,775.89) | 1152.99(1371.06,671.78) | -1.07(-1.12,-1.01) |
| Virginia | both | 94498.36(107859.10,83338.22) | 173932.60(200181.45,147882.26) | 0.84 | 1394.28(1610.41,1209.33) | 1409.67(1656.05,1175.59) | 0.10(0.04,0.17) |
| Yemen | both | 134978.16(175241.45,101601.46) | 232324.80(284441.59,190069.24) | 0.72 | 1995.27(2689.31,1467.33) | 1372.07(1678.74,1130.82) | -1.42(-1.51,-1.32) |
| Zambia | both | 70834.57(87067.34,57194.27) | 109753.87(129345.44,91801.14) | 0.55 | 1561.05(1823.62,1327.53) | 1161.24(1366.06,981.02) | -1.20(-1.35,-1.06) |
| Zimbabwe | both | 66984.02(75033.85,58725.63) | 112702.71(139198.91,88551.89) | 0.68 | 1388.72(1544.58,1217.15) | 1365.33(1683.98,1060.79) | 0.28(0.13,0.43) |
| Monaco | both | 428.62(496.38,363.51) | 525.38(602.43,450.09) | 0.23 | 751.48(907.32,618.14) | 701.14(845.54,575.07) | -0.16(-0.21,-0.10) |
| San Marino | both | 200.29(236.79,167.36) | 333.93(407.07,270.55) | 0.67 | 678.23(815.46,559.29) | 631.29(782.23,502.09) | -0.15(-0.22,-0.08) |
| Saint Kitts and Nevis | both | 324.57(383.88,278.15) | 424.26(505.01,347.00) | 0.31 | 825.22(954.63,715.45) | 715.49(865.02,583.67) | -0.54(-0.65,-0.43) |
| Cook Islands | both | 250.64(283.07,218.44) | 236.86(272.26,205.93) | -0.05 | 1858.19(2078.91,1627.80) | 1056.56(1218.17,910.17) | -2.00(-2.14,-1.87) |
| Nauru | both | 187.40(229.97,151.39) | 137.19(168.59,104.45) | -0.27 | 3628.07(4377.91,2957.84) | 2473.76(2977.84,1933.43) | -1.38(-1.70,-1.07) |
| Niue | both | 53.50(63.38,44.70) | 32.02(38.52,26.26) | -0.40 | 2399.83(2868.90,2005.67) | 1587.23(1913.78,1298.13) | -1.62(-1.70,-1.55) |
| Palau | both | 390.61(481.84,317.90) | 511.72(617.66,420.92) | 0.31 | 3630.02(4442.41,2999.85) | 2618.33(3131.23,2176.05) | -1.06(-1.13,-0.99) |
| Tokelau | both | 41.71(50.35,34.50) | 21.91(26.58,17.93) | -0.47 | 3000.02(3658.85,2467.41) | 1679.38(2043.15,1375.06) | -2.08(-2.15,-2.02) |
| Tuvalu | both | 300.94(364.80,248.56) | 221.28(275.83,178.74) | -0.26 | 4146.30(5071.49,3403.65) | 2176.63(2704.82,1760.30) | -2.29(-2.42,-2.16) |
| Afghanistan | male | 96706.72(116010.54,78573.20) | 136633.44(163879.06,110624.72) | 0.41 | 2383.67(2860.62,1936.37) | 1604.47(1888.31,1274.36) | -1.51(-1.75,-1.27) |
| Albania | male | 15875.77(17550.89,12780.65) | 12540.34(15595.05,10054.49) | -0.21 | 1765.26(1932.66,1349.44) | 689.31(842.74,557.72) | -3.54(-4.00,-3.07) |
| Algeria | male | 87737.81(109639.29,69820.92) | 143259.11(174446.41,117752.35) | 0.63 | 1282.08(1602.21,1017.61) | 836.90(1019.90,691.65) | -1.57(-1.65,-1.49) |
| American Samoa | male | 343.63(389.02,298.47) | 373.38(440.28,320.12) | 0.09 | 2680.49(3005.94,2337.78) | 1644.83(1918.46,1423.49) | -1.86(-1.94,-1.78) |
| Andorra | male | 366.52(451.21,298.39) | 630.41(744.89,523.77) | 0.72 | 1386.81(1691.90,1143.72) | 1011.75(1195.24,840.55) | -1.16(-1.23,-1.09) |
| Angola | male | 57832.22(72578.93,44362.20) | 86169.68(104139.42,70268.00) | 0.49 | 2141.35(2637.31,1668.55) | 1244.58(1485.56,1044.34) | -2.08(-2.18,-1.99) |
| Antigua and Barbuda | male | 153.95(192.05,126.61) | 222.04(271.97,178.26) | 0.44 | 591.45(712.25,498.17) | 542.89(674.96,428.44) | -0.32(-0.46,-0.17) |
| Argentina | male | 196278.17(219241.53,176314.32) | 277615.54(315910.89,244765.24) | 0.41 | 1351.86(1497.13,1219.95) | 1220.78(1391.71,1072.21) | -0.63(-0.80,-0.45) |
| Armenia | male | 18344.45(19805.10,17010.73) | 16097.61(18864.04,13711.48) | -0.12 | 1668.17(1793.38,1557.16) | 974.49(1133.92,832.21) | -2.36(-2.54,-2.17) |
| Australia | male | 139238.39(156429.69,123096.48) | 163353.06(186129.17,142761.97) | 0.17 | 1670.61(1888.06,1470.42) | 987.81(1175.69,830.30) | -2.12(-2.27,-1.96) |
| Austria | male | 45133.49(51433.61,39923.96) | 52787.27(60196.62,45856.01) | 0.17 | 1054.65(1208.00,929.72) | 770.99(907.11,652.87) | -1.08(-1.14,-1.02) |
| Azerbaijan | male | 33165.10(36311.77,30032.40) | 36810.82(47824.01,30151.43) | 0.11 | 1491.21(1620.49,1359.85) | 938.24(1243.92,763.80) | -2.21(-2.71,-1.71) |
| Bahrain | male | 1951.62(2243.23,1692.93) | 4355.25(5220.74,3627.68) | 1.23 | 1909.66(2167.10,1659.66) | 900.70(1067.24,764.31) | -2.87(-3.30,-2.44) |
| Bangladesh | male | 1145850.67(1305981.18,997679.54) | 1125278.30(1669542.21,914320.16) | -0.02 | 4231.89(4880.54,3697.34) | 1683.12(2532.02,1370.93) | -3.34(-3.57,-3.11) |
| Barbados | male | 838.76(1016.03,695.41) | 1112.00(1325.25,915.31) | 0.33 | 687.34(838.34,569.02) | 651.96(811.03,523.47) | -0.49(-0.64,-0.34) |
| Belarus | male | 95976.49(104831.27,87008.89) | 43176.56(53610.39,35238.03) | -0.55 | 2082.23(2263.09,1890.05) | 789.50(974.14,648.04) | -4.37(-4.86,-3.89) |
| Belgium | male | 109659.46(119335.72,100397.52) | 98335.67(110059.72,87691.44) | -0.10 | 1797.56(1969.84,1643.07) | 1053.20(1197.17,929.03) | -2.17(-2.28,-2.06) |
| Belize | male | 704.16(864.67,574.70) | 1934.56(2297.27,1628.70) | 1.75 | 929.81(1079.96,793.82) | 1190.57(1394.20,1010.56) | 0.48(0.08,0.87) |
| Benin | male | 27262.86(32179.93,22141.90) | 45342.23(58053.10,35754.34) | 0.66 | 2172.01(2556.32,1733.53) | 1405.76(1776.79,1106.59) | -1.34(-1.45,-1.22) |
| Bermuda | male | 221.74(257.22,191.76) | 292.99(344.66,243.85) | 0.32 | 854.03(997.81,734.79) | 688.59(843.22,562.72) | -0.67(-0.73,-0.60) |
| Bhutan | male | 4251.04(5853.96,3045.87) | 6242.56(9322.68,4578.76) | 0.47 | 3502.93(4821.37,2557.79) | 2278.55(3408.15,1674.74) | -1.42(-1.49,-1.36) |
| Bolivia (Plurinational State of) | male | 43602.40(56865.10,31822.71) | 49171.20(60598.97,39112.57) | 0.13 | 1740.58(2108.97,1372.25) | 1117.87(1368.79,878.68) | -1.54(-1.66,-1.43) |
| Bosnia and Herzegovina | male | 21350.11(24048.93,18853.78) | 20278.66(23994.78,16920.88) | -0.05 | 1259.10(1390.34,1123.56) | 874.62(1036.97,725.63) | -1.45(-1.57,-1.32) |
| Botswana | male | 9168.11(11906.88,7038.34) | 13368.67(16904.19,10255.17) | 0.46 | 3251.87(4256.28,2504.58) | 2128.91(2679.27,1667.53) | -2.06(-2.56,-1.55) |
| Brazil | male | 789478.88(908360.41,703982.61) | 1064707.67(1188236.41,960312.50) | 0.35 | 1678.02(1832.24,1546.24) | 1052.00(1180.24,944.30) | -2.02(-2.19,-1.85) |
| Brunei Darussalam | male | 1330.81(1512.29,1121.49) | 1766.77(2053.79,1512.21) | 0.33 | 3440.05(3857.85,2641.03) | 1809.08(2053.12,1592.37) | -2.03(-2.32,-1.74) |
| Bulgaria | male | 71479.73(78157.48,64883.73) | 49262.59(59742.44,40340.95) | -0.31 | 1328.27(1456.22,1205.50) | 902.86(1087.16,747.06) | -1.65(-1.80,-1.50) |
| Burkina Faso | male | 34127.01(41433.87,27425.31) | 65540.82(80035.89,52831.72) | 0.92 | 1246.63(1457.76,1034.51) | 1073.45(1275.04,888.31) | -0.57(-0.73,-0.41) |
| Burundi | male | 50142.77(63642.54,36569.37) | 58037.45(74020.27,44792.51) | 0.16 | 3253.13(4021.94,2441.27) | 1904.80(2412.24,1465.28) | -2.07(-2.17,-1.96) |
| Cambodia | male | 56025.89(65660.17,47009.52) | 89054.80(103735.84,73384.86) | 0.59 | 2391.57(2801.34,2011.99) | 1821.17(2102.52,1512.64) | -0.96(-1.01,-0.91) |
| Cameroon | male | 54097.09(64296.34,43864.89) | 106597.43(132319.21,81592.90) | 0.97 | 2051.16(2452.02,1633.93) | 1383.74(1704.08,1072.63) | -1.25(-1.34,-1.16) |
| Canada | male | 155871.30(174154.45,139634.37) | 227656.41(256898.89,199895.18) | 0.46 | 1152.84(1293.76,1030.00) | 820.12(952.42,709.73) | -1.37(-1.44,-1.29) |
| Cabo Verde | male | 2113.34(2394.21,1497.69) | 1959.91(2468.27,1669.09) | -0.07 | 1976.43(2226.69,1366.69) | 928.32(1182.64,799.08) | -2.93(-3.51,-2.35) |
| Central African Republic | male | 21292.47(26737.64,16027.29) | 32573.03(42353.33,23308.16) | 0.53 | 3143.52(3952.62,2336.30) | 2576.70(3285.79,1838.00) | -0.71(-0.75,-0.67) |
| Chad | male | 34752.86(46783.36,27500.03) | 66966.47(83313.64,50477.05) | 0.93 | 2040.12(2846.45,1581.36) | 1586.59(1989.96,1190.06) | -0.74(-0.79,-0.69) |
| Chile | male | 50222.31(57532.66,44496.96) | 79613.93(90713.47,69799.72) | 0.59 | 1064.28(1190.37,959.55) | 796.04(914.49,694.20) | -0.81(-0.92,-0.69) |
| China | male | 15428959.68(17938478.37,11690964.07) | 12757676.55(15031868.34,10792960.03) | -0.17 | 4518.35(5188.17,3522.71) | 1601.39(1870.73,1366.68) | -3.82(-3.97,-3.66) |
| Colombia | male | 118656.43(137890.46,104248.46) | 199921.76(244540.18,162757.59) | 0.68 | 1179.70(1301.34,1067.85) | 851.90(1045.18,693.67) | -1.46(-1.64,-1.28) |
| Comoros | male | 2727.66(3530.08,1728.29) | 3176.33(3880.28,2551.32) | 0.16 | 2017.23(2579.76,1315.21) | 1286.69(1574.00,1029.40) | -1.77(-1.97,-1.57) |
| Congo | male | 15272.19(18788.18,11215.18) | 20117.19(24728.68,14568.56) | 0.32 | 2579.19(3178.83,1889.43) | 1362.18(1643.33,953.16) | -2.51(-2.66,-2.35) |
| Costa Rica | male | 9490.13(11717.20,7822.96) | 18092.74(21797.06,14597.10) | 0.91 | 858.62(995.91,750.20) | 805.24(971.74,651.71) | -0.74(-1.14,-0.34) |
| Côte d'Ivoire | male | 63410.04(75325.18,51067.44) | 100947.49(124641.91,79500.24) | 0.59 | 2167.93(2579.98,1728.62) | 1360.60(1677.31,1073.54) | -1.64(-1.70,-1.57) |
| Croatia | male | 26088.11(29153.52,23292.21) | 28177.37(33210.27,23402.74) | 0.08 | 1069.96(1195.14,955.65) | 848.42(996.00,707.73) | -0.80(-0.88,-0.73) |
| Cuba | male | 39302.97(47298.63,33319.31) | 65868.39(79756.60,54895.95) | 0.68 | 761.01(919.67,644.52) | 892.86(1096.36,726.38) | 0.41(0.29,0.54) |
| Cyprus | male | 4625.32(5355.41,3959.03) | 7805.17(9014.77,6600.91) | 0.69 | 1329.05(1520.43,1139.97) | 961.20(1122.76,808.78) | -1.37(-1.51,-1.23) |
| Czechia | male | 60896.36(66093.09,56142.76) | 67214.87(78885.05,56351.42) | 0.10 | 1118.15(1218.16,1026.64) | 810.15(948.55,678.52) | -0.43(-0.81,-0.05) |
| Democratic Republic of the Congo | male | 201536.05(256483.99,148382.43) | 339843.86(432512.07,240958.57) | 0.69 | 2104.14(2615.66,1529.79) | 1709.36(2182.62,1161.27) | -0.74(-0.84,-0.64) |
| Denmark | male | 49219.52(53780.66,44994.29) | 52010.70(57913.02,45960.42) | 0.06 | 1476.52(1639.39,1337.92) | 1067.68(1209.04,934.66) | -1.37(-1.49,-1.24) |
| Djibouti | male | 2102.78(2840.66,1561.47) | 4473.99(6169.71,3335.03) | 1.13 | 1688.97(2244.40,1226.57) | 1135.46(1545.96,845.58) | -1.56(-1.63,-1.49) |
| Dominica | male | 349.09(424.22,295.49) | 418.87(503.86,336.39) | 0.20 | 1065.08(1281.91,915.01) | 1083.61(1313.29,864.71) | -0.01(-0.10,0.09) |
| Dominican Republic | male | 25217.36(30782.20,20124.28) | 35692.77(46944.85,27077.66) | 0.42 | 823.51(960.05,703.35) | 745.55(981.44,570.31) | 0.16(-0.07,0.38) |
| Ecuador | male | 41816.83(50157.40,33731.40) | 52416.59(64535.26,41946.78) | 0.25 | 1003.59(1137.29,869.95) | 729.49(889.24,593.70) | -0.63(-1.04,-0.23) |
| Egypt | male | 316896.24(355958.92,279572.62) | 519403.60(645090.72,400312.64) | 0.64 | 1728.59(1917.58,1554.98) | 1380.25(1719.14,1058.10) | -0.66(-0.74,-0.58) |
| El Salvador | male | 27363.37(33827.76,21944.36) | 19077.45(23579.05,15150.90) | -0.30 | 1206.40(1408.20,1021.00) | 696.69(851.63,557.10) | -2.02(-2.33,-1.72) |
| Equatorial Guinea | male | 2938.35(3827.88,2089.09) | 3565.50(4580.03,2581.52) | 0.21 | 2733.84(3565.50,1922.21) | 1171.88(1499.04,795.48) | -3.38(-3.77,-2.99) |
| Eritrea | male | 19909.55(25701.48,13373.84) | 29332.61(37104.83,22464.47) | 0.47 | 2838.32(3724.33,1778.65) | 1795.90(2248.38,1365.47) | -1.72(-1.80,-1.63) |
| Estonia | male | 6382.32(6989.78,5776.41) | 4262.11(5207.02,3487.50) | -0.33 | 858.33(937.62,778.93) | 482.75(584.51,395.01) | -2.18(-2.34,-2.02) |
| Ethiopia | male | 311258.85(365928.60,253056.64) | 315433.68(375685.00,262873.85) | 0.01 | 2268.17(2720.32,1850.45) | 1133.33(1335.07,951.28) | -2.58(-2.67,-2.49) |
| Micronesia (Federated States of) | male | 1317.37(1625.86,1042.18) | 1006.00(1290.35,719.05) | -0.24 | 4738.95(5905.72,3661.18) | 2859.85(3582.39,2146.14) | -1.86(-1.98,-1.75) |
| Fiji | male | 7835.80(9381.10,6382.58) | 7379.86(9201.07,5905.80) | -0.06 | 3981.90(4752.97,3261.49) | 2201.10(2694.86,1791.07) | -2.61(-2.91,-2.32) |
| Finland | male | 27713.80(31388.53,24511.98) | 34418.20(39234.38,30402.74) | 0.24 | 1046.33(1189.34,921.36) | 733.33(866.78,628.95) | -1.28(-1.34,-1.22) |
| France | male | 353065.63(409626.94,305105.20) | 299883.03(347275.60,258159.32) | -0.15 | 1108.85(1290.96,951.33) | 612.72(733.88,508.01) | -2.35(-2.56,-2.14) |
| Gabon | male | 5336.64(6347.27,4218.57) | 6277.11(8226.46,4759.49) | 0.18 | 1894.56(2255.26,1487.53) | 1179.72(1574.84,851.15) | -1.69(-1.76,-1.62) |
| Georgia | male | 20453.49(23207.65,18160.68) | 19423.60(23365.45,16299.98) | -0.05 | 861.74(966.70,774.53) | 880.83(1048.10,745.52) | 1.05(0.60,1.51) |
| Germany | male | 674894.77(745870.65,608436.02) | 643063.42(720572.01,565020.10) | -0.05 | 1466.51(1625.13,1321.53) | 848.21(962.70,740.49) | -1.80(-2.10,-1.50) |
| Ghana | male | 72125.87(88319.94,45044.57) | 155395.95(191251.69,97651.25) | 1.15 | 1895.90(2341.30,1128.17) | 1789.49(2201.10,1076.40) | 0.34(0.13,0.54) |
| Greece | male | 48119.42(54699.37,41756.65) | 77424.09(86727.72,67478.84) | 0.61 | 757.75(878.38,652.62) | 794.97(918.29,685.30) | 0.79(0.54,1.03) |
| Greenland | male | 343.87(400.68,290.72) | 432.79(502.93,357.59) | 0.26 | 2039.43(2314.53,1763.41) | 1332.11(1535.85,1100.72) | -1.57(-1.66,-1.49) |
| Grenada | male | 369.37(442.92,308.99) | 424.27(509.73,358.45) | 0.15 | 1015.20(1186.67,867.50) | 857.73(1032.20,723.08) | -0.52(-0.91,-0.12) |
| Guam | male | 673.66(777.10,579.84) | 883.13(1050.70,738.83) | 0.31 | 1647.43(1862.52,1453.11) | 1008.08(1192.61,848.13) | -1.80(-1.95,-1.65) |
| Guatemala | male | 49072.03(57365.35,41932.96) | 40999.70(49997.33,32979.14) | -0.16 | 1423.54(1621.12,1239.96) | 679.72(820.62,558.56) | -2.88(-3.36,-2.39) |
| Guinea | male | 40066.83(49232.02,31846.03) | 61059.84(76452.72,47340.89) | 0.52 | 1964.88(2530.33,1554.24) | 1686.61(2062.63,1301.72) | -0.15(-0.31,0.00) |
| Guinea-Bissau | male | 8577.01(10783.99,6631.69) | 9312.11(11773.19,6994.91) | 0.09 | 3387.34(4217.69,2618.37) | 2055.09(2571.97,1546.89) | -1.46(-1.66,-1.26) |
| Guyana | male | 2800.94(3378.52,2291.36) | 3220.09(3964.43,2550.69) | 0.15 | 1069.19(1254.43,899.44) | 987.50(1197.30,795.12) | -0.27(-0.36,-0.17) |
| Haiti | male | 69690.80(97088.74,38713.73) | 82360.46(109891.49,52730.73) | 0.18 | 2632.44(3415.70,1618.08) | 1829.44(2430.62,1159.59) | -1.05(-1.20,-0.89) |
| Honduras | male | 29228.72(35101.42,23610.60) | 47930.65(64527.41,35218.60) | 0.64 | 1625.45(1917.12,1319.97) | 1538.08(2105.05,1067.14) | -0.05(-0.22,0.13) |
| Hungary | male | 92393.23(99202.72,85985.27) | 87159.98(101769.04,74370.04) | -0.06 | 1610.83(1735.29,1492.98) | 1220.73(1419.81,1045.50) | -0.78(-1.06,-0.50) |
| Iceland | male | 1198.53(1426.23,1005.40) | 1632.50(1918.29,1388.04) | 0.36 | 935.53(1119.23,779.14) | 716.39(865.16,593.27) | -0.97(-1.07,-0.87) |
| India | male | 9844753.29(11168582.94,8161291.75) | 15702214.77(18543130.54,12662322.59) | 0.59 | 4505.99(5128.16,3660.58) | 2959.08(3498.71,2373.83) | -1.45(-1.54,-1.35) |
| Indonesia | male | 1302028.70(1473671.57,1107514.05) | 1993676.78(2342774.70,1658437.39) | 0.53 | 2491.72(2831.03,2103.61) | 2041.22(2368.15,1720.93) | -0.52(-0.62,-0.42) |
| Iran (Islamic Republic of) | male | 206040.82(241937.53,178518.97) | 334962.82(374789.21,296527.44) | 0.63 | 1241.64(1472.08,1100.77) | 898.61(997.75,801.82) | -1.08(-1.12,-1.05) |
| Iraq | male | 61025.22(73552.51,49652.49) | 92373.71(113016.61,74228.58) | 0.51 | 1138.11(1374.62,931.18) | 652.18(777.12,533.51) | -2.39(-2.58,-2.21) |
| Ireland | male | 36254.98(40502.48,32685.12) | 33076.68(37677.79,29104.67) | -0.09 | 2006.30(2239.44,1805.60) | 1042.37(1209.87,903.83) | -2.62(-2.79,-2.44) |
| Israel | male | 23414.55(27176.49,20531.10) | 34135.56(40002.58,29190.83) | 0.46 | 1038.13(1199.61,916.57) | 678.75(800.73,579.85) | -1.63(-1.74,-1.52) |
| Italy | male | 429521.35(473850.98,387689.18) | 377715.62(416726.03,337701.04) | -0.12 | 1211.13(1344.17,1087.67) | 668.02(758.64,581.18) | -2.31(-2.43,-2.19) |
| Jamaica | male | 9229.01(11177.88,7655.30) | 13849.78(16739.42,11224.08) | 0.50 | 905.61(1056.03,777.72) | 1007.11(1228.93,814.61) | 0.07(-0.39,0.53) |
| Japan | male | 691829.68(796866.58,599460.20) | 807774.89(891488.99,716428.42) | 0.17 | 1055.24(1220.68,912.64) | 578.14(666.28,499.41) | -2.55(-2.69,-2.40) |
| Jordan | male | 10668.82(12906.62,8645.64) | 30078.94(36984.04,24202.88) | 1.82 | 1162.78(1367.50,967.04) | 726.87(871.72,597.13) | -1.98(-2.11,-1.85) |
| Kazakhstan | male | 112041.96(120970.68,103769.69) | 147746.51(176187.50,124488.75) | 0.32 | 2320.51(2499.94,2156.42) | 2265.09(2701.85,1936.49) | -0.70(-1.22,-0.17) |
| Kenya | male | 75125.86(106273.55,57124.49) | 173917.60(214827.42,138509.07) | 1.32 | 1359.68(2075.08,999.07) | 1374.08(1717.21,1078.24) | 0.25(-0.02,0.51) |
| Kiribati | male | 1115.44(1342.23,920.10) | 1285.16(1606.71,1018.83) | 0.15 | 5627.29(6668.33,4655.37) | 3922.70(4820.12,3080.77) | -1.30(-1.35,-1.25) |
| Kuwait | male | 3772.97(4796.20,2987.56) | 8993.27(11301.39,7084.72) | 1.38 | 615.79(731.45,515.52) | 507.92(622.24,407.79) | -0.63(-0.76,-0.49) |
| Kyrgyzstan | male | 38206.43(41679.95,34752.35) | 25514.75(29547.08,21943.54) | -0.33 | 3114.01(3382.03,2860.21) | 1300.57(1510.91,1125.43) | -3.88(-4.48,-3.27) |
| Lao People's Democratic Republic | male | 51765.25(64572.14,38839.15) | 50535.58(62903.90,39342.58) | -0.02 | 4237.49(5182.93,3224.06) | 2360.20(2905.11,1851.33) | -2.21(-2.28,-2.14) |
| Latvia | male | 15024.77(16678.18,13498.87) | 6577.11(8062.68,5426.99) | -0.56 | 1162.58(1290.96,1047.03) | 532.66(658.22,438.68) | -2.71(-3.05,-2.38) |
| Lebanon | male | 13767.00(16855.94,11103.66) | 23702.36(28172.12,18929.97) | 0.72 | 1169.79(1447.87,950.65) | 993.78(1181.67,797.13) | -0.30(-0.44,-0.16) |
| Lesotho | male | 16084.97(22473.13,11264.96) | 19533.01(25633.62,14542.22) | 0.21 | 3618.37(5026.89,2519.28) | 3499.66(4540.72,2613.62) | 0.12(-0.10,0.33) |
| Liberia | male | 8481.94(10235.29,6830.95) | 11735.17(14464.80,9207.85) | 0.38 | 1123.42(1318.98,936.59) | 814.57(1014.77,641.24) | -1.14(-1.26,-1.01) |
| Libya | male | 13534.35(16706.61,10868.48) | 27623.98(33654.06,21937.58) | 1.04 | 1072.72(1360.19,864.07) | 999.89(1209.24,795.24) | -0.22(-0.32,-0.11) |
| Lithuania | male | 25375.11(27233.02,23496.97) | 11954.38(14157.83,10072.78) | -0.53 | 1471.61(1575.17,1365.48) | 630.01(740.27,530.24) | -3.05(-3.24,-2.87) |
| Luxembourg | male | 3429.04(4002.83,2982.79) | 3624.50(4287.64,3046.09) | 0.06 | 1640.97(1922.51,1420.93) | 897.16(1079.31,734.61) | -2.40(-2.50,-2.31) |
| North Macedonia | male | 11293.95(12787.34,9909.00) | 11354.80(13532.55,9451.29) | 0.01 | 1313.16(1478.53,1150.08) | 857.43(1013.78,720.75) | -1.73(-1.84,-1.62) |
| Madagascar | male | 121615.02(141444.89,104241.84) | 157130.84(190309.35,126689.77) | 0.29 | 2808.08(3183.39,2481.37) | 2110.35(2578.59,1701.75) | -1.15(-1.26,-1.04) |
| Malawi | male | 41171.54(49930.63,34248.49) | 60094.78(71087.10,49881.38) | 0.46 | 1583.57(1876.27,1341.29) | 1385.74(1602.49,1167.45) | -0.59(-0.78,-0.39) |
| Malaysia | male | 95826.07(110432.14,82158.58) | 151513.35(181193.66,126529.50) | 0.58 | 2031.11(2386.29,1698.22) | 1149.89(1390.93,956.70) | -2.82(-3.19,-2.44) |
| Maldives | male | 1695.18(2079.34,1274.41) | 1983.98(2286.46,1706.26) | 0.17 | 2813.34(3321.49,2177.46) | 1137.02(1312.66,978.87) | -3.57(-3.79,-3.34) |
| Mali | male | 54731.41(63933.29,45415.82) | 101230.30(126248.47,78642.65) | 0.85 | 2275.48(2682.77,1789.96) | 1806.65(2278.33,1385.06) | -0.72(-0.89,-0.55) |
| Malta | male | 2385.47(2716.06,2092.41) | 2959.01(3371.15,2558.72) | 0.24 | 1315.89(1488.27,1158.92) | 820.62(965.82,692.00) | -1.82(-1.90,-1.74) |
| Marshall Islands | male | 440.71(623.98,356.66) | 507.14(662.51,384.92) | 0.15 | 4382.58(6509.88,3508.21) | 2630.32(3439.99,2008.86) | -1.64(-1.77,-1.50) |
| Mauritania | male | 10598.56(12697.07,8541.58) | 12266.79(14970.47,9641.09) | 0.16 | 1787.03(2152.02,1452.29) | 928.20(1117.06,750.51) | -2.14(-2.38,-1.91) |
| Mauritius | male | 8551.85(9235.14,7893.65) | 8798.81(10419.04,7412.97) | 0.03 | 2498.52(2683.52,2322.68) | 1241.26(1467.33,1057.77) | -2.56(-2.70,-2.43) |
| Mexico | male | 282387.64(321141.83,253369.22) | 483369.49(568571.50,411571.39) | 0.71 | 1158.90(1251.11,1076.75) | 907.93(1065.95,774.36) | -1.03(-1.14,-0.93) |
| Republic of Moldova | male | 33291.99(36035.12,30790.41) | 16473.86(19261.04,14202.86) | -0.51 | 1826.39(1966.10,1699.34) | 749.28(876.09,649.04) | -3.57(-3.92,-3.21) |
| Mongolia | male | 9482.39(11208.72,7431.42) | 9302.20(12200.39,7546.77) | -0.02 | 1659.10(1956.72,1350.40) | 875.17(1117.22,721.46) | -3.02(-3.36,-2.68) |
| Montenegro | male | 1235.89(1466.77,1028.77) | 1715.55(2018.61,1430.46) | 0.39 | 461.53(538.94,385.68) | 463.31(557.65,383.45) | 0.07(0.00,0.15) |
| Morocco | male | 89121.77(117574.22,71846.17) | 164898.34(198961.13,132271.59) | 0.85 | 1175.44(1619.75,939.55) | 1102.84(1334.65,887.52) | -0.39(-0.57,-0.20) |
| Mozambique | male | 59152.15(72072.36,48552.84) | 113200.80(136945.66,92506.82) | 0.91 | 1549.48(1928.43,1299.93) | 1654.40(1984.29,1377.08) | 0.41(0.24,0.58) |
| Myanmar | male | 640791.77(784728.38,520506.62) | 646150.81(750636.50,554614.11) | 0.01 | 5494.86(6729.24,4468.34) | 3421.51(3900.95,2959.50) | -1.75(-1.83,-1.67) |
| Namibia | male | 11695.96(14387.92,8781.40) | 14184.73(17041.65,11590.13) | 0.21 | 3304.92(4075.42,2495.65) | 2290.78(2716.92,1894.32) | -1.42(-1.64,-1.19) |
| Nepal | male | 246100.91(296093.09,189274.80) | 429970.57(508547.85,319769.70) | 0.75 | 5476.11(6553.13,4045.47) | 4357.25(5104.27,3224.43) | -0.67(-0.87,-0.46) |
| Netherlands | male | 124847.16(140685.01,111768.62) | 146076.67(164424.34,127382.37) | 0.17 | 1554.17(1753.95,1385.71) | 1037.80(1198.79,893.19) | -1.73(-1.88,-1.57) |
| New Zealand | male | 30415.36(35407.12,26234.82) | 30579.69(34322.70,26948.99) | 0.01 | 1792.45(2093.56,1550.16) | 958.37(1101.62,827.83) | -2.59(-2.77,-2.41) |
| Nicaragua | male | 13073.90(16411.99,10447.33) | 19288.79(23238.06,15647.52) | 0.48 | 1025.42(1186.22,894.40) | 952.71(1121.80,777.04) | -0.19(-0.36,-0.01) |
| Niger | male | 48122.98(63432.36,36659.64) | 85513.46(115042.06,62326.93) | 0.78 | 2214.92(3066.11,1658.18) | 1483.74(2059.90,1069.06) | -1.16(-1.40,-0.92) |
| Nigeria | male | 394426.52(493292.58,304007.81) | 591662.10(736352.25,469939.01) | 0.50 | 1439.80(1794.36,1085.45) | 1045.57(1330.60,823.81) | -1.06(-1.11,-1.00) |
| Democratic People's Republic of Korea | male | 224702.52(286702.10,165669.03) | 351506.33(416373.78,269097.02) | 0.56 | 4227.11(5258.55,3240.50) | 3038.35(3533.07,2349.47) | -1.16(-1.29,-1.03) |
| Northern Mariana Islands | male | 275.55(329.26,225.82) | 328.42(393.93,281.07) | 0.19 | 2176.47(2499.37,1895.48) | 1466.38(1720.00,1275.39) | -1.30(-1.36,-1.24) |
| Norway | male | 32885.70(38633.67,27997.60) | 37645.07(42338.12,32989.98) | 0.14 | 1215.02(1454.06,1019.11) | 957.08(1121.13,819.29) | -0.93(-1.07,-0.79) |
| Oman | male | 4886.80(6534.21,3833.57) | 9961.39(12633.37,7875.25) | 1.04 | 1070.07(1499.41,820.48) | 762.09(895.47,623.54) | -0.88(-1.03,-0.72) |
| Pakistan | male | 1180832.84(1434043.67,952043.55) | 1588641.68(1981868.46,1260992.18) | 0.35 | 3656.95(4525.39,2935.71) | 2855.24(3543.34,2272.63) | -1.00(-1.24,-0.76) |
| Palestine | male | 6981.97(8968.33,5685.93) | 12600.07(14983.57,10552.11) | 0.80 | 1417.90(1926.99,1145.62) | 941.04(1079.93,808.07) | -1.59(-1.69,-1.49) |
| Panama | male | 8052.30(9631.86,6787.90) | 12473.56(15187.30,9990.73) | 0.55 | 825.96(950.08,722.11) | 617.55(750.63,495.83) | -1.41(-1.60,-1.21) |
| Papua New Guinea | male | 55877.30(68334.78,44629.23) | 114087.98(142754.82,90017.09) | 1.04 | 5357.56(6518.02,4267.76) | 4378.71(5439.51,3423.17) | -0.63(-0.69,-0.58) |
| Paraguay | male | 9718.61(12173.47,8005.85) | 22797.56(28184.78,18047.75) | 1.35 | 668.03(851.28,573.21) | 817.54(1012.57,644.09) | 0.95(0.83,1.08) |
| Peru | male | 93334.10(114100.75,75784.08) | 103668.97(129638.61,80724.94) | 0.11 | 990.44(1190.59,829.80) | 651.97(814.78,503.75) | -1.03(-1.22,-0.84) |
| Philippines | male | 480856.51(541147.84,422929.87) | 834471.72(1006376.76,692363.29) | 0.74 | 2594.25(2892.67,2295.93) | 2159.62(2583.39,1793.85) | -0.47(-0.58,-0.35) |
| Poland | male | 322286.68(359152.79,291233.03) | 208442.47(244542.26,174736.42) | -0.35 | 1809.37(2006.80,1637.41) | 807.30(953.60,676.08) | -2.93(-3.10,-2.76) |
| Portugal | male | 88253.65(100198.61,78006.99) | 88671.29(101646.85,77225.10) | 0.00 | 1604.01(1835.82,1410.33) | 1017.39(1212.57,856.69) | -1.68(-1.74,-1.63) |
| Puerto Rico | male | 18857.52(22750.84,15949.96) | 20819.81(25538.17,17091.98) | 0.10 | 1113.29(1334.67,944.59) | 881.36(1099.19,700.07) | -1.23(-1.44,-1.03) |
| Qatar | male | 1009.49(1237.90,808.84) | 5635.82(7155.84,4396.16) | 4.58 | 877.95(1177.36,710.51) | 580.90(727.61,478.45) | -1.56(-1.78,-1.34) |
| Romania | male | 229445.25(245774.75,213884.98) | 140795.00(165255.45,119663.98) | -0.39 | 1926.23(2062.16,1797.48) | 991.97(1161.47,839.25) | -2.41(-2.58,-2.24) |
| Russian Federation | male | 1047474.78(1129229.88,980994.16) | 674415.60(788992.83,577837.97) | -0.36 | 1695.73(1820.02,1587.75) | 815.14(948.91,697.63) | -3.32(-3.66,-2.98) |
| Rwanda | male | 68289.76(86456.38,50506.04) | 61261.33(76139.48,48427.99) | -0.10 | 3468.25(4313.38,2592.99) | 1832.47(2249.61,1447.36) | -2.93(-3.20,-2.66) |
| Saint Lucia | male | 738.37(872.10,627.56) | 1210.06(1426.41,1011.73) | 0.64 | 1537.05(1756.68,1350.44) | 1314.77(1555.65,1097.76) | -0.90(-1.05,-0.75) |
| Saint Vincent and the Grenadines | male | 328.40(410.76,263.85) | 468.71(547.08,396.07) | 0.43 | 749.48(888.27,633.90) | 765.85(905.34,640.38) | -0.12(-0.23,-0.02) |
| Samoa | male | 1737.67(2180.38,1366.13) | 1456.30(1810.36,1203.46) | -0.16 | 3730.55(4635.54,2965.70) | 1973.18(2486.50,1642.55) | -2.30(-2.43,-2.17) |
| Sao Tome and Principe | male | 1050.30(1245.44,852.08) | 1509.25(1913.65,1174.82) | 0.44 | 2968.34(3473.30,2423.19) | 2648.26(3401.05,2057.20) | -0.37(-0.46,-0.28) |
| Saudi Arabia | male | 46011.05(65300.29,36153.39) | 103940.72(125185.46,85684.70) | 1.26 | 1175.90(1774.72,908.10) | 867.56(1014.23,729.18) | -1.10(-1.16,-1.04) |
| Senegal | male | 41096.67(49828.66,31577.51) | 56960.76(70278.23,45618.53) | 0.39 | 2023.95(2464.97,1553.26) | 1305.97(1613.73,1053.02) | -1.21(-1.50,-0.93) |
| Serbia | male | 59464.79(68123.68,51654.45) | 59843.07(71450.44,50219.67) | 0.01 | 1196.70(1369.29,1048.73) | 901.34(1065.83,759.61) | -1.13(-1.29,-0.96) |
| Seychelles | male | 446.69(497.66,387.13) | 611.37(693.64,532.87) | 0.37 | 1761.46(1948.31,1524.99) | 1237.32(1403.63,1083.70) | -1.58(-1.75,-1.41) |
| Sierra Leone | male | 24133.65(29853.89,18739.22) | 33146.15(42854.54,24833.22) | 0.37 | 2019.43(2439.25,1570.65) | 1364.95(1752.63,1028.75) | -1.06(-1.25,-0.88) |
| Singapore | male | 20901.13(23262.71,18835.44) | 19526.16(22775.31,16703.81) | -0.07 | 2166.66(2369.42,1988.86) | 594.31(700.97,503.29) | -4.77(-5.01,-4.52) |
| Slovakia | male | 20584.78(26368.97,18326.95) | 22154.19(26553.08,18350.41) | 0.08 | 824.93(1058.92,735.28) | 636.93(760.97,526.80) | -0.60(-0.75,-0.45) |
| Slovenia | male | 12949.99(15483.80,10682.21) | 10257.42(12337.03,8527.52) | -0.21 | 1403.54(1670.53,1160.61) | 644.15(772.78,529.75) | -3.31(-3.56,-3.05) |
| Solomon Islands | male | 4032.03(5140.10,3142.55) | 6957.52(8380.72,5635.54) | 0.73 | 4670.08(6039.18,3654.73) | 3986.25(4731.25,3225.49) | -0.48(-0.54,-0.42) |
| Somalia | male | 54806.82(77488.90,39086.62) | 98606.42(145800.53,71635.71) | 0.80 | 3037.75(4523.37,2156.55) | 2268.69(3490.54,1584.41) | -1.00(-1.05,-0.95) |
| South Africa | male | 243919.58(272583.83,217503.33) | 334949.99(372198.09,301879.69) | 0.37 | 2264.33(2581.65,2037.47) | 1723.10(1888.98,1569.76) | -1.30(-1.86,-0.73) |
| Republic of Korea | male | 182922.43(204198.43,163692.46) | 262115.00(292774.97,229088.97) | 0.43 | 1725.56(1867.53,1547.99) | 795.40(903.33,687.84) | -3.08(-3.26,-2.89) |
| South Sudan | male | 37958.82(48961.18,27511.49) | 37034.22(47516.96,28382.52) | -0.02 | 2033.63(2542.76,1482.94) | 1404.55(1810.57,1046.71) | -1.34(-1.47,-1.22) |
| Spain | male | 350111.16(379073.37,323183.11) | 405880.10(454541.23,359020.29) | 0.16 | 1575.67(1716.34,1452.40) | 1020.62(1159.33,894.71) | -1.75(-1.85,-1.65) |
| Sri Lanka | male | 153817.63(168749.00,136036.85) | 187931.99(237751.58,145999.58) | 0.22 | 2797.34(3055.50,2491.69) | 1803.31(2256.75,1411.53) | -1.19(-1.39,-0.99) |
| Sudan | male | 137644.22(191273.38,84577.79) | 167728.91(218796.11,117892.22) | 0.22 | 2119.82(2891.95,1291.19) | 1374.44(1820.71,943.96) | -1.58(-1.68,-1.48) |
| Suriname | male | 1405.92(1691.32,1171.76) | 2309.98(2773.27,1899.25) | 0.64 | 923.09(1079.61,794.26) | 852.64(1024.39,701.64) | -0.48(-0.69,-0.28) |
| Eswatini | male | 5585.50(6861.26,4291.18) | 7671.78(9533.76,6221.99) | 0.37 | 3599.16(4392.94,2754.29) | 2829.09(3434.65,2332.60) | -0.66(-0.99,-0.34) |
| Sweden | male | 55918.11(67603.72,46865.97) | 59477.73(69112.93,51480.44) | 0.06 | 972.95(1205.05,796.34) | 728.84(889.17,602.95) | -1.23(-1.42,-1.04) |
| Switzerland | male | 51377.61(58499.88,44836.07) | 47216.45(54378.79,40656.11) | -0.08 | 1260.01(1452.45,1086.07) | 713.85(854.92,596.96) | -2.19(-2.29,-2.09) |
| Syrian Arab Republic | male | 48965.01(58190.95,40377.22) | 65371.40(81708.76,52516.63) | 0.34 | 1282.52(1557.49,1045.33) | 1041.36(1304.60,839.83) | -0.81(-0.96,-0.67) |
| Taiwan (Province of China) | male | 98393.05(109507.80,88588.52) | 125697.70(151928.18,104525.76) | 0.28 | 1268.34(1388.01,1147.38) | 770.91(929.73,642.06) | -1.96(-2.08,-1.83) |
| Tajikistan | male | 23953.62(26457.58,21686.35) | 31541.90(38953.55,26431.67) | 0.32 | 1762.85(1931.14,1600.95) | 1259.50(1640.32,1050.81) | -1.27(-1.58,-0.97) |
| United Republic of Tanzania | male | 111883.74(133293.39,92939.48) | 196535.60(241067.29,158381.59) | 0.76 | 1459.26(1684.09,1245.73) | 1166.28(1368.20,964.78) | -0.80(-0.85,-0.75) |
| Thailand | male | 401731.78(456794.02,341095.45) | 461805.25(554172.04,379181.74) | 0.15 | 2310.68(2629.38,1957.56) | 1103.96(1321.33,908.39) | -3.00(-3.19,-2.81) |
| Bahamas | male | 728.04(886.57,596.92) | 1236.34(1505.97,1017.17) | 0.70 | 796.47(929.22,683.06) | 729.09(901.58,598.69) | -0.40(-0.46,-0.34) |
| Gambia | male | 4726.38(6002.95,3562.09) | 9017.95(11273.12,7050.78) | 0.91 | 1950.03(2447.52,1479.27) | 1524.22(1894.72,1193.02) | -0.79(-0.99,-0.59) |
| Timor-Leste | male | 5214.12(6844.61,3721.89) | 9178.81(11755.07,6810.00) | 0.76 | 2544.56(3314.87,1793.02) | 2174.47(2779.94,1633.82) | -0.63(-0.87,-0.40) |
| Togo | male | 17115.02(20275.73,13925.57) | 33119.39(42074.01,25238.96) | 0.94 | 2055.53(2424.46,1658.20) | 1582.85(2022.08,1214.05) | -0.72(-0.81,-0.63) |
| Tonga | male | 693.07(804.68,577.69) | 686.02(817.59,567.79) | -0.01 | 2489.69(2902.81,2059.95) | 1835.08(2187.04,1518.05) | -1.14(-1.35,-0.93) |
| Trinidad and Tobago | male | 4481.02(5141.58,3938.64) | 5356.38(6751.84,4202.49) | 0.20 | 946.97(1052.71,855.47) | 700.25(876.08,552.91) | -1.10(-1.21,-0.98) |
| Tunisia | male | 29591.86(36467.58,24269.85) | 52872.91(65404.40,42165.99) | 0.79 | 1041.34(1315.09,864.46) | 920.90(1133.76,735.40) | -0.53(-0.59,-0.48) |
| Turkey | male | 367048.84(424207.60,309084.36) | 573192.29(674298.70,463233.87) | 0.56 | 1970.97(2277.41,1662.01) | 1448.52(1694.83,1167.46) | -0.69(-0.89,-0.50) |
| Turkmenistan | male | 15311.11(16760.41,13964.17) | 10302.56(12475.91,8347.14) | -0.33 | 1598.52(1712.34,1487.36) | 521.94(627.64,430.36) | -4.77(-5.20,-4.35) |
| Uganda | male | 111316.14(148944.30,82012.79) | 165650.27(211607.78,126010.02) | 0.49 | 2357.01(3384.43,1723.95) | 1725.82(2146.69,1291.82) | -1.45(-1.60,-1.29) |
| Ukraine | male | 531959.61(573157.11,494600.77) | 230627.78(277698.27,189855.94) | -0.57 | 2144.38(2305.30,1992.31) | 882.97(1062.30,729.57) | -4.54(-5.06,-4.02) |
| United Arab Emirates | male | 14723.81(19450.28,11017.92) | 94654.86(121736.85,66186.41) | 5.43 | 2328.15(3076.46,1888.91) | 1649.26(2005.26,1347.75) | -1.42(-1.60,-1.24) |
| United Kingdom | male | 635412.93(713793.40,568570.66) | 636352.37(701355.40,575617.17) | 0.00 | 1833.48(2095.73,1614.28) | 1236.50(1406.29,1094.17) | -1.53(-1.63,-1.44) |
| United States of America | male | 2042578.23(2291394.41,1834622.39) | 3360438.89(3690173.59,3048782.98) | 0.65 | 1563.78(1768.26,1392.54) | 1503.91(1704.68,1328.15) | -0.07(-0.11,-0.04) |
| Uruguay | male | 25153.28(27979.15,22471.44) | 29158.78(32673.58,25900.11) | 0.16 | 1522.81(1699.84,1363.08) | 1372.96(1556.92,1213.49) | -0.55(-0.75,-0.35) |
| Uzbekistan | male | 84928.07(94410.94,76169.05) | 87396.06(105054.60,72565.61) | 0.03 | 1579.90(1727.80,1439.86) | 871.07(1024.22,736.13) | -3.18(-3.73,-2.62) |
| Vanuatu | male | 1813.32(2377.32,1248.73) | 3182.48(4169.28,2364.92) | 0.76 | 4360.62(5665.27,2986.51) | 3237.19(4185.85,2381.07) | -1.28(-1.39,-1.16) |
| Venezuela (Bolivarian Republic of) | male | 52742.79(64632.79,43750.19) | 105511.89(132012.62,83438.69) | 1.00 | 782.81(896.02,686.21) | 806.19(1007.53,638.26) | -0.35(-0.61,-0.10) |
| Viet nam | male | 395296.42(490462.32,195938.69) | 634040.14(780023.19,332198.12) | 0.60 | 2279.52(2834.55,1035.60) | 1743.43(2118.70,874.98) | -0.94(-0.99,-0.88) |
| Virginia | male | 47146.02(53213.24,41930.84) | 82185.70(96462.47,69194.10) | 0.74 | 1617.47(1833.80,1438.36) | 1447.68(1710.11,1221.54) | -0.43(-0.48,-0.38) |
| Yemen | male | 70703.00(94514.69,46524.72) | 122864.26(153802.98,97899.66) | 0.74 | 2279.09(3115.65,1425.74) | 1520.24(1916.90,1210.85) | -1.48(-1.57,-1.39) |
| Zambia | male | 34280.82(41366.03,28428.02) | 58965.62(69761.64,49009.32) | 0.72 | 1663.65(2036.03,1387.05) | 1405.17(1644.23,1166.00) | -0.77(-1.01,-0.53) |
| Zimbabwe | male | 37318.68(42829.53,32103.72) | 57757.72(68896.02,47178.85) | 0.55 | 1678.06(1941.83,1450.21) | 1664.84(1973.28,1376.42) | -0.06(-0.26,0.14) |
| Monaco | male | 249.35(290.43,210.40) | 281.20(324.68,239.69) | 0.13 | 973.06(1150.99,815.97) | 785.31(936.85,652.60) | -0.71(-0.72,-0.69) |
| San Marino | male | 107.06(126.43,89.51) | 163.84(199.06,131.34) | 0.53 | 780.20(925.34,647.68) | 656.63(805.98,523.48) | -0.58(-0.65,-0.50) |
| Saint Kitts and Nevis | male | 157.67(191.01,131.69) | 234.23(279.85,190.03) | 0.49 | 877.10(1040.12,746.20) | 821.71(994.89,676.81) | -0.39(-0.50,-0.28) |
| Cook Islands | male | 128.97(145.86,112.51) | 115.74(133.18,100.14) | -0.10 | 1860.11(2077.73,1619.32) | 1062.78(1230.20,915.22) | -2.03(-2.15,-1.90) |
| Nauru | male | 113.76(141.61,88.44) | 75.96(99.35,58.44) | -0.33 | 4331.77(5319.73,3434.28) | 2914.59(3750.87,2290.66) | -1.47(-1.74,-1.19) |
| Niue | male | 27.85(34.29,22.99) | 17.09(21.39,14.12) | -0.39 | 2918.77(3603.79,2410.62) | 1869.87(2339.54,1555.40) | -1.75(-1.84,-1.66) |
| Palau | male | 241.55(295.95,197.80) | 322.52(393.69,263.15) | 0.34 | 4463.69(5383.83,3706.75) | 3173.04(3834.49,2627.15) | -1.10(-1.18,-1.02) |
| Tokelau | male | 17.86(22.14,14.40) | 9.94(11.95,8.20) | -0.44 | 2772.80(3459.16,2242.51) | 1529.79(1836.45,1267.25) | -2.11(-2.16,-2.07) |
| Tuvalu | male | 147.19(179.37,114.33) | 114.20(145.08,92.03) | -0.22 | 4769.87(5829.37,3702.38) | 2337.28(2949.59,1899.41) | -2.52(-2.67,-2.37) |
| Afghanistan | female | 95216.25(116356.68,74781.47) | 169727.29(207137.22,136142.27) | 0.78 | 2417.87(2963.98,1898.30) | 1899.14(2331.82,1469.29) | -1.04(-1.29,-0.78) |
| Albania | female | 8706.21(9857.00,6375.68) | 7411.04(9325.96,5845.02) | -0.15 | 745.64(841.46,528.59) | 395.37(498.12,311.90) | -2.45(-2.72,-2.18) |
| Algeria | female | 77627.47(98006.81,55115.41) | 124075.79(149840.19,103060.52) | 0.60 | 1058.72(1359.07,723.77) | 737.20(894.88,608.90) | -1.24(-1.32,-1.16) |
| American Samoa | female | 212.56(262.01,175.25) | 272.99(341.75,222.30) | 0.28 | 1577.66(1936.41,1283.53) | 1119.97(1407.84,914.10) | -1.29(-1.41,-1.16) |
| Andorra | female | 179.17(226.07,140.46) | 403.72(487.81,326.42) | 1.25 | 694.54(871.24,547.08) | 665.92(831.68,531.29) | -0.20(-0.31,-0.08) |
| Angola | female | 70554.22(104164.79,42946.47) | 102944.38(126382.16,76689.26) | 0.46 | 2243.49(3340.70,1507.45) | 1251.68(1627.08,902.10) | -2.26(-2.45,-2.07) |
| Antigua and Barbuda | female | 128.40(166.26,99.46) | 213.23(264.74,168.40) | 0.66 | 403.20(520.49,314.53) | 467.51(597.76,361.43) | 0.44(0.37,0.51) |
| Argentina | female | 144795.34(176770.39,119825.65) | 251732.52(296395.22,210307.85) | 0.74 | 836.35(1017.98,690.49) | 896.67(1071.31,743.74) | 0.17(0.08,0.27) |
| Armenia | female | 10346.29(11649.96,7654.55) | 12150.83(14296.76,9296.49) | 0.17 | 696.79(782.64,501.03) | 549.81(646.02,432.03) | -0.78(-0.95,-0.61) |
| Australia | female | 108932.12(128428.20,91685.80) | 164724.04(192817.37,137488.07) | 0.51 | 1125.01(1353.80,931.61) | 914.40(1120.83,739.70) | -1.10(-1.27,-0.92) |
| Austria | female | 35572.06(43047.05,29530.90) | 43231.39(50684.80,35485.42) | 0.22 | 596.26(737.48,476.38) | 558.56(690.72,451.00) | -0.17(-0.23,-0.12) |
| Azerbaijan | female | 25689.61(29261.30,22539.64) | 28489.51(40343.39,22579.54) | 0.11 | 827.26(945.35,729.27) | 620.51(916.03,472.35) | -1.61(-1.91,-1.32) |
| Bahrain | female | 1544.73(1796.97,1311.27) | 3216.16(4011.44,2672.47) | 1.08 | 1575.17(1818.65,1331.01) | 876.92(1079.19,739.23) | -2.36(-2.72,-2.01) |
| Bangladesh | female | 584797.69(814199.90,473391.61) | 817400.72(1285157.67,647141.55) | 0.40 | 2446.56(3605.19,1932.73) | 1295.47(2086.35,1021.17) | -2.32(-2.47,-2.18) |
| Barbados | female | 697.61(862.90,554.13) | 946.77(1162.05,755.62) | 0.36 | 488.53(613.01,382.65) | 516.39(668.28,394.22) | 0.26(0.20,0.32) |
| Belarus | female | 70803.40(81313.96,46081.09) | 27737.34(46698.88,21156.11) | -0.61 | 920.99(1069.13,616.55) | 354.75(557.48,267.33) | -3.94(-4.27,-3.62) |
| Belgium | female | 63121.20(73599.11,54123.37) | 79735.66(90779.38,67269.66) | 0.26 | 816.92(977.63,681.47) | 747.61(876.76,619.82) | -0.37(-0.46,-0.28) |
| Belize | female | 542.78(680.35,433.37) | 1193.76(1478.28,957.81) | 1.20 | 684.24(819.73,567.05) | 674.10(807.82,559.83) | -0.16(-0.33,0.00) |
| Benin | female | 22234.10(26962.60,17843.74) | 41438.08(52613.52,32902.34) | 0.86 | 1539.60(1878.00,1240.21) | 1114.77(1407.58,905.39) | -1.01(-1.14,-0.87) |
| Bermuda | female | 158.95(193.04,128.58) | 179.99(220.77,146.62) | 0.13 | 505.92(622.57,405.55) | 434.72(568.43,328.70) | -0.75(-0.85,-0.65) |
| Bhutan | female | 4744.80(6093.10,3422.89) | 6816.57(8373.47,5363.46) | 0.44 | 3614.99(4674.50,2639.64) | 2603.30(3219.12,2044.36) | -1.27(-1.32,-1.21) |
| Bolivia (Plurinational State of) | female | 42763.94(60115.38,27813.16) | 49096.01(59676.60,38460.94) | 0.15 | 1524.83(1925.83,1133.21) | 1018.15(1241.63,794.66) | -1.43(-1.55,-1.30) |
| Bosnia and Herzegovina | female | 15957.06(18350.06,13395.38) | 13640.08(16352.11,11061.93) | -0.15 | 740.90(847.77,612.17) | 500.92(610.64,405.99) | -1.61(-1.72,-1.50) |
| Botswana | female | 6248.95(9756.76,4310.00) | 10675.52(13702.00,7993.32) | 0.71 | 1667.03(2639.05,1140.15) | 1234.93(1597.03,920.58) | -1.05(-1.16,-0.94) |
| Brazil | female | 647005.86(769442.77,547750.73) | 977476.22(1143572.26,852555.59) | 0.51 | 1147.48(1309.35,994.94) | 812.57(963.00,700.57) | -1.62(-1.78,-1.46) |
| Brunei Darussalam | female | 948.91(1107.68,702.45) | 1403.93(1705.19,1201.46) | 0.48 | 1806.71(2107.32,1212.58) | 1037.11(1199.94,896.06) | -2.37(-2.61,-2.12) |
| Bulgaria | female | 39579.28(45352.89,33090.32) | 34398.85(42308.27,28208.25) | -0.13 | 679.72(791.22,570.73) | 537.56(658.81,439.01) | -1.08(-1.23,-0.93) |
| Burkina Faso | female | 38344.73(47908.52,29723.88) | 74181.48(93898.50,58175.52) | 0.93 | 1125.99(1349.47,902.52) | 935.58(1135.81,766.28) | -0.70(-0.83,-0.58) |
| Burundi | female | 46655.57(62373.15,28855.87) | 51079.57(73788.79,38229.46) | 0.09 | 2250.92(2857.88,1435.38) | 1449.57(2045.24,1091.87) | -1.72(-1.79,-1.64) |
| Cambodia | female | 40674.47(50445.70,29188.02) | 61227.78(71903.01,50845.07) | 0.51 | 1247.45(1496.08,932.11) | 896.30(1045.75,731.78) | -1.29(-1.36,-1.22) |
| Cameroon | female | 45924.97(54604.49,36935.69) | 88556.54(111223.66,68549.58) | 0.93 | 1577.37(1877.26,1258.76) | 1054.47(1325.46,820.34) | -1.28(-1.34,-1.23) |
| Canada | female | 113229.00(131479.84,97706.64) | 218675.91(248164.67,179733.34) | 0.93 | 674.97(794.89,570.97) | 685.53(797.36,569.90) | -0.03(-0.14,0.08) |
| Cabo Verde | female | 1810.19(2089.40,1289.57) | 1698.99(2080.43,1408.77) | -0.06 | 1252.43(1444.87,875.09) | 663.22(809.46,549.27) | -2.09(-2.55,-1.62) |
| Central African Republic | female | 20832.15(27703.51,13668.58) | 31313.54(45151.23,20750.13) | 0.50 | 2511.98(3817.70,1588.11) | 2107.92(3470.64,1331.06) | -0.57(-0.65,-0.49) |
| Chad | female | 30107.21(38315.55,23014.20) | 51920.69(65094.75,40847.36) | 0.72 | 1605.30(2093.45,1216.56) | 1267.49(1600.28,993.47) | -0.67(-0.75,-0.59) |
| Chile | female | 40503.93(47878.85,33929.62) | 80679.07(95688.08,62423.86) | 0.99 | 711.43(828.41,591.64) | 666.54(803.25,524.77) | -0.19(-0.26,-0.12) |
| China | female | 13563126.33(15597900.49,9354265.20) | 9762872.50(12068535.22,8202723.18) | -0.28 | 3453.79(3955.61,2395.19) | 1030.25(1262.34,866.81) | -4.60(-4.77,-4.42) |
| Colombia | female | 102889.88(121821.14,85512.14) | 188198.45(228402.37,147894.65) | 0.83 | 932.43(1057.12,767.91) | 676.41(817.20,535.68) | -1.40(-1.51,-1.30) |
| Comoros | female | 2283.38(3063.05,1385.18) | 2949.09(3623.61,2378.46) | 0.29 | 1391.82(1843.83,888.02) | 992.21(1214.57,803.64) | -1.32(-1.45,-1.19) |
| Congo | female | 14792.47(20323.28,9841.09) | 21432.39(27186.52,15584.52) | 0.45 | 2070.30(3145.97,1319.34) | 1374.00(1791.42,982.31) | -1.47(-1.59,-1.35) |
| Costa Rica | female | 10302.64(12917.01,8207.99) | 17797.52(21710.10,14418.89) | 0.73 | 880.79(1056.86,735.86) | 679.53(838.55,546.78) | -1.34(-1.63,-1.05) |
| Côte d'Ivoire | female | 40351.88(49891.15,32138.72) | 75663.22(93253.84,60563.10) | 0.88 | 1355.28(1668.52,1073.80) | 997.63(1221.93,812.87) | -0.76(-0.89,-0.63) |
| Croatia | female | 17794.08(21550.17,14716.45) | 19990.90(23616.83,16191.72) | 0.12 | 534.70(653.77,436.97) | 483.77(592.04,389.50) | -0.28(-0.36,-0.20) |
| Cuba | female | 42508.70(51623.63,34640.88) | 64508.42(77948.62,45977.22) | 0.52 | 795.97(966.25,649.07) | 823.70(1018.10,614.18) | 0.04(-0.03,0.11) |
| Cyprus | female | 4374.32(5309.28,3430.71) | 6768.53(8179.92,5369.30) | 0.55 | 1162.77(1412.61,898.56) | 787.19(974.77,613.58) | -1.42(-1.57,-1.26) |
| Czechia | female | 32854.12(40282.77,28325.63) | 46275.41(54221.31,37160.74) | 0.41 | 471.19(575.47,401.01) | 487.73(578.52,389.77) | 0.48(0.28,0.68) |
| Democratic Republic of the Congo | female | 242043.83(320588.53,154804.92) | 410331.15(640991.10,283836.29) | 0.70 | 2103.23(3316.98,1288.62) | 1765.10(3013.65,1144.32) | -0.54(-0.71,-0.36) |
| Denmark | female | 42186.09(47451.45,35602.30) | 55972.99(64340.29,41063.13) | 0.33 | 1057.43(1215.53,868.27) | 1009.06(1176.87,769.43) | -0.55(-0.84,-0.27) |
| Djibouti | female | 1654.55(2426.85,1089.15) | 3274.09(4367.21,2196.20) | 0.98 | 1185.22(1634.10,758.31) | 839.21(1133.29,500.72) | -1.34(-1.40,-1.28) |
| Dominica | female | 239.60(298.23,197.51) | 226.68(276.68,175.43) | -0.05 | 610.40(756.46,499.81) | 598.49(743.49,466.27) | -0.07(-0.17,0.03) |
| Dominican Republic | female | 24369.22(30068.44,18854.10) | 31612.84(40508.43,23623.00) | 0.30 | 766.96(902.76,636.53) | 619.93(794.71,463.89) | -0.33(-0.63,-0.04) |
| Ecuador | female | 39323.03(48806.46,27494.06) | 45077.42(55507.51,36858.96) | 0.15 | 888.41(1042.27,678.05) | 570.54(696.98,466.84) | -1.17(-1.63,-0.72) |
| Egypt | female | 277623.49(330543.31,223274.69) | 364909.89(477107.23,285956.40) | 0.31 | 1450.88(1650.99,1257.28) | 1154.68(1541.31,898.09) | -0.58(-0.68,-0.48) |
| El Salvador | female | 26338.51(32553.56,19777.70) | 22295.71(27176.34,17786.72) | -0.15 | 1112.13(1305.11,891.91) | 644.55(789.56,514.10) | -1.92(-2.21,-1.63) |
| Equatorial Guinea | female | 3019.57(4104.04,1966.39) | 3629.33(5924.91,2625.04) | 0.20 | 2113.69(3313.68,1346.12) | 1055.94(1932.97,727.94) | -2.70(-2.89,-2.51) |
| Eritrea | female | 18075.17(25444.97,11565.75) | 26931.14(34749.27,19895.50) | 0.49 | 1792.31(2467.82,1200.55) | 1290.08(1672.04,890.12) | -1.07(-1.16,-0.99) |
| Estonia | female | 4172.62(4771.53,3613.04) | 3420.88(4184.11,2859.42) | -0.18 | 377.14(435.85,321.38) | 279.98(339.61,228.49) | -1.26(-1.45,-1.07) |
| Ethiopia | female | 283635.65(359906.24,186206.18) | 268030.83(329319.37,219486.68) | -0.06 | 1762.33(2185.96,1227.23) | 866.95(1035.22,724.33) | -2.80(-2.95,-2.65) |
| Micronesia (Federated States of) | female | 953.27(1182.88,718.43) | 821.45(1132.31,532.08) | -0.14 | 3390.55(4342.38,2527.30) | 2187.86(2981.41,1429.91) | -1.62(-1.78,-1.47) |
| Fiji | female | 5144.79(7021.43,3920.40) | 4735.26(6149.85,3613.07) | -0.08 | 2308.08(3177.98,1744.50) | 1214.13(1570.14,932.77) | -2.58(-2.76,-2.39) |
| Finland | female | 17812.64(22055.55,14462.03) | 25451.15(30450.11,20362.05) | 0.43 | 523.55(670.05,409.87) | 538.46(680.15,414.76) | 0.27(0.19,0.34) |
| France | female | 291039.29(360293.45,234070.69) | 280809.98(347592.71,225058.66) | -0.04 | 712.92(921.71,555.79) | 516.54(679.47,392.87) | -1.22(-1.34,-1.09) |
| Gabon | female | 4861.06(6045.71,3239.93) | 5308.48(6521.62,3845.83) | 0.09 | 1390.96(1787.81,904.99) | 845.44(1040.74,585.61) | -1.81(-1.90,-1.72) |
| Georgia | female | 15787.35(21513.28,13593.97) | 13403.32(19012.78,10891.00) | -0.15 | 469.15(615.48,405.09) | 432.61(577.49,358.93) | 0.31(0.01,0.61) |
| Germany | female | 506937.16(599594.21,435820.52) | 574158.84(655002.70,492280.44) | 0.13 | 777.30(930.28,651.97) | 674.42(798.46,564.36) | -0.13(-0.36,0.10) |
| Ghana | female | 49554.65(61312.82,36415.46) | 84069.20(99378.54,67465.09) | 0.70 | 1068.37(1294.45,819.88) | 737.58(870.75,591.33) | -1.29(-1.33,-1.26) |
| Greece | female | 46068.45(54808.82,38167.82) | 73870.20(85382.20,59165.95) | 0.60 | 644.32(785.84,523.46) | 668.41(806.05,537.52) | 0.61(0.39,0.82) |
| Greenland | female | 489.51(567.14,406.13) | 522.07(613.35,420.44) | 0.07 | 2783.76(3190.70,2273.75) | 1733.11(2039.72,1377.71) | -2.31(-2.60,-2.01) |
| Grenada | female | 301.37(374.39,240.74) | 357.93(441.14,285.51) | 0.19 | 694.43(858.20,560.64) | 681.66(852.09,540.50) | -0.06(-0.17,0.04) |
| Guam | female | 430.52(506.58,363.15) | 626.66(745.81,525.96) | 0.46 | 1055.39(1219.70,893.88) | 683.23(809.68,571.50) | -1.82(-2.05,-1.58) |
| Guatemala | female | 42624.80(52054.63,27386.27) | 41744.20(53309.75,33277.77) | -0.02 | 1225.69(1419.55,955.22) | 585.26(759.88,467.17) | -2.76(-3.24,-2.28) |
| Guinea | female | 40002.63(49447.17,31471.77) | 54170.71(67657.76,42897.45) | 0.35 | 1879.57(2298.10,1487.20) | 1401.15(1767.84,1114.62) | -0.81(-0.97,-0.64) |
| Guinea-Bissau | female | 5859.70(7277.05,4417.47) | 7784.95(9573.44,6275.70) | 0.33 | 1979.13(2467.63,1484.01) | 1418.22(1762.30,1140.89) | -0.94(-1.09,-0.79) |
| Guyana | female | 2207.45(2775.17,1742.27) | 2318.32(2882.61,1821.91) | 0.05 | 697.15(847.73,572.23) | 631.50(782.85,499.43) | -0.54(-0.65,-0.43) |
| Haiti | female | 56710.51(75708.37,40134.57) | 74054.63(99618.24,53231.34) | 0.31 | 2015.43(2786.47,1422.46) | 1450.15(2147.48,1006.08) | -1.00(-1.07,-0.94) |
| Honduras | female | 33808.37(42541.29,24331.86) | 51657.68(70386.55,34837.76) | 0.53 | 1698.60(1990.61,1383.14) | 1463.43(2016.28,963.40) | -0.49(-0.63,-0.34) |
| Hungary | female | 55641.63(68022.17,49491.51) | 71911.04(83017.50,59568.74) | 0.29 | 740.31(882.09,646.51) | 762.33(896.64,619.50) | 0.30(0.10,0.50) |
| Iceland | female | 1487.84(1778.58,1238.81) | 2039.76(2433.33,1702.21) | 0.37 | 1027.46(1251.25,844.09) | 822.02(1015.72,667.84) | -0.95(-1.11,-0.78) |
| India | female | 7253959.96(8440243.09,5900349.29) | 13518421.71(16187135.70,10166461.88) | 0.86 | 3558.95(4217.03,2850.50) | 2415.47(2892.97,1805.97) | -1.51(-1.61,-1.40) |
| Indonesia | female | 1033819.24(1182545.86,867666.22) | 1187689.25(1393998.47,990804.01) | 0.15 | 1683.61(1995.83,1390.49) | 1069.35(1248.55,895.37) | -1.54(-1.66,-1.42) |
| Iran (Islamic Republic of) | female | 159387.73(190367.22,129921.45) | 252948.54(289415.53,219783.79) | 0.59 | 979.11(1180.87,792.26) | 690.45(784.40,600.60) | -1.32(-1.39,-1.25) |
| Iraq | female | 45164.35(55712.34,35371.69) | 84198.23(103904.50,67218.47) | 0.86 | 769.87(952.03,602.40) | 529.53(644.80,433.83) | -1.54(-1.66,-1.42) |
| Ireland | female | 28070.03(32298.66,23389.49) | 32668.27(37666.39,27467.84) | 0.16 | 1307.49(1521.85,1082.19) | 915.02(1085.74,761.26) | -1.50(-1.61,-1.38) |
| Israel | female | 19653.60(23096.34,16540.48) | 30611.20(37271.27,25264.14) | 0.56 | 773.92(909.19,650.07) | 533.73(661.91,434.11) | -1.43(-1.57,-1.30) |
| Italy | female | 247142.47(290965.97,210869.83) | 291052.76(334221.04,245337.00) | 0.18 | 555.43(667.16,463.83) | 448.34(557.10,363.35) | -0.96(-1.06,-0.85) |
| Jamaica | female | 7462.11(9266.50,5976.88) | 7189.23(9421.91,5588.45) | -0.04 | 638.74(780.90,518.95) | 495.30(646.08,376.96) | -1.24(-1.41,-1.07) |
| Japan | female | 512105.45(629961.00,409675.29) | 483278.12(572400.30,402254.08) | -0.06 | 636.40(810.76,501.12) | 366.78(469.22,284.55) | -2.16(-2.44,-1.88) |
| Jordan | female | 8820.98(10866.86,6912.43) | 21315.47(26729.01,16807.61) | 1.42 | 943.47(1119.13,724.48) | 530.06(643.11,435.39) | -2.51(-2.82,-2.21) |
| Kazakhstan | female | 78085.54(86561.50,60154.18) | 94996.35(109833.74,75465.64) | 0.22 | 1000.70(1108.17,768.88) | 980.52(1133.39,777.74) | -0.49(-0.98,0.00) |
| Kenya | female | 78371.31(105675.45,58201.08) | 156509.46(222346.47,116250.45) | 1.00 | 1239.03(1797.66,882.34) | 1026.37(1516.55,748.19) | -0.41(-0.52,-0.29) |
| Kiribati | female | 914.16(1131.06,739.57) | 1143.53(1473.57,867.49) | 0.25 | 3682.01(4801.30,2895.21) | 2762.42(3555.54,2047.79) | -0.94(-1.02,-0.86) |
| Kuwait | female | 2700.47(3393.47,2178.39) | 6866.27(8849.81,5300.46) | 1.54 | 589.79(697.47,487.47) | 412.55(515.19,328.81) | -1.15(-1.28,-1.02) |
| Kyrgyzstan | female | 29806.21(33639.92,19826.62) | 19396.55(26749.54,16277.87) | -0.35 | 1576.73(1779.00,1034.41) | 738.79(1012.65,621.61) | -3.26(-3.69,-2.83) |
| Lao People's Democratic Republic | female | 39532.28(55372.63,25672.36) | 34043.16(45994.23,26466.24) | -0.14 | 2691.08(3469.99,1950.28) | 1368.24(1851.27,1063.62) | -2.59(-2.68,-2.49) |
| Latvia | female | 9334.30(10820.67,7887.72) | 5366.82(7346.12,4298.38) | -0.43 | 482.01(572.18,399.26) | 301.56(394.11,237.48) | -1.62(-1.86,-1.38) |
| Lebanon | female | 11656.24(14067.32,9382.34) | 20341.83(24923.30,16587.78) | 0.75 | 942.32(1135.89,755.94) | 733.03(902.45,596.61) | -0.97(-1.03,-0.90) |
| Lesotho | female | 13019.46(21517.22,8397.58) | 15780.95(22193.60,10305.14) | 0.21 | 2137.86(3542.80,1361.68) | 2034.09(2876.91,1330.71) | 0.61(0.27,0.94) |
| Liberia | female | 9951.36(13030.13,7755.87) | 14258.95(20123.70,10775.81) | 0.43 | 1311.12(1613.93,1077.63) | 987.36(1341.32,758.83) | -1.09(-1.20,-0.99) |
| Libya | female | 10426.40(12807.00,8075.01) | 21085.87(25492.36,17096.79) | 1.02 | 860.04(1051.98,669.62) | 761.22(917.35,622.28) | -0.31(-0.48,-0.13) |
| Lithuania | female | 14643.56(16716.63,10798.96) | 7854.86(10623.28,6524.05) | -0.46 | 575.93(665.55,428.79) | 300.35(391.63,240.83) | -2.47(-2.68,-2.26) |
| Luxembourg | female | 2151.17(2562.64,1820.27) | 3285.32(3890.62,2724.03) | 0.53 | 780.20(940.55,642.67) | 696.38(847.07,561.76) | -0.50(-0.56,-0.44) |
| North Macedonia | female | 8921.07(10732.68,7333.44) | 8707.98(10828.07,7026.44) | -0.02 | 954.29(1146.01,781.86) | 614.52(761.54,494.68) | -1.73(-1.84,-1.62) |
| Madagascar | female | 103556.13(122034.04,86531.48) | 153748.54(201494.21,119506.68) | 0.48 | 2271.06(2632.23,1953.42) | 1783.02(2263.48,1416.66) | -1.02(-1.13,-0.92) |
| Malawi | female | 47795.69(64047.91,33313.75) | 57064.13(69428.59,46517.72) | 0.19 | 1353.60(1639.21,1033.27) | 921.96(1089.49,762.42) | -1.45(-1.54,-1.36) |
| Malaysia | female | 63419.86(73359.41,49634.76) | 92208.03(115083.47,75584.25) | 0.45 | 1180.84(1365.85,931.01) | 699.08(891.10,576.55) | -2.26(-2.46,-2.06) |
| Maldives | female | 2070.96(2639.34,1339.85) | 1947.93(2241.77,1677.49) | -0.06 | 3896.74(4670.35,2695.14) | 1392.59(1603.17,1190.76) | -4.00(-4.24,-3.76) |
| Mali | female | 49982.40(59423.15,38510.56) | 84432.31(117549.12,62021.73) | 0.69 | 1960.40(2347.45,1406.98) | 1518.69(2110.57,1066.23) | -0.98(-1.02,-0.93) |
| Malta | female | 1480.75(1870.39,1163.33) | 1963.67(2433.57,1563.70) | 0.33 | 684.82(882.02,531.51) | 570.05(746.24,430.93) | -0.79(-0.84,-0.74) |
| Marshall Islands | female | 321.80(425.62,170.02) | 424.76(591.79,241.95) | 0.32 | 2939.48(3855.32,1486.29) | 2226.10(3101.41,1255.02) | -1.12(-1.28,-0.96) |
| Mauritania | female | 10165.14(12584.87,6799.35) | 12974.36(15698.47,10469.57) | 0.28 | 1578.55(1959.25,1039.91) | 977.57(1189.60,784.70) | -1.56(-1.69,-1.43) |
| Mauritius | female | 5458.24(6087.87,4445.23) | 6034.56(7891.27,4961.00) | 0.11 | 1270.80(1408.41,1026.21) | 740.13(965.34,610.68) | -2.10(-2.25,-1.94) |
| Mexico | female | 257020.95(296851.19,222605.17) | 438085.47(514658.03,372392.11) | 0.70 | 941.36(1040.52,813.97) | 722.53(846.34,616.80) | -1.03(-1.15,-0.92) |
| Republic of Moldova | female | 21112.36(23852.31,15022.15) | 10813.44(14119.98,9011.45) | -0.49 | 846.61(958.74,607.39) | 378.41(489.09,308.15) | -3.29(-3.58,-2.99) |
| Mongolia | female | 7704.39(9266.41,5543.17) | 6872.70(8905.58,5602.18) | -0.11 | 1092.47(1299.93,837.21) | 525.22(666.76,430.89) | -3.16(-3.43,-2.89) |
| Montenegro | female | 986.25(1279.16,766.59) | 1279.31(1573.19,1020.93) | 0.30 | 301.16(390.05,232.52) | 313.24(398.40,243.67) | 0.17(0.13,0.20) |
| Morocco | female | 82948.58(106048.68,50867.31) | 132977.59(165153.42,102586.95) | 0.60 | 996.73(1262.58,604.02) | 834.17(1032.03,650.85) | -0.58(-0.64,-0.52) |
| Mozambique | female | 62939.86(89458.37,41168.63) | 86081.11(109513.23,68181.39) | 0.37 | 1130.97(1441.74,838.27) | 847.90(1065.82,684.01) | -1.04(-1.15,-0.92) |
| Myanmar | female | 486026.42(642596.06,281125.52) | 474040.42(562596.30,323433.02) | -0.02 | 3536.55(4590.33,2061.76) | 1896.67(2224.73,1282.58) | -2.44(-2.59,-2.30) |
| Namibia | female | 7878.62(12237.87,5465.70) | 9928.32(13714.28,7597.80) | 0.26 | 1783.15(2838.16,1224.37) | 1124.02(1564.22,854.44) | -1.81(-1.98,-1.64) |
| Nepal | female | 264376.65(332174.72,182273.02) | 475593.54(576441.24,350352.52) | 0.80 | 5746.61(7201.65,4055.94) | 4317.44(5195.27,3178.94) | -0.95(-1.09,-0.81) |
| Netherlands | female | 81782.74(97649.99,68302.36) | 145413.78(169232.80,110703.44) | 0.78 | 810.39(998.85,654.66) | 948.98(1135.53,725.25) | 0.49(0.34,0.63) |
| New Zealand | female | 27063.60(32544.77,22612.08) | 33194.05(38010.17,27998.29) | 0.23 | 1373.06(1678.04,1133.36) | 945.60(1121.10,796.31) | -1.56(-1.66,-1.46) |
| Nicaragua | female | 12856.70(16325.90,9823.77) | 18530.32(21918.30,14402.74) | 0.44 | 742.34(886.25,613.96) | 730.83(852.03,553.75) | -0.03(-0.24,0.18) |
| Niger | female | 42021.60(56488.87,30150.89) | 85313.52(113000.77,63314.18) | 1.03 | 1822.07(2289.51,1361.95) | 1372.52(1851.98,1028.75) | -1.01(-1.13,-0.90) |
| Nigeria | female | 351381.98(438212.03,279080.46) | 584513.91(725264.42,478220.36) | 0.66 | 1236.67(1560.38,988.12) | 890.28(1096.95,712.40) | -1.15(-1.18,-1.12) |
| Democratic People's Republic of Korea | female | 267532.92(355024.84,188839.09) | 369229.13(444364.76,302688.54) | 0.38 | 3028.65(4034.25,2144.97) | 1979.97(2359.59,1625.54) | -1.62(-1.74,-1.49) |
| Northern Mariana Islands | female | 162.24(198.29,126.76) | 187.52(234.80,155.56) | 0.16 | 1513.92(1848.48,1190.71) | 879.18(1099.95,732.69) | -1.91(-2.01,-1.82) |
| Norway | female | 25291.44(30638.73,20696.40) | 36325.88(42189.33,25706.46) | 0.44 | 810.92(1004.28,645.42) | 838.41(1008.59,607.97) | 0.07(-0.05,0.20) |
| Oman | female | 3239.43(4249.96,2429.60) | 6340.59(8045.96,4998.99) | 0.96 | 792.20(1073.40,573.84) | 625.38(754.28,493.19) | -0.58(-0.73,-0.43) |
| Pakistan | female | 497621.76(660891.49,392485.24) | 877676.84(1076952.19,731557.15) | 0.76 | 1842.95(2515.07,1422.81) | 1570.69(1948.14,1307.54) | -0.70(-0.88,-0.52) |
| Palestine | female | 5597.14(7106.80,4140.20) | 10088.19(12157.40,8224.08) | 0.80 | 926.90(1181.96,689.64) | 636.08(746.49,529.00) | -1.46(-1.58,-1.35) |
| Panama | female | 7114.56(8696.08,5816.85) | 12342.30(15028.80,10011.29) | 0.73 | 706.76(835.88,602.67) | 578.12(708.89,466.15) | -0.78(-0.88,-0.69) |
| Papua New Guinea | female | 54583.41(69666.41,43641.12) | 113872.90(150034.54,87423.15) | 1.09 | 5165.00(6311.61,4154.99) | 4522.15(5743.50,3473.40) | -0.37(-0.44,-0.30) |
| Paraguay | female | 10632.82(13642.31,8392.29) | 17976.83(23301.56,13695.83) | 0.69 | 598.19(734.09,498.05) | 560.35(716.55,429.70) | -0.19(-0.37,-0.01) |
| Peru | female | 87166.86(111155.83,68368.02) | 94485.88(117441.22,73776.74) | 0.08 | 855.47(1046.36,705.35) | 566.94(704.24,442.92) | -1.38(-1.55,-1.22) |
| Philippines | female | 341421.33(398188.18,282991.19) | 470379.73(567142.30,394235.10) | 0.38 | 1616.35(1842.21,1359.09) | 1020.19(1258.66,856.22) | -1.58(-1.68,-1.47) |
| Poland | female | 191978.30(234547.44,155754.76) | 169981.44(208020.35,140221.18) | -0.11 | 824.17(1021.52,658.43) | 553.60(697.80,440.15) | -1.58(-1.71,-1.45) |
| Portugal | female | 65920.56(80875.07,53569.90) | 78996.53(94787.49,64711.98) | 0.20 | 977.81(1231.91,777.13) | 747.75(963.22,580.70) | -0.93(-1.11,-0.74) |
| Puerto Rico | female | 18217.00(22386.70,14774.80) | 21747.91(25979.79,17421.83) | 0.19 | 963.70(1186.85,778.68) | 795.25(1009.21,620.50) | -1.13(-1.35,-0.90) |
| Qatar | female | 676.91(847.45,528.57) | 2655.14(3382.03,2058.88) | 2.92 | 946.13(1206.45,763.40) | 734.66(937.78,598.20) | -0.84(-0.96,-0.72) |
| Romania | female | 137105.07(157884.79,93524.86) | 82572.19(107394.22,67393.93) | -0.40 | 974.30(1133.42,664.10) | 497.34(641.48,395.17) | -2.37(-2.58,-2.16) |
| Russian Federation | female | 722157.37(838252.33,520205.47) | 435708.36(540267.87,370918.32) | -0.40 | 686.57(807.28,502.40) | 373.58(463.25,312.69) | -2.57(-2.76,-2.38) |
| Rwanda | female | 70097.34(90352.85,47188.75) | 67552.23(94240.48,51320.37) | -0.04 | 2636.90(3242.37,1844.33) | 1426.88(2011.48,1097.44) | -2.53(-2.70,-2.37) |
| Saint Lucia | female | 465.54(566.86,385.17) | 617.86(734.32,501.39) | 0.33 | 771.37(910.76,649.71) | 641.81(783.45,512.30) | -0.93(-1.09,-0.76) |
| Saint Vincent and the Grenadines | female | 247.07(323.60,192.95) | 258.28(324.95,203.88) | 0.05 | 477.71(608.34,382.14) | 454.24(580.13,351.81) | -0.29(-0.42,-0.17) |
| Samoa | female | 1245.98(1863.79,902.87) | 1358.96(1757.79,1015.56) | 0.09 | 2483.66(3730.69,1810.92) | 1734.02(2245.68,1295.05) | -1.31(-1.42,-1.20) |
| Sao Tome and Principe | female | 914.50(1074.62,744.46) | 1238.02(1570.79,900.47) | 0.35 | 2304.02(2671.17,1887.55) | 1980.30(2523.57,1379.47) | -0.72(-0.89,-0.55) |
| Saudi Arabia | female | 41009.58(56703.70,30264.04) | 77882.84(94685.68,63012.17) | 0.90 | 1284.57(1837.96,926.05) | 862.90(1040.34,706.31) | -1.41(-1.46,-1.35) |
| Senegal | female | 31293.26(38298.07,24488.05) | 49830.30(61651.13,39969.94) | 0.59 | 1393.60(1682.68,1093.17) | 1026.90(1253.58,838.12) | -0.83(-1.05,-0.60) |
| Serbia | female | 36684.03(44578.56,31178.48) | 42392.73(49901.67,34919.47) | 0.16 | 662.33(805.10,563.76) | 576.97(679.83,475.41) | -0.61(-0.76,-0.47) |
| Seychelles | female | 255.66(288.76,219.99) | 338.82(387.41,286.00) | 0.33 | 760.53(855.41,654.58) | 632.49(727.99,532.19) | -0.64(-0.70,-0.59) |
| Sierra Leone | female | 17643.25(22071.54,13390.79) | 31456.26(39373.06,24636.75) | 0.78 | 1427.09(1770.13,1096.55) | 1253.50(1589.66,992.85) | -0.17(-0.31,-0.04) |
| Singapore | female | 10680.21(12735.11,7934.18) | 10761.37(14216.76,8529.35) | 0.01 | 850.26(998.32,624.13) | 322.09(426.77,250.79) | -3.48(-3.80,-3.17) |
| Slovakia | female | 12636.67(16406.36,10531.21) | 15230.57(18344.14,12581.91) | 0.21 | 405.71(513.10,335.22) | 368.81(451.86,297.45) | -0.13(-0.25,-0.02) |
| Slovenia | female | 7113.83(8717.23,5580.55) | 7549.47(9198.10,6087.06) | 0.06 | 546.77(678.15,427.14) | 414.27(527.75,322.00) | -1.25(-1.37,-1.12) |
| Solomon Islands | female | 2478.90(3274.16,1879.69) | 4763.55(5872.30,3750.07) | 0.92 | 3186.92(4338.75,2368.36) | 2655.17(3226.50,2072.12) | -0.58(-0.63,-0.53) |
| Somalia | female | 48668.91(75936.17,30300.54) | 96046.49(164609.47,65177.21) | 0.97 | 2080.35(3424.56,1339.06) | 1609.58(2838.00,1057.44) | -0.75(-0.85,-0.64) |
| South Africa | female | 235307.47(273192.56,196330.50) | 288531.15(334314.80,252759.39) | 0.23 | 1621.68(1899.05,1348.35) | 1099.45(1271.12,965.12) | -1.57(-1.91,-1.23) |
| Republic of Korea | female | 140194.89(162043.25,104242.92) | 159539.58(188401.09,132897.76) | 0.14 | 905.11(1031.97,620.93) | 399.22(490.20,325.57) | -3.24(-3.43,-3.05) |
| South Sudan | female | 27044.53(38255.74,17192.16) | 28289.29(38053.61,21425.91) | 0.05 | 1337.30(1756.35,887.26) | 923.86(1276.14,696.66) | -1.29(-1.45,-1.12) |
| Spain | female | 186552.68(212467.76,160479.50) | 272000.58(316779.74,220133.43) | 0.46 | 656.90(766.47,558.92) | 550.94(665.85,455.73) | -0.69(-0.76,-0.63) |
| Sri Lanka | female | 103903.30(118157.94,86078.83) | 139356.71(174196.66,103177.84) | 0.34 | 1875.89(2131.85,1513.64) | 1081.26(1339.15,800.34) | -1.74(-1.84,-1.64) |
| Sudan | female | 101478.67(135224.37,74414.86) | 120299.77(154318.65,92319.61) | 0.19 | 1589.56(2218.06,1079.24) | 1005.16(1354.32,759.94) | -1.69(-1.75,-1.63) |
| Suriname | female | 1282.70(1556.71,1049.76) | 1962.59(2362.57,1605.70) | 0.53 | 764.30(903.99,637.80) | 653.33(789.87,531.87) | -0.73(-0.85,-0.61) |
| Eswatini | female | 4083.98(5952.82,2990.89) | 5015.07(6423.43,3800.33) | 0.23 | 1860.53(2867.52,1337.90) | 1271.67(1656.43,945.19) | -1.17(-1.43,-0.91) |
| Sweden | female | 56229.41(69059.13,44981.17) | 75398.79(88987.56,57985.22) | 0.34 | 867.32(1115.12,673.23) | 846.63(1053.49,652.07) | -0.08(-0.12,-0.05) |
| Switzerland | female | 32316.15(38581.06,26647.97) | 44791.63(53203.94,37091.82) | 0.39 | 653.78(814.99,516.63) | 616.86(772.30,486.39) | -0.27(-0.31,-0.23) |
| Syrian Arab Republic | female | 43373.20(53023.13,33050.01) | 56261.36(69248.41,46590.12) | 0.30 | 1169.33(1450.83,899.94) | 928.99(1144.80,767.56) | -0.94(-1.08,-0.80) |
| Taiwan (Province of China) | female | 61352.11(69543.67,51417.02) | 74617.69(99083.66,60872.42) | 0.22 | 832.51(932.20,695.45) | 429.55(556.25,347.13) | -2.70(-2.83,-2.57) |
| Tajikistan | female | 23631.63(26429.46,19493.79) | 27351.83(36984.70,22213.53) | 0.16 | 1362.35(1519.16,1097.74) | 965.10(1401.40,769.78) | -1.47(-1.59,-1.34) |
| United Republic of Tanzania | female | 109637.89(135296.77,87720.11) | 206232.99(267409.97,162637.32) | 0.88 | 1153.76(1351.26,964.21) | 978.29(1196.39,802.31) | -0.41(-0.49,-0.33) |
| Thailand | female | 261824.05(311856.86,200152.98) | 250752.33(314990.10,205846.84) | -0.04 | 1304.73(1561.86,939.14) | 531.43(657.51,432.60) | -3.63(-3.81,-3.45) |
| Bahamas | female | 600.87(769.61,479.60) | 974.77(1202.56,778.19) | 0.62 | 519.44(639.31,425.97) | 500.75(629.50,393.55) | -0.20(-0.26,-0.13) |
| Gambia | female | 3405.75(4564.80,2587.17) | 7772.58(10662.53,6006.23) | 1.28 | 1377.66(1829.86,1039.43) | 1207.27(1662.12,942.33) | -0.47(-0.65,-0.28) |
| Timor-Leste | female | 5570.05(8277.94,3603.73) | 6322.45(8557.42,4539.71) | 0.14 | 2325.30(2983.30,1652.18) | 1399.19(1925.48,1025.93) | -2.08(-2.33,-1.82) |
| Togo | female | 15725.68(19208.04,12773.27) | 29356.78(37702.04,23321.86) | 0.87 | 1597.48(1950.81,1278.85) | 1109.76(1435.55,888.54) | -1.13(-1.31,-0.94) |
| Tonga | female | 386.11(474.29,316.41) | 380.87(481.13,285.14) | -0.01 | 1180.42(1454.50,966.92) | 829.88(1054.60,615.17) | -1.28(-1.40,-1.16) |
| Trinidad and Tobago | female | 3416.29(4008.36,2884.40) | 3620.41(4903.74,2770.49) | 0.06 | 642.37(742.46,555.38) | 481.18(633.21,363.42) | -1.34(-1.65,-1.04) |
| Tunisia | female | 22486.16(29201.35,17938.61) | 37032.51(45971.92,29834.39) | 0.65 | 781.18(1032.81,619.52) | 604.38(748.84,486.58) | -0.92(-0.97,-0.87) |
| Turkey | female | 273722.22(322635.92,228433.15) | 418551.12(494475.34,339680.88) | 0.53 | 1283.61(1495.94,1069.00) | 941.30(1113.41,763.92) | -1.10(-1.26,-0.93) |
| Turkmenistan | female | 14008.48(15911.31,9280.80) | 8809.29(15735.46,6732.20) | -0.37 | 1097.76(1236.60,732.94) | 386.76(720.31,292.99) | -4.26(-4.68,-3.82) |
| Uganda | female | 80173.83(115071.23,54689.28) | 124074.53(169571.86,94340.43) | 0.55 | 1402.61(2010.14,954.40) | 973.37(1330.46,752.00) | -1.51(-1.63,-1.39) |
| Ukraine | female | 376368.42(427850.45,258161.29) | 134272.44(230009.07,104681.95) | -0.64 | 907.15(1042.95,640.08) | 376.94(582.50,291.12) | -3.85(-4.17,-3.54) |
| United Arab Emirates | female | 4411.07(5678.44,3324.66) | 19427.42(24192.72,15150.32) | 3.40 | 1726.15(2338.86,1267.81) | 1172.52(1422.55,948.39) | -1.15(-1.70,-0.59) |
| United Kingdom | female | 540000.05(648360.74,453381.66) | 649390.31(749525.35,521446.46) | 0.20 | 1277.34(1567.47,1032.88) | 1158.71(1395.33,924.61) | -0.47(-0.52,-0.42) |
| United States of America | female | 1999698.79(2329281.91,1717588.35) | 3643271.28(4098272.92,2978718.60) | 0.82 | 1226.32(1456.89,1034.33) | 1409.84(1633.69,1157.69) | 0.69(0.61,0.77) |
| Uruguay | female | 13440.46(16963.22,11427.22) | 21098.19(24173.85,17626.58) | 0.57 | 698.69(861.45,586.81) | 787.65(918.42,653.34) | 0.45(0.42,0.49) |
| Uzbekistan | female | 78129.58(90129.14,55394.60) | 79897.05(116651.80,64392.24) | 0.02 | 1080.50(1235.84,743.14) | 669.21(1082.49,538.13) | -2.58(-3.02,-2.14) |
| Vanuatu | female | 1089.77(1700.64,719.05) | 2139.00(3068.30,1521.35) | 0.96 | 2950.13(4724.40,1920.31) | 2273.04(3286.03,1625.28) | -1.27(-1.45,-1.09) |
| Venezuela (Bolivarian Republic of) | female | 48559.51(58582.95,39918.02) | 94156.90(115660.93,76627.66) | 0.94 | 657.10(777.16,562.69) | 636.02(777.23,519.28) | -0.35(-0.50,-0.20) |
| Viet nam | female | 271517.69(353598.16,149777.23) | 372588.49(468528.76,226106.34) | 0.37 | 1095.25(1454.10,566.29) | 751.18(944.75,453.38) | -1.30(-1.36,-1.24) |
| Virginia | female | 47352.34(55639.77,40731.74) | 91746.90(108205.82,73466.83) | 0.94 | 1265.10(1502.69,1074.05) | 1390.57(1666.80,1104.69) | 0.47(0.39,0.55) |
| Yemen | female | 64275.16(87303.78,46308.71) | 109460.54(135597.55,86237.89) | 0.70 | 1786.08(2728.76,1202.74) | 1231.86(1541.35,958.70) | -1.46(-1.58,-1.33) |
| Zambia | female | 36553.74(50053.40,25834.51) | 50788.25(61442.00,41862.23) | 0.39 | 1428.77(1739.42,1124.36) | 958.64(1155.28,784.15) | -1.51(-1.56,-1.46) |
| Zimbabwe | female | 29665.34(34168.38,24514.40) | 54944.99(72625.07,37416.81) | 0.85 | 1141.57(1304.60,931.31) | 1160.63(1548.27,769.37) | 0.96(0.53,1.40) |
| Monaco | female | 179.27(222.83,146.87) | 244.19(285.55,202.81) | 0.36 | 595.59(748.17,470.61) | 632.94(780.59,504.43) | 0.36(0.26,0.45) |
| San Marino | female | 93.23(113.18,76.35) | 170.09(210.26,135.09) | 0.82 | 602.79(748.87,483.56) | 608.74(773.99,476.46) | 0.21(0.13,0.29) |
| Saint Kitts and Nevis | female | 166.90(199.35,138.81) | 190.03(233.34,151.27) | 0.14 | 800.95(952.27,670.22) | 626.50(779.56,495.07) | -0.76(-0.88,-0.64) |
| Cook Islands | female | 121.66(144.25,100.03) | 121.13(142.75,101.85) | 0.00 | 1852.81(2192.08,1538.39) | 1048.31(1236.97,881.94) | -1.97(-2.13,-1.82) |
| Nauru | female | 73.64(95.38,52.14) | 61.23(81.95,38.08) | -0.17 | 2873.75(3578.30,2148.05) | 2087.62(2740.09,1317.15) | -1.13(-1.43,-0.83) |
| Niue | female | 25.66(32.18,18.48) | 14.93(19.30,10.52) | -0.42 | 2008.77(2515.77,1464.36) | 1366.59(1772.34,972.05) | -1.48(-1.56,-1.39) |
| Palau | female | 149.06(197.21,113.51) | 189.20(231.39,150.07) | 0.27 | 2827.51(3768.96,2146.44) | 2030.13(2458.12,1618.93) | -1.05(-1.16,-0.95) |
| Tokelau | female | 23.85(30.03,18.54) | 11.97(15.17,8.85) | -0.50 | 3178.33(3995.34,2475.64) | 1832.96(2330.03,1369.62) | -2.01(-2.08,-1.93) |
| Tuvalu | female | 153.75(196.88,119.16) | 107.08(139.22,75.78) | -0.30 | 3748.02(4886.28,2887.68) | 2039.35(2639.70,1444.75) | -2.17(-2.30,-2.03) |

**Supplementary Table 6. Age distribution of incidence rate for chronic obstructive pulmonary disease in different countries in 2019.**

| 2019incidence rate | 15 to 19 | 20 to 24 | 25 to 29 | 30 to 34 | 35 to 39 | 40 to 44 | 45 to 49 | 50 to 54 | 55 to 59 | 60 to 64 | 65 to 69 | 70 to 74 | 75 to 79 | 80 plus | 80-84 | 85-89 | 90-94 | all ages |
| --- | --- | --- | --- | --- | --- | --- | --- | --- | --- | --- | --- | --- | --- | --- | --- | --- | --- | --- |
| Afghanistan | 28384.14086 | 21948.97131 | 18925.71074 | 12841.78287 | 9790.669518 | 10689.6399 | 17353.50119 | 13653.06177 | 7476.185947 | 4818.517553 | 5167.757429 | 4663.272483 | 3195.465842 | 3105.503139 | 1866.907387 | 889.2034257 | 285.7561565 | 360801.1905 |
| Albania | 1181.079455 | 1166.440538 | 977.8000969 | 973.9050464 | 1083.62743 | 1249.23638 | 1609.813491 | 2053.180375 | 2593.838344 | 2828.405062 | 2635.74281 | 2606.241044 | 2215.971397 | 2770.542015 | 1644.14788 | 774.0118165 | 277.4818679 | 29866.84122 |
| Algeria | 17292.32009 | 14818.20247 | 15625.39201 | 17084.53712 | 18139.27125 | 17888.68846 | 18585.84985 | 18702.63193 | 18663.29558 | 18263.13691 | 17007.08421 | 13070.60655 | 10410.35827 | 13028.89009 | 7868.306402 | 4350.357287 | 714.1424212 | 365668.175 |
| American Samoa | 31.5642081 | 21.61309563 | 16.52112444 | 13.72913665 | 15.35819412 | 18.55938997 | 21.30144915 | 25.49217106 | 26.3065208 | 25.33530621 | 23.08432063 | 20.00556254 | 14.96019512 | 21.98190474 | 11.11653636 | 6.920091147 | 3.08018403 | 496.6571807 |
| Andorra | 25.04427181 | 16.5383092 | 14.66861648 | 15.73277386 | 21.4163815 | 31.74456117 | 43.10590101 | 46.4505635 | 47.57047362 | 49.4239066 | 50.34018674 | 51.20765205 | 41.93688974 | 86.58179939 | 33.21286899 | 29.44925675 | 17.26069218 | 646.202679 |
| Angola | 16217.73402 | 9268.118866 | 6596.723163 | 6005.979259 | 6295.200854 | 7273.897691 | 7170.136202 | 7325.075622 | 7317.952822 | 6168.57985 | 5133.337714 | 3200.168055 | 2292.1213 | 1804.815857 | 1070.498563 | 505.8761777 | 183.4530414 | 254221.6847 |
| Antigua and Barbuda | 61.4480618 | 45.97951039 | 30.49106407 | 22.87507265 | 22.74679131 | 26.06819287 | 32.01443352 | 37.01011563 | 37.79494661 | 34.41280006 | 30.10738438 | 27.37503313 | 19.07782171 | 22.74700202 | 11.34548488 | 6.982729972 | 3.270631373 | 836.2566345 |
| Argentina | 24383.24362 | 20592.63069 | 16122.32183 | 14576.8765 | 16455.19553 | 21417.76782 | 24633.2664 | 26473.60832 | 32464.96277 | 38449.07117 | 39860.78195 | 35678.85285 | 25398.70464 | 43210.25922 | 19760.87116 | 14262.81861 | 6843.399786 | 546399.1557 |
| Armenia | 626.5408421 | 588.4706586 | 811.2863226 | 981.2751649 | 1070.26935 | 1130.393194 | 1319.901377 | 1672.979059 | 2568.930205 | 3217.910307 | 3152.204789 | 2446.430623 | 2002.83167 | 3914.909748 | 2384.451459 | 1066.284328 | 410.9762983 | 30266.43833 |
| Australia | 6717.614219 | 5278.233466 | 5209.340818 | 5483.671045 | 6156.86663 | 7903.297641 | 12427.39788 | 14755.08921 | 19422.58512 | 26605.1309 | 34657.11825 | 39026.72702 | 30167.94483 | 49807.29186 | 23341.93794 | 15694.912 | 8150.781641 | 334143.3796 |
| Austria | 2491.897114 | 1979.208565 | 1858.332562 | 1772.578332 | 1913.509302 | 2434.455759 | 3602.462419 | 4678.968498 | 4953.878211 | 5485.565059 | 5457.86695 | 4646.99913 | 5126.853645 | 12373.67017 | 5270.581446 | 4094.753319 | 2236.675666 | 70487.09025 |
| Azerbaijan | 2471.207326 | 2400.852479 | 3006.723495 | 3398.535003 | 3351.420161 | 3518.741609 | 4471.545063 | 5814.254851 | 7009.544888 | 6290.069152 | 4287.348525 | 2424.270689 | 1945.049379 | 2758.707107 | 1937.925403 | 685.798444 | 120.9602258 | 70776.70484 |
| Bahrain | 531.1230892 | 386.4898582 | 415.6119827 | 656.9066643 | 1147.696773 | 1060.693966 | 941.2318466 | 957.991757 | 1081.592962 | 1058.336562 | 744.5423982 | 437.9718966 | 264.7457294 | 241.7469611 | 162.9248136 | 67.19927562 | 10.84149562 | 12828.24429 |
| Bangladesh | 27105.49629 | 29962.3947 | 32414.15727 | 37339.63815 | 43749.18412 | 69380.62031 | 96640.22367 | 109612.611 | 122535.0997 | 135428.2769 | 131669.2174 | 117358.8639 | 78675.65854 | 81890.92544 | 46779.25581 | 25650.86471 | 8032.14755 | 1229420.837 |
| Barbados | 198.2076212 | 141.4725429 | 109.0417417 | 83.06794417 | 85.76015508 | 96.87278215 | 136.8905524 | 167.2438809 | 199.5897345 | 217.281481 | 201.893499 | 195.8194051 | 135.0773368 | 232.0880336 | 102.5189053 | 79.06146807 | 40.66881494 | 3379.683192 |
| Belarus | 2521.473099 | 2085.016564 | 3000.434597 | 4460.884659 | 5210.195577 | 5774.464623 | 6609.251472 | 7704.234833 | 10467.51916 | 10953.13196 | 8306.56485 | 5088.994758 | 2943.142294 | 5526.228868 | 2587.415566 | 1701.303756 | 977.5519774 | 99798.03308 |
| Belgium | 3315.847477 | 2429.709177 | 2184.466903 | 2022.452435 | 2307.894824 | 3723.998842 | 5304.866811 | 5599.974455 | 6035.740419 | 8314.890022 | 9366.681419 | 9194.198413 | 7513.114362 | 18342.19459 | 8463.938979 | 6179.245159 | 2796.589677 | 101925.5441 |
| Belize | 430.0929598 | 285.6632549 | 180.7699539 | 135.6161889 | 130.5666648 | 153.8639522 | 191.7677002 | 196.5013163 | 196.5774136 | 169.1434324 | 137.0864957 | 117.069554 | 81.07228171 | 102.9399196 | 51.86484634 | 29.88957303 | 15.26623112 | 5271.820345 |
| Benin | 6220.599442 | 4508.716037 | 2914.569495 | 2510.228397 | 2871.342853 | 3131.270371 | 2902.443462 | 2654.488634 | 2635.494727 | 2161.215127 | 1437.454342 | 1180.272761 | 916.0876999 | 1016.985219 | 576.7317441 | 298.239081 | 111.9874616 | 93621.99862 |
| Bermuda | 28.34653429 | 20.57696577 | 16.16040657 | 15.25552849 | 18.01180495 | 22.62603861 | 32.29694965 | 46.31309308 | 63.91290244 | 72.82475206 | 71.12397019 | 75.16965133 | 64.37403131 | 96.64807244 | 46.45050818 | 30.44422566 | 14.54714724 | 851.833768 |
| Bhutan | 130.5625805 | 165.1573167 | 177.7304089 | 204.0351517 | 242.5431445 | 319.8909518 | 400.2654813 | 441.5736556 | 496.8555627 | 567.581377 | 598.7080588 | 543.4666529 | 392.14135 | 407.5497262 | 241.5085613 | 114.7164935 | 39.83341086 | 5626.03874 |
| Bolivia (Plurinational State of) | 5157.687401 | 3178.52313 | 2641.278495 | 2396.053598 | 2698.32769 | 3391.178145 | 4154.747536 | 4949.85059 | 6395.438777 | 7628.290453 | 8645.916025 | 8841.333832 | 7570.068265 | 8760.068037 | 5043.358706 | 2613.677881 | 891.8292733 | 170324.7405 |
| Bosnia and Herzegovina | 1511.548037 | 1510.645292 | 1132.281133 | 1288.33633 | 1710.843768 | 2326.741067 | 2664.986545 | 3380.071114 | 4291.179254 | 4731.58752 | 4659.618495 | 3689.409253 | 2929.36341 | 3422.044331 | 2219.578649 | 912.1135487 | 240.5877755 | 44485.01434 |
| Botswana | 889.9877945 | 662.2538961 | 725.1388043 | 881.5658403 | 1059.055048 | 1106.118033 | 1126.621809 | 1036.929394 | 1023.850389 | 1014.575177 | 978.4198486 | 752.8108154 | 437.9689388 | 401.4008906 | 240.8373878 | 115.8634228 | 36.32807121 | 18581.54575 |
| Brazil | 134277.5318 | 72369.26418 | 54065.55624 | 45408.05257 | 44872.85696 | 59488.70016 | 81450.16536 | 93101.31407 | 95359.92861 | 100234.5621 | 102878.2396 | 97251.95322 | 83479.32852 | 140404.9185 | 67747.27832 | 43284.58564 | 21248.42291 | 2410958.97 |
| Brunei Darussalam | 197.3522249 | 177.8333493 | 155.3141368 | 152.8034747 | 150.4605021 | 154.0233044 | 167.8148337 | 181.1515637 | 210.7064466 | 302.0574179 | 325.4239696 | 233.9863314 | 132.267353 | 94.74204521 | 66.00447456 | 23.53111373 | 4.523862473 | 3667.288154 |
| Bulgaria | 2376.061092 | 2040.081853 | 2151.846993 | 2810.415408 | 3612.40586 | 5506.60088 | 6816.138017 | 6810.768228 | 7306.971111 | 7857.064161 | 7876.323162 | 7385.434812 | 5081.721961 | 8156.227077 | 4116.591974 | 2998.651102 | 910.1400893 | 85538.12372 |
| Burkina Faso | 10914.4588 | 7409.661702 | 4831.889414 | 4420.075722 | 5082.857256 | 5010.228643 | 4341.888846 | 4107.905965 | 4182.566385 | 3309.33005 | 2040.556133 | 1579.102169 | 1133.886159 | 1257.04843 | 709.1858747 | 375.5447556 | 137.7637121 | 165387.5343 |
| Burundi | 8433.924725 | 4758.805712 | 3002.635889 | 2762.792737 | 3228.975941 | 2997.37146 | 2589.01148 | 2653.703004 | 2887.87239 | 2556.268693 | 1869.412718 | 1234.794384 | 824.1179794 | 823.5868961 | 497.1583422 | 238.6754389 | 71.99850427 | 120893.3798 |
| Cambodia | 5297.322639 | 4288.873307 | 3288.533184 | 2707.302314 | 2588.749496 | 2992.472792 | 4032.416003 | 5228.63689 | 5722.460619 | 5647.500997 | 5079.414808 | 4260.830231 | 2866.906741 | 3759.241488 | 2096.139124 | 1187.188108 | 386.7750658 | 102125.3864 |
| Cameroon | 13736.65713 | 9720.129195 | 6786.884608 | 6271.743707 | 6835.941538 | 7570.788954 | 7323.098125 | 6600.626134 | 6537.806437 | 5466.216801 | 3811.485223 | 3068.67584 | 2193.475395 | 2422.56455 | 1404.368309 | 720.9610969 | 245.821 | 195270.3826 |
| Canada | 15060.17716 | 13765.85321 | 12325.02174 | 12630.28997 | 16265.34032 | 21208.23645 | 29429.70426 | 39935.45827 | 54084.45329 | 63379.60185 | 67597.79931 | 70526.80137 | 58197.88732 | 84567.39204 | 42296.01907 | 26105.57748 | 12091.94364 | 663786.1219 |
| Cabo Verde | 230.5061373 | 193.3105143 | 157.9214431 | 160.9670093 | 168.2822845 | 157.2194944 | 146.6192703 | 153.6530298 | 170.9364559 | 152.3551932 | 97.25852548 | 66.0324574 | 59.3531666 | 163.1473494 | 75.57788809 | 54.28450885 | 23.87366484 | 3680.242153 |
| Central African Republic | 3247.289938 | 2183.394977 | 1510.823155 | 1326.667539 | 1358.354702 | 1481.586477 | 1580.140578 | 1544.207003 | 1423.553081 | 1230.483375 | 900.2515654 | 629.3505426 | 376.4798454 | 304.275102 | 193.8742379 | 80.53110715 | 24.04881425 | 44749.66972 |
| Chad | 7161.754604 | 4530.041957 | 2869.037229 | 2466.660364 | 2730.562637 | 2922.024341 | 2846.594672 | 2669.512706 | 2617.654347 | 2138.415632 | 1583.270483 | 1295.066025 | 926.0439573 | 1001.325427 | 561.7523317 | 320.4174182 | 93.49121726 | 109485.4576 |
| Chile | 6716.95025 | 6057.911541 | 5392.040878 | 4918.017465 | 5390.38722 | 6016.936704 | 7821.043451 | 10919.42064 | 15973.37568 | 19367.46108 | 19737.83008 | 18572.35198 | 13892.08559 | 24568.63454 | 11301.16001 | 8433.604016 | 3747.360427 | 210644.5955 |
| China | 222823.1764 | 168811.8389 | 188409.4535 | 224763.3969 | 230317.2624 | 327443.2773 | 604999.1937 | 924253.8401 | 970369.9326 | 1170141.43 | 1489665.893 | 1351106.718 | 942950.908 | 1076141.175 | 652692.4708 | 317662.0072 | 87023.64068 | 11907502.35 |
| Colombia | 24519.49178 | 16914.39939 | 13722.33922 | 11045.88157 | 11031.98204 | 13179.14792 | 19169.59616 | 25111.57058 | 27922.8637 | 27647.59097 | 26136.58984 | 27191.10198 | 25508.44388 | 47446.93365 | 23090.71564 | 14620.34369 | 6958.106159 | 491691.3072 |
| Comoros | 486.3982647 | 305.4928046 | 207.7944403 | 184.5536439 | 219.9614299 | 237.0948954 | 227.3766933 | 231.0133969 | 239.9975284 | 211.1628777 | 163.6331342 | 156.7948391 | 111.6518125 | 116.6829097 | 68.30948855 | 33.51246143 | 11.84653418 | 6457.581248 |
| Congo | 2918.318388 | 1801.988948 | 1397.112066 | 1398.278201 | 1669.308491 | 1946.084379 | 2076.932729 | 1917.526559 | 1752.014446 | 1523.99451 | 1284.403542 | 991.0209989 | 625.5068047 | 599.0809565 | 361.3533323 | 175.1398496 | 51.78578871 | 46614.91176 |
| Costa Rica | 2937.200196 | 2240.483012 | 1975.00482 | 1712.076472 | 1475.021682 | 1643.81994 | 2189.468752 | 3067.157585 | 3943.365953 | 4507.772007 | 4406.201245 | 4538.8261 | 3892.803836 | 6008.937304 | 2921.614939 | 1815.201303 | 867.4132287 | 66457.16915 |
| Côte d'Ivoire | 13886.14108 | 10506.76347 | 7414.540432 | 6990.826578 | 7903.254485 | 7513.46893 | 6313.244675 | 5828.578662 | 6078.460824 | 4741.877017 | 2856.037843 | 2073.312029 | 1482.998462 | 1661.920999 | 931.1074324 | 510.0746393 | 178.1200674 | 200427.4831 |
| Croatia | 1650.3116 | 1732.717714 | 1425.601439 | 1726.145286 | 2422.78158 | 3027.549905 | 3822.35367 | 5038.278183 | 5856.482479 | 6716.815448 | 6479.070589 | 5511.619236 | 5078.803548 | 7835.670278 | 4384.569524 | 2599.306252 | 742.2059309 | 64552.22043 |
| Cuba | 6880.33469 | 5311.977861 | 4141.975143 | 3357.283921 | 2644.210138 | 3911.864393 | 8247.57932 | 10361.57003 | 9676.209064 | 6940.485009 | 6422.178535 | 5851.560928 | 4464.785297 | 6931.884585 | 3130.666276 | 2059.130652 | 1173.415101 | 132094.6011 |
| Cyprus | 426.5172183 | 347.7062483 | 361.9696441 | 380.7332206 | 432.5852908 | 410.4656907 | 374.8236959 | 414.2707679 | 533.4803731 | 793.7084937 | 974.3992286 | 1011.772186 | 819.8833608 | 1182.972545 | 703.8934979 | 375.560604 | 91.73411875 | 10688.36696 |
| Czechia | 3229.213287 | 2847.882732 | 3162.900723 | 3811.436482 | 5243.515742 | 8380.773579 | 10011.73818 | 10085.12213 | 11542.31455 | 14080.5518 | 16676.18586 | 15949.66705 | 10364.05936 | 11209.85163 | 5605.969505 | 3578.697901 | 1561.866458 | 141984.5762 |
| Democratic Republic of the Congo | 49018.21857 | 29780.24423 | 21038.65022 | 18230.02658 | 18595.83705 | 19864.76912 | 22131.25508 | 23083.60672 | 22879.79445 | 20509.77329 | 16599.98974 | 12510.79923 | 8276.372708 | 9349.518833 | 5253.213763 | 2842.357185 | 1007.948465 | 700746.5132 |
| Denmark | 2011.337753 | 1458.448135 | 1171.682977 | 937.0786466 | 981.9941982 | 1875.501917 | 2641.729974 | 2887.328429 | 2811.037586 | 4745.99431 | 6047.791716 | 6839.70042 | 4750.720165 | 6649.661356 | 3392.342374 | 1962.666692 | 939.6547576 | 55022.51714 |
| Djibouti | 666.5167371 | 451.4242573 | 344.1753469 | 376.3402274 | 470.7802757 | 466.6293487 | 397.0005275 | 370.5411023 | 369.0699793 | 314.0486457 | 227.7476926 | 170.3135566 | 95.67467045 | 70.17375944 | 45.17399397 | 18.41062416 | 5.396659261 | 10974.88917 |
| Dominica | 61.80336751 | 39.94861035 | 26.21591385 | 19.63546162 | 17.43955937 | 17.95266223 | 25.66361512 | 34.01954474 | 37.62165969 | 37.54179955 | 33.30933366 | 30.04240271 | 27.20230927 | 40.76283817 | 21.33381393 | 12.51572173 | 5.336272984 | 796.9518528 |
| Dominican Republic | 7146.017425 | 5109.451117 | 3443.766559 | 2465.56731 | 2266.978049 | 2436.143851 | 2790.114524 | 2941.623981 | 2876.151649 | 2586.314565 | 2275.814353 | 2032.639714 | 1498.902884 | 2703.864853 | 1216.887999 | 917.933138 | 420.403187 | 96450.07965 |
| Ecuador | 7360.618774 | 4368.504885 | 3500.970525 | 3380.072839 | 4038.024084 | 4836.783745 | 6122.050456 | 7330.107311 | 9444.672442 | 10782.55976 | 12652.90261 | 13630.15669 | 12801.52108 | 20076.80655 | 10649.2266 | 6566.64133 | 2467.963855 | 229588.5711 |
| Egypt | 52997.18654 | 40859.05584 | 36767.91575 | 37569.92141 | 39347.39905 | 47757.45329 | 52391.54063 | 52229.36928 | 54177.33621 | 53109.82062 | 46714.07393 | 33306.92737 | 19492.41138 | 15494.67707 | 9477.992655 | 4448.231529 | 1278.088389 | 934265.1403 |
| El Salvador | 4526.031045 | 3237.20081 | 2411.248999 | 1669.837334 | 1456.939873 | 1806.145961 | 2385.82494 | 2853.831456 | 3215.797704 | 3346.503588 | 3507.54522 | 3964.237449 | 3790.252957 | 6777.230058 | 2983.309515 | 2118.979377 | 1071.580987 | 79608.10869 |
| Equatorial Guinea | 925.5759993 | 532.7437034 | 367.4776085 | 312.9178325 | 306.7909168 | 288.5623108 | 294.6954746 | 295.5307256 | 294.4830492 | 283.9051276 | 268.4089849 | 193.4873284 | 147.9955404 | 143.3940461 | 83.85095808 | 40.99134139 | 14.44491556 | 11076.60685 |
| Eritrea | 5136.501938 | 3198.908572 | 2062.564867 | 1887.572905 | 2286.740628 | 2366.548988 | 2074.873416 | 1869.204965 | 1844.849083 | 1482.051763 | 992.8303296 | 630.254121 | 433.8376369 | 303.1405703 | 202.7459885 | 77.71052181 | 19.14601909 | 64405.72782 |
| Estonia | 268.7272218 | 223.5504067 | 344.4326279 | 488.0497992 | 565.957524 | 657.1833855 | 789.1638462 | 861.6191144 | 1091.490857 | 1199.242633 | 1136.545721 | 937.6353788 | 743.9132006 | 1128.048444 | 535.438544 | 368.8317983 | 181.0295941 | 12223.48072 |
| Ethiopia | 60781.83531 | 33603.10649 | 22071.80361 | 19271.65955 | 21883.60544 | 20854.38432 | 16992.7403 | 15511.11766 | 15212.34767 | 13560.53078 | 11304.23924 | 9340.667703 | 6907.496021 | 6799.727994 | 4122.470688 | 1922.97964 | 617.4516493 | 838230.6464 |
| Micronesia (Federated States of) | 54.9459641 | 42.27693406 | 39.17146167 | 37.01389725 | 37.67206014 | 40.22830011 | 41.5081678 | 43.22887841 | 48.28982421 | 52.33344769 | 38.89494811 | 27.39534736 | 14.39103565 | 18.87885953 | 10.22122895 | 6.263817715 | 2.006276412 | 869.6782386 |
| Fiji | 378.2884813 | 306.2331509 | 282.7108134 | 304.3006279 | 359.671058 | 364.0847336 | 369.3345024 | 426.4269386 | 468.5961773 | 443.0610731 | 375.5159995 | 267.1026613 | 154.8508943 | 152.6972396 | 88.74093398 | 48.01065449 | 13.01912315 | 7142.165587 |
| Finland | 1557.49481 | 1116.753876 | 1028.525273 | 959.3477486 | 1122.619142 | 1271.143483 | 1389.274766 | 1971.928008 | 2547.04814 | 3151.270687 | 3820.575318 | 4188.817342 | 2755.234696 | 5696.954724 | 2421.07176 | 1740.399064 | 1086.144705 | 40188.84066 |
| France | 25907.11642 | 15572.45045 | 12408.68945 | 12244.98405 | 14299.74337 | 15863.72581 | 18435.81002 | 20305.13566 | 24119.3321 | 26870.52784 | 30441.94609 | 29345.18315 | 25064.19972 | 133079.1552 | 44197.1127 | 48932.43172 | 29760.48525 | 525293.9652 |
| Gabon | 872.2890721 | 561.2516699 | 437.2740938 | 397.7304566 | 432.7365515 | 482.8929531 | 540.699385 | 566.7440759 | 589.8933409 | 537.2388829 | 460.2279234 | 304.0321251 | 228.8536964 | 267.9623069 | 148.5972688 | 82.00672321 | 29.34448543 | 12983.57528 |
| Georgia | 734.5929698 | 636.2910582 | 726.936825 | 820.0111382 | 899.2177385 | 1251.234919 | 1648.876938 | 1812.247746 | 2334.030137 | 2688.817427 | 2832.479555 | 2258.103479 | 2010.995092 | 3749.303777 | 1914.570726 | 1064.942404 | 587.0197556 | 30204.34263 |
| Germany | 20204.33328 | 15958.23715 | 14342.38153 | 14405.09203 | 15097.33082 | 22225.82178 | 32752.08049 | 44400.87048 | 48036.69894 | 53898.0076 | 57771.00464 | 48864.65102 | 64345.05925 | 148265.9813 | 79181.69017 | 43985.91408 | 19282.10767 | 693342.9643 |
| Ghana | 13551.05992 | 10599.52026 | 7895.742338 | 7372.630458 | 8236.313717 | 8868.94439 | 8210.996103 | 7150.551689 | 6961.115215 | 6009.148188 | 4164.668635 | 3234.806189 | 2415.467868 | 2470.415251 | 1472.549912 | 709.2585348 | 230.2795757 | 192385.8676 |
| Greece | 2833.062047 | 1911.430827 | 1615.379843 | 1689.739458 | 2325.877146 | 3055.563071 | 3593.9048 | 3842.404139 | 4281.564586 | 5065.859504 | 6124.491283 | 8910.973806 | 9296.199955 | 23113.99427 | 11380.19032 | 7915.552965 | 3075.987952 | 90533.54929 |
| Greenland | 32.66741258 | 32.87875233 | 29.68638495 | 27.47891747 | 30.12836344 | 32.23006023 | 48.86534555 | 101.9693695 | 117.4746953 | 115.9115926 | 89.05875034 | 65.21749072 | 46.92372069 | 38.71755191 | 23.62957068 | 11.33421937 | 3.197022617 | 1049.35704 |
| Grenada | 88.46900349 | 74.25383065 | 51.13344516 | 32.79260724 | 26.66562759 | 33.3822613 | 50.17942103 | 61.05046907 | 57.0511493 | 47.60099755 | 42.42724992 | 42.87173665 | 31.88387973 | 29.96104017 | 14.82298826 | 8.243304826 | 4.869726906 | 1241.687423 |
| Guam | 76.10905193 | 68.41366966 | 59.81839867 | 55.50334434 | 63.79503717 | 73.61858585 | 90.60767977 | 107.8664905 | 120.3671291 | 126.0835037 | 129.3395413 | 115.134546 | 69.19141572 | 115.8827945 | 53.49045877 | 33.76262976 | 19.87535942 | 1875.161498 |
| Guatemala | 10471.36762 | 6561.519599 | 4709.887118 | 3366.425449 | 3150.96527 | 3408.263529 | 3848.121383 | 4190.300082 | 4835.399495 | 5379.035682 | 5545.117327 | 5847.658289 | 4987.951713 | 7758.359421 | 4717.113879 | 2295.828588 | 669.3651602 | 154762.1211 |
| Guinea | 7544.525934 | 5444.485556 | 3356.241939 | 2881.660562 | 3206.413063 | 3284.425792 | 3029.723622 | 2793.073545 | 2993.859243 | 2629.04999 | 1811.152003 | 1405.180607 | 1055.70067 | 1422.661717 | 717.4531666 | 458.9160752 | 196.3884705 | 106404.0826 |
| Guinea-Bissau | 1088.834714 | 882.9397508 | 605.1889885 | 539.4784982 | 580.8010387 | 570.9719871 | 474.8039488 | 406.6200173 | 411.4971937 | 333.2145254 | 212.6915326 | 155.950268 | 104.0793805 | 103.1822952 | 60.58223151 | 29.97575948 | 10.0823775 | 14055.65484 |
| Guyana | 744.297683 | 608.1902687 | 361.5441009 | 214.060716 | 202.2185606 | 238.0828093 | 277.3417319 | 294.3558894 | 294.6940579 | 261.9107379 | 226.1827251 | 162.7822327 | 103.2172368 | 108.8093231 | 57.53862072 | 30.75913344 | 15.96963184 | 8954.582153 |
| Haiti | 12796.85372 | 8866.605413 | 5989.231333 | 4268.782591 | 3561.59738 | 3499.933884 | 3759.5178 | 3704.283511 | 3541.114283 | 3268.519534 | 2968.825457 | 2297.87092 | 1579.608987 | 1694.662157 | 968.0039827 | 501.2717749 | 181.8523616 | 174852.8634 |
| Honduras | 7119.999186 | 4447.032438 | 3026.580153 | 2152.514647 | 1916.640022 | 2524.205163 | 3265.679451 | 3478.76485 | 3876.991001 | 4085.420112 | 3996.062448 | 3799.296615 | 2700.78925 | 2747.443614 | 1627.156422 | 788.6384152 | 269.0201797 | 106303.9316 |
| Hungary | 3491.241298 | 3341.368919 | 3137.346361 | 3441.183981 | 4874.690456 | 9284.066241 | 10511.71654 | 10274.36805 | 10281.49199 | 15043.89874 | 15545.10539 | 12488.48514 | 9219.849084 | 11894.10936 | 5959.228333 | 3812.103156 | 1665.954373 | 136056.261 |
| Iceland | 191.8940085 | 130.5929789 | 111.9453796 | 92.71375087 | 107.2988637 | 110.2621118 | 120.1496454 | 139.5678513 | 180.0548626 | 221.2436108 | 259.6674916 | 266.2582405 | 197.6182572 | 411.6062912 | 173.2850652 | 137.3765429 | 71.81191147 | 3481.963372 |
| India | 272415.232 | 300430.1318 | 326461.2597 | 417353.8057 | 540648.873 | 751750.2296 | 982739.1282 | 1119545.28 | 1291678.558 | 1553458.58 | 1625981.716 | 1347332.121 | 926597.9423 | 860935.1707 | 542593.0034 | 233623.5192 | 73404.29727 | 13942845.7 |
| Indonesia | 130210.3003 | 104655.5912 | 66423.25099 | 52729.6669 | 61601.91128 | 82149.89346 | 105502.337 | 119123.0485 | 126119.6282 | 123899.3834 | 108709.0316 | 81455.77339 | 54218.64821 | 59814.12962 | 35315.96578 | 17753.83021 | 5576.760331 | 1963623.837 |
| Iran (Islamic Republic of) | 32295.53781 | 26553.88514 | 30552.597 | 40540.34141 | 44461.84977 | 41538.6531 | 42107.99633 | 42524.96607 | 40579.78096 | 38125.78124 | 29491.54888 | 24646.2315 | 20883.17115 | 38071.12223 | 19417.80417 | 13043.71756 | 4601.703309 | 731231.2713 |
| Iraq | 25636.14252 | 20032.68602 | 18100.30412 | 15651.69589 | 13652.29152 | 13998.94988 | 14369.90406 | 12916.51622 | 12784.3212 | 11732.35949 | 9460.181119 | 7300.555414 | 4264.087639 | 4241.453651 | 2240.137402 | 1257.18213 | 565.6483362 | 349360.0725 |
| Ireland | 2054.941111 | 1182.027988 | 920.4692815 | 963.1745431 | 1290.540166 | 1479.570517 | 1779.627443 | 1974.563974 | 2220.629459 | 3360.606934 | 4245.021633 | 4325.821029 | 3033.112164 | 4648.51094 | 2353.871138 | 1452.590866 | 629.9405918 | 44874.80832 |
| Israel | 3802.074029 | 2307.710004 | 1724.998946 | 1552.527409 | 1712.622426 | 1986.071515 | 2092.870861 | 1939.105591 | 2130.982389 | 2524.079608 | 3123.778441 | 3281.575092 | 2321.000031 | 7292.161971 | 3048.583057 | 2367.929681 | 1353.770682 | 60919.11828 |
| Italy | 18855.07423 | 10041.31101 | 8939.680784 | 8974.695923 | 11164.93513 | 14611.97443 | 17407.62622 | 22017.35569 | 26563.43864 | 30781.03306 | 39121.06162 | 50320.36527 | 53753.10511 | 130416.8509 | 58975.09338 | 43922.86727 | 21132.23596 | 515349.1459 |
| Jamaica | 2212.497311 | 1726.079449 | 1181.017971 | 797.4127571 | 687.9860382 | 793.8849487 | 1068.085787 | 1152.044383 | 1148.947482 | 1039.243529 | 900.3752445 | 876.7448208 | 586.2168079 | 1037.380574 | 389.312023 | 297.9502696 | 204.0016231 | 29547.87363 |
| Japan | 36026.06929 | 30715.29476 | 25703.30519 | 26673.39322 | 32557.98227 | 40500.65801 | 50199.64414 | 55714.00171 | 74669.93905 | 110059.2965 | 185030.3613 | 257181.1315 | 249338.069 | 487023.8625 | 200186.5946 | 153597.2726 | 93326.36 | 1862023.32 |
| Jordan | 7630.794797 | 5564.4336 | 5093.306959 | 4570.700332 | 4810.306093 | 4817.148619 | 5396.091859 | 5817.660023 | 5853.521418 | 5314.315184 | 4485.190361 | 4278.492557 | 2950.129715 | 2287.667964 | 1446.224031 | 607.2060447 | 187.5562707 | 118945.3602 |
| Kazakhstan | 3798.339777 | 3348.473663 | 4277.013927 | 5109.756033 | 5173.255137 | 7532.498443 | 9677.415015 | 9795.640093 | 11094.26339 | 12348.02183 | 11380.79517 | 6339.90045 | 4194.053938 | 4334.093682 | 2711.172475 | 990.2434356 | 541.9985382 | 130951.6144 |
| Kenya | 28538.19293 | 16953.00702 | 11934.51937 | 11373.21717 | 12773.93422 | 12839.09449 | 12037.75704 | 11424.12066 | 11329.446 | 9742.774629 | 7557.758572 | 6089.515254 | 4252.840855 | 3952.219713 | 2411.502606 | 1100.922222 | 350.5306415 | 374627.7963 |
| Kiribati | 78.79217342 | 75.07331267 | 80.83469094 | 68.92216571 | 57.70924547 | 49.32314025 | 41.59717109 | 44.08431879 | 47.15472429 | 48.94060711 | 38.86572302 | 25.96928167 | 15.49891662 | 11.17793527 | 7.112229952 | 3.037926225 | 0.86876239 | 1111.065131 |
| Kuwait | 1533.082575 | 1568.126779 | 2160.086219 | 3153.846716 | 3293.13364 | 3191.466612 | 2673.714936 | 2379.022455 | 2036.434814 | 1738.626242 | 1099.924015 | 1048.752766 | 609.5623138 | 877.9752795 | 395.5647217 | 302.0403733 | 134.2364273 | 39402.4629 |
| Kyrgyzstan | 2151.574534 | 1964.251785 | 2069.513504 | 2091.914498 | 1898.125839 | 2359.008678 | 2957.314987 | 3079.121383 | 3277.460009 | 3059.283973 | 2365.577832 | 1258.188912 | 1029.591496 | 1528.073399 | 843.9538688 | 428.4401388 | 213.3239251 | 49027.1791 |
| Lao People's Democratic Republic | 2524.806824 | 2095.661915 | 1556.425363 | 1282.375608 | 1241.948258 | 1811.539838 | 2323.203055 | 2272.313282 | 2236.82738 | 2167.324302 | 1992.366683 | 1525.22467 | 1094.62585 | 1353.409335 | 743.3268164 | 415.8273506 | 153.8035223 | 41701.79704 |
| Latvia | 489.4952742 | 382.5436471 | 601.6057399 | 828.2603925 | 940.4035542 | 1098.170459 | 1346.293652 | 1561.871686 | 2003.266568 | 1942.336885 | 1590.167797 | 1272.01349 | 1123.39401 | 1624.011698 | 782.2044286 | 519.839927 | 261.2029071 | 20230.60757 |
| Lebanon | 2215.895799 | 1860.462741 | 2413.274808 | 2644.237976 | 2458.104112 | 2612.183776 | 3124.531775 | 3082.08377 | 3362.626387 | 3170.609395 | 3343.122686 | 3395.579128 | 2744.554651 | 4301.079644 | 2251.101716 | 1419.811038 | 521.2685329 | 59704.42749 |
| Lesotho | 678.1919168 | 588.6162027 | 615.0655487 | 669.5070939 | 731.6526471 | 855.7417815 | 871.2695759 | 838.0833228 | 802.8663042 | 824.7934878 | 846.451051 | 654.7855465 | 377.9739858 | 342.4827158 | 205.3785891 | 99.22251689 | 31.03522536 | 12718.05758 |
| Liberia | 2249.261298 | 1529.211671 | 1044.250534 | 958.2276567 | 1166.394855 | 1283.795985 | 1244.213438 | 925.9549541 | 894.4976838 | 683.9431544 | 462.7084313 | 343.3606375 | 243.4522469 | 316.46825 | 165.1023521 | 99.95268703 | 40.77752548 | 31337.53266 |
| Libya | 3520.566881 | 2864.036083 | 2824.002057 | 3153.462831 | 3521.311949 | 4496.201209 | 4758.165495 | 3972.865495 | 3371.812446 | 2901.075063 | 2375.951962 | 2093.273611 | 1583.983046 | 2533.472879 | 1193.438352 | 841.7010506 | 378.5356366 | 61082.66847 |
| Lithuania | 694.3876827 | 641.0664995 | 797.8357321 | 907.3527164 | 999.5515148 | 1274.396329 | 1703.953793 | 2082.411383 | 2660.148362 | 2485.737439 | 2082.683613 | 1620.618594 | 1352.220496 | 2260.90409 | 1058.537963 | 744.2700699 | 371.3411313 | 25740.85256 |
| Luxembourg | 205.5018604 | 150.0381171 | 142.2976238 | 143.8195958 | 162.948747 | 195.8571779 | 246.4348519 | 305.1463761 | 333.2800702 | 385.2559696 | 434.1582089 | 435.355127 | 360.8731323 | 708.1640157 | 325.4324073 | 235.6463797 | 111.1709496 | 5247.856379 |
| North Macedonia | 1023.496287 | 1058.010413 | 960.3855457 | 1058.76428 | 1345.223776 | 1540.905063 | 1805.335548 | 2174.620428 | 2527.377591 | 2800.702249 | 2836.747589 | 2211.888846 | 1476.5524 | 1189.596059 | 813.6956612 | 318.8318818 | 53.71579108 | 27811.37282 |
| Madagascar | 30107.28945 | 17838.00847 | 10634.95475 | 8109.240059 | 8635.835206 | 9020.286162 | 8644.855302 | 8409.332497 | 8666.15201 | 7296.80919 | 4924.397247 | 3526.499896 | 2189.454899 | 2116.544887 | 1225.486854 | 632.5886092 | 210.906481 | 325782.3601 |
| Malawi | 14226.03518 | 7576.036982 | 4615.886184 | 4186.265551 | 4687.82463 | 4425.150527 | 3857.502075 | 3347.047714 | 3340.107679 | 3219.739108 | 2759.259317 | 2206.24287 | 1353.627639 | 1213.252415 | 691.132504 | 349.6178128 | 135.6299886 | 165644.2614 |
| Malaysia | 10845.5411 | 9490.829758 | 7867.381117 | 6987.02397 | 6569.565963 | 7458.116084 | 8821.859637 | 9984.09476 | 11679.60695 | 13047.904 | 12973.26737 | 11425.7062 | 6831.486943 | 8269.058022 | 4505.115361 | 2424.783561 | 1150.604014 | 205263.4714 |
| Maldives | 119.7693234 | 128.9985515 | 146.6598743 | 152.518925 | 154.6356781 | 187.0546826 | 232.2905708 | 244.2425611 | 257.2048187 | 266.4504665 | 209.2898025 | 179.0160399 | 153.0722144 | 204.9372048 | 114.7991086 | 64.00577695 | 21.0235703 | 3756.82363 |
| Mali | 10390.65172 | 7674.992129 | 4601.809031 | 3747.075735 | 4059.741865 | 5112.657682 | 5429.745901 | 5001.945908 | 4876.423189 | 4091.927367 | 2876.50759 | 2214.493627 | 1421.898586 | 1541.755174 | 835.0483885 | 475.0196872 | 185.7718287 | 146244.891 |
| Malta | 133.1472569 | 103.4142766 | 97.69024113 | 97.50184849 | 115.388807 | 126.6938485 | 119.8822779 | 132.2244915 | 209.3617728 | 300.972363 | 383.3758757 | 477.6167047 | 289.8586573 | 586.2252645 | 271.066247 | 185.9717704 | 91.34178395 | 3828.872914 |
| Marshall Islands | 26.93870293 | 21.27449439 | 21.86655403 | 24.0362648 | 27.02350811 | 25.26168534 | 21.85026299 | 21.77550274 | 22.61308678 | 23.26010317 | 21.98728283 | 14.98297587 | 7.24467545 | 6.108640546 | 3.698905935 | 1.72062634 | 0.560972142 | 474.9548228 |
| Mauritania | 2814.67265 | 1944.147419 | 1188.952406 | 1053.662811 | 1250.419317 | 1263.620568 | 1126.66467 | 1196.778618 | 1428.143185 | 1212.880162 | 747.2997664 | 560.267031 | 457.9753664 | 581.6271092 | 317.5421358 | 186.7141814 | 58.3838108 | 38186.8731 |
| Mauritius | 390.2899982 | 324.3144084 | 259.2405904 | 206.4390219 | 272.9555572 | 426.8333886 | 528.1448841 | 785.7362868 | 1158.218036 | 1331.767395 | 1347.958617 | 1211.83944 | 784.5931331 | 1142.109268 | 519.9165307 | 373.8089643 | 181.7572375 | 12231.38678 |
| Mexico | 55830.17821 | 35394.5027 | 26724.59539 | 23017.59417 | 25100.42104 | 33989.30146 | 49227.73216 | 61663.02925 | 73583.35275 | 81851.67454 | 81358.61459 | 88589.763 | 80472.03853 | 114988.8349 | 60988.57309 | 35323.99042 | 14335.30833 | 1289300.789 |
| Republic of Moldova | 1002.669544 | 864.8188547 | 1205.205196 | 1796.669876 | 2069.893649 | 2347.528911 | 2499.309997 | 2703.267245 | 3540.79049 | 3461.226957 | 2940.309664 | 1442.466731 | 1201.639448 | 1248.72076 | 728.8454532 | 312.7179314 | 151.7433523 | 34111.59413 |
| Mongolia | 787.4047895 | 713.518913 | 876.2798298 | 1067.971251 | 1074.040613 | 1190.592569 | 1277.644255 | 1482.897752 | 1680.550454 | 1360.903741 | 791.1669746 | 561.6993007 | 379.4096873 | 469.3464592 | 274.7769064 | 163.8826157 | 28.6485694 | 21516.52032 |
| Montenegro | 301.5488131 | 267.3602292 | 216.17948 | 247.3393547 | 334.9973186 | 386.9738179 | 426.9392781 | 493.2892337 | 585.012237 | 596.9641441 | 525.4032517 | 345.8775066 | 251.031121 | 317.2799387 | 188.3544588 | 92.40563809 | 30.19840209 | 6377.072943 |
| Morocco | 16413.33095 | 12939.54737 | 12470.89546 | 12683.5421 | 13484.37851 | 15110.22066 | 16800.21464 | 17713.71629 | 19465.38038 | 18959.54458 | 15466.79768 | 11533.31789 | 8172.805868 | 10362.92667 | 5550.505124 | 3153.918272 | 1311.095216 | 296905.6702 |
| Mozambique | 23189.35899 | 13294.97106 | 7683.903248 | 6260.96561 | 7893.17715 | 7869.12644 | 6681.860982 | 5860.060372 | 5939.023981 | 5200.869611 | 3904.587277 | 2916.355653 | 1859.681643 | 1724.056743 | 1054.842124 | 465.6216491 | 160.2214191 | 299873.3439 |
| Myanmar | 16678.33331 | 13290.43241 | 9296.77872 | 7886.742466 | 8542.154934 | 17577.22495 | 26225.07787 | 28625.64493 | 31308.58076 | 40636.31924 | 40974.56644 | 31440.69407 | 19724.85307 | 24316.05078 | 13174.95073 | 7507.492592 | 2924.119753 | 411483.8676 |
| Namibia | 948.4767575 | 726.3080705 | 677.5753141 | 695.3886232 | 755.8343472 | 863.1895974 | 971.0033609 | 942.5392128 | 865.0757634 | 846.0019057 | 861.5629234 | 779.3350383 | 570.7326125 | 581.425963 | 324.356818 | 172.1763893 | 65.4946233 | 16824.82272 |
| Nepal | 4971.303678 | 5201.204286 | 4709.264415 | 4971.539257 | 6114.658888 | 10610.70049 | 15438.53903 | 18227.9561 | 21033.05132 | 26343.94355 | 28102.47178 | 23599.59848 | 15639.51118 | 13884.28826 | 8626.684084 | 3741.940812 | 1187.634891 | 218389.3293 |
| Netherlands | 3937.904854 | 2962.727605 | 2722.289954 | 2583.476195 | 2770.674127 | 4431.245061 | 7427.883623 | 8325.651255 | 8899.229359 | 12989.53853 | 16950.32025 | 18906.65017 | 13168.49315 | 22587.1226 | 10889.09831 | 7262.711571 | 3319.543328 | 145207.0435 |
| New Zealand | 1446.472792 | 1054.306995 | 927.4757255 | 874.0186209 | 983.8691864 | 1377.057236 | 2293.746552 | 2894.872636 | 3762.464544 | 5112.248363 | 6631.877083 | 7509.01949 | 6198.537023 | 8510.057216 | 4317.995948 | 2613.261517 | 1197.005826 | 65884.44671 |
| Nicaragua | 3926.817697 | 2575.271029 | 2043.646031 | 1635.296593 | 1475.76392 | 1637.331883 | 1980.96442 | 2197.389108 | 2336.159909 | 2228.509655 | 1987.283604 | 1983.36895 | 1672.84979 | 2012.307848 | 1160.904273 | 594.607737 | 203.5739901 | 62913.94675 |
| Niger | 11658.41657 | 7554.677472 | 4402.435719 | 3576.316487 | 4114.540968 | 4132.080041 | 3676.278615 | 3951.936365 | 4541.932442 | 3781.16384 | 2412.007822 | 1895.287779 | 1384.950044 | 1166.029336 | 694.0192973 | 317.5813798 | 125.978545 | 180133.1172 |
| Nigeria | 130693.5344 | 91756.46911 | 57407.59204 | 49885.36689 | 57063.01114 | 54148.74629 | 45726.89469 | 47476.80494 | 51769.12107 | 38525.45464 | 22601.80716 | 15975.3001 | 16523.3604 | 18405.63942 | 10361.15638 | 5357.741939 | 2074.592616 | 1864933.306 |
| Democratic People's Republic of Korea | 7571.66561 | 6654.785308 | 5961.424745 | 5648.700593 | 7055.937931 | 11680.6652 | 19972.78856 | 22046.95637 | 18735.22243 | 25935.95935 | 24435.39319 | 24837.98782 | 18546.03065 | 19549.1661 | 11812.6896 | 5337.097899 | 1893.845063 | 266642.4482 |
| Northern Mariana Islands | 23.96624489 | 16.02096731 | 13.323441 | 8.113924145 | 10.31815163 | 21.93925943 | 29.22383676 | 45.99389434 | 51.50831909 | 48.4896711 | 44.50992251 | 19.13114115 | 16.06757042 | 15.40547318 | 7.85577966 | 5.268046496 | 1.739395099 | 457.3918188 |
| Norway | 2382.712094 | 1788.844511 | 1678.068085 | 1634.009801 | 1780.026466 | 2187.48328 | 2872.555789 | 3172.541992 | 3366.883668 | 4840.150411 | 5920.477026 | 6325.953025 | 4064.519998 | 6697.182033 | 2937.86178 | 2135.624703 | 1161.367655 | 61767.69715 |
| Oman | 1597.263337 | 1997.311015 | 3152.484574 | 3706.744718 | 3073.944298 | 2289.64009 | 1707.995851 | 1364.938997 | 1277.605741 | 1018.385432 | 746.9229115 | 644.4633295 | 351.2657152 | 249.7919553 | 151.8621961 | 62.31899152 | 30.76273437 | 36962.00314 |
| Pakistan | 51381.87934 | 50042.60455 | 47474.00349 | 48991.51912 | 53110.95388 | 62960.05396 | 73484.52456 | 75399.76783 | 75256.24919 | 73624.31689 | 68089.42603 | 56055.55435 | 40614.71837 | 42970.48563 | 25677.21081 | 12121.02774 | 4135.344458 | 1133046.381 |
| Palestine | 2939.447796 | 2228.338584 | 1956.103909 | 1615.355946 | 1510.410909 | 1654.16017 | 1918.881301 | 2299.765659 | 2534.621382 | 2439.879926 | 2088.956259 | 1685.21557 | 988.8635304 | 862.6593012 | 472.2062368 | 269.3375587 | 94.03882584 | 48647.53202 |
| Panama | 2562.414372 | 1700.335406 | 1218.472668 | 980.1755796 | 933.2536092 | 1140.668601 | 1599.990004 | 2021.826097 | 2395.407275 | 2444.580732 | 2465.189466 | 2752.053705 | 2404.337846 | 4011.725186 | 1815.625005 | 1208.738017 | 671.9862505 | 51186.289 |
| Papua New Guinea | 5527.532633 | 5067.188188 | 5382.559644 | 5199.470285 | 4845.262716 | 5940.767151 | 6544.45594 | 6010.338863 | 5254.232457 | 5602.475709 | 4873.841277 | 3854.74627 | 2143.744664 | 1916.913318 | 1148.001096 | 515.6367307 | 198.7739692 | 114573.9849 |
| Paraguay | 4657.672225 | 2996.980938 | 2203.026898 | 1663.428061 | 1431.673714 | 1463.580221 | 1722.080797 | 1958.79057 | 2164.741774 | 2239.534597 | 2200.078273 | 1900.706373 | 1771.449609 | 2899.261688 | 1375.791999 | 838.1465463 | 435.7307372 | 79235.5177 |
| Peru | 13502.27522 | 8554.749726 | 7674.995482 | 7725.187393 | 9602.351911 | 12432.2125 | 16187.76699 | 21094.21302 | 27688.46557 | 33182.45893 | 36466.07509 | 36384.30982 | 32525.22918 | 53054.69586 | 25022.23358 | 17007.36541 | 7930.713156 | 526071.2557 |
| Philippines | 60940.51171 | 50236.96093 | 36361.85551 | 27920.15043 | 27788.62646 | 31740.7056 | 35285.59647 | 36751.17929 | 38572.20208 | 39967.31007 | 34699.87609 | 24350.22014 | 15683.68404 | 21242.99602 | 11570.96169 | 6342.541299 | 2641.350869 | 1093088.397 |
| Poland | 17477.42146 | 17263.05358 | 17215.42274 | 21167.69943 | 28192.59905 | 30900.37006 | 31745.89501 | 35082.21359 | 46978.55445 | 60955.2578 | 57877.93888 | 44000.60388 | 27745.24816 | 45027.2637 | 22587.11938 | 14727.78319 | 6158.761245 | 566302.4182 |
| Portugal | 4231.597851 | 2566.601863 | 2078.884771 | 2070.775386 | 2858.15192 | 3988.075384 | 4221.574447 | 4536.953641 | 5591.220455 | 6703.322476 | 8560.424618 | 11086.011 | 10740.04799 | 23239.07822 | 11309.18957 | 7652.10568 | 3327.116391 | 109546.9445 |
| Puerto Rico | 2655.024554 | 2019.584207 | 1396.091815 | 1046.265598 | 1073.992718 | 1461.652285 | 2026.455154 | 2482.103934 | 2920.867902 | 3316.143804 | 3693.486407 | 4552.85927 | 3880.358177 | 7373.952815 | 3253.165875 | 2277.130659 | 1221.129466 | 54199.68381 |
| Qatar | 625.0557759 | 1159.638478 | 1992.035316 | 2313.638026 | 1969.639068 | 1614.380895 | 1355.230711 | 1055.872931 | 961.1874883 | 693.0950968 | 418.278059 | 237.5215416 | 93.78316212 | 46.50492648 | 33.35747509 | 10.74380467 | 2.167872643 | 19538.76594 |
| Romania | 8590.183841 | 7239.462238 | 5925.800691 | 7830.66082 | 9830.209138 | 16117.24482 | 19797.99255 | 21684.10378 | 19769.68284 | 28488.73079 | 29230.86108 | 23068.50251 | 17312.40938 | 27036.527 | 14228.56584 | 8540.173051 | 3498.691287 | 274949.2872 |
| Russian Federation | 36273.07222 | 28215.83737 | 39006.18646 | 65828.89273 | 77823.43224 | 85668.60104 | 92966.84565 | 91963.0942 | 117809.7373 | 118819.5419 | 98921.56179 | 60123.36594 | 33669.26323 | 70645.97275 | 34753.31965 | 20403.38421 | 12775.15752 | 1303724.338 |
| Rwanda | 12792.04954 | 8081.576921 | 4958.056496 | 5171.219037 | 6077.226151 | 5294.672573 | 4069.414125 | 4380.14519 | 5489.346388 | 4962.609258 | 3459.105878 | 2168.648757 | 1439.660786 | 1395.81075 | 853.8828889 | 390.9595934 | 125.8288447 | 174929.8548 |
| Saint Lucia | 141.2487405 | 109.2631934 | 76.8913674 | 55.20064429 | 57.97554923 | 79.84655959 | 111.2799601 | 119.661731 | 114.180094 | 102.0259794 | 85.71449132 | 91.75912547 | 66.56597015 | 107.8975647 | 48.92075206 | 35.52354452 | 18.7227199 | 2067.790053 |
| Saint Vincent and the Grenadines | 84.67805483 | 59.24913938 | 38.27062407 | 27.97411677 | 28.52164673 | 33.79000765 | 42.10248356 | 47.44707145 | 49.14987116 | 46.42840129 | 38.15248233 | 35.85138687 | 26.98549039 | 34.01724571 | 18.96519649 | 10.34303969 | 3.740443509 | 1164.713413 |
| Samoa | 109.8745924 | 71.34288536 | 64.4974134 | 62.93931883 | 60.50522089 | 63.65843668 | 74.27758132 | 79.43119338 | 83.43923758 | 82.50028859 | 73.24116843 | 58.79750326 | 48.53858909 | 75.90542696 | 39.18307155 | 24.20077583 | 10.08914221 | 1756.306051 |
| Sao Tome and Principe | 142.4358306 | 103.4968051 | 65.76433388 | 62.87138541 | 78.14725483 | 95.61580067 | 95.00679631 | 90.07611925 | 101.2498398 | 89.81706091 | 63.61337117 | 45.02283335 | 35.71238064 | 42.70876506 | 25.7717432 | 12.20653703 | 3.890968777 | 1928.420072 |
| Saudi Arabia | 13250.76064 | 12551.30413 | 14673.31044 | 18725.59607 | 22466.77067 | 24842.76108 | 25147.93949 | 22709.14566 | 20679.35004 | 18190.99101 | 14534.66551 | 10746.60161 | 5500.695577 | 5155.073882 | 2707.323206 | 1525.479919 | 762.4336442 | 303531.636 |
| Senegal | 7111.135424 | 5023.35565 | 3412.93681 | 3089.925774 | 3469.369825 | 3633.39947 | 3446.789701 | 3440.700057 | 3714.696412 | 3158.392054 | 2187.468709 | 1756.81887 | 1293.246793 | 1611.244505 | 880.0758512 | 494.7447949 | 188.2854461 | 98125.05892 |
| Serbia | 3271.349323 | 2896.111731 | 2569.430713 | 2851.116884 | 3768.331462 | 5095.837965 | 6344.670278 | 7004.385938 | 7538.817663 | 9746.730029 | 11145.43887 | 8913.362219 | 8103.993336 | 9988.091362 | 6694.731438 | 2698.276531 | 528.1092086 | 101429.9803 |
| Seychelles | 27.51315439 | 23.75671767 | 19.64927494 | 17.76074038 | 19.19529033 | 29.68577124 | 39.86964596 | 42.06568164 | 46.6902718 | 47.69487499 | 39.67689072 | 34.14836047 | 26.4470371 | 39.79074469 | 18.97747425 | 13.34056142 | 5.834321886 | 657.9244455 |
| Sierra Leone | 4574.44787 | 3423.476259 | 2233.367323 | 1925.137938 | 2046.951379 | 2131.858978 | 1888.369573 | 1701.40726 | 1706.563912 | 1417.352453 | 984.8425037 | 804.5531126 | 656.5371821 | 722.0418371 | 418.2521763 | 206.5085534 | 77.71440212 | 60061.18491 |
| Singapore | 1503.53023 | 1459.544303 | 2193.607812 | 2220.652658 | 3292.167021 | 2874.615075 | 3079.503577 | 3652.415457 | 4607.901558 | 5445.06883 | 5676.191362 | 5256.819632 | 3166.668233 | 3480.222783 | 1660.38187 | 1065.896075 | 547.9569343 | 56974.96032 |
| Slovakia | 1723.04186 | 1723.366636 | 1825.14284 | 2264.578839 | 3109.690501 | 4011.594913 | 4257.306147 | 4546.12976 | 5500.210719 | 6319.532963 | 6145.600528 | 4703.616783 | 3064.712936 | 3724.929992 | 1865.574835 | 1200.313627 | 505.1313383 | 60338.0555 |
| Slovenia | 799.3533886 | 752.0791751 | 693.7777254 | 894.4095684 | 1288.808698 | 1579.420015 | 1756.353278 | 2188.952284 | 2567.938638 | 2909.40618 | 3075.816778 | 2636.559765 | 2534.583342 | 4505.884918 | 2077.710209 | 1503.101516 | 716.6495824 | 31783.23858 |
| Solomon Islands | 318.6706143 | 259.4383312 | 272.241776 | 260.5429449 | 231.9031536 | 231.4963162 | 226.9166003 | 207.8289891 | 197.8717823 | 187.1198074 | 174.0933929 | 132.5405031 | 78.48190873 | 62.82892814 | 42.75444977 | 15.90155857 | 3.599271972 | 5679.411172 |
| Somalia | 16261.58686 | 9558.780602 | 5487.717258 | 4341.310059 | 5761.622215 | 6286.890154 | 4599.016429 | 3143.63735 | 3523.54027 | 3563.963701 | 2766.461583 | 1803.105993 | 978.327758 | 674.7854191 | 443.4674359 | 174.0767454 | 48.06017185 | 205915.3302 |
| South Africa | 17766.6492 | 14268.29827 | 16489.82719 | 19456.76344 | 21090.84843 | 24786.62137 | 31825.04494 | 34139.0742 | 35122.44238 | 36244.05767 | 33802.8254 | 28230.97451 | 20097.72764 | 24817.90508 | 13487.61364 | 7870.29763 | 2863.461377 | 570530.9884 |
| Republic of Korea | 13539.78581 | 13548.97475 | 11200.9743 | 8951.957619 | 11303.65761 | 12080.8329 | 15794.5688 | 17893.90556 | 24078.6164 | 32986.12509 | 38191.82178 | 47676.47051 | 46207.99163 | 77656.95798 | 41733.11039 | 24563.33108 | 8934.850867 | 441955.2599 |
| South Sudan | 6762.5188 | 3254.234166 | 1583.784145 | 1753.000156 | 2435.647562 | 2621.754078 | 2439.142931 | 2431.426537 | 2353.904718 | 1921.127838 | 1358.595171 | 1113.110925 | 745.8216623 | 751.5816091 | 428.1571679 | 211.6070997 | 86.00057981 | 91081.60306 |
| Spain | 12758.95531 | 8149.546956 | 7277.692181 | 7569.758609 | 10278.20826 | 15669.16247 | 18715.73229 | 19772.49243 | 20697.11744 | 29024.3231 | 34872.5628 | 40219.8608 | 37352.45964 | 95624.18975 | 42029.30326 | 33241.69183 | 15360.10123 | 419332.7569 |
| Sri Lanka | 6462.032612 | 4797.274889 | 3603.419685 | 3264.795714 | 4051.237586 | 6258.938134 | 9105.063487 | 11474.55709 | 14059.35872 | 18159.96657 | 21928.15489 | 18560.07364 | 11584.35406 | 15485.81252 | 7978.377244 | 5036.538189 | 1961.166309 | 193526.2731 |
| Sudan | 27867.49401 | 20467.15316 | 17458.94084 | 15745.01065 | 14774.07811 | 14566.08758 | 14827.94253 | 13662.25307 | 11642.2922 | 10517.92199 | 9228.795884 | 8062.237082 | 5674.05091 | 6801.069659 | 3597.907071 | 1994.604062 | 870.7975557 | 383234.5109 |
| Suriname | 425.9340343 | 292.3112319 | 189.6793484 | 148.5073709 | 139.8193241 | 157.1979076 | 211.7549849 | 257.8168801 | 261.5576251 | 223.4677995 | 183.1421479 | 174.3083413 | 139.9968567 | 173.263166 | 91.01534307 | 49.35527616 | 22.35096791 | 6301.45584 |
| Eswatini | 656.5707812 | 543.52936 | 571.6647403 | 589.3139436 | 568.0768094 | 574.8290364 | 559.506602 | 484.8801746 | 426.3674049 | 440.0991491 | 446.9090572 | 356.1521848 | 207.2575236 | 149.5411846 | 95.51654737 | 39.36990141 | 12.02830111 | 10719.66227 |
| Sweden | 4190.721494 | 2815.683982 | 2803.551219 | 2403.138157 | 2369.869129 | 2598.668982 | 3262.910393 | 4331.581159 | 5245.118257 | 6881.468519 | 9014.770078 | 11638.64404 | 9850.942213 | 15749.72163 | 7327.961477 | 4842.361093 | 2549.58854 | 105253.6926 |
| Switzerland | 2692.332545 | 2018.980512 | 1864.274575 | 1844.776597 | 2088.934469 | 2272.475618 | 2657.347046 | 3482.297223 | 4294.312974 | 4573.864893 | 4766.555843 | 5223.133073 | 5275.070774 | 14006.94709 | 5691.051279 | 4708.661816 | 2558.539685 | 71197.09261 |
| Syrian Arab Republic | 10851.70769 | 6322.356709 | 2674.973563 | 3105.713789 | 5146.01478 | 6290.123133 | 8016.94526 | 9224.833315 | 9680.51716 | 8552.071894 | 7331.700065 | 5687.647458 | 4090.734047 | 4154.656555 | 2497.71307 | 1351.211939 | 264.6527955 | 135424.0259 |
| Taiwan (Province of China) | 5550.617803 | 4883.691173 | 4097.14854 | 3790.397369 | 5439.329909 | 6473.007027 | 7042.582059 | 9203.842667 | 12823.68303 | 16978.96542 | 20105.68478 | 15816.77896 | 13272.36784 | 24716.00244 | 10159.04583 | 6924.205088 | 5060.61679 | 187319.349 |
| Tajikistan | 3216.692561 | 2734.384748 | 2795.699598 | 2992.58131 | 2815.58871 | 3085.238136 | 3601.936684 | 4193.17807 | 5044.226665 | 4592.787856 | 3553.452258 | 2044.703734 | 1186.942415 | 1081.536356 | 662.1721959 | 275.5354108 | 112.2096776 | 68024.76816 |
| United Republic of Tanzania | 47441.97435 | 28223.26073 | 17991.66176 | 15474.32301 | 17791.75973 | 17168.47262 | 14516.07087 | 14177.28823 | 14708.70606 | 12254.54308 | 9320.590698 | 7060.892991 | 5060.822755 | 5097.770934 | 3033.116298 | 1453.246293 | 485.4528311 | 700308.7263 |
| Thailand | 20161.65817 | 19388.17641 | 14977.14154 | 13666.06127 | 16734.07627 | 23774.57944 | 30071.85023 | 33161.76989 | 38099.74344 | 42209.29513 | 40267.04561 | 37472.01042 | 28091.24002 | 44121.854 | 21624.21458 | 13694.85106 | 6483.677423 | 517887.1947 |
| Bahamas | 284.3420607 | 206.4893638 | 141.5808191 | 103.1608931 | 104.6170437 | 125.0228588 | 162.3109919 | 181.1459245 | 187.2544547 | 177.9605273 | 143.2823403 | 121.7894918 | 89.77918969 | 98.80224695 | 51.45995999 | 27.33908653 | 13.49815579 | 3916.523381 |
| Gambia | 1216.408137 | 863.1210312 | 555.7394197 | 499.6035145 | 577.7072995 | 608.0975627 | 530.9405885 | 481.1074905 | 482.4702778 | 413.7819465 | 279.6856935 | 222.4547199 | 199.4701004 | 231.4360279 | 141.3268119 | 66.2702679 | 19.31852919 | 16134.1217 |
| Timor-Leste | 685.2557409 | 462.167496 | 265.362558 | 170.3369103 | 149.4208031 | 215.5501296 | 322.5552938 | 320.4882725 | 315.3131212 | 436.3112404 | 516.1191329 | 406.0645199 | 237.8494846 | 257.911374 | 145.8202741 | 84.00525472 | 23.29430405 | 9891.217497 |
| Togo | 4489.744232 | 3365.69914 | 2289.137249 | 2123.740948 | 2480.327831 | 2732.790755 | 2539.78274 | 2354.421362 | 2407.049229 | 1806.018875 | 1181.566624 | 817.206289 | 597.8132727 | 568.0077389 | 333.7816425 | 165.0972471 | 54.54110483 | 65740.6178 |
| Tonga | 57.04870151 | 38.3898984 | 30.55077198 | 28.52396427 | 31.27689553 | 33.3245117 | 35.18704956 | 37.25058617 | 36.16446766 | 38.5265317 | 43.04621982 | 41.47998008 | 33.82909457 | 55.90095593 | 26.50984812 | 18.05387553 | 8.450886456 | 997.1606331 |
| Trinidad and Tobago | 742.7342214 | 504.8018168 | 390.6956901 | 364.2234792 | 384.2495445 | 395.5578317 | 462.3111591 | 566.9713805 | 769.6114927 | 831.9245305 | 801.1776125 | 711.9255235 | 468.3236685 | 526.9822091 | 273.6226186 | 150.6903531 | 69.20719268 | 13508.8666 |
| Tunisia | 4618.996818 | 3779.022382 | 4099.850506 | 4591.992417 | 5368.855365 | 5823.119431 | 6056.350654 | 6897.714685 | 8127.055173 | 8509.673921 | 7857.459939 | 6924.851694 | 5194.974235 | 7330.674485 | 3942.28315 | 2360.438498 | 808.3798173 | 115871.347 |
| Turkey | 41975.5752 | 36454.12066 | 37621.17394 | 40594.29433 | 44571.9663 | 57057.7617 | 61424.68478 | 65078.58188 | 73057.6986 | 91260.22588 | 93271.95816 | 82449.28786 | 47550.5405 | 61464.82109 | 31849.87597 | 19353.84843 | 7873.490202 | 1031952.924 |
| Turkmenistan | 1510.656944 | 1294.425874 | 1404.256443 | 1482.705635 | 1494.25035 | 1591.694043 | 1732.798047 | 1848.007555 | 2072.168869 | 1753.763866 | 1226.272714 | 614.7426953 | 462.8637568 | 812.3681176 | 368.8937107 | 238.4460225 | 150.1740127 | 31106.91536 |
| Uganda | 33447.41224 | 18438.29902 | 10987.66155 | 8979.964951 | 9693.949826 | 9502.533721 | 8640.610791 | 8608.401241 | 9133.224809 | 7676.552637 | 5823.4167 | 4822.963964 | 3215.622855 | 2881.133024 | 1758.742228 | 778.9362145 | 269.7699927 | 460042.8616 |
| Ukraine | 12353.40486 | 10583.90582 | 14068.61528 | 20391.89955 | 24030.06741 | 26493.08068 | 29086.5379 | 29183.48761 | 34468.6285 | 33109.74073 | 27192.70427 | 18155.49946 | 14212.45988 | 25643.05899 | 14191.50185 | 7036.924092 | 3682.970001 | 412669.1295 |
| United Arab Emirates | 2767.731695 | 2132.65571 | 2820.23164 | 12160.93547 | 15521.80574 | 16774.04532 | 13269.73579 | 9207.683394 | 5440.672722 | 3593.354809 | 1797.572294 | 674.121951 | 297.3169451 | 244.2974411 | 154.8480357 | 69.33656852 | 17.97574169 | 104966.8384 |
| United Kingdom | 33360.84323 | 23164.64908 | 20392.3922 | 19087.2292 | 20217.17458 | 23921.9453 | 31982.63709 | 36015.65244 | 39050.44403 | 53066.59858 | 65382.46744 | 72701.6841 | 52474.52468 | 97381.84062 | 44727.48102 | 31647.46687 | 15998.52338 | 773974.8856 |
| United States of America | 244406.5711 | 187506.611 | 191054.6052 | 201904.0151 | 244233.4021 | 302236.4233 | 415672.3402 | 526763.8843 | 711145.8773 | 902633.1332 | 959304.529 | 890123.8579 | 675302.72 | 777148.8492 | 413334.5118 | 224811.7375 | 103221.3508 | 9271837.103 |
| Uruguay | 1536.228758 | 1299.735743 | 939.5122492 | 854.837296 | 999.5445105 | 1537.675637 | 1926.102277 | 2112.318097 | 2544.832588 | 3221.335394 | 3324.452895 | 2998.484038 | 2349.70488 | 4865.352328 | 1994.704766 | 1654.544918 | 896.5854226 | 40694.24218 |
| Uzbekistan | 11797.94658 | 10396.17333 | 11285.49501 | 12064.53567 | 11889.74215 | 12724.57923 | 14208.35621 | 16865.9922 | 21047.53947 | 18832.81629 | 13469.50446 | 6446.302352 | 4244.311589 | 2205.174006 | 1659.55836 | 443.7510339 | 93.63955177 | 257478.3302 |
| Vanuatu | 137.076572 | 108.6303147 | 106.6706879 | 100.1117842 | 96.56035739 | 94.5105925 | 91.39993244 | 89.4293355 | 90.5285098 | 104.1348662 | 102.9977675 | 90.31391089 | 69.20968135 | 57.98070925 | 37.72882264 | 15.73790164 | 3.867333087 | 2526.302281 |
| Venezuela (Bolivarian Republic of) | 15463.39065 | 9424.586151 | 7741.533073 | 6882.653186 | 6812.512965 | 8833.099733 | 11940.28911 | 13842.28791 | 14693.701 | 14893.16671 | 14137.3807 | 13727.341 | 10934.33479 | 16869.58031 | 7698.254861 | 4804.893942 | 2942.093113 | 286231.1046 |
| Viet nam | 27635.63764 | 24432.72875 | 22640.50628 | 19837.66563 | 20921.14569 | 31979.41943 | 45975.8869 | 51146.37044 | 58147.39326 | 65579.35908 | 56159.92318 | 39986.6315 | 30471.13201 | 49676.09342 | 26094.84454 | 16637.34601 | 5509.50509 | 739901.8849 |
| Virginia | 6554.094147 | 5140.288338 | 5284.212749 | 5830.115516 | 7298.676868 | 8965.169413 | 12304.32286 | 15600.36611 | 20511.76171 | 24747.66972 | 25563.76116 | 23823.79718 | 17440.15379 | 18511.20471 | 10249.08146 | 5359.058959 | 2252.892739 | 252410.2255 |
| Yemen | 19807.91487 | 13263.73262 | 12134.42618 | 12428.66009 | 12381.28563 | 11924.664 | 10348.19954 | 8770.893393 | 8068.171746 | 7681.824294 | 6924.087323 | 5720.322767 | 3669.352185 | 3684.530472 | 2078.64716 | 1097.158359 | 397.312055 | 287143.7606 |
| Zambia | 9458.288214 | 5534.70531 | 3828.95668 | 3333.807032 | 3601.194089 | 3773.146663 | 3615.070312 | 3271.668288 | 3003.541764 | 2557.639258 | 1987.286838 | 1783.738233 | 1232.028215 | 1048.78305 | 659.3497314 | 294.5838042 | 79.09573553 | 127134.0775 |
| Zimbabwe | 5772.00208 | 4134.073709 | 3678.931964 | 4170.678322 | 5055.972469 | 5483.23 | 5313.729951 | 4508.571274 | 4054.147428 | 3827.990812 | 3462.730773 | 2555.930491 | 1332.129039 | 1126.23923 | 648.2591021 | 317.172296 | 127.1903683 | 89091.23945 |
| Monaco | 10.77179743 | 6.727487869 | 5.598729412 | 5.494323925 | 6.792974189 | 9.511672687 | 14.4868073 | 20.38514699 | 22.78669074 | 25.75324275 | 28.00923761 | 38.74222985 | 34.02625777 | 74.58748869 | 33.48154748 | 23.13216814 | 12.41355139 | 352.5511547 |
| San Marino | 12.05383726 | 8.068137499 | 6.411490575 | 5.878947879 | 6.747911117 | 9.440789477 | 13.41552555 | 15.6290116 | 15.77332351 | 16.2105236 | 18.39815498 | 21.22366892 | 21.08490677 | 51.50662691 | 23.26936708 | 16.70940591 | 8.28553299 | 273.812958 |
| Saint Kitts and Nevis | 40.09914868 | 29.09589927 | 20.57053886 | 16.59628049 | 16.28886295 | 20.17263279 | 29.16566046 | 34.23534064 | 37.756325 | 38.24922577 | 29.59236516 | 20.05880284 | 12.91349068 | 16.59216614 | 8.676442609 | 4.619915794 | 2.312051084 | 638.2043376 |
| Cook Islands | 7.734173295 | 6.168732916 | 5.278598932 | 5.311170982 | 5.772968334 | 9.048457938 | 12.68209571 | 13.86849531 | 18.19209757 | 16.60531414 | 23.81598043 | 13.6671832 | 16.21168856 | 15.88670618 | 8.305899761 | 5.32662447 | 1.74256798 | 224.8776632 |
| Nauru | 5.697285017 | 4.339344603 | 4.194224909 | 4.171720137 | 4.089460018 | 4.794057646 | 5.063062293 | 4.379056637 | 3.850717211 | 3.686374283 | 2.300236683 | 1.336124569 | 0.701545192 | 0.529118284 | 0.327509844 | 0.142848667 | 0.048431665 | 94.81984225 |
| Niue | 0.747989055 | 0.532184289 | 0.490617741 | 0.470375593 | 0.575455323 | 0.85179039 | 1.131329819 | 1.345942001 | 1.52987819 | 1.736050661 | 1.640850438 | 1.44096691 | 1.114425047 | 1.529332118 | 0.806832278 | 0.486180572 | 0.190319506 | 20.10902904 |
| Palau | 6.550021903 | 5.724523838 | 5.856966927 | 7.256487928 | 10.46549347 | 16.02867511 | 20.22086295 | 21.77962103 | 22.81345346 | 23.48654999 | 21.99083351 | 15.89436178 | 7.508071252 | 6.649501064 | 3.659284323 | 2.187295147 | 0.677873336 | 232.181601 |
| Tokelau | 0.640617519 | 0.478307303 | 0.38346242 | 0.4747054 | 0.403792932 | 0.660462346 | 0.817912595 | 0.760115944 | 0.890467574 | 0.798017902 | 1.632697277 | 0.757796208 | 0.537378306 | 0.861128702 | 0.515425019 | 0.166624331 | 0.139514131 | 16.13354728 |
| Tuvalu | 6.005577925 | 4.673827257 | 4.231885058 | 4.025613536 | 4.008914012 | 5.049148358 | 6.134041347 | 6.590686045 | 6.739058496 | 8.171207918 | 8.805734927 | 7.014788252 | 4.571422663 | 4.569685384 | 2.593517419 | 1.363124913 | 0.508027207 | 121.2639517 |

**Supplementary Table 7. Age distribution of deaths rate for chronic obstructive pulmonary disease in different countries in 2019.**

| 2019death rate | 15 to 19 | 20 to 24 | 25 to 29 | 30 to 34 | 35 to 39 | 40 to 44 | 45 to 49 | 50 to 54 | 55 to 59 | 60 to 64 | 65 to 69 | 70 to 74 | 75 to 79 | 80 plus | 80-84 | 85-89 | 90-94 | all ages |
| --- | --- | --- | --- | --- | --- | --- | --- | --- | --- | --- | --- | --- | --- | --- | --- | --- | --- | --- |
| Afghanistan | 98.79362225 | 108.1948624 | 130.3906287 | 130.4649629 | 156.9088354 | 229.2664059 | 524.8531198 | 638.5448316 | 529.8748525 | 473.244591 | 709.1021662 | 869.2739192 | 915.6018431 | 1339.689099 | 729.6309349 | 422.6235196 | 149.1641476 | 7082.190146 |
| Albania | 0.539216204 | 1.068587498 | 1.252779577 | 1.190103377 | 1.381397692 | 1.976073155 | 3.935532385 | 8.32569989 | 17.42874541 | 40.13862478 | 63.42714357 | 108.4155677 | 152.0049862 | 411.1372422 | 191.7684939 | 132.9641814 | 62.81084804 | 814.6387069 |
| Algeria | 24.12670515 | 29.78007754 | 44.62115788 | 73.4557261 | 104.6578709 | 132.7428479 | 180.5559331 | 252.5377269 | 352.3788869 | 521.9230795 | 707.8308134 | 757.8648721 | 1129.599068 | 3165.002549 | 1309.160291 | 1478.652915 | 330.7814271 | 7527.834018 |
| American Samoa | 0.15271357 | 0.182124919 | 0.183732293 | 0.224297879 | 0.348779035 | 0.487746133 | 0.710717186 | 1.125044005 | 1.485867562 | 1.965263424 | 2.31670295 | 2.841344062 | 3.164743332 | 7.62151856 | 3.275880155 | 2.398857801 | 1.382059195 | 22.94587683 |
| Andorra | 0.013076543 | 0.022525415 | 0.030880541 | 0.04396369 | 0.080453212 | 0.152639344 | 0.309796838 | 0.622598542 | 1.114769001 | 1.762211631 | 2.596727115 | 4.095449173 | 5.374483604 | 23.18881323 | 5.872768994 | 7.656507383 | 6.163246401 | 39.42155534 |
| Angola | 38.61441123 | 49.55022171 | 44.838966 | 66.20075416 | 66.74563647 | 114.0289561 | 159.1575944 | 225.2982339 | 344.5921372 | 434.8127349 | 511.9700703 | 489.7944987 | 520.3720832 | 688.1715549 | 374.5852985 | 195.9454989 | 92.61525929 | 3933.852734 |
| Antigua and Barbuda | 0.025720709 | 0.041719613 | 0.054595621 | 0.054617755 | 0.082700696 | 0.110339641 | 0.189061593 | 0.361491315 | 0.600924038 | 0.929577798 | 1.055732691 | 1.393912956 | 1.550105054 | 4.019042223 | 1.694815009 | 1.275001461 | 0.737751233 | 10.55341317 |
| Argentina | 31.83373515 | 43.2928979 | 53.90761439 | 67.79415942 | 88.90866336 | 128.610505 | 211.9393613 | 371.6263425 | 675.6986491 | 1220.112747 | 1781.077961 | 2423.684485 | 2886.98479 | 9304.160563 | 3278.210839 | 3059.887624 | 1939.359236 | 19348.12346 |
| Armenia | 0.248173841 | 0.508999431 | 0.829590547 | 1.482452493 | 2.494427487 | 4.899300536 | 8.041650973 | 17.38716737 | 40.52642055 | 82.51055094 | 114.1827367 | 135.4028819 | 162.2366442 | 751.7549211 | 329.3414614 | 261.0028931 | 137.9585976 | 1323.02375 |
| Australia | 6.533618344 | 8.947337534 | 11.23054244 | 15.89967845 | 21.1550813 | 31.27430676 | 62.68968112 | 118.0807102 | 245.3740494 | 449.2962652 | 833.81451 | 1440.725035 | 1779.775638 | 6168.200695 | 2073.161898 | 2065.031097 | 1411.10012 | 11202.85953 |
| Austria | 1.152945474 | 1.631576909 | 1.644741286 | 2.210531644 | 3.693529244 | 6.300988861 | 15.47895439 | 41.5182887 | 94.96314688 | 174.3283501 | 305.3394694 | 432.7761619 | 564.8977895 | 1758.618593 | 606.9071119 | 571.4361207 | 390.7394995 | 3405.858789 |
| Azerbaijan | 4.416527664 | 8.560422189 | 12.58935237 | 16.76138399 | 20.95332604 | 28.11167042 | 44.85181797 | 91.88814723 | 169.7945756 | 241.2334148 | 259.0814804 | 208.0314985 | 271.083136 | 622.2457727 | 380.5167252 | 196.9554934 | 40.52908545 | 2005.93876 |
| Bahrain | 0.702026382 | 0.988385565 | 0.927571351 | 1.32229976 | 2.980382741 | 3.654514944 | 4.72903039 | 6.461801937 | 10.99851543 | 15.92397203 | 20.4496891 | 20.12753999 | 25.87377141 | 45.00261333 | 25.56792579 | 15.55162019 | 3.540882585 | 161.1821912 |
| Bangladesh | 108.7750931 | 143.6016958 | 226.1699362 | 288.8953014 | 370.1350481 | 562.806035 | 1088.324419 | 2519.577998 | 3790.041566 | 6349.908487 | 8850.480118 | 11774.1119 | 11671.11145 | 23002.23965 | 12473.27973 | 7291.07076 | 2622.595502 | 70892.80767 |
| Barbados | 0.125175868 | 0.140228567 | 0.20567814 | 0.279651775 | 0.381045117 | 0.524943703 | 0.898807188 | 1.51621782 | 2.758106751 | 4.745067103 | 5.966006097 | 8.611112713 | 9.339421664 | 27.13636246 | 9.96417962 | 9.666151699 | 5.72920225 | 62.8082031 |
| Belarus | 0.547467858 | 0.97793671 | 2.118425913 | 4.889789114 | 8.810758093 | 14.6895777 | 29.85227727 | 60.79829658 | 130.1221124 | 239.1022095 | 287.5578961 | 311.8047714 | 328.3450576 | 1017.78096 | 434.8476477 | 335.4647497 | 193.5594028 | 2437.915715 |
| Belgium | 1.868805671 | 2.488119324 | 3.40250496 | 4.552734766 | 7.519274399 | 15.05821682 | 34.32901703 | 82.12257021 | 188.0884813 | 358.7304704 | 541.8336052 | 815.7046558 | 961.9481725 | 4379.89721 | 1400.067716 | 1517.602997 | 988.1957926 | 7400.326167 |
| Belize | 0.718752006 | 0.856678753 | 1.214083234 | 1.427901911 | 1.444600667 | 1.738521784 | 2.495030122 | 3.623919399 | 5.316055513 | 6.846411562 | 7.414782868 | 8.81903307 | 8.589389125 | 21.68860826 | 9.316176824 | 6.426061103 | 3.951645638 | 73.60117125 |
| Benin | 26.65477747 | 33.41043056 | 36.43679061 | 40.05187372 | 47.18837397 | 60.68117899 | 79.59253856 | 97.44378693 | 125.5553406 | 163.7584307 | 193.6977773 | 219.812969 | 252.6169095 | 400.605327 | 204.4468279 | 121.2488792 | 57.28055225 | 1887.95238 |
| Bermuda | 0.014262299 | 0.018408572 | 0.023023196 | 0.032831639 | 0.056065768 | 0.094486457 | 0.176063942 | 0.340669731 | 0.617929753 | 1.027590746 | 1.436063159 | 2.101378378 | 2.80542255 | 9.346766131 | 3.353740568 | 3.04274542 | 1.944400559 | 18.12961888 |
| Bhutan | 0.464994982 | 0.77935408 | 1.097406322 | 1.724350164 | 2.672894174 | 4.118395751 | 7.141984145 | 12.84022148 | 21.47142364 | 38.76948435 | 59.1298419 | 87.62881721 | 103.6544425 | 228.6108656 | 117.985611 | 71.97330203 | 28.64847137 | 570.962613 |
| Bolivia (Plurinational State of) | 9.425072206 | 11.52734846 | 13.19301473 | 13.53471937 | 19.41170416 | 27.29133789 | 42.93174053 | 68.3912686 | 106.9174571 | 193.7153284 | 312.0706748 | 497.8931739 | 641.8805271 | 1549.147035 | 684.1574754 | 536.2899877 | 244.0094419 | 3577.625003 |
| Bosnia and Herzegovina | 0.335372142 | 0.505739979 | 0.444793746 | 0.769295727 | 1.313048559 | 3.102549264 | 8.129659924 | 18.02596111 | 40.79408473 | 81.93212221 | 129.5312472 | 169.2960135 | 219.684939 | 529.9860193 | 280.349158 | 174.9927926 | 58.75691825 | 1204.214642 |
| Botswana | 3.176720575 | 3.724308811 | 5.975558308 | 9.091101067 | 14.49195261 | 20.67443924 | 27.64755193 | 36.00960815 | 54.20729355 | 64.59156362 | 86.42192344 | 104.3269249 | 89.0150921 | 118.1684263 | 67.29997042 | 35.89827844 | 12.06324256 | 649.0443742 |
| Brazil | 117.8789207 | 160.5574391 | 216.1880995 | 309.1079538 | 484.7169361 | 709.9590458 | 1160.290312 | 2135.2353 | 3579.49939 | 5585.424008 | 7721.898357 | 10210.12087 | 11713.50695 | 32158.83921 | 12354.79879 | 10234.69473 | 6025.061701 | 76550.64537 |
| Brunei Darussalam | 0.170262098 | 0.238554246 | 0.362641609 | 0.527502072 | 0.768825308 | 1.1171706 | 1.688221114 | 2.315557647 | 3.744579602 | 6.292820198 | 10.59019165 | 14.14048388 | 17.15008102 | 33.54243203 | 18.21148502 | 11.56647119 | 3.163101633 | 92.97972796 |
| Bulgaria | 0.813387916 | 1.20560609 | 2.149402935 | 3.971361741 | 7.423008539 | 15.89254929 | 38.66514732 | 69.30422914 | 120.7497429 | 225.9754887 | 324.4893951 | 446.6332198 | 489.5462568 | 1288.256387 | 540.1837135 | 508.9518398 | 202.6166866 | 3035.829583 |
| Burkina Faso | 33.26756292 | 37.91804548 | 41.13388405 | 49.74983979 | 63.84826867 | 84.04937673 | 113.1572491 | 142.9821265 | 205.3235415 | 257.5152488 | 290.0800601 | 310.6420543 | 307.6575605 | 484.7206686 | 249.5380052 | 145.1365186 | 70.83355477 | 2713.915231 |
| Burundi | 34.04936772 | 42.32450482 | 35.88758442 | 44.94900686 | 56.84332254 | 66.09819324 | 84.84174112 | 119.5333635 | 207.4054574 | 289.3097571 | 311.2829446 | 300.6574293 | 263.0927539 | 391.2552676 | 231.0319164 | 107.5749629 | 43.22355406 | 2399.211093 |
| Cambodia | 17.26098809 | 26.81307461 | 30.50597707 | 34.16470564 | 42.28776956 | 54.99933542 | 90.4181777 | 180.1346657 | 305.5493071 | 472.4875681 | 591.5052461 | 743.0192382 | 755.0439456 | 1575.026687 | 759.7358182 | 546.1784229 | 208.9663832 | 4959.277205 |
| Cameroon | 59.81652532 | 75.71031427 | 86.45434754 | 104.7852556 | 123.6673398 | 153.3984446 | 190.6013592 | 229.6456715 | 311.6837995 | 404.5634729 | 471.0379971 | 500.6259582 | 516.447913 | 787.6210161 | 419.7922075 | 242.4616327 | 101.3710241 | 4165.721624 |
| Canada | 5.149157206 | 7.665125545 | 10.97402911 | 12.87255262 | 19.28315871 | 30.874797 | 69.48497481 | 173.1418621 | 420.2598685 | 851.7331571 | 1464.203296 | 2365.640727 | 2914.80388 | 10028.10496 | 3272.934127 | 3283.429449 | 2274.884094 | 18385.19028 |
| Cabo Verde | 0.496320006 | 0.709682364 | 0.883938528 | 1.184221569 | 1.461018519 | 1.887276376 | 2.7790556 | 3.778018801 | 5.841847253 | 8.069384857 | 9.181915673 | 8.821073366 | 11.75117456 | 49.51454939 | 19.02555619 | 17.28041221 | 8.945999293 | 106.7110935 |
| Central African Republic | 17.11903569 | 21.00576625 | 16.56438838 | 24.16968149 | 29.51850453 | 56.32660127 | 93.35615648 | 125.0990811 | 177.2792541 | 208.2435445 | 200.1534187 | 194.7219894 | 157.0900669 | 176.3786546 | 105.9603766 | 48.97172115 | 17.37211031 | 1578.545281 |
| Chad | 45.83166239 | 49.15899137 | 48.63489957 | 53.85183798 | 63.73254269 | 84.57536324 | 116.3082632 | 149.5292422 | 194.2972491 | 252.8239347 | 317.3839911 | 342.7992134 | 337.1071278 | 498.506815 | 263.378368 | 160.691401 | 57.30988465 | 2760.967508 |
| Chile | 4.214990304 | 5.421263204 | 7.775013522 | 9.829468821 | 13.55096792 | 20.29702001 | 38.96745439 | 84.05622077 | 172.0103168 | 313.0555321 | 484.9914615 | 697.6671627 | 954.6246309 | 3574.906702 | 1150.092045 | 1235.186554 | 813.4223747 | 6389.122437 |
| China | 178.1036219 | 330.4078411 | 571.0794917 | 1176.682979 | 1623.530297 | 3376.807393 | 6899.738588 | 15056.8003 | 23177.37946 | 42946.65276 | 83993.55948 | 148597.7525 | 193302.4858 | 563779.0124 | 260174.401 | 211459.5497 | 71931.12864 | 1085272.611 |
| Colombia | 18.70919462 | 28.49449649 | 35.97429772 | 44.48181705 | 58.32321288 | 73.82253554 | 123.1840508 | 252.4254233 | 446.2216962 | 806.8759478 | 1209.45063 | 1809.212748 | 2464.724222 | 10319.48886 | 3176.466579 | 3146.25089 | 2339.735464 | 17772.26378 |
| Comoros | 0.883021222 | 1.431051904 | 1.435287172 | 1.775045853 | 2.365388674 | 3.105761585 | 4.452495628 | 6.110553921 | 10.14924639 | 14.66376214 | 17.52823121 | 26.69172956 | 26.80209217 | 46.88814584 | 25.66496361 | 13.09195848 | 6.482790595 | 167.1424942 |
| Congo | 5.412361428 | 7.504342446 | 7.84869201 | 12.95865607 | 16.89943468 | 28.90611507 | 44.3492593 | 56.41625675 | 81.48321929 | 102.7689896 | 122.003018 | 142.4445472 | 136.7480896 | 217.5757306 | 115.9826573 | 67.36667127 | 27.99158708 | 996.9409063 |
| Costa Rica | 1.846514655 | 3.146786643 | 3.963928269 | 5.17714297 | 6.203942152 | 8.425378789 | 11.28781725 | 18.72235623 | 35.69240181 | 62.21226153 | 84.70826849 | 131.2892056 | 193.73288 | 854.0316598 | 244.5528561 | 257.2386391 | 197.9468609 | 1424.59757 |
| Côte d'Ivoire | 50.27690919 | 66.68948641 | 75.972374 | 97.74789177 | 117.1326703 | 152.9434505 | 185.0563955 | 214.269011 | 283.8046445 | 360.572915 | 412.2875514 | 411.5489753 | 423.8545001 | 649.4381191 | 338.4251738 | 202.9868426 | 86.09961787 | 3642.292848 |
| Croatia | 0.325572585 | 0.436979774 | 0.633508994 | 0.86103332 | 1.729436985 | 3.34067835 | 7.721835487 | 17.93444315 | 40.88172114 | 87.0022221 | 151.4486256 | 198.4585098 | 320.8477093 | 1045.652785 | 453.4406214 | 387.9005889 | 172.2279866 | 1877.63096 |
| Cuba | 3.316921236 | 4.80929438 | 7.312234644 | 10.60517801 | 14.12203647 | 22.35250216 | 52.38649698 | 124.1938961 | 248.6993127 | 330.518218 | 513.5256593 | 673.104379 | 782.3505179 | 1794.236766 | 709.2757503 | 543.9382765 | 341.579334 | 4586.190616 |
| Cyprus | 0.180181065 | 0.282748305 | 0.372527876 | 0.3853298 | 0.862033704 | 1.020583092 | 1.736349152 | 3.785703702 | 7.350191523 | 15.91205662 | 31.42523918 | 55.63667625 | 92.21217219 | 329.7306228 | 142.6701602 | 125.4674366 | 51.90968693 | 541.0893873 |
| Czechia | 1.306650539 | 2.233400718 | 2.818090484 | 4.108901668 | 7.157234943 | 17.39788956 | 37.34259738 | 62.85584751 | 139.7573955 | 288.5767196 | 515.1828259 | 720.3010781 | 730.6041284 | 1531.480445 | 634.3612718 | 539.8515951 | 262.1734427 | 4062.324453 |
| Democratic Republic of the Congo | 166.7499934 | 215.4650724 | 192.6474686 | 289.5440365 | 307.9130503 | 482.3532631 | 741.7211907 | 1095.278232 | 1684.565023 | 2234.486875 | 2442.30635 | 2704.676257 | 2498.40666 | 4403.786044 | 2248.292968 | 1356.075539 | 622.8581842 | 19997.35401 |
| Denmark | 1.178540916 | 1.806195468 | 1.630261321 | 1.920249541 | 3.044159715 | 6.756607396 | 17.22251856 | 47.44588853 | 104.636695 | 195.0032132 | 321.9078181 | 664.5953685 | 876.4940965 | 2327.470491 | 994.3178847 | 741.9120113 | 413.1745492 | 4572.458307 |
| Djibouti | 1.008420832 | 1.560546013 | 1.620246722 | 2.509381394 | 3.381155255 | 4.455836493 | 6.084440365 | 7.851470771 | 12.4136488 | 16.85628841 | 19.00579869 | 22.37676152 | 18.07938719 | 22.36074511 | 13.65449621 | 5.834453302 | 2.353550962 | 145.6505624 |
| Dominica | 0.075739059 | 0.082982443 | 0.115282496 | 0.136216697 | 0.176329033 | 0.197840929 | 0.326058862 | 0.636796662 | 1.030965557 | 1.585874635 | 2.118411329 | 2.653743493 | 3.299837376 | 9.236959319 | 4.161589649 | 2.920478584 | 1.620104763 | 21.86306046 |
| Dominican Republic | 10.0529034 | 16.54399128 | 19.77614292 | 24.09685811 | 27.09206044 | 37.11765162 | 50.56357604 | 77.33086012 | 98.23144841 | 157.3808667 | 164.8158563 | 242.7621127 | 230.7736181 | 728.5437673 | 260.0770178 | 273.6284755 | 136.9079365 | 1936.601797 |
| Ecuador | 9.775794173 | 14.31312143 | 15.49131542 | 14.96020111 | 19.49430273 | 23.62123906 | 34.49565929 | 50.30810388 | 82.37611113 | 127.7533285 | 203.3230717 | 344.6784326 | 505.5728031 | 2196.058474 | 679.6027258 | 790.3371437 | 566.8893083 | 3670.042249 |
| Egypt | 99.70594916 | 111.6316429 | 142.7939566 | 183.1576134 | 262.3019266 | 367.6837809 | 641.5951853 | 1292.693512 | 2091.649176 | 3059.689487 | 3635.007732 | 3326.038439 | 3116.769867 | 3931.829299 | 2154.129608 | 1235.937264 | 425.4840848 | 22560.48228 |
| El Salvador | 2.749150738 | 4.0888026 | 5.092432201 | 5.789196905 | 6.54082509 | 9.684908945 | 14.56215099 | 22.84786972 | 37.63911992 | 60.54861552 | 89.9527681 | 149.4261067 | 213.4138236 | 885.5468501 | 266.4134714 | 284.0957142 | 177.1406954 | 1513.765606 |
| Equatorial Guinea | 1.548581436 | 2.076155789 | 1.743239731 | 2.497341438 | 2.386984184 | 3.271009614 | 4.761235165 | 6.242325766 | 9.285652531 | 12.89330583 | 18.24007784 | 20.14057142 | 25.0036001 | 44.52981917 | 22.71853844 | 13.53506258 | 6.281732066 | 156.8528499 |
| Eritrea | 14.08769627 | 20.39266508 | 18.45040056 | 23.44547592 | 31.89247619 | 41.42391228 | 57.88901765 | 72.76114496 | 114.3345883 | 138.1875299 | 139.9458864 | 129.5176441 | 125.7074159 | 136.2444961 | 88.00069384 | 35.54416333 | 10.8261527 | 1116.511277 |
| Estonia | 0.101029106 | 0.165442857 | 0.283264709 | 0.472877598 | 0.651388598 | 1.129824697 | 2.523758309 | 4.325616763 | 9.658762849 | 17.38045047 | 31.081715 | 35.54752756 | 50.69190784 | 149.2844706 | 59.84954563 | 52.85627023 | 28.81675782 | 303.3777112 |
| Ethiopia | 128.0466868 | 169.0729143 | 145.9785962 | 173.3990947 | 221.1474003 | 272.624622 | 358.2664618 | 471.9840514 | 798.6602513 | 1169.121686 | 1538.285418 | 2137.298249 | 2228.269545 | 3368.252292 | 1950.6156 | 919.52859 | 413.6183887 | 13727.34303 |
| Micronesia (Federated States of) | 0.551686706 | 0.802377025 | 1.029072065 | 1.353755081 | 1.524573107 | 1.829956596 | 2.896878949 | 4.101882542 | 5.775423167 | 7.732853402 | 6.657604142 | 6.368191431 | 5.088478377 | 10.62427098 | 5.182739679 | 3.643341347 | 1.459118976 | 56.76857615 |
| Fiji | 2.766541623 | 3.321278631 | 4.092865101 | 5.817004278 | 9.315974252 | 10.73906808 | 14.9137796 | 24.21008872 | 33.48466743 | 46.24630248 | 49.67643292 | 58.4867519 | 57.32958458 | 86.21675921 | 45.32182857 | 29.36485125 | 9.252544564 | 412.4495316 |
| Finland | 0.427843718 | 0.736097897 | 0.881426477 | 0.977783965 | 1.578036557 | 2.625681748 | 5.710380059 | 16.36162337 | 40.34518797 | 87.53925877 | 174.2690417 | 314.54204 | 353.4388276 | 1043.233567 | 414.6383491 | 343.5203495 | 202.1949267 | 2043.229149 |
| France | 8.952397441 | 11.6010685 | 13.98139797 | 17.66032563 | 29.68088524 | 53.72623315 | 114.3601206 | 227.5514025 | 434.4181729 | 774.337429 | 1255.90903 | 1914.731559 | 2301.679615 | 14890.90486 | 3636.149189 | 4874.292204 | 4152.159636 | 22060.48034 |
| Gabon | 1.46269895 | 2.099210943 | 1.911024344 | 2.980289662 | 3.41998186 | 5.956064251 | 9.685396408 | 13.81405203 | 22.76840563 | 30.60941342 | 38.90078344 | 38.99906876 | 43.55437175 | 89.57834194 | 42.78629464 | 29.08001477 | 13.63411228 | 308.1771368 |
| Georgia | 2.407588837 | 3.20862334 | 4.81944796 | 6.23150339 | 9.1241926 | 14.46905248 | 20.3436571 | 33.3376273 | 59.27289859 | 88.93670314 | 119.6736176 | 138.6781129 | 178.8730051 | 539.0299715 | 242.9460714 | 145.6587182 | 104.889675 | 1221.558412 |
| Germany | 9.905531944 | 15.06528774 | 18.60140545 | 27.35105972 | 45.73293208 | 79.67407193 | 196.0383606 | 592.6315311 | 1355.913107 | 2363.063598 | 3631.288463 | 4647.456753 | 7418.894645 | 24751.72817 | 10399.1527 | 8025.569556 | 4542.878625 | 45165.49489 |
| Ghana | 67.97464966 | 101.1850239 | 107.509838 | 134.9139415 | 156.3057242 | 219.6724972 | 279.3890014 | 331.7955895 | 437.3320122 | 608.3817244 | 736.8870693 | 736.2021375 | 745.7954574 | 1145.291627 | 613.2197044 | 360.7391808 | 135.4523344 | 5938.018657 |
| Greece | 1.065961642 | 1.47639573 | 1.772969319 | 2.732883246 | 5.180536756 | 9.956008843 | 19.28102867 | 41.44594394 | 66.14380791 | 129.462205 | 224.6841666 | 418.4214515 | 697.467069 | 4779.091996 | 1361.737485 | 1753.909001 | 1194.501602 | 6399.324642 |
| Greenland | 0.011199028 | 0.026070726 | 0.039594717 | 0.055413123 | 0.093578582 | 0.126400771 | 0.343704139 | 1.235415813 | 2.229837914 | 3.436818013 | 4.12911466 | 4.96707841 | 5.791229587 | 9.470575051 | 4.706372146 | 3.266221876 | 1.225895049 | 32.00005164 |
| Grenada | 0.075508647 | 0.106238857 | 0.125921011 | 0.163329782 | 0.198858204 | 0.267252553 | 0.47796671 | 0.863580274 | 1.35530963 | 1.830463065 | 2.437388254 | 3.061159654 | 3.350758066 | 4.583651976 | 2.129337033 | 1.263824973 | 0.796369378 | 19.04993355 |
| Guam | 0.233786038 | 0.259722449 | 0.380305123 | 0.539847459 | 0.86212375 | 0.959984837 | 1.317529366 | 2.316703886 | 3.150867967 | 4.207597802 | 5.301900594 | 5.766346974 | 5.35623612 | 22.10569505 | 6.684460023 | 6.34119096 | 5.418812665 | 53.0382222 |
| Guatemala | 20.063779 | 26.3398396 | 27.63315916 | 29.09186001 | 33.5425206 | 39.28274187 | 45.2170438 | 58.8218014 | 84.94133971 | 131.1585283 | 185.926005 | 258.3426694 | 305.1502729 | 1224.868772 | 543.0030931 | 440.1212497 | 208.901801 | 2529.11819 |
| Guinea | 35.96524644 | 43.24998961 | 48.6625023 | 56.13809538 | 62.91669927 | 77.1703192 | 102.4730609 | 131.1800532 | 184.8626189 | 259.260754 | 320.8820518 | 338.2949852 | 338.0960944 | 632.3360669 | 294.0905912 | 201.9995589 | 106.2790201 | 2787.320365 |
| Guinea-Bissau | 6.290598799 | 8.299872509 | 9.37075577 | 11.4601712 | 14.26608432 | 17.52525328 | 22.42748445 | 25.77879194 | 34.15577983 | 41.1195903 | 46.64287685 | 45.6441956 | 40.24605949 | 50.59863446 | 27.47100182 | 15.73376265 | 5.90164387 | 385.64617 |
| Guyana | 0.890911416 | 1.548435515 | 1.973553641 | 2.007897911 | 2.564565199 | 3.631362161 | 4.87066897 | 7.349878944 | 10.09202194 | 12.91978013 | 14.50949692 | 14.47645791 | 13.6559164 | 25.87267834 | 11.76541614 | 7.592283 | 4.772334686 | 118.5745698 |
| Haiti | 31.23290274 | 35.20557439 | 49.3586202 | 62.12739031 | 79.45450112 | 98.50129894 | 119.0618908 | 169.0247949 | 229.6529546 | 321.9961625 | 391.9495815 | 408.0404871 | 394.5545663 | 630.8075267 | 332.3257709 | 195.3085364 | 80.41383214 | 3419.913818 |
| Honduras | 13.39080359 | 15.72871207 | 16.26727289 | 18.9773763 | 24.80180894 | 43.29033717 | 69.09491764 | 110.7717395 | 183.6320891 | 298.8834953 | 416.3033849 | 570.7216414 | 609.3358709 | 1078.082658 | 550.6864927 | 345.018781 | 139.3998219 | 3536.333094 |
| Hungary | 1.421855377 | 2.372186914 | 3.825463237 | 5.955961436 | 14.94881225 | 39.22791974 | 91.67313263 | 178.4397818 | 292.0291177 | 571.9149623 | 757.014473 | 826.7513323 | 923.3155423 | 2224.713394 | 918.8898508 | 769.5653901 | 400.6320827 | 5935.529727 |
| Iceland | 0.062261199 | 0.101309631 | 0.111372557 | 0.121652834 | 0.187707146 | 0.290244215 | 0.561279532 | 1.155855129 | 2.231470266 | 4.027026828 | 7.100058803 | 11.58584274 | 14.8508021 | 68.35685013 | 20.71661017 | 22.01954905 | 15.69476804 | 110.810542 |
| India | 998.5037209 | 1753.179854 | 2273.630286 | 3670.300894 | 6457.199391 | 10454.69385 | 18776.08237 | 37433.78243 | 60940.21443 | 102575.8216 | 151168.5794 | 195730.3094 | 201551.9022 | 373409.2331 | 216156.2197 | 104174.253 | 43882.42557 | 1168381.111 |
| Indonesia | 464.5918731 | 670.6746976 | 690.8771342 | 767.5352321 | 1100.76023 | 1644.069677 | 2643.312285 | 4260.62483 | 6476.140135 | 10595.10745 | 13467.73029 | 15505.26068 | 15265.36272 | 26617.97653 | 13484.27158 | 8914.116332 | 3345.615324 | 100930.0101 |
| Iran (Islamic Republic of) | 56.14970986 | 61.06553656 | 92.18463231 | 160.2031658 | 208.5771642 | 247.6047036 | 358.6244075 | 557.7763967 | 835.2964047 | 1247.221849 | 1478.575097 | 1739.642076 | 2366.764957 | 7346.911752 | 2908.81218 | 2681.168145 | 1338.282397 | 16834.61998 |
| Iraq | 31.07338093 | 35.16189839 | 40.31267106 | 45.76001026 | 59.1624516 | 89.02412715 | 156.9453582 | 185.5141368 | 235.37609 | 309.3104686 | 377.8453743 | 437.2260989 | 424.2960382 | 807.3269445 | 364.0345859 | 254.667498 | 134.5137521 | 3292.302351 |
| Ireland | 1.244878209 | 1.347479094 | 1.552886442 | 2.200168098 | 3.166158168 | 5.328023132 | 9.734000866 | 20.23814165 | 43.15422969 | 86.93751848 | 168.5218232 | 301.782825 | 428.0210091 | 1473.404814 | 522.6121294 | 509.1952981 | 304.7904093 | 2547.961094 |
| Israel | 2.317343905 | 2.339226217 | 2.648907835 | 3.41944894 | 4.584657466 | 8.36280964 | 15.81463364 | 29.87121225 | 52.77672992 | 91.32393209 | 157.9313501 | 243.5741337 | 269.1318 | 1137.546629 | 361.3099222 | 370.9410258 | 261.9441312 | 2026.876146 |
| Italy | 5.575948198 | 7.467612851 | 9.783290466 | 12.93880512 | 21.15444465 | 42.26743556 | 85.6049103 | 181.4017472 | 335.3851683 | 616.7875658 | 1162.981302 | 2225.018432 | 3847.679015 | 22784.31788 | 6412.261695 | 7952.731066 | 5894.317663 | 31345.33014 |
| Jamaica | 1.840660396 | 2.734687569 | 3.764605867 | 4.424381652 | 5.802553108 | 7.413006904 | 11.18561287 | 19.72569673 | 33.6604096 | 48.08197923 | 59.93345221 | 92.98036221 | 92.13774544 | 252.766868 | 88.95278009 | 73.46957979 | 50.07217851 | 641.6670952 |
| Japan | 9.634010533 | 13.42618712 | 16.27976947 | 25.91907701 | 41.39415429 | 72.03011551 | 142.7132113 | 245.8639128 | 443.6739307 | 975.9260661 | 2316.445743 | 4949.482947 | 8059.557211 | 38454.01751 | 11153.80301 | 12838.08857 | 9023.060734 | 55793.66713 |
| Jordan | 5.229825931 | 4.702618257 | 5.733741519 | 7.661270959 | 11.19766631 | 16.50635174 | 25.81526101 | 41.11407632 | 56.51180076 | 75.30484762 | 103.3320688 | 122.6939325 | 150.5539116 | 222.7596899 | 114.647199 | 65.66632173 | 29.53975632 | 855.995213 |
| Kazakhstan | 7.882290402 | 14.97367359 | 30.09619048 | 52.93077612 | 82.29415455 | 113.6239623 | 188.5254096 | 321.1607133 | 630.9273948 | 1008.085497 | 1424.590988 | 1346.923197 | 1612.456139 | 2927.520809 | 1770.419846 | 696.0242455 | 390.0838209 | 9772.387005 |
| Kenya | 77.47644064 | 104.3459083 | 92.36423971 | 122.4605792 | 167.8743161 | 202.4792186 | 282.7315049 | 363.5443194 | 599.3869543 | 811.0245203 | 930.1116272 | 1141.182962 | 1063.244424 | 1578.511997 | 891.7039766 | 445.7920022 | 193.0056974 | 7704.710402 |
| Kiribati | 0.874783437 | 1.051106295 | 1.411575098 | 1.859906678 | 2.594339624 | 2.944795214 | 3.393313933 | 4.882371876 | 7.151756099 | 8.889813649 | 8.067709265 | 8.520522508 | 9.617611334 | 10.3289397 | 6.498749925 | 2.773525 | 0.879091631 | 72.9228226 |
| Kuwait | 0.646993713 | 0.746168233 | 1.472900987 | 3.131390659 | 3.279670775 | 4.738860626 | 5.947110566 | 8.351817643 | 11.42190097 | 14.94269197 | 16.72689451 | 26.47113144 | 25.48035135 | 104.0966835 | 34.03230251 | 36.60822361 | 22.86485776 | 229.5631745 |
| Kyrgyzstan | 1.791429659 | 4.576679459 | 9.135690845 | 13.56240131 | 17.03703314 | 23.74356925 | 33.41011838 | 56.14964075 | 96.33785321 | 136.9495463 | 174.791995 | 133.2541096 | 190.3779961 | 620.8343884 | 265.3946886 | 208.4056063 | 118.4754436 | 1513.466354 |
| Lao People's Democratic Republic | 17.48821137 | 23.41465801 | 27.75002565 | 32.54962454 | 39.64795452 | 52.02926193 | 73.15623924 | 110.7084695 | 173.4200222 | 270.9759157 | 334.1276747 | 377.5165357 | 398.068546 | 679.1489627 | 350.3154875 | 216.1277843 | 85.89895239 | 2663.067908 |
| Latvia | 0.137154889 | 0.175314641 | 0.417104546 | 0.68545716 | 1.041536594 | 1.707061852 | 3.780593285 | 8.143354681 | 17.27257993 | 29.41160964 | 40.94525682 | 50.27071957 | 72.47851459 | 206.4177891 | 83.99646815 | 70.04621057 | 38.76426426 | 433.0040333 |
| Lebanon | 2.480848632 | 3.015665872 | 4.93853422 | 7.177302935 | 8.69489148 | 11.26520111 | 18.00136714 | 27.05779885 | 45.29947211 | 64.06519481 | 105.8484796 | 155.0899486 | 200.3058645 | 529.2919987 | 221.5971397 | 184.4011518 | 93.8218698 | 1185.206989 |
| Lesotho | 6.529031455 | 7.194915185 | 10.08473175 | 14.13201029 | 21.67170239 | 31.48539689 | 43.52061004 | 65.58145556 | 100.5305124 | 116.466311 | 160.2579479 | 189.9610314 | 149.3594257 | 185.2569003 | 107.7005816 | 54.88776304 | 18.32878243 | 1115.955217 |
| Liberia | 6.412024343 | 8.761128223 | 8.971762558 | 10.36335622 | 14.57410513 | 19.75172262 | 28.82722021 | 27.80202158 | 36.45123475 | 45.29820368 | 56.24935342 | 59.27049003 | 62.15781704 | 124.4672201 | 56.71196152 | 41.15443915 | 20.54265283 | 534.4396498 |
| Libya | 4.184768033 | 5.093208785 | 6.99286069 | 12.64825685 | 19.95767159 | 30.53539251 | 46.53597209 | 54.80474505 | 67.43382625 | 91.47913005 | 112.7806711 | 133.2347752 | 173.7994649 | 407.9913678 | 153.9513247 | 134.6178638 | 78.9625884 | 1172.90343 |
| Lithuania | 0.173850902 | 0.286350851 | 0.51944674 | 1.030059726 | 1.45023283 | 2.880116334 | 6.454152221 | 13.76163277 | 30.31459175 | 55.09151836 | 74.79517017 | 87.52965302 | 130.7308272 | 373.5576207 | 154.2132638 | 137.6993526 | 66.06685226 | 778.7270363 |
| Luxembourg | 0.114965255 | 0.180914369 | 0.228034993 | 0.300859543 | 0.425967902 | 0.649334619 | 1.287620822 | 2.799024155 | 5.100371512 | 8.89622276 | 15.48580707 | 23.11807421 | 32.24476803 | 130.4432536 | 40.55347742 | 44.4100507 | 30.64183495 | 221.3781419 |
| North Macedonia | 0.308959022 | 0.436253361 | 0.54124348 | 0.695287436 | 1.539873 | 2.339863865 | 6.319690101 | 12.04045027 | 25.17806653 | 47.93926783 | 77.36556882 | 98.4501191 | 141.8219817 | 236.9233396 | 130.4626527 | 84.33888777 | 20.59394307 | 652.3759566 |
| Madagascar | 137.908695 | 196.9671351 | 178.2310755 | 186.3448332 | 207.2161589 | 191.0339414 | 236.5451253 | 314.4455367 | 497.4517064 | 657.8072889 | 672.9385751 | 702.787215 | 592.7260031 | 906.5847603 | 486.7346104 | 270.7731659 | 119.5478048 | 6029.567066 |
| Malawi | 31.7337328 | 38.57228847 | 29.33065615 | 38.49172709 | 46.39113417 | 57.78004102 | 79.14843445 | 94.47622355 | 153.0557943 | 231.9089228 | 309.1857071 | 382.2901207 | 334.3536905 | 462.2390194 | 261.8191827 | 124.1017023 | 60.55694192 | 2402.226561 |
| Malaysia | 17.89880283 | 30.62821498 | 46.98651341 | 63.65545328 | 80.54357493 | 93.38515446 | 133.1371936 | 217.0872867 | 359.2297988 | 616.2892601 | 904.9033739 | 1299.51831 | 1209.332727 | 2382.045548 | 1141.50526 | 717.6832713 | 420.2445142 | 7473.115965 |
| Maldives | 0.240248883 | 0.39051888 | 0.798108186 | 1.317727621 | 1.647944526 | 1.762678311 | 2.278363188 | 3.141879421 | 4.723736418 | 7.127063112 | 8.320080903 | 11.83147596 | 19.15477048 | 59.01034432 | 23.70053705 | 21.61484556 | 10.18933336 | 122.9578629 |
| Mali | 64.1593858 | 91.34762727 | 107.9317605 | 115.9104493 | 119.1417447 | 150.9755801 | 227.3993499 | 297.2060939 | 410.1507585 | 519.6772524 | 627.8573369 | 608.8319005 | 512.74246 | 653.6688769 | 341.7085038 | 195.6964809 | 90.26586049 | 4573.530304 |
| Malta | 0.070669406 | 0.100392365 | 0.137304123 | 0.159059745 | 0.232715493 | 0.365328105 | 0.629079842 | 1.158762767 | 2.629089489 | 5.969262818 | 12.68686332 | 24.02651334 | 24.72743866 | 84.27533496 | 33.15936257 | 27.63947662 | 15.09095733 | 157.2425063 |
| Marshall Islands | 0.274483987 | 0.386542993 | 0.537981283 | 0.852083767 | 1.243061323 | 1.329643562 | 1.544980381 | 1.870012931 | 2.349149804 | 3.067509956 | 3.514412541 | 3.286826378 | 2.440471265 | 3.380273376 | 1.817732995 | 1.044263539 | 0.405886914 | 26.41049332 |
| Mauritania | 4.186751272 | 5.425513518 | 5.92684102 | 6.981274963 | 8.377692191 | 10.88239332 | 15.77445782 | 21.02408168 | 31.05931331 | 45.55577511 | 59.09740796 | 65.94315944 | 76.45390336 | 159.9628744 | 71.91880156 | 55.58165933 | 23.37005969 | 524.6967928 |
| Mauritius | 1.278609003 | 2.031521906 | 2.474016593 | 2.351911283 | 4.831097811 | 6.949196322 | 9.360661283 | 13.73543123 | 22.15625157 | 39.83905074 | 55.71355628 | 70.92918392 | 74.475059 | 228.7342446 | 78.76473571 | 71.3141898 | 48.46707761 | 537.3497923 |
| Mexico | 71.61936029 | 106.0116178 | 131.7691725 | 164.3251342 | 226.664311 | 331.631529 | 507.2062184 | 807.226052 | 1272.277717 | 2039.904043 | 2885.75051 | 4243.259768 | 5746.576577 | 19767.60209 | 6831.448025 | 6607.435304 | 4285.989677 | 38524.30795 |
| Republic of Moldova | 0.28838064 | 0.515183159 | 1.034023918 | 2.2142813 | 3.993558575 | 6.891241627 | 12.21341255 | 22.93282502 | 50.55504246 | 93.67347319 | 131.2875871 | 102.168745 | 147.1346744 | 396.1974652 | 173.6146721 | 121.1736409 | 70.04559825 | 971.3868656 |
| Mongolia | 1.372401597 | 2.675413971 | 3.598778761 | 6.050277723 | 7.734608755 | 12.65394273 | 17.40246529 | 29.96289369 | 42.28598159 | 56.06817 | 53.84558427 | 50.21021773 | 56.51955816 | 108.5423042 | 55.03045575 | 43.25896428 | 9.458184074 | 453.1328117 |
| Montenegro | 0.02837462 | 0.054837172 | 0.056059619 | 0.071637714 | 0.136609221 | 0.223794903 | 0.485161914 | 1.127741691 | 2.42160433 | 5.080949853 | 9.018566841 | 10.56876417 | 15.13159116 | 40.54350772 | 19.45753128 | 14.17858202 | 5.504452084 | 84.9790984 |
| Morocco | 28.81937433 | 28.24993923 | 36.6824059 | 55.31172533 | 85.21655651 | 137.1887263 | 224.9298155 | 362.1958856 | 620.5753033 | 926.3545765 | 1154.600478 | 1226.596872 | 1418.305825 | 3026.928679 | 1380.701218 | 967.8529616 | 487.4890071 | 9373.436508 |
| Mozambique | 56.34307719 | 67.59916713 | 54.41042293 | 60.50375155 | 75.28188414 | 111.6735425 | 156.3221647 | 184.3378649 | 280.3236326 | 419.2174478 | 496.6070424 | 518.4146355 | 406.2446131 | 662.4002682 | 380.78778 | 179.5681881 | 80.49347874 | 3758.835346 |
| Myanmar | 137.7446593 | 183.1932433 | 191.4182313 | 239.2517834 | 309.379386 | 453.4359759 | 742.2802076 | 1479.210565 | 2531.316699 | 3969.844924 | 5147.551949 | 6301.966923 | 6356.276009 | 14827.69562 | 6896.010878 | 5027.433633 | 2259.737819 | 43284.39924 |
| Namibia | 3.413418407 | 3.512822495 | 4.629693458 | 6.120563069 | 8.917038847 | 14.37104817 | 22.30098979 | 33.30180375 | 48.73790735 | 60.280076 | 88.27111661 | 131.2298489 | 139.9946406 | 217.4611176 | 114.6688769 | 67.75252622 | 26.5050326 | 790.9723891 |
| Nepal | 19.59214742 | 39.00142158 | 48.88760508 | 60.81878643 | 118.731148 | 285.7582404 | 706.5957035 | 1378.573303 | 2139.83553 | 3775.510974 | 5586.126058 | 6882.575562 | 7160.957041 | 12582.52385 | 6421.791469 | 3950.395688 | 1616.099175 | 40799.55377 |
| Netherlands | 2.246774902 | 2.612415941 | 3.535010176 | 4.529323561 | 6.988099766 | 14.13754035 | 43.11104896 | 104.1747817 | 229.6423615 | 430.3007534 | 733.3865704 | 1368.95181 | 1715.846641 | 6103.764355 | 2116.352046 | 2075.021325 | 1320.326707 | 10766.17353 |
| New Zealand | 2.299521651 | 2.738332769 | 3.193946912 | 3.661655608 | 4.47660988 | 6.969022266 | 15.21494229 | 30.21197968 | 62.4260157 | 111.6408644 | 195.2802688 | 319.9697766 | 400.7127655 | 1234.897014 | 422.8299149 | 414.3321882 | 272.7413887 | 2396.172231 |
| Nicaragua | 2.630487068 | 2.904137097 | 2.678661182 | 3.284739412 | 4.292055125 | 7.304631501 | 13.58200539 | 21.53229923 | 37.41403724 | 69.50266054 | 109.0434384 | 180.38552 | 242.537275 | 627.6830361 | 272.5163236 | 215.3906273 | 103.2723511 | 1342.915834 |
| Niger | 59.64024229 | 64.95557678 | 66.69422065 | 69.46482589 | 84.86665202 | 102.6819334 | 132.5871381 | 189.9694355 | 277.479474 | 371.0815005 | 419.5652142 | 440.6346741 | 456.6668033 | 532.9897536 | 287.9004531 | 149.0463064 | 76.24380869 | 3618.958655 |
| Nigeria | 204.8886203 | 251.8505874 | 273.8815557 | 330.394072 | 420.4684767 | 560.0953458 | 851.1788778 | 1151.685432 | 1694.687054 | 2237.346235 | 2698.714793 | 2887.769585 | 3804.500388 | 6130.078097 | 3083.15423 | 1845.825286 | 931.3963773 | 24505.92168 |
| Democratic People's Republic of Korea | 15.48349047 | 26.68563157 | 39.41221329 | 56.79224128 | 87.14736868 | 166.1702791 | 397.5778117 | 864.0460189 | 1321.581908 | 2278.544367 | 2863.596954 | 4859.394775 | 6347.168864 | 12370.47638 | 6504.979816 | 3784.737622 | 1600.315959 | 31704.02007 |
| Northern Mariana Islands | 0.068755702 | 0.074605704 | 0.089596832 | 0.083487783 | 0.1354069 | 0.322431339 | 0.62114054 | 1.210818878 | 1.831991467 | 2.126123071 | 2.689484858 | 1.650894144 | 2.345353546 | 4.06459227 | 1.628274724 | 1.443481736 | 0.676062337 | 17.36868689 |
| Norway | 0.744345223 | 1.020410939 | 1.415326297 | 1.727871198 | 2.914488648 | 4.765049967 | 10.78571989 | 25.41962926 | 48.4585902 | 117.5947338 | 213.4551871 | 408.1760578 | 468.9496758 | 1464.503377 | 515.4852845 | 491.8339494 | 305.7962065 | 2770.71138 |
| Oman | 1.608576146 | 2.661711245 | 3.495314431 | 4.281075142 | 4.185453274 | 4.620270611 | 6.280558831 | 8.518772787 | 14.60027712 | 21.52658568 | 32.07453433 | 40.15079511 | 41.39008706 | 57.00030371 | 32.02898453 | 14.18806754 | 9.040514747 | 243.7357104 |
| Pakistan | 254.2899932 | 364.9030202 | 493.3930128 | 675.1340945 | 932.1858173 | 1398.648358 | 2161.322381 | 3698.858538 | 5399.898911 | 8552.349453 | 10633.74576 | 13260.06821 | 12829.34529 | 21613.74811 | 12134.99778 | 6235.468751 | 2480.161011 | 82757.82455 |
| Palestine | 2.255419504 | 2.794308762 | 3.963318788 | 4.05050024 | 5.488972188 | 7.396482543 | 13.39035492 | 22.17091372 | 35.16499162 | 45.66405552 | 59.80201369 | 70.94826514 | 76.59871536 | 133.3860368 | 61.5217281 | 46.85974198 | 19.34370664 | 487.9100882 |
| Panama | 2.383986741 | 2.862634955 | 3.214311555 | 3.72619275 | 4.188722754 | 6.810228287 | 9.742403856 | 14.48885486 | 26.69017133 | 36.96427739 | 56.71661361 | 86.25430808 | 114.5499177 | 497.1733907 | 140.7930768 | 142.0741254 | 116.9467302 | 876.6617689 |
| Papua New Guinea | 57.82134053 | 70.75717349 | 86.75523505 | 113.5330118 | 153.4088765 | 201.7218321 | 287.2333653 | 422.5070212 | 586.1403732 | 791.1081919 | 894.7740815 | 1062.030357 | 875.9360266 | 1325.979022 | 673.216074 | 390.9688728 | 194.9029966 | 7154.076229 |
| Paraguay | 3.826823911 | 4.890569848 | 6.100198715 | 7.557638689 | 8.965192793 | 12.16111304 | 18.30410537 | 30.53738981 | 49.35499469 | 84.04859581 | 113.0747497 | 146.5796599 | 174.2063338 | 482.8384544 | 181.1380554 | 140.1175084 | 88.55656928 | 1147.832966 |
| Peru | 20.29592361 | 28.09393901 | 37.19610614 | 38.27936019 | 49.0917026 | 63.08184515 | 88.19271588 | 133.9738024 | 194.4598623 | 302.379816 | 462.3976136 | 685.1970752 | 966.3114652 | 3839.671335 | 1169.717977 | 1274.90403 | 865.3644937 | 6961.172518 |
| Philippines | 192.1893763 | 300.298765 | 375.2458806 | 425.8666094 | 540.8453384 | 675.9439874 | 966.0133488 | 1393.413318 | 2145.772417 | 3402.222167 | 4419.858894 | 4842.657594 | 4588.06668 | 7976.919799 | 3982.486985 | 2396.232298 | 1182.358715 | 33228.90956 |
| Poland | 2.789079025 | 4.095415895 | 6.498565933 | 10.94171252 | 22.37908686 | 43.41498897 | 90.84534726 | 179.3262418 | 406.1522768 | 945.4395641 | 1456.905349 | 1688.014215 | 1601.848197 | 4887.341296 | 1968.996667 | 1757.848116 | 876.9374836 | 11348.61622 |
| Portugal | 1.739723736 | 2.660167466 | 3.14231017 | 3.780090482 | 7.397952837 | 15.97132342 | 28.2618711 | 54.58819432 | 96.6308526 | 167.4992409 | 282.5839792 | 512.691942 | 846.1306203 | 5027.244017 | 1473.158746 | 1794.493467 | 1230.921837 | 7051.852114 |
| Puerto Rico | 1.325409016 | 2.090261691 | 3.105704225 | 3.947486145 | 6.111862774 | 9.277923344 | 14.18265639 | 27.66678462 | 43.90746782 | 75.94059 | 112.7682651 | 198.6606788 | 247.2308445 | 1083.98382 | 308.8082425 | 313.1218262 | 239.9641984 | 1832.095505 |
| Qatar | 0.485553254 | 0.971538218 | 1.996567912 | 2.588423617 | 3.302721145 | 2.884458026 | 4.160342605 | 3.608278176 | 5.350143716 | 6.245487094 | 8.012847111 | 8.659693425 | 7.804443452 | 8.807626142 | 5.76354036 | 2.413894239 | 0.559482275 | 66.18849463 |
| Romania | 1.976083247 | 2.615615706 | 4.257297854 | 8.446915776 | 19.0554032 | 52.87905141 | 121.6727466 | 235.6296665 | 330.3994784 | 641.4236873 | 875.0874579 | 992.553615 | 1246.269847 | 3571.499905 | 1581.651201 | 1238.502197 | 588.9238215 | 8106.340964 |
| Russian Federation | 17.64821013 | 29.15449369 | 59.49477077 | 143.0230734 | 223.8115152 | 300.5573082 | 507.2445634 | 925.7042211 | 2112.238468 | 3984.535446 | 5227.28211 | 5425.871508 | 5151.083825 | 15261.75576 | 7253.643269 | 4533.701665 | 2799.941638 | 39390.41143 |
| Rwanda | 26.93791579 | 37.37708787 | 31.80590751 | 45.37971232 | 54.27063467 | 58.05717511 | 72.80970578 | 105.340081 | 192.2317355 | 281.4868268 | 331.1011396 | 338.4666128 | 321.9326464 | 515.7478047 | 297.2699524 | 143.4124536 | 62.35636901 | 2519.692366 |
| Saint Lucia | 0.172823241 | 0.267290343 | 0.40441867 | 0.482006251 | 0.666850437 | 1.039866239 | 1.698204325 | 2.563689957 | 3.432660445 | 4.7682686 | 4.810409776 | 8.02800739 | 7.847557775 | 22.13282611 | 8.396910658 | 7.030179806 | 5.132485979 | 58.68974097 |
| Saint Vincent and the Grenadines | 0.069557139 | 0.080470344 | 0.119372025 | 0.136495075 | 0.202696753 | 0.309751754 | 0.55033175 | 0.853428008 | 1.228263166 | 1.678483025 | 1.97059267 | 2.48391141 | 3.07957544 | 7.159738383 | 3.27963615 | 2.503343707 | 1.105865903 | 20.06531863 |
| Samoa | 0.784229838 | 0.918822447 | 1.064784749 | 1.538620346 | 2.07865271 | 2.450096955 | 3.570367955 | 4.883988745 | 6.683198237 | 8.748634582 | 9.809234498 | 10.88851895 | 13.96635663 | 36.76839647 | 15.74134066 | 12.09061797 | 6.752857412 | 104.8317172 |
| Sao Tome and Principe | 0.794735296 | 0.934439977 | 0.951121526 | 1.159587369 | 1.522889657 | 1.947331686 | 2.980796313 | 3.781486234 | 5.688430795 | 7.619384614 | 9.4452786 | 9.584044184 | 10.7341832 | 20.76998391 | 10.12080895 | 6.778888172 | 3.028580306 | 78.64908435 |
| Saudi Arabia | 16.21596001 | 27.93294176 | 43.1318361 | 80.10960828 | 136.2477588 | 187.2342007 | 251.7523497 | 272.6796512 | 336.6043033 | 387.566973 | 419.9504483 | 421.185572 | 377.063771 | 754.4132854 | 291.4968961 | 239.4159257 | 168.4162191 | 3723.314895 |
| Senegal | 31.45313114 | 40.78130113 | 42.87043888 | 47.64195856 | 54.16433517 | 70.91691698 | 95.80057965 | 128.7048335 | 189.6530368 | 262.8314631 | 317.9338756 | 352.1854972 | 375.9612482 | 691.4044073 | 336.5122892 | 221.5175098 | 103.4544325 | 2762.951066 |
| Serbia | 1.118666932 | 1.783348195 | 2.481665842 | 3.426955961 | 6.734508175 | 13.00761178 | 29.27561897 | 67.69261446 | 129.6355979 | 281.9930588 | 506.8305872 | 492.808708 | 672.6617381 | 1654.656044 | 964.6776848 | 524.8184577 | 140.5756866 | 3865.050405 |
| Seychelles | 0.041591072 | 0.086561828 | 0.12851318 | 0.20123632 | 0.280916545 | 0.467287053 | 0.712511758 | 1.044337923 | 1.645208361 | 2.560595902 | 3.14857849 | 3.53700812 | 4.949698762 | 11.63848745 | 4.707269591 | 3.997961047 | 2.140574566 | 30.51418223 |
| Sierra Leone | 19.61578929 | 25.66838394 | 29.6744171 | 33.69882539 | 38.20803303 | 48.20631067 | 60.55196603 | 74.68487521 | 96.97460617 | 127.5999745 | 157.6686631 | 173.4088822 | 198.2796268 | 311.822111 | 164.3928689 | 91.70135739 | 43.56803483 | 1478.616974 |
| Singapore | 0.424040974 | 0.538244596 | 1.169423032 | 1.445240258 | 2.892058685 | 3.983624632 | 6.482643491 | 12.61308631 | 24.34827295 | 44.50723027 | 67.59877425 | 108.6477306 | 119.6894847 | 354.7366057 | 134.5848005 | 110.766209 | 68.74105211 | 749.9192694 |
| Slovakia | 0.618914405 | 1.061549495 | 1.312526702 | 2.210368747 | 4.495603338 | 7.907045054 | 14.03776919 | 27.04754943 | 53.1210452 | 102.1609441 | 145.3115615 | 166.2700912 | 184.7887148 | 435.4666156 | 182.8625614 | 150.6650352 | 73.62519993 | 1146.702909 |
| Slovenia | 0.105126414 | 0.127627383 | 0.151869356 | 0.239197335 | 0.533108621 | 0.929089032 | 2.215693265 | 5.213163828 | 11.65634666 | 25.81676476 | 48.34225353 | 65.66984142 | 102.6067091 | 374.9090887 | 142.3101896 | 125.851743 | 75.46532206 | 638.617668 |
| Solomon Islands | 3.26728254 | 5.163580059 | 6.703975568 | 8.672248096 | 11.74588652 | 15.67209117 | 21.57595031 | 27.81232291 | 34.14100703 | 40.09620458 | 43.91490505 | 43.79179277 | 37.11569184 | 42.98358168 | 27.77442979 | 11.75591909 | 2.916164977 | 346.6792848 |
| Somalia | 68.27608034 | 83.14307304 | 64.69524864 | 72.22632992 | 111.4560374 | 169.2521737 | 200.9296652 | 187.6357409 | 328.4250445 | 491.3066371 | 572.3833836 | 536.1883566 | 380.1383022 | 372.2075209 | 244.342458 | 91.84642116 | 30.67129114 | 3992.267114 |
| South Africa | 33.55002338 | 69.77661468 | 169.8039995 | 272.6194238 | 289.7289863 | 318.622112 | 404.9689359 | 654.4629533 | 1127.626057 | 1515.996188 | 2079.016484 | 2612.021865 | 2408.925115 | 5765.775136 | 2604.290235 | 1968.691734 | 927.5515509 | 17826.87421 |
| Republic of Korea | 2.730296676 | 4.107965552 | 6.249554425 | 8.673199002 | 16.7738287 | 28.02303337 | 62.23144547 | 129.9914616 | 255.6213925 | 508.7471798 | 763.1142801 | 1395.570448 | 2483.706985 | 9834.875588 | 3387.946844 | 3369.194071 | 2073.561584 | 15506.39098 |
| South Sudan | 14.24024963 | 15.03097993 | 10.42199042 | 15.2421386 | 22.55700071 | 29.08643535 | 42.3831067 | 60.11062691 | 94.64716375 | 125.761306 | 142.6489745 | 186.8941254 | 179.9763612 | 286.8122355 | 154.8776587 | 76.12739081 | 43.96821784 | 1330.771087 |
| Spain | 6.178223324 | 7.324071903 | 10.40294704 | 15.33878527 | 28.333753 | 56.62917812 | 112.7952357 | 232.2788657 | 421.0680162 | 765.1256854 | 1303.415357 | 2461.624416 | 3893.917561 | 27238.9631 | 6652.680278 | 9577.855355 | 7317.091143 | 36560.14027 |
| Sri Lanka | 16.74823758 | 24.75129332 | 29.89394082 | 39.11690425 | 62.14935244 | 90.17257408 | 152.9333869 | 273.9588011 | 468.8464839 | 886.818424 | 1507.270244 | 2162.863603 | 2173.370724 | 5685.361606 | 2288.49328 | 2046.48146 | 996.7338693 | 13596.92952 |
| Sudan | 63.59070852 | 67.6398294 | 80.72500771 | 103.3537486 | 127.2753802 | 166.9084457 | 230.7347558 | 323.3369456 | 410.3816807 | 573.5641617 | 752.7041244 | 943.2806799 | 1080.494823 | 2045.440597 | 972.0096173 | 630.3815497 | 302.1609146 | 7190.931343 |
| Suriname | 0.670351225 | 0.685907596 | 1.051257654 | 1.30369336 | 1.842152631 | 2.114707453 | 3.126433662 | 5.474857309 | 8.267185566 | 10.49388617 | 11.95414999 | 13.49747394 | 16.16810664 | 38.37853605 | 16.8638247 | 12.20394752 | 5.723098801 | 116.6942812 |
| Eswatini | 2.499057765 | 2.606500415 | 3.555516094 | 4.920765142 | 6.957390013 | 10.21492201 | 13.85054724 | 18.36431093 | 25.16041457 | 30.13197449 | 42.82594428 | 55.06387632 | 47.6992607 | 49.74975485 | 31.13416738 | 13.40551579 | 4.215849666 | 319.4826786 |
| Sweden | 1.51530337 | 1.697647891 | 2.392226423 | 2.824684758 | 3.317179266 | 5.126852849 | 10.93745345 | 25.61747144 | 58.48519294 | 126.9886527 | 273.0020436 | 546.215518 | 833.3072578 | 2584.431968 | 865.0954053 | 808.6361985 | 576.5368982 | 4477.785478 |
| Switzerland | 0.908381822 | 1.289077604 | 1.511197188 | 2.048963341 | 3.050463857 | 4.963185207 | 10.65439392 | 28.40910708 | 60.73587368 | 121.6915812 | 191.7129553 | 331.6558001 | 473.5694784 | 1841.122723 | 578.68672 | 600.6351637 | 422.2673677 | 3074.260923 |
| Syrian Arab Republic | 42.19817888 | 26.87579884 | 14.29872722 | 19.16854061 | 35.12709537 | 48.7530606 | 90.7889139 | 153.4736652 | 229.9764116 | 330.4207418 | 406.964886 | 425.5272698 | 460.4941093 | 886.3972555 | 430.4218919 | 353.1712787 | 85.72930837 | 3249.08214 |
| Taiwan (Province of China) | 2.427237481 | 4.049005144 | 7.799097264 | 11.35400845 | 21.93550162 | 35.56679951 | 55.29363485 | 97.68449018 | 176.3755216 | 333.5142017 | 559.9725141 | 781.7248722 | 1265.945971 | 6612.718593 | 1949.299042 | 2149.508016 | 1552.508411 | 9968.914729 |
| Tajikistan | 7.953106352 | 16.20242946 | 23.67529893 | 34.50074046 | 39.50978469 | 44.22136188 | 58.7584057 | 93.28353059 | 162.3354468 | 204.5291268 | 225.7697935 | 185.7000256 | 204.311854 | 353.6472983 | 199.013769 | 100.6032032 | 42.38356945 | 1669.253798 |
| United Republic of Tanzania | 71.2054881 | 88.82695043 | 79.02175861 | 97.97325332 | 124.3140193 | 153.4988445 | 210.5013513 | 296.388785 | 476.7985962 | 635.4881569 | 787.4625019 | 939.6612008 | 959.6886249 | 1573.00816 | 893.7462279 | 427.9158113 | 197.2814743 | 7001.098108 |
| Thailand | 38.08251214 | 56.26361246 | 86.63832807 | 132.4882111 | 199.9724843 | 262.373513 | 377.5792542 | 599.5096321 | 865.1988291 | 1408.018197 | 1946.391191 | 2819.516153 | 3579.331606 | 11496.93887 | 4386.911105 | 3572.373309 | 2229.98858 | 23924.78025 |
| Bahamas | 0.235877296 | 0.37611106 | 0.57785459 | 0.721416044 | 1.001923529 | 1.269798481 | 1.773314432 | 2.76411873 | 3.786513051 | 4.776520042 | 5.454387691 | 5.997098584 | 6.775096447 | 14.91545689 | 6.181402458 | 4.394410186 | 2.757543869 | 50.84084563 |
| Gambia | 5.370759296 | 6.905786477 | 6.719986766 | 7.659219879 | 9.605755126 | 12.10261879 | 15.71675008 | 19.8316359 | 26.85220375 | 37.05071204 | 46.79354813 | 50.87746482 | 63.55920627 | 105.4945813 | 56.31949361 | 34.07563807 | 12.0958854 | 423.8847798 |
| Timor-Leste | 3.3512261 | 3.528769055 | 3.180913879 | 3.117793744 | 3.663966639 | 5.700177994 | 9.73481434 | 14.1703346 | 21.58527108 | 47.89706522 | 79.01219806 | 93.24600729 | 83.81019975 | 131.4789874 | 66.47701431 | 46.05823419 | 15.11130404 | 515.6874123 |
| Togo | 16.3960549 | 21.81219469 | 24.43010242 | 30.56887417 | 38.08963493 | 53.72998378 | 72.70506007 | 88.31783943 | 114.882509 | 137.3368298 | 167.9239255 | 159.0753997 | 162.0008565 | 210.2782953 | 112.1573091 | 64.53608714 | 25.8689778 | 1331.912687 |
| Tonga | 0.209853991 | 0.21934642 | 0.246228524 | 0.332240838 | 0.521639621 | 0.669004737 | 0.900113327 | 1.258849827 | 1.591269988 | 2.230327641 | 3.631520592 | 5.300238949 | 6.781923799 | 17.76941994 | 7.413216268 | 5.753907326 | 3.185235771 | 41.96963218 |
| Trinidad and Tobago | 1.063677771 | 1.348708838 | 2.018483173 | 3.013124733 | 3.828789557 | 4.079946318 | 5.923605388 | 9.126181984 | 15.74389123 | 23.58995889 | 31.89147832 | 38.90802476 | 37.73372474 | 90.35342523 | 35.50967505 | 27.89782705 | 16.17885996 | 271.2150225 |
| Tunisia | 4.094985989 | 5.413185438 | 8.716082361 | 14.94352011 | 21.98929576 | 31.22938814 | 43.65355126 | 67.8804919 | 123.0501039 | 193.0262879 | 272.696164 | 340.2499746 | 417.8849791 | 1095.9027 | 481.3284711 | 375.1740804 | 172.0370714 | 2649.248347 |
| Turkey | 26.83071891 | 29.51396421 | 44.63965959 | 61.18476015 | 101.593927 | 189.6799802 | 344.7650123 | 691.1853375 | 1286.640151 | 2198.195355 | 3298.081293 | 4988.705056 | 5194.769354 | 14471.52314 | 5803.621755 | 5178.070416 | 2582.122789 | 32980.76592 |
| Turkmenistan | 2.590859166 | 5.04958577 | 7.425249354 | 9.567610798 | 11.92025047 | 15.19242497 | 18.7595931 | 24.08077979 | 37.21053011 | 44.8705232 | 47.08483636 | 39.76380319 | 49.1874154 | 126.5794141 | 56.26862896 | 38.50842585 | 22.91389943 | 442.1951106 |
| Uganda | 82.81913356 | 102.599726 | 82.73298773 | 95.4995107 | 105.9048344 | 126.0519164 | 173.3251011 | 233.1305334 | 379.5504594 | 502.9823421 | 594.019233 | 772.5339274 | 768.2998059 | 1118.892039 | 661.405176 | 291.9603301 | 129.9978373 | 5480.309812 |
| Ukraine | 8.353747246 | 14.89399234 | 31.1739798 | 59.13671431 | 98.57959119 | 149.029337 | 237.9817674 | 388.7700468 | 725.8437028 | 1216.637881 | 1501.498287 | 1500.606966 | 1738.768425 | 4505.326657 | 2307.236697 | 1317.281753 | 713.2653182 | 12181.14069 |
| United Arab Emirates | 5.407793077 | 7.594410603 | 17.66020666 | 131.8015743 | 200.1165559 | 276.6420101 | 299.8315082 | 270.2383239 | 172.6452461 | 158.2518011 | 110.885854 | 46.24108123 | 31.4655392 | 45.68841592 | 27.03350431 | 13.61703838 | 4.363200088 | 1777.866015 |
| United Kingdom | 19.66879305 | 26.87028684 | 34.36272938 | 46.66015947 | 67.76680688 | 107.4756179 | 221.0622601 | 548.7024593 | 1067.150496 | 2069.056816 | 3598.536675 | 6797.717369 | 8346.970901 | 28896.5827 | 10131.56374 | 9549.610943 | 6422.736755 | 51878.55215 |
| United States of America | 107.1146761 | 143.9223233 | 196.8130891 | 283.8328298 | 422.2312016 | 729.7987397 | 1585.835127 | 3820.871403 | 8355.280816 | 15032.83158 | 22980.88281 | 33882.06707 | 37101.23321 | 100086.2551 | 36321.74929 | 31124.8031 | 21036.10761 | 224987.73 |
| Uruguay | 2.308116468 | 3.507488664 | 3.39201585 | 4.744337982 | 7.00846423 | 10.69284776 | 19.98682739 | 37.51310174 | 76.50343097 | 128.4984671 | 188.8603918 | 253.2721545 | 311.3964957 | 1102.974197 | 380.0158982 | 346.6202771 | 235.9094449 | 2154.628796 |
| Uzbekistan | 17.2890376 | 35.26550231 | 56.83781063 | 83.86966601 | 96.15809963 | 122.7996255 | 158.8224652 | 250.2363393 | 401.1929002 | 585.1475922 | 549.928977 | 455.5544144 | 551.2681643 | 463.7500906 | 343.4988369 | 98.77181124 | 19.60561427 | 3836.507952 |
| Vanuatu | 1.664885898 | 2.320804288 | 3.120930147 | 4.2128388 | 5.502179192 | 6.194450678 | 7.331034157 | 9.142262304 | 11.17652375 | 16.25003569 | 19.45084117 | 23.19381185 | 26.06070202 | 33.81830112 | 19.96842768 | 10.18550273 | 3.039179835 | 171.9951493 |
[truncated: 58,459 more chars]
